# Influence of the Nucleophilic Ligand on the Reactivity of Carbonyl Rhenium(I) Complexes towards Methyl Propiolate: A Computational Chemistry Perspective

**DOI:** 10.3390/molecules25184134

**Published:** 2020-09-10

**Authors:** Daniel Álvarez, Elena López-Castro, Arturo Guerrero, Lucía Riera, Julio Pérez, Jesús Díaz, M. Isabel Menéndez, Ramón López

**Affiliations:** 1 Departamento de Química Física y Analítica, Universidad de Oviedo, C/Julián Clavería 8, 33006 Oviedo, Asturias, Spain; alvarezldaniel@uniovi.es (D.Á.); elena.castro.lpz@gmail.com (E.L.-C.); uo244375@uniovi.es (A.G.); isabel@uniovi.es (M.I.M.); 2 Centro de Investigación en Nanomateriales y Nanotecnología (CINN), CSIC-Universidad de Oviedo-Principado de Asturias, Avenida de la Vega 4-6, 33940 El Entrego, Spain; l.riera@cinn.es (L.R.); japm@uniovi.es (J.P.); 3 Departamento de Química Orgánica e Inorgánica, Facultad de Química, Universidad de Oviedo, C/Julián Clavería 8, 33006 Oviedo, Spain; 4 Departamento de Química Orgánica e Inorgánica, Universidad de Extremadura, Avenida de la Universidad s/n, 10071 Cáceres, Extremadura, Spain; jdal@unex.es

**Keywords:** organometallic chemistry, rhenium complexes, activated alkynes, computational chemistry, reaction mechanisms

## Abstract

A comparative theoretical study on the reactivity of the complexes [ReY(CO)_3_(bipy)] (Y = NH_2_, NHMe, NH*p*Tol, OH, OMe, OPh, PH_2_, PHMe, PMe_2_, PHPh, PPh_2_, PMePh, SH, SMe, SPh; bipy = 2,2′-bipyridine) towards methyl propiolate was carried out to analyze the influence of both the heteroatom (N, O, P, S) and the alkyl and/or aryl substituents of the Y ligand on the nature of the product obtained. The methyl substituent tends to accelerate the reactions. However, an aromatic ring bonded to N and O makes the reaction more difficult, whereas its linkage to P and S favour it. On the whole, ligands with O and S heteroatoms seem to disfavour these processes more than ligands with N and P heteroatoms, respectively. Phosphido and thiolato ligands tend to yield a coupling product with the bipy ligand, which is not the general case for hydroxo, alcoxo or amido ligands. When the Y ligand has an O/N and an H atom the most likely product is the one containing a coupling with the carbonyl ligand, which is not always obtained when Y contains P/S. Only for OMe and OPh, the product resulting from formal insertion into the Re-Y bond is the preferred.

## 1. Introduction

**Scheme 1:**
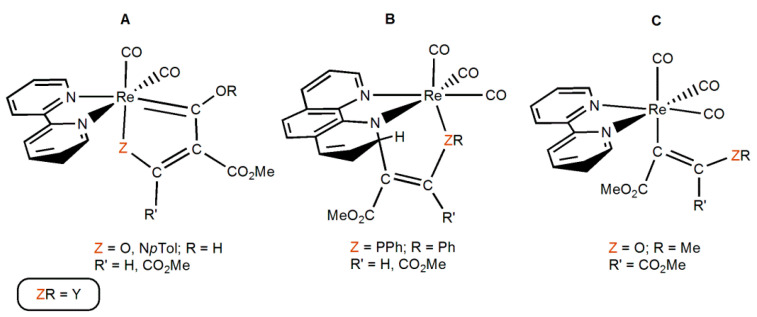
Types of products experimentally obtained in the reaction of [ReY(CO)_3_(N-N)] (Y = NH*p*Tol, OH, OMe, PPh_2_; N-N = bipy and/or phen) with activated alkynes and herein denoted as **A** (**P*ccoh***), **B** (**P*ccb***), and **C** (**P*ins***).

Rhenium(I) *fac*-tricarbonyl complexes bearing conjugated diimine bidentate ligands (i.e., bipyridines, phenanthrolines, etc.) and neutral or anionic monodentate ligands (e.g., halides, pyridines, aqua, phosphines, alkyls, etc.) are functional molecules with applications in several important areas, such as catalytic reduction of CO_2_ [1,2,3,4,5,6,7,8,9,10,11,12,13,14,15,16], luminescence [17,18,19,20,21,22,23,24,25,26,27,28,29,30], medicinal chemistry [31,32,33,34,35,36,37,38,39,40,41,42,43,44,45,46], supramolecular chemistry [47], etc. We reported, among others, the synthesis of the complexes [ReY(CO)_3_(N-N)] (Y = alkoxo [48,49], amido [50,51], hydroxo [52], phosphido [53,54], thiolato [55]; N-N = 2,2′-bipyridine (bipy) and/or 1,10 phenanthroline (phen)). The Y ligand in these complexes bears one or more lone electron pairs. Due to their filled *d*^6^ electron configuration, the Y ligands cannot act as π-donors in these complexes (see Supporting Information for more details). On the other hand, the kinetically inert character of these *d*^6^ third row complexes prevents the formation of Y-bridged polynuclear species. These new compounds showed different reactivity patterns when reacting towards activated alkynes such as methyl propiolate (HC≡CCO_2_Me = HMAD) and/or dimethylacetylenedicarboxylate (MeO_2_CC≡CCO_2_Me = DMAD). The first step is common and consists in a nucleophilic attack from the heteroatom (O, N, P) of the Y ligand onto an acetylene carbon atom, generating a zwitteronic species, which evolves to afford different types of products [56]. Specifically, the Re complexes with Y = NH*p*Tol (*para*-tolylamido) and OH (hydroxo) led to the formation of a coupling product with one of the carbonyl ligands in *cis* disposition to Y. This carbonyl oxygen is protonated while the N and O atoms of the Y ligand have lost their respective hydrogen atoms (see **A** in Scheme 1, **P*ccoh*** products). It is thought that there is another stable species prior to the formation of **P*ccoh***, denoted as **P*cco***, where the carbonyl oxygen is not protonated while the N and O atoms of the Y ligand still display the hydrogen atom [51,52]. A coupling product with one of the non-subtituted *ortho* carbons of the bidentate ligand was obtained for the Re complex containing the diphenylphosphanido (PPh_2_) ligand (see **B** in Scheme 1, **P*ccb*** products) [53,54]. Finally, for the Re methoxo (OMe) complex, the product obtained corresponds to the alkyne insertion into the Re-OMe bond (see **C** in Scheme 1, **P*ins*** products) [48].

Our theoretical investigations on the reaction between the complexes [ReY(CO)_3_(bipy)] (Y = NH*p*Tol, OH, OMe, PPh_2_) and HMAD uncovered that the reaction rate is, on the whole, controlled by the first stage of the reaction mechanism, that is, the nucleophilic attack of the Y ligand on the non-substituted alkyne carbon [57,58]. Nonetheless, the subtle balance between kinetics and thermodynamics of the formation step of the plausible products determines the type of product formed. While this detailed investigation was relevant to understand these processes, an overlap of different factors in the nucleophilic Y ligand complicates a clear rationalization of the reactivity trends and therefore hinders the attempts to obtain the relevant information for developing new rhenium complexes. Two simultaneous factors are present in the Y ligand of the Re complex, namely the donor atom directly linked to Re (O, N, P) and the nature of its substituents (one or two hydrogen, alkyl, aryl groups). For instance, we noted that the results obtained for the Re hydroxo and methoxo complexes allow us to understand the effect of an alkyl substituent, but not the effect of an aryl one. By contrast, the comparison between the NH*p*Tol and PPh_2_ cases is not reliable to rationalize either the effect of an aryl group or the effect of the donor atom directly linked to Re. The situation is even worse if we wanted to extract chemical trends when comparing the four cases mentioned above at the same time. The results obtained for the reactivity of the [Re(PPh_2_)(CO)_3_(bipy)] complex are not comparable with those found for [ReY(CO)_3_(bipy)] (Y = OH, OMe) because there are different substituents and donor atoms belonging to different groups of the Periodic System.

Therefore, to get more general conclusions about these processes, ultimately aiming at tuning the Re complexes in order to obtain more interesting synthetic, industrial or biochemical applications [59,60], a more systematic computational investigation is needed. To accomplish this task, we undertook a theoretical study on the reaction mechanism of the reactivity towards the substrate HMAD of the complexes [ReY(CO)_3_(bipy)] (Y = NH_2_, NHMe, OPh, PH_2_, PHMe, PMe_2_, PHPh, PMePh, SH, SMe, SPh). The theoretical results obtained on these hypothetical reactions along with those previously found for the reactivity between the complexes [ReY(CO)_3_(bipy)] (Y = NH*p*Tol, OH, OMe, PPh_2_) and HMAD may be beneficial in gaining a broader understanding of the effect of Y ligand focusing on the alkyl and aryl substituents and the heteroatom directly bonded to Re.

## 2. Results

The presentation and analysis of the results obtained for the reaction between the 15 complexes [ReY(CO)_3_(bipy)] (Y = NH_2_, NHMe, NH*p*Tol, OH, OMe, OPh, PH_2_, PHMe, PMe_2_, PHPh, PPh_2_, PMePh, SH, SMe, SPh) and HMAD will be done as follows. First, we will report a general description of the types of potential energy surfaces found taking into consideration our DLPNO-CCSD(T) data. Second, we will analyze the effect of alkyl and aryl substituents at the nucleophilic ligand Y on the energetics of the reactive processes investigated. The same objective will be pursued when analyzing the influence of replacing, within Groups 15 and 16, the heteroatom of the second period by its counterpart of the third period (i.e., P instead of N). Finally, we will provide a general discussion by considering all the results mentioned in the previous subsections.

### 2.1. General Description of the Potential Energy Surfaces

**Scheme 2:**
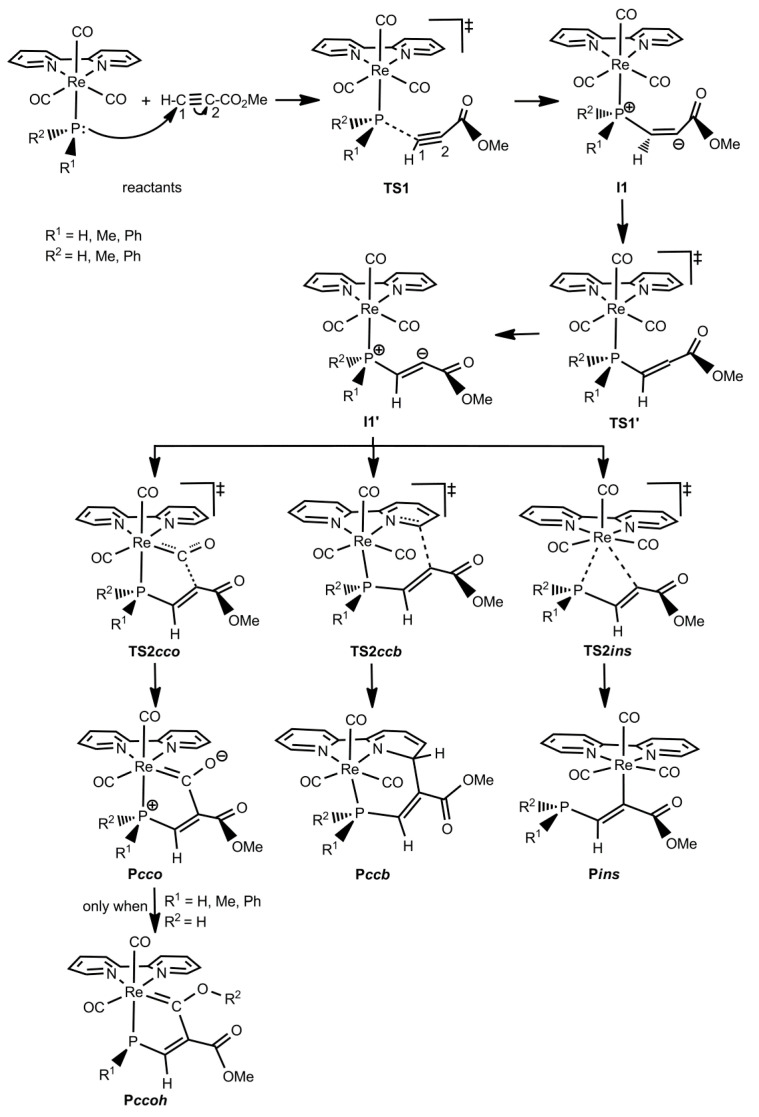
Schematic structures of the species located on the type-I potential energy surfaces (PES) found for the reaction of the complexes [ReY(CO)_3_(bipy)] (Y = PR^1^R^2^ with R^1^ = R^2^ = H, Me, Ph) towards HMAD. The connectivities among these species are also shown with arrows. Acetylenic carbon atoms of HMAD are numbered both in the isolated reactant and in **TS1**. Analogous structures were found for the Re complex with Y = OH.

**Scheme 3:**
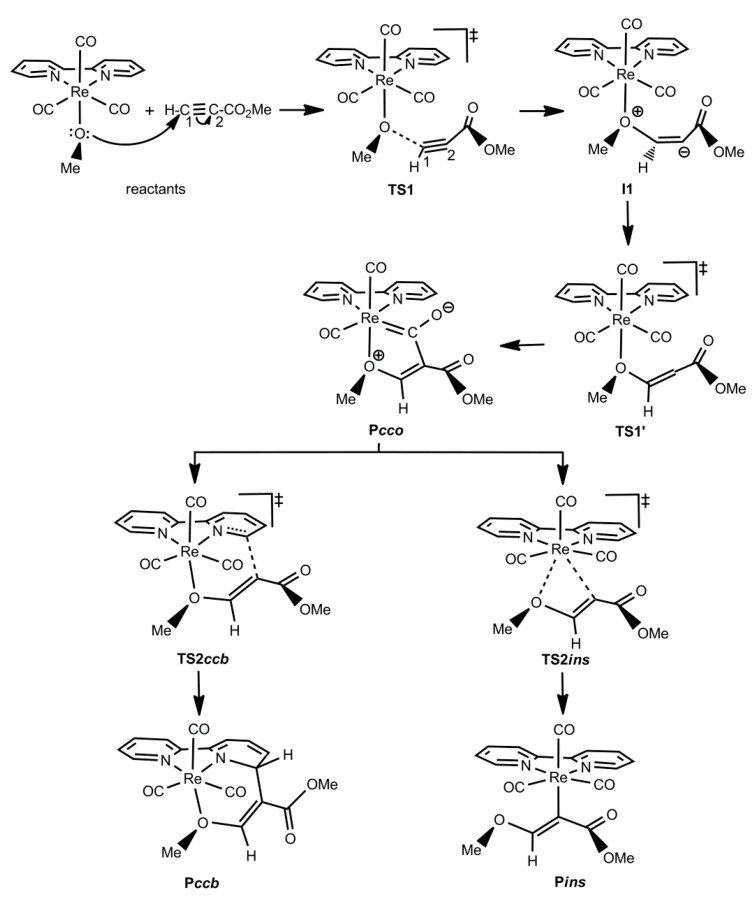
Schematic structures of the species located on the type-II PES found for the reaction of the complex [Re(OMe)(CO)_3_(bipy)] towards HMAD. The connectivities among these species are also shown with arrows. Acetylenic carbon atoms of HMAD are numbered both in the isolated reactant and in **TS1**.

**Scheme 4:**
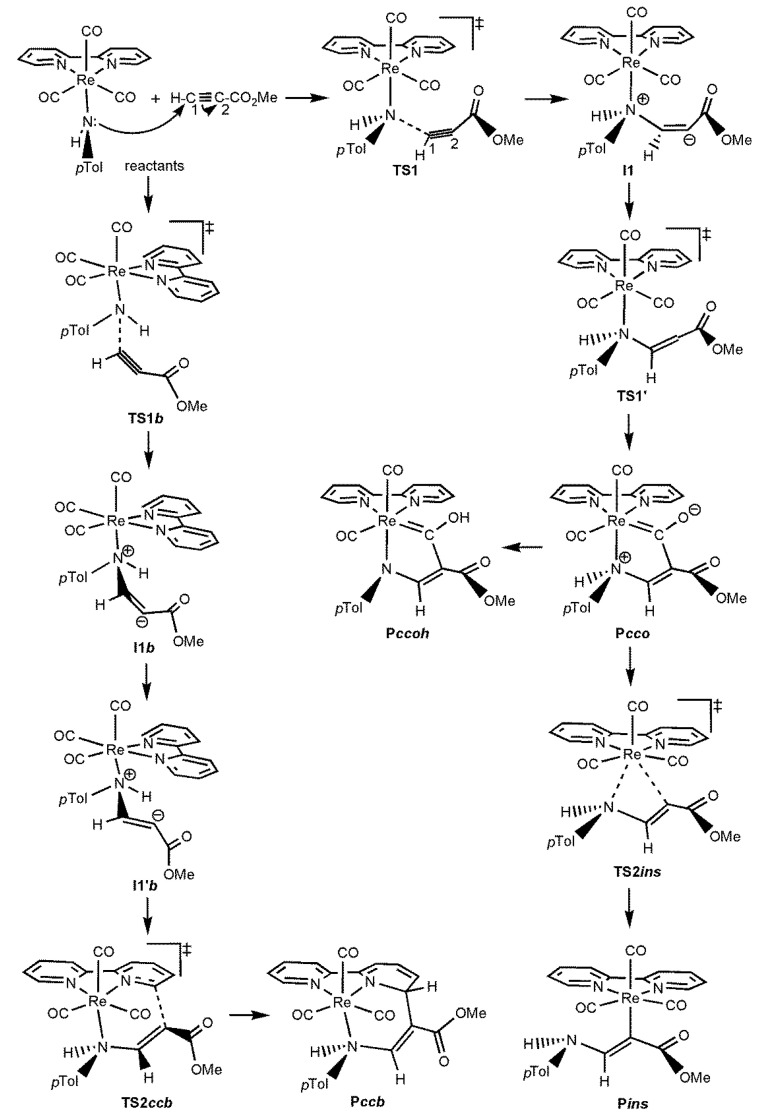
Schematic structures of the species located on the type-III PES found for the reaction of the complex [Re(NH*p*Tol)(CO)_3_(bipy)] towards HMAD. The connectivities among these species are also shown with arrows. Acetylenic carbon atoms of HMAD are numbered both in the isolated reactant and in **TS1**.

**Figure 1:**
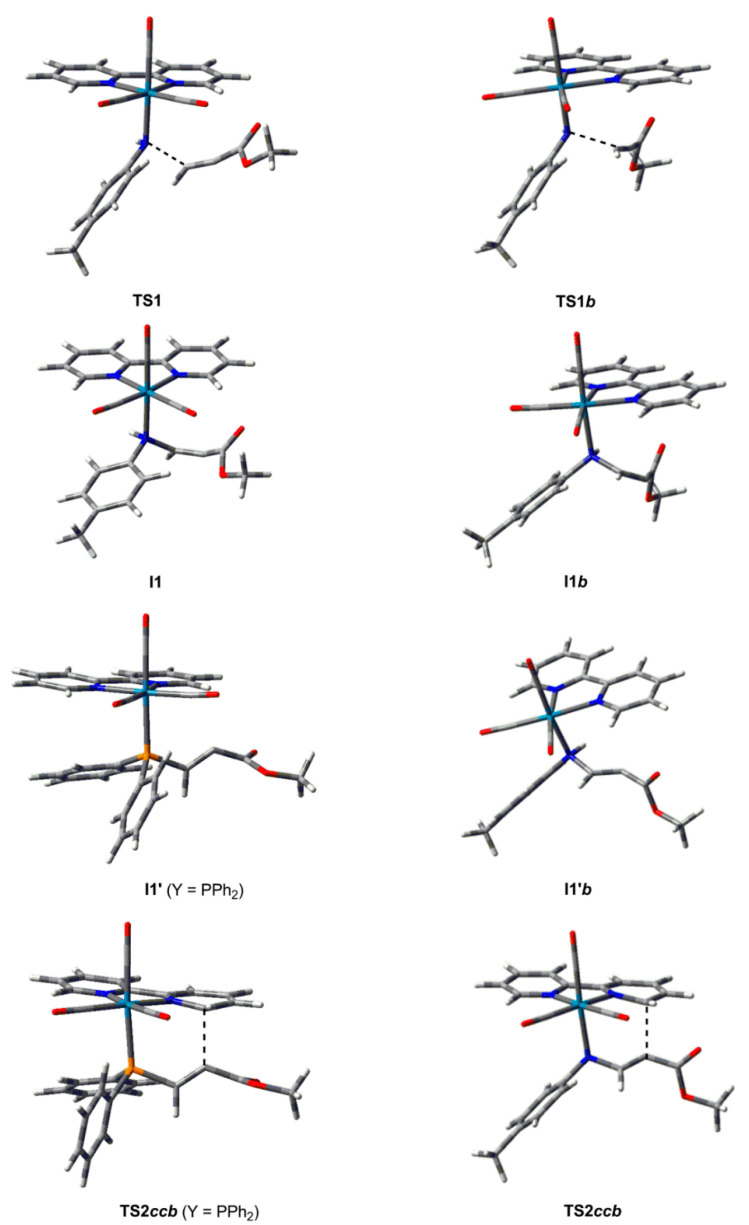
B3LYP optimized structures of some species located for the reaction of the complex [Re(NH*p*Tol)(CO)_3_(bipy)] towards HMAD. B3LYP optimized structures of **I1′** and **TS2*ccb*** for Y = PPh_2_ are also given for comparison purposes.

As just mentioned in the Introduction section, experimental studies on the reactivity of the complexes [ReY(CO)_3_(N-N)] (Y = NH*p*Tol, OH, OMe, PPh_2_; N-N = bipy and/or phen) towards activated alkynes (HMAD and/or DMAD) have shown the formation of three types of products (see Scheme 1) [48,51,52,53,54]. Theoretical investigations using the bipy ligand and the HMAD activated alkyne have not only corroborated the formation of such products, but also shown all the species involved in the reaction mechanisms leading to the **P*cco***/**P*ccoh***, **P*ccb***, and **P*ins*** products [57,58]. Looking at the reaction mechanisms involved, three different patterns of potential energy surfaces (PES) were found [58]. The type-I PES found for Y = OH and PPh_2_ starts with the nucleophilic attack of the Y ligand on the non-substituted acetylenic carbon of HMAD (C1) through the TS **TS1** to give a zwitterionic intermediate (**I1**), in which a new HO/Ph_2_P-C1 bond is formed and, consequently, the original triple bond between C1 and the substituted acetylenic carbon of the HMAD fragment (C2) presents now a double bond character (see Scheme 2). Evolving via **TS1′**, **I1** isomerizes into another intermediate (**I1′**) wherein C2 is readily oriented to yield any of the products. **I1′** is the common precursor species of **P*cco***/**P*ccoh***, **P*ccb***, and **P*ins*** via the TS **TS2*cco***, **TS2*ccb***, and **TS2*ins***, respectively (see Scheme 2). For OH, **P*cco*** evolves to **P*ccoh*** through an intermolecular transformation with a Gibbs energy barrier lower than that found for the nucleophilic attack step [58]. This fact along with the greater stability of **P*ccoh*** over **P*cco*** explains the formation of the former product as experimentally observed [52]. A different PES named as type II was found for OMe (see Scheme 3). Reactants evolve to **I1** via **TS1**, which in turn directly transforms into **P*cco*** via **TS1′**. **P*cco*** is now the species from which the products **P*ccb*** and **P*ins*** are formed via **TS2*ccb*** and **TS2*ins***, respectively. All these species show structures analogous to those identified with the same acronyms on the type-I PES. The type-III PES was obtained for the NH*p*Tol case wherein two splitting species were found, the first one being the reactants (see Scheme 4). On the one hand, reactants may become **I1**, first, and then **P*cco*** passing through **TS1** and **TS1′**, respectively. **P*cco*** can lead to either **P*ccoh*** as in the OH case [58] or **P*ins*** via **TS2*ins***. On the other hand, reactants may proceed through **TS1*b*** for the attack of the NH*p*Tol ligand on the C1 atom of the alkyne fragment, which is oriented in the opposite direction to that presented at **TS1** or **TS1′** (see Scheme 4 and Figure 1). **TS1*b*** evolves to the corresponding intermediate **I1*b***, in which the new H*p*TolN-C1 bond is just established. **I1*b*** becomes an isomer **I1′*b*** without any TS to finally give **P*ccb*** via **TS2*ccb***. **I1′*b*** mainly differs from **I1*b*** in the orientation of the C2-bonded CO_2_Me group of the alkyne moiety. This group and the C1-bonded H atom are on opposite sides of the C1=C2 double bond at **I1*b***, while at **I1′b** they are on the same side to favour the attack of C2 on the bipy ligand (see Scheme 4 and Figure 1). All the species implied in the reaction mechanism for the formation of **P*ccoh*** and **P*ins*** display structures analogous to those denoted with the same acronyms on the type-I and II PES. However, for the reaction mechanism leading to **P*ccb***, the geometry discrepancy mentioned above between **TS1*b*** and **TS1** or **TS1′** is also present when comparing **I1*b*** and **I1′*b*** with **I1** and **I1′** (when located for OH and PPh_2_), respectively (see Figure 1). In addition, we also note that the plane defined by the heteroatom of the NH*p*Tol ligand, C1, and C2 is practically perpendicular to the plane defined by Re and the bipy ligand at **I1′**, while an almost parallel disposition between these planes was located at **I1′*b***. A similar geometry difference was detected when comparing the TS **TS2*ccb*** located for OH and PPh_2_ with the one found for NH*p*Tol (see, for instance, **TS2*ccb*** for PPh_2_ and NH*p*Tol in Figure 1). Intermediates analogous to **I1*b*** and/or **I1′*b*** were also located when Y = OH, OMe, and PPh_2_, but **TS2*ccb*** connects **P*ccb*** with **I1′** (**P*cco*** for the OMe case) instead of **I1*b*** and/or **I1′*b*** [58].

**Scheme 5:**
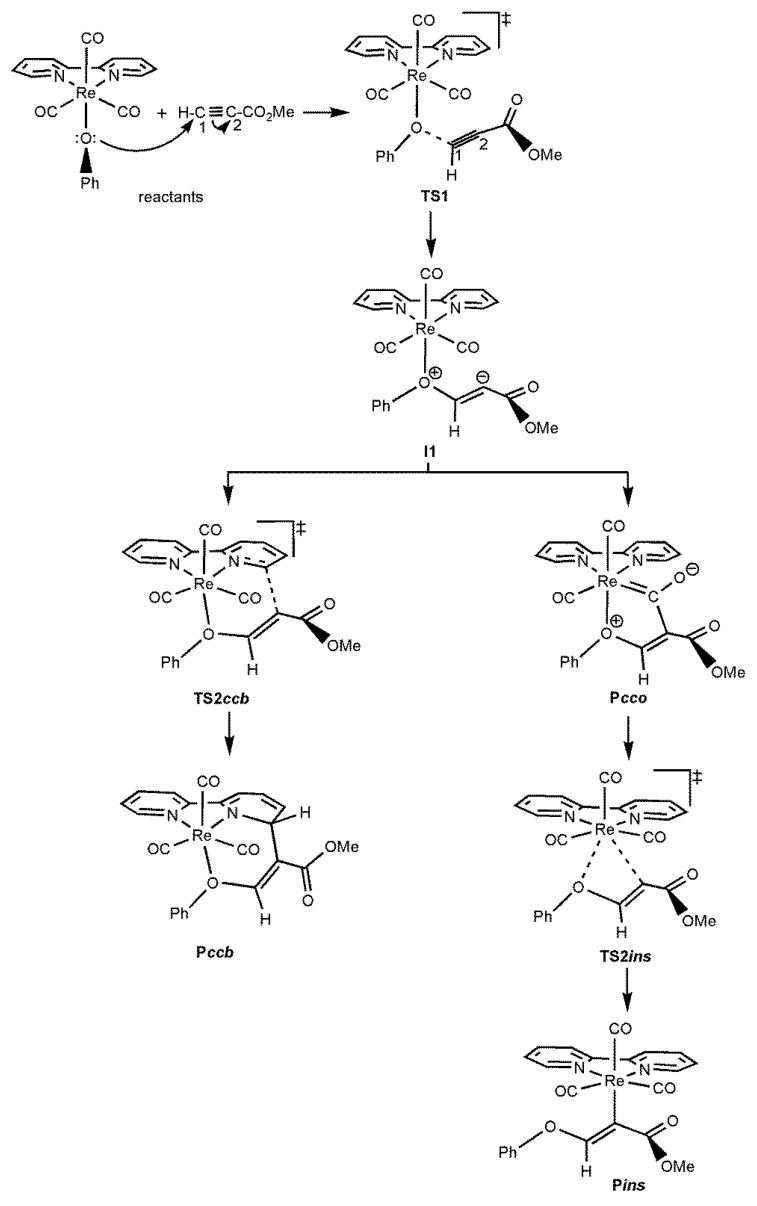
Schematic structures of the species located on the type-IV PES found for the reaction of the complex [Re(OPh)(CO)_3_(bipy)] towards HMAD. The connectivities among these species are also shown with arrows. Acetylenic carbon atoms of HMAD are numbered both in the isolated reactant and in **TS1**.

With all of this in mind, we extended our theoretical mechanistic investigation by considering the new nucleophilic ligands NH_2_, NHMe, OPh, PH_2_, PHMe, PMe_2_, PHPh, PMePh, SH, SMe, and SPh. All the geometry and energy data of the species located are collected in the Supporting Information (Figures S1–S11 and Tables S1–S33). For comparison purposes, data previously reported for the analogous Re complexes with Y = NH*p*Tol, OH, OMe, and PPh_2_ have also been included in the Supporting Information (Figures S12–S15 and Tables S34–S45). All the PES found can be grouped according to the number of splitting species and the type of the first splitting species. First, all the Re complexes with a nucleophilic ligand containing a phosphorus atom directly linked to the metal center present the type-I PES as **I1′** is the only splitting species (see Scheme 2). All the optimized structures for the five ligands with P and denoted with the same acronyms as in Scheme 2 are analogous to those previously reported for the PPh_2_ case [58]. The PES obtained for the nucleophilic ligands NH_2_, NHMe, SH, SMe, and SPh resemble the type III (see Scheme 4), since a separate channel to **P*ccb*** is found from the reactants, although with some differences among them. Particularly, for SH and SPh **I1** (instead of **P*cco***) is the second splitting species that connects reactants with **P*cco*** (**P*ccoh*** for SH) and **P*ins***. **T**he transformations reactants → **I1**, **I1** → **P*cco***/**P*ccoh***, and **I1** → **P*ins*** are controlled by **TS1**, **TS2*cco***, and **TS2*ins***, respectively. For NH_2_, NHMe, and SMe **I1′** (instead of **P*cco***) is the second splitting species, which in turn may transform into **P*cco*** (**P*ccoh*** for NH_2_ and NHMe) and **P*ins***. The evolution to these products from the reactants is the same as that found for the generation of **P*cco***/**P*ccoh*** and **P*ins*** with the OH and PR^1^R^2^ (R^1^ = R^2^ = H, Me, Ph) ligands (see Scheme 2). Despite these differences in the type-III PES obtained, all the species implied in the reaction mechanism leading to **P*cco***/**P*ccoh*** and **P*ins*** with Y = NH_2_, NHMe, SH, SMe, and SPh show optimized structures similar to those identified with the same acronyms for NH*p*Tol, OH, OMe, and PPh*_2_* (see Scheme 2, Scheme 3 and Scheme 4), except **I1** when Y = SH and SPh, whose respective optimized structures are analogous to those found for the type-**I1′** species (see Figure 1). For the reaction mechanism leading to **P*ccb*** when Y = NH_2_, NHMe, SH, SMe, and SPh, the optimized structures of all the species found (**TS1*b***, **I1*b***, **I1′*b***, **TS2*ccb***, and **P*ccb***) are similar to those of their analogues when Y = NH*p*Tol (see Scheme 4 and Figure 1). Finally, the OPh case uncovers a new mode of PES, type IV, where reactants proceed through **TS1** to **I1** (see Scheme 5). This intermediate resulting from the attack of the nucleophilic ligand on the HMAD non-substituted carbon is the splitting species now and can evolve either to **P*cco*** without any barrier to subsequently transform into **P*ins*** via **TS2*ins*** or to **P*ccb*** via **TS2*ccb***. As for SH and SPh, the optimized structure of **I1** resembles that of **I1′** when Y = NH_2_, NHMe, OH, PR^1^R^2^, and SMe. The remaining species found on the type-IV PES display structures similar to those denoted with the same acronyms in Scheme 2, Scheme 3 and Scheme 4.

A general view of Scheme 2, Scheme 3, Scheme 4 and Scheme 5 shows that only those Y ligands with N and S atoms display the alternative route reactants → **TS1*b*** → **I1*b*** → **I1′*b*** → **TS2*ccb*** → **P*ccb***. While **I1*b*** and/or **I1′*b*** are expected to exist for PH_2_, PHMe, PMe_2_, PHPh, PMePh, and OPh, computations show that **TS2*ccb*** connects **P*ccb*** with **I1′** for the five ligands containing the phosphorus atom as for the OH and PPh_2_ (see Scheme 2) and with **I1** when Y = OPh (see Scheme 5). Besides, OMe and OPh ligands are the only ones whose PES do not present intermediate **I1′**. This is a consequence of the geometry of their **I1** intermediates, whose lone pair at the C2 atom of HMAD is quite close to the electrophilic positions in the equatorial ligands, which avoid the existence of an intermediate such as **I1′** with that lone pair ready but not reacting with these positions.

### 2.2. Effect of the Alkyl and Aryl Substituents of the Nucleophilic Ligand

The substitution of one of the hydrogen atoms in the NH_2_ ligand by a methyl group causes a relative stabilization of all the species between 1.3 and 7.4 kcal/mol, except **I1**, which slightly destabilizes by 0.8 kcal/mol (see Table S46). It is interesting to note that such a replacement relatively stabilizes **TS1*b*** from 19.3 to 14.0 kcal/mol whereas **TS1** only changes from 18.5 kcal/mol to 17.2 kcal/mol when going from NH_2_ to NHMe (see Table 1). Since **TS1** and **TS1*b*** are the rate limiting steps for both alternative routes, the presence of the methyl substituent would change the preferred product from **P*ccoh*** (−24.9 kcal/mol) for NH_2_ to **P*ccb*** (−19.3 kcal/mol) for NHMe without changing the kind of PES (type-III). The replacement of one of the hydrogen atoms in the NH_2_ ligand by an aryl substituent (pTol), as in NHpTol, shows the opposite effect since all the species relatively destabilize between 2.1 and 14.8 kcal/mol (see Table S46). As seen in Table 1, the reaction channel controlled by **TS1** leading to **P*cco***/**P*ccoh*** and **P*ins*** is only 1.0 kcal/mol larger in Gibbs energy than that controlled by **TS1*b*** leading to **P*ccb***. The small difference between these determining Gibbs energy barriers and the large stability of **P*ccoh*** (−21.1 kcal/mol) points to **P*ccoh*** as the expected product, as experimentally observed [51].

Let us now consider the case of the OR ligands (R = H, Me, OPh). Taking as reference the relative Gibbs energies of the species found for the OH case and comparing them with those for OMe and OPh (see Table S47), similar trends to those of the NHR’ (R’ = H, Me, pTol) ligands are found. Thus, all the species stabilize between 1.1 and 5.3 kcal/mol and destabilize between 6.5 and 9.0 kcal/mol when replacing OH by OMe and OPh, respectively. The presence of the alkyl substituent provokes a modification of the PES from type-I for OH to type-II for OMe (see Scheme 2 and Scheme 3). Besides, the product observed changes from **P*ccoh*** for OH ligand to **P*ins*** for OMe ligand in good agreement with experimental findings [48,52]. With both OH and OMe ligands **TS1** and **TS1′** compete against each other to be the rate-determining TS of the overall process; **TS1** is 1.0 kcal/mol more stable than **TS1′** for OH, while the reverse situation was found for OMe as **TS1** is 1.1 less stable than **TS1′** (see Table 1). A different situation was found for the OPh ligand. As collected in Table 1, contrary to the case with the OH ligand (yielding the **P*ccoh*** product), the expected product for the OPh ligand would be **P*ins***, since this product is the most stable structure (−5.6 kcal/mol) and the Gibbs energy barrier for its formation (ΔG^≠^(**TS2*ins***) = 36.5 kcal/mol) is lower than that for the formation of the alternative product **P*ccb*** (ΔG^≠^(**TS2*ccb***) = 38.8 kcal/mol). It has to be noted that the large values of both **TS1** (33.5 kcal/mol) and **TS2ins** make the reaction of OPh complex quite unlikely. In addition, the replacement of OH by OPh changes the PES from type-I to type-IV (see Scheme 2 and Scheme 5).

How are the trends described above when the alkyl and aryl substituents are bonded to phosphorus and sulfur atoms instead of nitrogen and oxygen, respectively? We start with ligands containing P and take PH_2_ as the initial reference. As previously noted, all these ligands render the type-I PES (see Scheme 2); in all cases **TS1** is the rate-determining TS and the insertion route is highly penalized due to the large barrier of **TS2*ins*** ranging from 17.5 kcal/mol for Y = PHPh to 34.2 kcal/mol for Y = PH_2_ (see Table 1). **TS2*cco*** and **TS2*ccb***, notably less unstable than **TS2*ins***, have similar Gibbs energy barriers, but **P*ccb*** is always much more stable than **P*cco***/**P*ccoh*** (see Table 1). Despite these similarities, particular features of each substituent deserve some comments. The substitution of the PH_2_ ligand by the PHMe one relatively stabilizes all the structures between 4.6 and 13.1 kcal/mol, except **TS1**, which destabilizes by 3.3 kcal/mol (see Table S48). The alkyl substituent does not change the fact that **TS1** remains the rate-determining TS and **P*ccb*** is the expected product for both ligands (see Table 1). For the case of PMe_2_ the presence of a second methyl group further stabilizes all the species, including **TS1 (**except **TS2*cco*** that is 0.5 kcal/mol larger for PMe_2_ than for PHMe) making this ligand specially adequate to yield **P*ccb***. On the other hand, the substitution of PH_2_ by PHPh also produces a relative stabilization of all the species, including **TS1**, between 3.3 and 16.7 kcal/mol (see Table S48). This trend is contrary to that found for the substitution of one H atom by an aromatic group in the Y ligands containing the N and O donor atoms. The presence of two Ph groups at PPh_2_ futher stabilizes all the species between 0.1 and 7.2 kcal/mol with respect to PHPh, except **TS2*ins*** (17.5 kcal/mol for PHPh and 20.6 kcal/mol for PPh_2_) and **P*ins*** (−17.5 kcal/mol for PHPh and −16.8 kcal/mol for PPh_2_). As for the alkyl substitution cases, the presence of one or two aryl groups bonded to P also points to the formation of **P*ccb*** products due to their large stability (−12.8 and −20.0 kcal/mol for PHPh and PPh_2_, respectively) and the accessible and competitive barriers for their generation (9.4 and 5.0 kcal/mol for PHPh and PPh_2_, respectively). Whithin the accuracy of the computational level used, as seen in Table 1, PMe_2_ and PPh_2_ present very similar Gibbs energies for the TSs for all the reaction routes, being the largest difference that for **TS2*ccb***, which is lower for PMe_2_ (4.3 kcalmol) than for PPh_2_ (5.0 kcal/mol). Besides, all kinds of products are considerably more stable with the PMe_2_ substituent. On the other hand, comparing the PHMe and PHPh cases, we see that the substitution of Me by Ph produces a destabilization of all the species between 0.1 and 4.7 kcal/mol except **TS1** and **TS2*ins***, which stabilize 13.3 and 3.6 kcal/mol, respectively (see Table S48). We have also considered the hybrid ligand PMePh, whose barriers parallel those of PPh_2_ and slightly favour the coupling route with a carbonyl ligand but not as much as to change the preference for the formation of **P*ccb*** (see Table 1).

The three ligands SH, SMe, and SPh have in common the presence of **TS1*b*** (typical of the type-III PES collected in Scheme 4) leading to **P*ccb*** and the fact that the route to **P*ins*** has the largest barrier to yield the most stable product (see Table 1). The energy trends obtained for the SR ligands (R = H, Me, Ph) resemble those found for the PR^1^R^2^ ligands. Indeed, both the alkyl and aryl substituents give rise to a stabilization of all the species, mainly with the Me replacement in SH. In particular, the methyl substitution relatively stabilizes all the species in the gap 6.7−10.6 kcal/mol, whereas a relative stabilization range between 1.9 and 6.7 kcal/mol was obtained for the phenyl substitution (see Table S49). On the whole, the degree of stabilization due to SMe is less than when replacing OH by OMe. For SH, as seen in Table 1, the formation of **P*ccoh*** is preferred, since it has the smallest limiting barrier (32.9 kcal/mol for **TS2*cco***) and shows the largest stability (−2.6 kcal/mol). This image changes when replacing SH by SMe. Now, the barrier for **P*cco*** is still the smallest one (24.7 kcal/mol for **TS2*cco***) followed by the barrier to **P*ccb*** (26.4 kcal/mol for **TS2*ccb***), but **Pcco** is less stable (11.0 kcal/mol) than **P*ccb*** (3.1 kcal/mol), although both products are less stable than their isolated reactants (see Table 1). These reasons make difficult to suggest the preferred product for the SMe case. A similar picture was found for the SPh case in which **TS1** (30.8 kcal/mol) competes with **TS2*ccb*** (31.0 kcal/mol) and both **P*cco*** (14.4 kcal/mol) and **P*ccb*** (8.9 kcal/mol) are higher in energy than their reactants. Actually, the barriers found the SH (~33 kcal/mol) and SPh (~31 kcal/mol) ligands indicate a low reaction probability with these ligands (see Table 1).

The previous analysis shows that ligands NH_2_, OH, PH_2_, and SH are bad nucleophiles (worsening from NH_2_ to SH in the previous list) for the reaction between the corresponding Re complex and HMAD. On the one hand, a methyl group replacing one of the H atoms in these ligands enhances the process, since it reduces the barriers involved. In some cases, this substitution determines a change in the nature of the most favourable product. On the other hand, an aromatic ring bonded to N and O impairs the reaction, whereas when it is a substituent bonded to P and S the reverse effect is observed. Since the π electrons in the aromatic ring are able to conjugate with the lone pair at N atom in the NH*p*Tol ligand this lone pair becomes less available for the nucleophilic attack on the HMAD molecule. The larger size of P prevents this conjugation, which explains the higher reactivity of PHPh compared to that of NH*p*Tol.

### 2.3. Effect of the Heteroatom of the Nucleophilic Ligand

We now focus on the replacement of the heteroatom of the Y ligand when going from the second period to the third one along Groups 15 and 16 of the periodic table. The substitution of the nitrogen atom of NH_2_ by phosphorus in PH_2_ leads to an important relative destabilization of all the species between 5.1 and 25.2 kcal/mol (see Table S50). The rate-determining Gibbs energy barrier determined by **TS1**, goes from 18.5 kcal/mol for NH_2_ to 27.6 kcal/mol for PH_2_ (see Table 1). From NH_2_ to PH_2_, **TS2*cco***, **TS2*ccb***, and **TS2*ins***, increase by 9.2, 3.6, and 23.0 kcal/mol, respectively, being **P*ins*** remarkably kinetically penalized (see Table 1). Besides, the expected product changes from **P*ccoh*** for NH_2_ to **P*ccb*** for PH_2_. When comparing the NHMe and PHMe cases (see Table S50), all the species destabilize between 1.0 and 28.0 kcal/mol, except **I1**, which only stabilizes 0.3 kcal/mol and **TS2ccb** whose relative Gibbs energy changes from 9.6 kcal/mol for NHMe to 5.8 kcal/mol for PHMe (see Table 1). The Gibbs energy barriers for **P*cco***/**P*ccoh*** and **P*ins*** increase by 4.4 and 11.9 kcal/mol, respectively. When Y = NHMe there is a kinetics competition between **P*ccb*** and **P*cco***/**P*ccoh*** formation, as previously explained, whereas for PHMe, **P*ccb*** is clearly the preferred product (see Table 1). By contrast, the comparison of the results for the NHpTol and PHPh cases reflects that all the species stabilize when replacing N by P between 2.9 and 9.6 kcal/mol, except **Pccoh** and **Pins**, which destabilize 16.9 and 4.9 kcal/mol, respectively (see Table S50). Even **TS2*ins*** lowers its barrier, although it is still one of the largest. **P*ccoh*** is the product formed for NHpTol, in good agreement with experimental evidences [51], while **P*ccb*** is expected to be formed when [Re(PHPh)(CO)_3_(bipy)] reacts with HMAD. In the presence of an aromatic ring, its available conjugation with the N atom, which does not occur with the P atom, overcomes the destabilization observed in the two previous cases (see below).

On the other hand, for Group 16, the substitution of OH by SH relatively destabilizes all the species between 4.6 and 11.3 kcal/mol as it was found when replacing NH_2_ by PH_2_ (see Table S51). Again, **TS2*ins*** is the most penalized TS and, in this case, **P*ccoh*** remains as the preferred product for both ligands. On the whole, the replacement of OMe by SMe show trends similar to those found when replacing NHMe by PHMe. Most of the species destabilize between 1.1 and 8.1 kcal/mol, whereas **TS1**, **TS2*ccb***, and **P*ccb***, stabilize by 2.8, 3.1, and 1.8 kcal/mol, respectively (see Table S51). As deduced from Table 1, **P*ins*** becomes again the most kinetically penalized product as found for the PHR (R = H, Me, Ph) ligands. **P*cco*** and **P*ccb*** compete with each other to be the preferred product for SMe while **P*ins*** is the most stable one. In contrast, when replacing OPh by SPh, all the species stabilize between 0.1 and 9.8 kcal/mol. **TS1**, **TS2*ccb***, and **TS2*ins*** (**TS2*cco*** was not located for OPh) become 2.7, 7.8, and 1.7 kcal/mol more stable when going from OPh to SPh, with **TS2*ins*** again the highest in energy (see Table 1 and Table S51). As previously said, for OPh **P*ins*** is the expected product, which changes to a competition between **P*cco*** and **P*ccb*** for SPh, although both OPh and SPh ligands are quite unreactive towards HMAD.

With respect to the heteroatom of the Y ligand directly linked to Re, it is clear that the insertion route is greatly penalized for P and S ligands, although **P*ins*** is very stable. This fact can be atributed to the strong bond formed between these large atoms and Re, which is weaker in the case of N and O atoms, as later explained. This bond needs to be broken for the insertion of the HMAD fragment in the Re-Y bond. Besides, P and S ligands tend to yield **P*ccb*** as the main product, which is not the general case for O and N ones. On the other hand, when the Y ligand has an O/N heteroatom bonded to an H atom the most probable product is **P*ccoh***, which is not always obtained when Y contains P/S.

### 2.4. General Discussion

The investigation on the reaction mechanism of the reactivity of the 15 Re complexes [ReY(CO)_3_(bipy)] (Y = NH_2_, NHMe, NHpTol, OH, OMe, OPh, PH_2_, PHMe, PMe_2_, PHPh, PPh_2_, PMePh, SH, SMe, SPh) towards HMAD displayed a variety of PES. However, all of them can be grouped into four different types on the basis of the number and the first splitting species as collected in Scheme 2, Scheme 3, Scheme 4 and Scheme 5. Whereas all ligands of the PR^1^R^2^ family always present the type-I PES the three ligands of the OR family (R = H, Me, Ph) show three different types of PES named as I, II, and IV, respectively. The remaining Y ligands (NH_2_, NHMe, NHpTol, SH, SMe, SPh), which present two splitting species, have been grouped within the type-III PES.

On the whole, the overall reaction rate is determined by the first stage of the reaction mechanism, that is, the TS for the attack of the nucleophilic Y ligand of the Re complex on HMAD (**TS1** or **TS1*b***, when present). For the sake of generality, we also consider **TS1** as the determinant step for complexes with OH, OPh, and SH ligands, although their **TS1′**, **TS2*ins***, and **TS2*cco*** are only 1.0, 3.0, and 0.2 kcal/mol higher in energy than their **TS1**, respectively (see Table 1).

**Figure 2:**
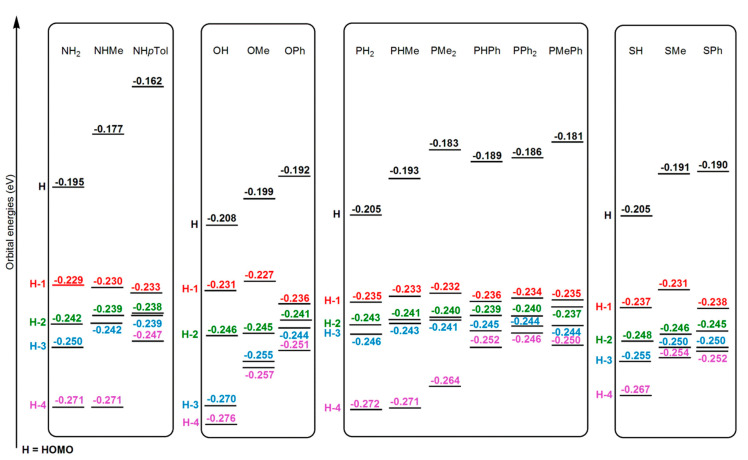
Energies (in eV) of the five highest occupied orbitals in the reactant complexes [ReY(CO)_3_(bipy)] (Y = NH_2_, NHMe, NH*p*Tol, OH, OMe, OPh, PH_2_, PHMe, PMe_2_, PHPh, PPh_2_, PMePh, SH, SMe, SPh).

**Figure 3:**
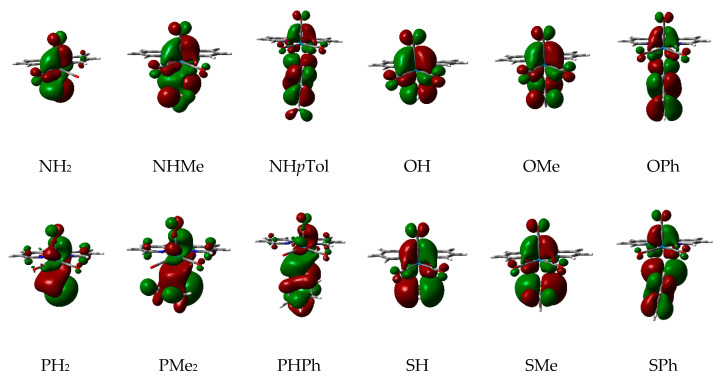
Pictures of the computed HOMO of the reactant complexes [ReY(CO)_3_(bipy)] (Y = NH_2_, NHMe, NH*p*Tol, OH, OMe, OPh, PH_2_, PMe_2_, PHPh, SH, SMe, SPh).

As seen in Table 1 the replacement of a hydrogen atom by a methyl substituent at the NH_2_, OH, and SH as well as the change from the PH_2_ to the PMe_2_ ligands reduces the Gibbs energy barrier controlled by **TS1** by 1.3, 1.1, 9.6, and 10.2 kcal/mol, respectively. It is well-known that the higher the Y-centered HOMO energy of the Re complex, the greater the nucleophilic character of the ligand and, consequently, the lowest Gibbs energy barrier for **TS1**. Accordingly, as seen in Figure 2, the energies of the HOMOs of the complexes with methyl substituents are always higher than those unsubstituted ones. Figure 3 shows how the presence of a methyl substituent adds an extra antibonding π interaction in the Y ligand environment, which explains the instability of the HOMO of these methyl substituted complexes.

For the aryl substitution at the Y ligand the HOMOs of the complexes with Y = NHpTol, OPh, PHPh, and SPh are 0.015, 0.007, 0.004, and 0.001 eV higher in energy than those with Y = NHMe, OMe, PHMe, and SMe, respectively (see Figure 2). The presence of an aromatic ring bonded to the nucleophilic atom increases even more the antibonding interactions above mentioned (see Figure 3). However, a reduction in the Gibbs energy barrier of **TS1** only happens for PHPh and SPh but not for NHpTol and OPh (see Table 1). As previously reported [58], apart from the HOMO energy of the reactant complex, other factors must be considered to rationalize this unexpected trend, so we now focus on the availability of the attacking electron lone pair of the nucleophilic atom at the Y ligand. Firstly, the N atom of the NHpTol ligand presents a sp^2^ hybridization, whereas a sp^3^ one happens for the NHMe ligand. As seen in Figure 3, sp^2^ hybridization reduces the availability of the electron lone pair of the nitrogen atom due to its conjugation with the aromatic ring, which provokes a rise of the Gibbs energy barrier for the nucleophilic addition. To confirm this point, the electron delocalization indexes (DI) of the bonds between the N atom at NHMe and NHpTol and the carbon atom (C_S_) of the alkyl or aryl substituent directly linked to it have been calculated, obtaining the values 1.0399 and 1.1322, respectively (see Table 2). The increase in DI agrees with the 0.089 Å shortening of the N-C_S_ bond distance when replacing NHMe by NHpTol. Similar but less accentuated trends were found when comparing the DI of the O-Me (0.9459) and S-Ph (1.1956) bonds at the corresponding reactant complexes. This bodes well with the fact that the O-Me bond distance is 0.071 Å longer than the O-Ph one. In addition, when analyzing the reactant Re complexes, we note that the net natural atomic charge (NAC) of the donor atoms N, O, P, and S reflects an electron depopulation of 0.009, 0.074, 0.048, and 0.294 e when going from NHMe, OMe, PHMe, and SMe to NHpTol, OPh, PHPh, and SPh, respectively (see Table 2).

In summary, for complexes wherein the aryl group is electronically conjugated with a lone pair at the donor atom, as for NHpTol and OPh, the negative effect of the unavailability of the electron lone pair prevails over the positive effect of the increase in the HOMO energy and, consequently, the instability of **TS1** increases. By contrast, when the aryl group is not strongly conjugated with the nucleophilic atom, as for PHPh (see Figure 3), the availability of the electron lone pair further favours the positive effect of the rise in the HOMO energy, thus provoking a notable stabilization of **TS1** (see Table 1).

The replacement of the N- and P-containing Y ligands by the O- and S-containing analogues disfavours their reactivity with HMAD (see Table 1). As displayed in Figure 2, the HOMO of the reactant complexes with NH_2_, NHMe, and NHpTol are 0.013, 0.022, and 0.020 eV higher in energy than those with OH, OMe, and OPh, thus yielding an increase in the Gibbs energy barrier of 8.5, 8.7, and 11.1 kcal/mol when going from NHR to OR (R = H, Me, *p*Tol/Ph), respectively. In consonance with this, the HOMO of the OH and OMe reactant complexes shows a simultaneous bonding overlap of a Re *d* orbital with three CO antibonding π orbitals, whereas only two CO antibonding π orbitals interact with a Re *d* orbital for the NH_2_ and NHMe cases (see Figure 3). For both NH*p*Tol and OPh the HOMO display a bonding overlap similar to that mentioned for the OH and OMe cases, but the greater antibonding nature of the π orbital of the NH*p*Tol that interacts with the Re *d* orbitals explains the greater stability of the HOMO of the OPh reactant complex. The same reasoning cannot be used to explain the instability rise of **TS1,** between 5.1 and 13.2 kcal/mol, when replacing ligands with P by the ones with S since the HOMO energy is practically the same when comparing analogous complexes (see Figure 2). Looking at the pictures of the HOMO of the reactant complexes with P ligands in Figure 3 and Figure S16, we note that such orbitals are mainly composed of a P-centered π orbital, whereas for the SR (R = H, Me, Ph) complexes the HOMO shows a notable antibonding *π* overlap between a Re *d* orbital and a π orbital of the SR ligand (see Figure 3). Therefore, the attacking electron lone pair of the S atom is less available to attack the activated alkyne than that of the P atom, thus involving a more energy-demanding step as mentioned above.

On the other hand, as previously mentioned, the regioselectivity of these processes is controlled by the Gibbs energy barriers for the formation of the possible products (**P*cco***/**P*ccoh***, **P*ccb***, **P*ins***) and their relative stability. As seen in Table 1, despite the remarkable relative stability of **P*ins***, this product is the most kinetically penalized except for the OR ligands. This fact is related to the strength in the Re-Y bond that can be quantified by the variation of the DI values of the Re-Y bond when going from reactants to **I1′**, the intermediate immediately prior to the formation of **P*ins*** in most cases. Table 2 and Table 3 show that all DIs reduce, but mainly those for N and O-containing ligands. The important weakening of the Re-O bond at **I1′** (or **I1**) explains why **TS2*ins*** competes with **TS2*ccb*** and **TS2*cco*** (when located), and **P*ins*** is the detected product for OMe, as experimentally found [48], and the suggested one for OPh. On the other hand, ligands with P and S display the smallest weakening of the R-Y bond (the DI reduction is 0.0046 and 0.0307 for the PMePh and PHMe cases, respectively). The similar size of the atomic orbitals of the P and Re atoms promotes a large overlap between them that makes their bond strong, so difficult to break in the insertion process. Concerning the Gibbs energy barriers for the formation of **P*cco***/**P*ccoh*** and **P*ccb***, **TS2*cco*** is, on the whole, less energy-demanding than **TS2*ccb*** as the attacked carbon of the carbonyl ligand in cis disposition to Y is less electronically populated than the attacked non-substituted carbon of the bipy ligand. Only for PPh_2_ **TS2*cco*** is 0.2 kcal/mol larger in energy than **TS2*ccb*** while for PH_2_ **TS2*cco*** and **TS2*ccb*** show the same relative instability. This explains why **P*cco*** (or **P*ccoh*** when present) is the preferred kinetic product. Nonetheless, when those TSs compete each other, **P*ccb*** is the predominat product as these products are always notably more stable than **P*cco***.

## 3. Computational Methods

Based on our previous theoretical studies on the reactions of [ReY(CO)_3_(bipy)] (Y = NH*p*Tol, OH, OMe, PPh_2_) complexes towards HMAD [57,58] and for comparison purposes, the present research was carried out using the levels of theory PCM-B3LYP/6-31+G(d, p)-LANL2DZ and CPCM-DLPNO-CCSD(T)/def2-TZVPP//PCM-B3LYP/6-31+G(d, p)-LANL2DZ for obtaining molecular geometries and energies, respectively (see Computational Chemistry Details in the Supporting Information for a more detailed description). For brevity, the levels of theory PCM-B3LYP/6-31+G(d, p)-LANL2DZ and CPCM-DLPNO-CCSD(T)/def2-TZVPP//PCM-B3LYP/6-31+G(d, p)-LANL2DZ have been denoted as B3LYP and DLPNO-CCSD(T), respectively. A relative permittivity of 7.58 was assumed in these calculations to simulate THF as the solvent experimentally used when investigating the reactivity of [ReY(CO)_3_(N-N)] (Y = NH*p*Tol, OH, OMe, PPh_2_; N-N = bipy and/or phen) towards activated alkynes [48,51,52,53,54]. All the B3LYP species located present singlet electronic state without spin contamination. Similarly, all the DLPNO-CCSD(T) species investigated showed T1 diagnostic values less than 0.02 [61], suggesting that a multi-reference treatment is not necessary.

Thermal free energy corrections in THF solution (G*_therm_*) were calculated at the B3LYP level using the standard procedure starting from the molecular partition functions developed for computing gas-phase thermodynamics properties within the ideal gas, rigid rotor, and harmonic oscillator approximations at a pressure of 1 atm and a temperature of 298.15 K [62,63]. For each species, Gibbs free energy in solution was determined by adding G*_therm_* to the highly accurate DLPNO-CCSD(T) energy. Unless stated otherwise, for each reactive process investigated, energies discussed in the following sections are all Gibbs free energies in THF solution referred to the corresponding separate reactants.

For interpretation purposes, the electronic structure of some relevant critical structures along the reaction mechanisms found was analyzed using the natural bond orbital (NBO) method [64] and the Bader’s Quantum Theory of Atoms in Molecules [65,66,67] to get, among other electron properties, net atomic charges (NAC) and electron delocalization indexes (DI) [68,69], respectively. The DI is a measure of the number of electrons shared between two atoms in a molecular system and therefore, of the covalency of the bond between them.

All B3LYP computations were carried out with the Gaussian 09 suite of programs [70], while DLPNO-CCSD(T) calculations employed the ORCA (version 4.0.1) program [71] and the frozen-core approximation. NAC and DI were computed with the NBO (version 3.1) and AIMAll (version 10.12.11) programs, respectively [72,73].

## 4. Conclusions

The systematic study of the reaction of [ReY(CO)_3_(bipy)] complexes (with Y being 15 different ligands of general formula NHR, OR, PR^1^R^2^, or SR; R^1^ = R^2^ = R = H, Me, Ph or *p*Tol) with HMDA gave rise to several potential energy surfaces, all of them with an initial large energy TS for the nucleophilic attack of Y to HMAD followed by diverse reaction channels towards three kinds of products, **P*cco***/**P*ccoh***, **P*ccb***, and **P*ins***. Ligands with N and S show an alternative approach of the reactants leading to **P*ccb***. The particular features of each ligand cause slight differences that determine the resulting product. Thus, ligands NH_2_, OH, PH_2_, and SH are poorer nucleophiles for the reaction with HMAD, whereas the presence of a moderate electron-donating substituent like a methyl group replacing one of the H atoms in these ligands favours the processes. The energy destabilization of the HOMO of the reactant complexes seems to be responsible for the diminution of the Gibbs energy barrier corresponding to the nucleophilic attack step. The presence of an aryl substituent replacing one of the H atoms in these ligands increases even more the energy destabilization of the HOMO of the reactant complexes, but this does not always imply an easier process as factors other than the HOMO energy may be predominant. Particularly, an aromatic ring bonded to N and O makes the reaction more difficult, whereas its linkage to P and S favours it. Since the π electrons in the aromatic ring conjugate with the lone pair at N atom in NH*p*Tol, this pair becomes less available for the nucleophilic attack on the HMAD molecule. The larger size of P prevents this conjugation, which explains the easier reactivity of PHPh compared to that of NH*p*Tol. It is also interesting to note that a more contracted donor atom at the Y ligand tends to disfavour the reactivity of these Re complexes towards HMAD. On the whole, ligands with N and P atoms show lower rate-determining Gibbs energy barriers than ligands with O and S atoms, respectively. This trend is ascribed to the stability of the HOMO of the reactant complexes and the availability of the attacking electron lone pair of the donor atom of the Y ligand.

Concerning the reaction products, the insertion route is greatly penalized for P and S ligands (although **P*ins*** is very stable) due to the strong bond formed between these large atoms and Re, which is weaker in the case of N and O atoms. P and S ligands tend to yield **P*ccb*** as the main product, which is not the general case for O and N ones. When the Y ligand has an O/N and an H atom the most likely product is **P*ccoh***, which is not always obtained when Y contains P/S. For OMe and OPh, the weakness of the Re-O bonds together with the instability of **P*cco*** and the absence of an O-bonded hydrogen atom explain why **P*ins*** is the preferred product only for these two situations.

## Figures and Tables

**Table 1:** Gibbs energy barriers, in kcal/mol, determined by the TS located along the reaction mechanisms found for the reaction between the complexes [ReY(CO)_3_(bipy)] (Y = NH_2_, NHMe, NH*p*Tol, OH, OMe, OPh, PH_2_, PHMe, PMe_2_, PHPh, PPh_2_, PMePh, SH, SMe, SPh) and HMAD at the DLPNO-CCSD(T) level. ^1,2^ Gibbs energy, in kcal/mol, of the plausible products referred to that of the respective separate reactants at the same level of theory and type of PES found for each Y ligand are also given.

Y	TS1	TS1′	TS1*b*	TS2*cco*	TS2*ccb*	TS2*ins*	P*cco* (P*ccoh*)	P*ccb*	P*ins*	PES
NH_2_	**18.5**	**8.5**	19.3	**4.3**	9.9	11.2	−8.0 (**−24.9**)	−15.1	−29.1	III
NHMe	17.2	4.3	**14.0**	0.7	**9.6**	9.2	−12.4 (−32.3)	**−19.3**	−31.2	III
NH*p*Tol	**22.4**	**17.0**	21.4		19.0	20.6	−0.2 (**−21.1** ^3^)	−5.1	−22.4	III
OH	**27.0**	**28.0**		**26.8**	31.2	28.3	15.2 (**−12.0** ^3^)	8.1	−13.7	I
OMe	**25.9**	**24.8**			29.5	**24.0**	**9.9**	4.9	**−16.4** ^3^	II
OPh	**33.5**				38.8	**36.5**	24.2	16.2	**−5.6**	IV
PH_2_	**27.6**	**14.6**		13.5	**13.5**	34.2	0.1 (0.3)	**−9.5**	−13.1	I
PHMe	**30.9**	**6.2**		5.1	**5.8**	21.1	−8.9 (−4.3)	**−16.3**	−18.3	I
PMe_2_	**17.4**	**4.2**		5.6	**4.3**	20.3	−17.3	**−25.5**	−24.1	I
PHPh	**17.6**	**9.7**		8.2	**9.4**	17.5	−7.4 (−4.2)	**−12.8**	−17.5	I
PPh_2_	**17.5**	**4.2**		5.2	**5.0**	20.6	−9.3	**−20.0** ^3^	−16.8	I
PMePh	**18.7**	**3.9**		3.7	**5.1**	18.7	−11.6	**−20.0**	−21.1	I
SH	**32.7**		28.0	**32.9**	35.8	39.1	21.1 **(−2.6)**	13.7	−2.4	III
SMe	23.1	26.1	**21.0**	24.7	**26.4**	32.1	11.0	**3.1**	−9.1	III
SPh	30.8		**25.6**	29.0	**31.0**	34.8	14.4	**8.9**	−5.7	III

^1^ All the Gibbs energy barriers are determined with respect to the sum of the Gibbs energies of the respective separate reactants or with respect to the Gibbs energy of the immediately preceding intermediate when this species is more stable than the corresponding separate reactants. ^2^ Bold data show the most preferred reaction route. ^3^ The product theoretically preferred is consistent with available experimental data (see Scheme 1).

**Table 2:** Bond distances between Re and the heteroatom of the Y ligand and between the heteroatom and the carbon of the alkyl or aryl substituent directly linked to it (d(Re-Y) and d(Y-C_S_) in Å, respectively), electron delocalization indexes at the bond critical points located between each of the two pairs of atoms mentioned above (DI(Re-Y) and DI(Y-C_S_), respectively), and net natural atomic charges of the nucleophilic atom of the Y ligand, the attacked carbon of the carbonyl ligand in *cis* disposition to the Y ligand, and the attacked non-substituted *ortho* carbon of the bipy ligand (NAC(Y), NAC(C*_CO_*), and NAC(C*_bipy_*) in *e*, respectively) of the reactant complexes [ReY(CO)_3_(bipy)] (Y = NH_2_, NHMe, NH*p*Tol, OH, OMe, OPh, PH_2_, PHMe, PMe_2_, PHPh, PPh_2_, PMePh, SH, SMe, SPh) at the B3LYP level.

Y	d(Re-Y)	d(Y-C_S_)	DI(Re-Y)	DI(Y-C_S_)	NAC(Y)	NAC(C*_CO_*)	NAC(C*_bipy_*)
NH_2_	2.174		0.6687		−1.164	0.734	0.063
NHMe	2.156	1.461	0.6689	1.0399	−0.799	0.733	0.066
NH*p*Tol	2.147	1.372	0.6218	1.1322	−0.790	0.734	0.068
OH	2.115		0.6145		−1.016	0.724	0.069
OMe	2.096	1.400	0.5998	0.9459	−0.752	0.724	0.067
OPh	2.134	1.329	0.5231	0.9925	−0.678	0.728	0.069
PH_2_	2.586		0.6736		−0.128	0.739	0.056
PHMe	2.582	1.882	0.6803	0.9166	0.180	0.737	0.059
PMe_2_	2.602	1.876	0.6661	0.8962	0.462	0.734	0.058
PHPh	2.591	1.844	0.6525	0.9337	0.232	0.735	0.062
PPh_2_	2.614	1.864	0.6352	0.8793	0.519	0.739	0.060
PMePh	2.615	1.847	0.6341	0.9256	0.510	0.735	0.058
SH	2.551		0.6541		−0.482	0.747	0.068
SMe	2.535	1.841	0.6758	1.1198	−0.188	0.745	0.066
SPh	2.545	1.782	0.6427	1.1956	−0.085	0.740	0.068

**Table 3:** Bond distances between Re and the heteroatom of the Y ligand (d(Re-Y) in Å), electron delocalization indexes at the bond critical points located between Re and Y (DI(Re-Y)), and net natural atomic charges of the nucleophilic atom of the Y ligand, the attacked carbon of the carbonyl ligand in *cis* disposition to the Y ligand, the attacked non-substituted *ortho* carbon of the bipy ligand, the non-substituted acetylenic carbon, and the substituted acetylenic carbon (NAC(Y), NAC(C*_CO_*), NAC(C*_bipy_*), NAC(C1), and NAC(C2) in *e*, respectively) at the intermediate **I1′** when Y = NH_2_, NHMe, NH*p*Tol, OH, OMe, OPh, PH_2_, PHMe, PMe_2_, PHPh, PPh_2_, PMePh, SH, SMe, SPh) at the B3LYP level.

Y	d(Re-Y)	DI(Re-Y)	NAC(Y)	NAC(C*_CO_*)	NAC(C*_bipy_*)	NAC(C1)	NAC(C2)
NH_2_	2.271	0.4893	−0.825	0.740	0.081	−0.155	−0.348
NHMe	2.292	0.4650	−0.646	0.727	0.080	−0.139	−0.336
NH*p*Tol ^1^	2.314	0.4594	−0.652	0.737	0.078	−0.154	−0.317
OH	2.253	0.3747	−0.706	0.730	0.082	−0.004	−0.392
OMe ^1^	2.256	0.3930	−0.568	0.725	0.089	−0.047	−0.388
OPh ^1^	2.316	0.3269	−0.544	0.726	0.087	0.003	−0.370
PH_2_	2.514	0.6458	0.686	0.762	0.079	−0.671	−0.298
PHMe	2.518	0.6496	0.956	0.763	0.081	−0.681	−0.295
PMe_2_	2.534	0.6412	1.199	0.761	0.080	−0.679	−0.290
PHPh	2.525	0.6389	0.970	0.762	0.081	−0.669	−0.290
PPh_2_	2.561	0.6135	1.225	0.756	0.078	−0.671	−0.293
PMePh	2.544	0.6295	1.215	0.756	0.080	−0.672	−0.288
SH ^1^	2.572	0.5528	0.199	0.756	0.083	−0.456	−0.333
SMe	2.565	0.5593	0.444	0.756	0.084	−0.479	−0.319
SPh ^1^	2.582	0.5372	0.470	0.750	0.085	−0.464	−0.311

^1^ For the sake of some degree of comparison, the non-appearance of **I1′** led us to analyze **I1** instead.

## Supplementary Materials

The following are available online, Explanation about the *d*^6^ electron configuration on these Re complexes, Computational Chemistry Details, Figures S1–S15: B3LYP optimized geometries of the species involved in the reaction between [ReY(CO)_3_(bipy)] (Y = NH_2_, NHMe, OPh, PH_2_, PHMe, PMe_2_, PHPh, PMePh, SH, SMe, SPh, NH*p*Tol, OH, OMe, and PPh_2_) towards HMAD, Tables S1–S45: B3LYP and DLPNO-CCSD(T) absolute and relative energies and entropies as well as Cartesian coordinates of the key species implied in the reaction of [ReY(CO)_3_(bipy)] (Y = NH_2_, NHMe, OPh, PH_2_, PHMe, PMe_2_, PHPh, PMePh, SH, SMe, SPh, NH*p*Tol, OH, OMe, and PPh_2_) towards HMAD, Figure S16: Pictures of the HOMO of the reactant complexes [ReY(CO)_3_(bipy)] (Y = PHMe, PPh_2_, PMePh), Table S46: Variation of the DLPNO-CCSD(T) relative Gibbs energies for all the analogous species when going from NH_2_ to NHMe and NH*p*Tol, Table S47: Variation of the DLPNO-CCSD(T) relative Gibbs energies for all the analogous species when going from OH to OMe and OPh, Table S48: Variation of the DLPNO-CCSD(T) relative Gibbs energies for all the analogous species when going from PH_2_ to PHMe, PMe_2_, PHPh, PPh_2_, and PMePh, Table S49: Variation of the DLPNO-CCSD(T) relative Gibbs energies for all the analogous species when going from SH to SMe and SPh, Table S50: Variation of the DLPNO-CCSD(T) relative Gibbs energies for all the analogous species when going from NH_2_, NHMe, and NH*p*Tol to PH_2_, PHMe, and PHPh, respectively, Table S51: Variation of the DLPNO-CCSD(T) relative Gibbs energies for all the analogous species when going from OH, OMe, and OPh to SH, SMe, and SPh, respectively.
