# Supplementary material for: Influence of the Nucleophilic Ligand on the Reactivity of Carbonyl Rhenium(I) Complexes towards Methyl Propiolate: A Computational Chemistry Perspective
Source: Molecules. 2020 Sep 10;25(18):4134. doi: 10.3390/molecules25184134 (PMC7571231; doi:10.3390/molecules25184134)
Supplement: Supplementary file 1 [file molecules-25-04134-s001.pdf]

Supporting Information

Influence of the Nucleophilic Ligand on the Reactivity of Carbonyl Rhenium(I) Complexes towards Methyl Propiolate: A Computational Chemistry Perspective

Daniel Álvarez, Elena López-Castro, Arturo Guerrero, Lucía Riera, Julio Pérez, Jesús Díaz, M. Isabel Menéndez and Ramón López

CONTENTS

1.	Table S1: B3LYP and DLPNO-CCSD(T) absolute energies and entropies of key species implied in the reaction of [Re(NH_2)($CO)_3$(bipy)] towards HMAD	S6
2.	Table S2: B3LYP and DLPNO-CCSD(T) relative energies and entropies of key species implied in the reaction of [Re(NH_2)($CO)_3$(bipy)] towards HMAD	S7
3.	Table S3: B3LYP cartesian coordinates of the optimized structures in the reaction between [Re(NH_2)($CO)_3$(bipy)] towards HMAD	S8-S11
4.	Figure S1: B3LYP optimized geometries of the species involved in the reaction between [Re(NH_2)($CO)_3$(bipy)] towards HMAD	S12-S14
5.	Table S4: B3LYP and DLPNO-CCSD(T) absolute energies and entropies of key species implied in the reaction of [Re(NHMe)($CO)_3$(bipy)] towards HMAD	S15
6.	Table S5: B3LYP and DLPNO-CCSD(T) relative energies and entropies of key species implied in the reaction of [Re(NHMe)($CO)_3$(bipy)] towards HMAD	S16
7.	Table S6: B3LYP cartesian coordinates of the optimized structures in the reaction between [Re(NHMe)($CO)_3$(bipy)] towards HMAD	S17-S21
8.	Figure S2: B3LYP optimized geometries of the species involved in the reaction between [Re(NHMe)($CO)_3$(bipy)] towards HMAD	S22-S24
9.	Table S7: B3LYP and DLPNO-CCSD(T) absolute energies and entropies of key species implied in the reaction of [Re(OPh)($CO)_3$(bipy)] towards HMAD	S25
10.	Table S8: B3LYP and DLPNO-CCSD(T) relative energies and entropies of key species implied in the reaction of [Re(OPh)($CO)_3$(bipy)] towards HMAD	S26

11.	Table S9: B3LYP cartesian coordinates of the optimized structures in the reaction between [Re(OPh)$(CO)_3$(bipy)] towards HMAD	S27-S29
12.	Figure S3: B3LYP optimized geometries of the species involved in the reaction between [Re(OPh)$(CO)_3$(bipy)] towards HMAD	S30-S31
13.	Table S10: B3LYP and DLPNO-CCSD(T) absolute energies and entropies of key species implied in the reaction of [Re$(PH_2)(CO)_3$(bipy)] towards HMAD	S32
14.	Table S11: B3LYP and DLPNO-CCSD(T) relative energies and entropies of key species implied in the reaction of [Re$(PH_2)(CO)_3$(bipy)] towards HMAD	S33
15.	Table S12: B3LYP cartesian coordinates of the optimized structures in the reaction between [Re$(PH_2)(CO)_3$(bipy)] towards HMAD	S34-S37
16.	Figure S4: B3LYP optimized geometries of the species involved in the reaction between [Re$(PH_2)(CO)_3$(bipy)] towards HMAD	S38-S39
17.	Table S13: B3LYP and DLPNO-CCSD(T) absolute energies and entropies of key species implied in the reaction of [Re(PHMe)$(CO)_3$(bipy)] towards HMAD	S40
18.	Table S14: B3LYP and DLPNO-CCSD(T) relative energies and entropies of key species implied in the reaction of [Re(PHMe)$(CO)_3$(bipy)] towards HMAD	S41
19.	Table S15: B3LYP cartesian coordinates of the optimized structures in the reaction between [Re(PHMe)$(CO)_3$(bipy)] towards HMAD	S42-S45
20.	Figure S5: B3LYP optimized geometries of the species involved in the reaction between [Re(PHMe)$(CO)_3$(bipy)] towards HMAD	S46-S47
21.	Table S16: B3LYP and DLPNO-CCSD(T) absolute energies and entropies of key species implied in the reaction of [Re$(PMe_2)(CO)_3$(bipy)] towards HMAD	S48
22.	Table S17: B3LYP and DLPNO-CCSD(T) relative energies and entropies of key species implied in the reaction of [Re$(PMe_2)(CO)_3$(bipy)] towards HMAD	S49
23.	Table S18: B3LYP cartesian coordinates of the optimized structures in the reaction between [Re$(PMe_2)(CO)_3$(bipy)] towards HMAD	S50-S53
24.	Figure S6: B3LYP optimized geometries of the species involved in the reaction between [Re$(PMe_2)(CO)_3$(bipy)] towards HMAD	S54-S55
25.	Table S19: B3LYP and DLPNO-CCSD(T) absolute energies and entropies of key species implied in the reaction of [Re(PHPh)$(CO)_3$(bipy)] towards HMAD	S56
26.	Table S20: B3LYP and DLPNO-CCSD(T) relative energies and entropies of key species implied in the reaction of	S57

	[Re(PHPh)(CO)$_3$(bipy)] towards HMAD	
27.	Table S21: B3LYP cartesian coordinates of the optimized structures in the reaction between [Re(PHPh)(CO)$_3$(bipy)] towards HMAD	S58-S61
28.	Figure S7: B3LYP optimized geometries of the species involved in the reaction between [Re(PHPh)(CO)$_3$(bipy)] towards HMAD	S62-S63
29.	Table S22: B3LYP and DLPNO-CCSD(T) absolute energies and entropies of key species implied in the reaction of [Re(PMePh)(CO)$_3$(bipy)] towards HMAD	S64
30.	Table S23: B3LYP and DLPNO-CCSD(T) relative energies and entropies of key species implied in the reaction of [Re(PMePh)(CO)$_3$(bipy)] towards HMAD	S65
31.	Table S24: B3LYP cartesian coordinates of the optimized structures in the reaction between [Re(PMePh)(CO)$_3$(bipy)] towards HMAD	S66-S69
32.	Figure S8: B3LYP optimized geometries of the species involved in the reaction between [Re(PMePh)(CO)$_3$(bipy)] towards HMAD	S70-S71
33.	Table S25: B3LYP and DLPNO-CCSD(T) absolute energies and entropies of key species implied in the reaction of [Re(SH)(CO)$_3$(bipy)] towards HMAD	S72
34.	Table S26: B3LYP and DLPNO-CCSD(T) relative energies and entropies of key species implied in the reaction of [Re(SH)(CO)$_3$(bipy)] towards HMAD	S73
35.	Table S27: B3LYP cartesian coordinates of the optimized structures in the reaction between [Re(SH)(CO)$_3$(bipy)] towards HMAD	S74-S77
36.	Figure S9: B3LYP optimized geometries of the species involved in the reaction between [Re(SH)(CO)$_3$(bipy)] towards HMAD	S78-S80
37.	Table S28: B3LYP and DLPNO-CCSD(T) absolute energies and entropies of key species implied in the reaction of [Re(SMe)(CO)$_3$(bipy)] towards HMAD	S81
38.	Table S29: B3LYP and DLPNO-CCSD(T) relative energies and entropies of key species implied in the reaction of [Re(SMe)(CO)$_3$(bipy)] towards HMAD	S82
39.	Table S30: B3LYP cartesian coordinates of the optimized structures in the reaction between [Re(SMe)(CO)$_3$(bipy)] towards HMAD	S83-S86
40.	Figure S10: B3LYP optimized geometries of the species involved in the reaction between [Re(SMe)(CO)$_3$(bipy)] towards HMAD	S87-S89
41.	Table S31: B3LYP and DLPNO-CCSD(T) absolute energies and entropies of key species implied in the reaction of [Re(SPh)(CO)$_3$(bipy)] towards HMAD	S90
42.	Table S32: B3LYP and DLPNO-CCSD(T) relative energies and	

	entropies of key species implied in the reaction of [Re(SPh)$(CO)_3$(bipy)] towards HMAD	S91
43.	Table S33: B3LYP cartesian coordinates of the optimized structures in the reaction between [Re(SPh)$(CO)_3$(bipy)] towards HMAD	S92-S95
44.	Figure S11: B3LYP optimized geometries of the species involved in the reaction between [Re(SPh)$(CO)_3$(bipy)] towards HMAD	S96-S97
45.	Table S34: B3LYP and DLPNO-CCSD(T) absolute energies and entropies of key species implied in the reaction of [Re(NH*p*Tol)$(CO)_3$(bipy)] towards HMAD	S98
46.	Table S35: B3LYP and DLPNO-CCSD(T) relative energies and entropies of key species implied in the reaction of [Re(NH*p*Tol)$(CO)_3$(bipy)] towards HMAD	S99
47.	Table S36: B3LYP cartesian coordinates of the optimized structures in the reaction between [Re(NH*p*Tol)$(CO)_3$(bipy)] towards HMAD	S100-S104
48.	Figure S12: B3LYP optimized geometries of the species involved in the reaction between [Re(NH*p*Tol)$(CO)_3$(bipy)] towards HMAD	S105-S107
49.	Table S37: B3LYP and DLPNO-CCSD(T) absolute energies and entropies of key species implied in the reaction of [Re(OH)$(CO)_3$(bipy)] towards HMAD	S108
50.	Table S38: B3LYP and DLPNO-CCSD(T) relative energies and entropies of key species implied in the reaction of [Re(OH)$(CO)_3$(bipy)] towards HMAD	S109
51.	Table S39: B3LYP cartesian coordinates of the optimized structures in the reaction between [Re(OH)$(CO)_3$(bipy)] towards HMAD	S110-S112
52.	Figure S13: B3LYP optimized geometries of the species involved in the reaction between [Re(OH)$(CO)_3$(bipy)] towards HMAD	S113-S114
53.	Table S40: B3LYP and DLPNO-CCSD(T) absolute energies and entropies of key species implied in the reaction of [Re(OMe)$(CO)_3$(bipy)] towards HMAD	S115
54.	Table S41: B3LYP and DLPNO-CCSD(T) relative energies and entropies of key species implied in the reaction of [Re(OMe)$(CO)_3$(bipy)] towards HMAD	S116
55.	Table S42: B3LYP cartesian coordinates of the optimized structures in the reaction between [Re(OMe)$(CO)_3$(bipy)] towards HMAD	S117-S119
56.	Figure S14: B3LYP optimized geometries of the species involved in the reaction between [Re(OMe)$(CO)_3$(bipy)] towards HMAD	S120-S121
57.	Table S43: B3LYP and DLPNO-CCSD(T) absolute energies and entropies of key species implied in the reaction of [Re$(PPh_2)(CO)_3$(bipy)] towards HMAD	S122

58.	Table S44: B3LYP and DLPNO-CCSD(T) relative energies and entropies of key species implied in the reaction of [Re(PPh_2)(CO)$_3$(bipy)] towards HMAD	S123
59.	Table S45: B3LYP cartesian coordinates of the optimized structures in the reaction between [Re(PPh_2)(CO)$_3$(bipy)] towards HMAD	S124-S128
60.	Figure S15: B3LYP optimized geometries of the species involved in the reaction between [Re(PPh_2)(CO)$_3$(bipy)] towards HMAD	S129-S130
61.	Figure S16: Pictures of the HOMO of the reactant complexes [ReY(CO)$_3$(bipy)] (Y = PHMe, PPh_2, PMePh)	S131
62.	Table S46: Variation of the DLPNO-CCSD(T) relative Gibbs energies for all the analogous species when going from NH_2 to NHMe and NH*p*Tol.	S132
63.	Table S47: Variation of the DLPNO-CCSD(T) relative Gibbs energies for all the analogous species when going from OH to OMe and OPh.	S132
64.	Table S48: Variation of the DLPNO-CCSD(T) relative Gibbs energies for all the analogous species when going from PH_2 to PHMe, PMe_2, PHPh, PPh_2, and PMePh.	S132
65.	Table S49: Variation of the DLPNO-CCSD(T) relative Gibbs energies for all the analogous species when going from SH to SMe and SPh.	S132
66.	Table S50: Variation of the DLPNO-CCSD(T) relative Gibbs energies for all the analogous species when going from NH_2, NHMe, and NH*p*Tol to PH_2, PHMe, and PHPh, respectively.	S133
67.	Table S51: Variation of the DLPNO-CCSD(T) relative Gibbs energies for all the analogous species when going from OH, OMe, and OPh to SH, SMe, and SPh, respectively.	S133

Table S1. PCM-B3LYP/6-31+G(d,p) (LANL2DZ for Re) energies without and with including thermal corrections (E and G, respectively), enthalpies (H), and entropies (S), and CPCM-DLPNO-CCSD(T)/def2-TZVPP//PCM-B3LYP/6-31+G(d,p) (LANL2DZ for Re) energies without and with including thermal corrections (E' and G', respectively) in THF solution of the critical structures involved in the reaction between the complex [Re(NH_2)$(CO)_3$(bipy)] (bipy = 2,2'-bipyridine) and methyl propiolate (HMAD, HC≡CCO_2Me). All the values are given in hartree, except entropies that are in cal/K mol.[a]

Species	E	H	S	G	E'	G'[b]
[Re(NH_2)$(CO)_3$(bipy)]	-970.598953	-970.366824	141.207	-970.433916	-968.097099	-967.932062
HMAD	-305.215726	-305.136831	77.228	-305.173524	-304.738441	-304.696239
Reactants	-1275.814679	-1275.503655	218.435	-1275.607440	-1272.835540	-1272.628301
**TS1**	-1275.805718	-1275.493451	174.248	-1275.576242	-1272.828258	-1272.598782
**I1**	-1275.834854	-1275.518229	174.291	-1275.601040	-1272.861501	-1272.627687
**TS1'**	-1275.827933	-1275.512990	169.824	-1275.593679	-1272.849052	-1272.614798
**I1'**	-1275.834372	-1275.518741	178.771	-1275.603681	-1272.856790	-1272.626099
**TS2*cco***	-1275.834136	-1275.519686	168.976	-1275.599972	-1272.855639	-1272.621475
**P*cco***	-1275.860475	-1275.543068	170.172	-1275.623923	-1272.877564	-1272.641012
**P*ccoh***	-1275.897011	-1275.579757	164.736	-1275.658029	-1272.906941	-1272.667959
**TS1*b***	-1275.803975	-1275.491727	175.796	-1275.575254	-1272.826268	-1272.597547
**I1*b***	-1275.836211	-1275.519980	174.076	-1275.602689	-1272.863846	-1272.630324
**I1'*b***	-1275.839651	-1275.523131	173.994	-1275.605801	-1272.864538	-1272.630688
**TS2*ccb***	-1275.827296	-1275.512362	168.231	-1275.592294	-1272.849846	-1272.614844
**P*ccb***	-1275.858903	-1275.541537	164.636	-1275.619761	-1272.891503	-1272.652361
**TS2*ins***	-1275.819721	-1275.505945	172.800	-1275.588048	-1272.842167	-1272.610494
**P*ins***	-1275.882167	-1275.565698	175.205	-1275.648944	-1272.907968	-1272.674745

[a] Thermal magnitudes were computed in THF solution at 298.15 K and 1 atm. [b] For each species, G' was calculated as G' = G – E + E', in which G is the PCM-B3LYP/6-31+G(d,p) (LANL2DZ for Re) energy with including thermal corrections and E and E' are the PCM-B3LYP/6-31+G(d,p) (LANL2DZ for Re) and CPCM-DLPNO-CCSD(T)/def2-TZVPP//PCM-B3LYP/6-31+G(d,p) (LANL2DZ for Re) energies without including thermal corrections, respectively.

Table S2. PCM-B3LYP/6-31+G(d,p) (LANL2DZ for Re) relative energies without and with including thermal corrections (ΔE and ΔG, respectively), enthalpies (ΔH), and entropic contributions (TΔS), and CPCM-DLPNO-CCSD(T)/def2-TZVPP//PCM-B3LYP/6-31+G(d,p) (LANL2DZ for Re) relative energies without and with including thermal corrections (E' and G', respectively) in THF solution of the critical structures involved in the reaction between the complex [Re(NH_2)$(CO)_3$(bipy)] (bipy = 2,2'-bipyridine) and methyl propiolate (HMAD, HC≡CO_2Me). All the values are given in kcal/mol.[a]

Species	ΔE	ΔH	TΔS	ΔG	ΔE'	ΔG'
[Re(NH_2)$(CO)_3$(bipy)] + HMAD	0.0	0.0	0.0	0.0	0.0	0.0
**TS1**	5.6	6.4	-13.2	19.6	4.6	18.5
**I1**	-12.7	-9.1	-13.2	4.0	-16.3	0.4
**TS1'**	-8.3	-5.9	-14.5	8.6	-8.5	8.5
**I1'**	-12.4	-9.5	-11.8	2.4	-13.3	1.4
**TS2*cco***	-12.2	-10.1	-14.7	4.7	-12.6	4.3
**P*cco***	-28.7	-24.7	-14.4	-10.3	-26.4	-8.0
**P*ccoh***	-51.7	-47.8	-16.0	-31.7	-44.8	-24.9
**TS1*b***	6.7	7.5	-12.7	20.2	5.8	19.3
**I1*b***	-13.5	-10.2	-13.2	3.0	-17.8	-1.3
**I1'*b***	-15.7	-12.2	-13.3	1.0	-18.2	-1.5
**TS2*ccb***	-7.9	-5.5	-15.0	9.5	-9.0	8.4
**P*ccb***	-27.8	-23.8	-16.0	-7.7	-35.1	-15.1
**TS2*ins***	-3.2	-1.4	-13.6	12.2	-4.2	11.2
**P*ins***	-42.3	-38.9	-12.9	-26.0	-45.4	-29.1

[a] Thermal magnitudes were evaluated in THF solution at 298.15 K and 1 atm.

Table S3. PCM-B3LYP/6-31+G(d,p) (LANL2DZ for Re) optimized cartesian coordinates, in Å, for the critical structures involved in the reaction of the complex [Re(NH*p*Tol)$(CO)_3$(bipy)] (bipy = 2,2'-bipyridine) towards methyl propiolate (HMAD, HC≡CCO_2Me).

[Re(NH_2)$(CO)_3$(bipy)]

N	-0.501718	-0.070628	1.904390
Re	-0.834615	0.031711	-0.241936
C	-2.196885	-1.314012	-0.201850
O	-3.010283	-2.151730	-0.166178
N	0.924927	1.376164	-0.173356
C	2.135566	0.779158	-0.041912
C	3.311313	1.536912	0.012652
C	3.237953	2.924929	-0.046416
C	1.986407	3.529556	-0.162352
C	0.858962	2.718320	-0.224884
C	2.128964	-0.697639	0.002244
N	0.923893	-1.296772	-0.186092
C	0.854635	-2.641968	-0.179387
C	1.965948	-3.452066	0.022698
C	3.205075	-2.846053	0.228091
C	3.284952	-1.457579	0.213799
C	-2.185676	1.379259	-0.051162
O	-2.982723	2.219657	0.098747
C	-0.916928	0.128133	-2.180065
O	-0.986734	0.167675	-3.347091
H	-0.131165	3.145572	-0.320155
H	1.876713	4.605339	-0.207022
H	4.141732	3.520257	-0.004495
H	4.094064	-3.440979	0.397713
H	1.851989	-4.528383	0.022086
H	-0.127157	-3.070230	-0.335193
H	4.274424	1.051303	0.094708
H	4.236938	-0.971174	0.378275
H	-1.328577	0.197748	2.435801
H	-0.333941	-1.039763	2.177835

TS1

C	1.584895	3.447561	-0.232531
C	1.475592	2.053820	-0.194270
N	0.304642	1.432559	-0.491575
C	-0.774907	2.177262	-0.790448
C	-0.730610	3.567080	-0.846090
C	0.472801	4.213288	-0.572495
C	2.603326	1.166858	0.161845
N	2.339508	-0.165597	0.138995
C	3.320854	-1.033769	0.450482
C	4.603772	-0.624488	0.794807
C	4.884202	0.741326	0.822662
C	3.874149	1.643326	0.503897
Re	0.290995	-0.783916	-0.441916
C	0.811496	-0.928576	-2.304292
O	1.145968	-1.025536	-3.419565
N	-0.181056	-0.451369	1.648012
C	-1.980920	0.830829	2.337039
C	-2.992052	0.887977	1.637122
C	-3.945579	0.843629	0.587579
O	-3.960577	1.604634	-0.381870
O	-4.870174	-0.130287	0.764445
C	-5.830376	-0.272943	-0.300864
C	-1.546222	-1.088435	-0.923968
O	-2.653284	-1.269600	-1.242779
C	0.505590	-2.669460	-0.187238
O	0.664376	-3.811083	0.000798
H	-1.381483	1.032589	3.195867
H	-6.485378	-1.084418	0.009246
H	-6.396561	0.650972	-0.429833
H	-5.317033	-0.523732	-1.230439
H	3.058572	-2.082977	0.416279
H	5.357059	-1.364253	1.033755
H	5.871695	1.100273	1.086196
H	4.075035	2.706152	0.517309
H	2.524854	3.931305	-0.004553
H	0.546286	5.293701	-0.613310
H	-1.627931	4.117354	-1.097921
H	-1.700906	1.650961	-0.981799
H	0.602650	-0.056493	2.169148
H	-0.448807	-1.291031	2.157022

I1

C	1.409357	3.503450	-0.300578
C	1.314876	2.114238	-0.198765
N	0.279529	1.437484	-0.764137
C	-0.699542	2.128193	-1.376396
C	-0.662525	3.513517	-1.508350
C	0.416313	4.211653	-0.974063
C	2.294942	1.291992	0.539464
N	2.060913	-0.048490	0.549116
C	2.903795	-0.856588	1.222868
C	4.011303	-0.377686	1.912332
C	4.262746	0.993699	1.903378
C	3.395811	1.833282	1.211089
Re	0.327029	-0.756073	-0.613928
C	1.367143	-0.914326	-2.226699
O	2.003963	-1.017130	-3.195097
N	-0.823922	-0.531685	1.338953
C	-1.193749	0.826623	1.832040
C	-2.344066	1.434332	1.588979
C	-3.422971	0.949030	0.798219
O	-3.609180	1.150933	-0.415319
O	-4.388828	0.285767	1.539112
C	-5.599456	-0.042200	0.843969
C	-1.290734	-1.119522	-1.605881
O	-2.253764	-1.328753	-2.219762
C	0.526634	-2.640418	-0.275783
O	0.668195	-3.774962	-0.052465
H	-0.358304	1.263823	2.374971
H	-6.227641	-0.553346	1.572865
H	-6.099152	0.861239	0.485947
H	-5.402622	-0.697084	-0.007717
H	2.678011	-1.915147	1.197235
H	4.655832	-1.070806	2.437290
H	5.118207	1.405944	2.424764
H	3.574365	2.900008	1.196004
H	2.241933	4.029950	0.144939
H	0.479802	5.289496	-1.061014
H	-1.474909	4.020709	-2.011796
H	-1.542571	1.556072	-1.738708
H	-1.681915	-1.070369	1.234945
H	-0.282280	-1.009565	2.059646

TS1'

C	-1.441475	3.423911	0.346453
C	-1.360740	2.040620	0.167669
N	-0.221287	1.356736	0.462908
C	0.855937	2.036498	0.903014
C	0.832047	3.413763	1.107275
C	-0.337021	4.118273	0.833343
C	-2.482272	1.234197	-0.353897
N	-2.245665	-0.099252	-0.481690
C	-3.225931	-0.897516	-0.952004
C	-4.474844	-0.414312	-1.320922
C	-4.726970	0.951674	-1.193739
C	-3.722530	1.779355	-0.703088
Re	-0.288115	-0.836593	0.211871
C	-0.944114	-1.100671	2.000070
O	-1.345603	-1.265571	3.080598
N	0.404350	-0.451388	-1.919640
C	1.489095	0.565332	-2.179872
C	2.613490	0.597990	-1.532577
C	3.735491	0.663777	-0.760361
O	3.886434	1.317562	0.307668
O	4.802222	-0.100476	-1.252501
C	5.984643	-0.083232	-0.448713
C	1.503000	-1.275732	0.815525
O	2.540533	-1.574565	1.240792
C	-0.591906	-2.690613	-0.186103
O	-0.820367	-3.803300	-0.450345
H	1.178476	1.237405	-2.984505
H	6.690888	-0.744807	-0.951449
H	6.405272	0.923743	-0.379025
H	5.784999	-0.448928	0.561192
H	-2.990308	-1.951510	-1.019151
H	-5.225887	-1.099066	-1.693175
H	-5.689767	1.366552	-1.466095
H	-3.905547	2.839128	-0.588138
H	-2.351680	3.955451	0.104987
H	-0.389454	5.190160	0.982945
H	1.725174	3.910836	1.462745
H	1.772012	1.478494	1.063364

H	-0.399916	-0.215117	-2.503436
H	0.736270	-1.346001	-2.277883

I1′

C	-1.921789	-0.900841	-0.049312
C	-1.957554	-1.716985	-1.184808
N	-3.115914	-1.911827	-1.871556
C	-4.237776	-1.294168	-1.448473
C	-4.265157	-0.469772	-0.330203
C	-3.084407	-0.273191	0.385586
C	-0.768663	-2.420667	-1.710037
N	-0.974502	-3.195466	-2.807331
C	0.066430	-3.853640	-3.352113
C	1.348749	-3.795015	-2.816153
C	1.568217	-3.009694	-1.685961
C	0.500037	-2.310644	-1.131790
Re	-3.051248	-3.340215	-3.558409
C	-2.753646	-4.603025	-5.011248
O	-2.669237	-5.484262	-5.764395
C	-4.896954	-3.219915	-4.059467
O	-6.026497	-3.114834	-4.335503
N	-2.511935	-1.489994	-4.759639
H	-3.375631	-1.067218	-5.101216
C	-3.466942	-4.816193	-2.407523
O	-3.724564	-5.707000	-1.699911
C	-1.570297	-1.594842	-5.908498
C	-1.052591	-2.749748	-6.283621
C	-0.136552	-2.943364	-7.355289
O	-0.761996	-3.226794	-8.553867
C	0.097198	-3.610382	-9.635661
O	1.103149	-2.982092	-7.277630
H	-1.387058	-0.612063	-6.354900
H	0.653280	-1.686916	-0.261632
H	-0.997647	-0.764353	0.495632
H	-0.147810	-4.415745	-4.251005
H	2.150330	-4.348578	-3.287544
H	2.554967	-2.936224	-1.244123
H	-3.066276	0.355566	1.267723
H	-5.194823	-0.002643	-0.031753
H	-5.133539	-1.481669	-2.026209
H	-2.140637	-0.795614	-4.108061
H	-0.564741	-3.844633	-10.468785
H	0.771895	-2.795995	-9.910142
H	0.692818	-4.486478	-9.368829

TS2*cco*

C	-2.528159	0.035278	1.745588
C	-1.241300	-0.025542	2.290040
N	-1.050633	-0.189080	3.625929
C	-2.123535	-0.268023	4.436672
C	-3.427784	-0.220164	3.954809
C	-3.633438	-0.066338	2.585068
C	-0.016451	0.089397	1.471529
C	-0.039054	0.277806	0.085146
C	1.156999	0.385100	-0.616498
C	2.359289	0.302588	0.086054
C	2.316846	0.112266	1.461371
N	1.160733	0.006051	2.148416
Re	1.051750	-0.365525	4.322827
C	1.054999	-2.267526	4.099035
O	1.054658	-3.426714	3.960864
C	0.745699	-0.602026	6.240755
O	0.685503	-0.945311	7.351575
C	2.938315	-0.413346	4.635496
O	4.099013	-0.436324	4.772507
N	0.966628	1.902180	4.451553
C	0.052333	2.492874	5.464960
C	-0.527132	1.733921	6.372508
C	-1.453675	2.065934	7.392226
O	-0.853560	2.326003	8.608014
C	-1.738386	2.458077	9.728372
O	-2.693180	2.013001	7.305608
H	-0.050492	3.573871	5.336654
H	-2.471222	3.249818	9.560513
H	-1.102506	2.706443	10.577336
H	-2.267599	1.520578	9.919024
H	-4.635466	-0.020224	2.175110
H	1.148514	0.528838	-1.690289
H	3.316186	0.379960	-0.413835
H	3.226199	0.030798	2.042590
H	-0.981539	0.337334	-0.441800
H	-2.668127	0.165787	0.681140
H	-4.255458	-0.289126	4.648837
H	-1.913397	-0.339819	5.495345

H	0.714598	2.273393	3.533961
H	1.907115	2.261250	4.618188

P*cco*

N	-1.055739	1.480954	0.191223
C	-2.331319	1.464501	-0.281761
C	-2.995752	2.654514	-0.601753
C	-2.354493	3.873658	-0.408429
C	-1.056169	3.880990	0.103999
C	-0.442242	2.666042	0.385487
C	-2.953927	0.131182	-0.392541
N	-2.189734	-0.910858	0.038042
C	-2.710516	-2.155962	-0.016714
C	-3.990166	-2.420048	-0.487279
C	-4.780636	-1.354448	-0.924268
C	-4.254200	-0.068585	-0.873686
Re	-0.114011	-0.466467	0.676142
C	0.485810	-2.232565	1.017919
O	0.806885	-3.348804	1.222379
N	0.448253	-0.570091	-1.533525
C	1.848895	-0.221995	-1.712194
C	2.544444	0.166009	-0.640496
C	3.976455	0.564536	-0.790171
O	4.380877	1.439158	-1.541642
H	2.256529	-0.290110	-2.715543
C	-0.372673	-0.207326	2.530282
O	-0.492039	-0.021090	3.687541
C	1.823249	0.308423	0.719676
O	2.448482	0.962570	1.572716
O	4.778907	-0.151192	0.008440
C	6.179265	0.200426	-0.017772
H	-4.007110	2.630428	-0.984205
H	-4.848353	0.772655	-1.205120
H	0.563645	2.614489	0.782968
H	-0.523421	4.806204	0.282749
H	-2.862379	4.800782	-0.648358
H	-5.784744	-1.520797	-1.295376
H	-4.355114	-3.439317	-0.501921
H	-2.065798	-2.949808	0.338144
H	6.658009	-0.465991	0.695218
H	6.586357	0.053996	-1.019241
H	6.305687	1.242199	0.279696
H	0.279769	-1.501392	-1.913667
H	-0.126156	0.056035	-2.101965

P*ccoh*

C	-2.464128	3.123423	0.106314
C	-2.015433	1.797900	0.068859
N	-0.786574	1.462298	0.541972
C	-0.001151	2.421851	1.068439
C	-0.394679	3.752945	1.146977
C	-1.649121	4.110196	0.650735
C	-2.822310	0.681840	-0.458220
N	-2.249756	-0.550374	-0.367452
C	-2.942580	-1.616111	-0.823668
C	-4.214192	-1.512226	-1.372070
C	-4.808240	-0.251884	-1.462443
C	-4.102322	0.852857	-0.999927
Re	-0.186459	-0.664535	0.408508
C	0.050016	-2.542186	0.218606
O	0.135214	-3.709623	0.103086
C	1.820765	0.143904	-1.655629
C	2.590170	0.030389	-0.463694
C	4.010669	0.260703	-0.415574
O	4.698975	0.148804	0.619015
C	1.834311	-0.376233	0.689041
O	2.502035	-0.505501	1.842578
C	-0.537274	-0.938798	2.256627
O	-0.699706	-1.088101	3.413522
O	4.578527	0.611261	-1.592186
C	6.001183	0.839123	-1.571662
H	2.286402	0.442429	-2.592429
H	3.463941	-0.307898	1.674349
H	-2.448617	-2.575299	-0.732587
H	0.963799	2.092500	1.431937
H	-3.441613	3.379928	-0.278821
H	-4.723194	-2.404461	-1.714474
H	-4.542291	1.839622	-1.060700
H	-5.799601	-0.131301	-1.882576
H	0.271308	4.485628	1.584867
H	-1.988583	5.138032	0.688561
H	6.268726	1.081315	-2.597952
H	6.525097	-0.058257	-1.239644
H	6.244697	1.667845	-0.905139

N	0.544034	−0.133279	−1.590505
H	0.039085	−0.051093	−2.466631

TS1*b*

C	2.913294	1.180079	1.293369
N	1.819221	1.083199	0.515053
C	1.091796	2.199269	0.254075
C	1.471272	3.443775	0.769871
C	2.605510	3.538563	1.570001
C	3.340635	2.384303	1.840119
Re	1.152055	−0.804800	−0.439053
N	−0.254997	−0.956717	1.200820
C	−0.105706	2.003215	−0.589571
N	−0.298799	0.743672	−1.061145
C	−1.400100	0.478869	−1.787095
C	−2.349899	1.447995	−2.090514
C	−2.145705	2.748580	−1.635331
C	−1.011916	3.028232	−0.877937
C	2.355521	−0.509784	−1.931772
O	3.095408	−0.332328	−2.818147
C	0.381835	−2.306594	−1.354821
O	−0.100364	−3.200353	−1.930930
C	2.420392	−1.967495	0.406159
O	3.195986	−2.639551	0.962688
C	−2.317960	−1.877123	0.686725
C	−3.335105	−1.190130	0.762915
C	−4.367976	−0.223521	0.846995
O	−4.757908	0.481484	−0.083535
O	−4.917381	−0.167197	2.089568
H	−1.740093	−2.753151	0.499784
H	−1.516110	−0.542515	−2.122094
H	−3.230810	1.175759	−2.655965
H	−2.861317	3.531698	−1.855025
H	2.909968	4.497884	1.972351
H	4.227930	2.407698	2.459751
H	3.452157	0.258687	1.472639
H	0.888770	4.327765	0.548851
H	−0.844046	4.028870	−0.502627
H	0.039455	−1.524469	1.992424
H	−0.536925	−0.057439	1.587367
C	−6.002072	0.764273	2.257232
H	−6.283575	0.693571	3.305692
H	−5.678655	1.777178	2.013852
H	−6.842768	0.490154	1.617324

I1*b*

C	−0.882001	−0.334949	0.025592
N	0.127398	−0.040118	0.871300
C	0.885357	1.064245	0.638250
C	0.633690	1.888507	−0.462777
C	−0.412207	1.584101	−1.325792
C	−1.187488	0.450477	−1.077393
Re	0.680104	−1.271797	2.607997
N	2.170476	−2.196220	1.164709
C	3.607805	−1.827589	1.330231
C	4.342601	−1.177869	0.444823
C	3.981387	−0.654781	−0.823231
O	3.518838	0.476425	−1.065634
C	1.946927	1.337163	1.626323
N	2.044833	0.452122	2.651180
C	2.971473	0.656628	3.607348
C	3.830116	1.747932	3.595823
C	3.738119	2.660049	2.544675
C	2.790007	2.448741	1.550757
C	−0.573985	−0.403933	3.786886
O	−1.332541	0.113114	4.501028
C	1.445601	−2.244685	4.086702
O	1.933222	−2.812669	4.977617
C	−0.574855	−2.701831	2.304951
O	−1.353344	−3.541188	2.091939
O	4.319600	−1.497228	−1.873338
H	3.947405	−2.137380	2.317064
H	3.011152	−0.076717	4.401403
H	4.553353	1.869377	4.392142
H	4.395854	3.519424	2.496835
H	−0.615567	2.218563	−2.180145
H	−2.011865	0.172918	−1.720765
H	−1.453972	−1.223920	0.256514
H	1.253382	2.754251	−0.647277
H	2.709418	3.137254	0.722121
H	1.896620	−1.981587	0.205560
H	2.078183	−3.207560	1.257597
C	4.213995	−0.933261	−3.185353
H	4.528258	−1.719607	−3.871223

H	3.187811	−0.629313	−3.405518
H	4.868424	−0.064200	−3.288368

I1′*b*

C	0.857309	−0.854482	−0.477160
N	0.184766	0.280557	−0.754105
C	0.674041	1.467100	−0.303228
C	1.864130	1.521845	0.428785
C	2.550984	0.346198	0.715601
C	2.037764	−0.866187	0.255297
Re	−1.626714	0.368183	−2.009931
N	−2.752900	0.316910	−0.047371
C	−3.900542	1.242560	0.060857
C	−3.870319	2.279745	0.882864
C	−4.884787	3.279986	0.940698
O	−4.946876	4.313442	0.254577
C	−0.131401	2.661989	−0.628315
N	−1.232081	2.444176	−1.396621
C	−2.050350	3.474820	−1.675616
C	−1.797962	4.771427	−1.240071
C	−0.657926	5.008490	−0.478145
C	0.178832	3.940394	−0.161612
C	−0.565245	0.494845	−3.615004
O	0.090758	0.563695	−4.573098
C	−3.231146	0.692383	−3.031157
O	−4.183095	0.912710	−3.661719
C	−1.848048	−1.525164	−2.280738
O	−1.961723	−2.676260	−2.416367
O	−5.798040	3.075902	1.959109
H	−4.682058	0.961249	−0.649526
H	−2.928527	3.244086	−2.261899
H	−2.494203	5.561588	−1.485508
H	−0.427229	6.003819	−0.117756
H	3.472665	0.378787	1.283778
H	2.535866	−1.806609	0.452431
H	0.429211	−1.770252	−0.863755
H	2.249682	2.472330	0.771425
H	1.055261	4.101467	0.451064
H	−2.122140	0.557186	0.719515
H	−3.062148	−0.638610	0.127147
C	−6.767921	4.114058	2.147734
H	−7.372791	3.802096	2.998262
H	−6.282272	5.069167	2.360496
H	−7.396121	4.230836	1.261395

TS2*ccb*

C	0.717967	−0.853081	−0.345386
N	0.085496	0.313804	−0.592083
C	0.711673	1.485685	−0.298247
C	2.001847	1.486967	0.248301
C	2.645663	0.282763	0.502994
C	1.992673	−0.914306	0.198600
Re	−1.866678	0.473142	−1.612117
N	−2.725520	0.902517	0.473109
C	−3.828790	1.868789	0.479649
C	−3.636166	3.159186	0.223193
C	−4.683098	4.140739	0.152571
O	−5.030790	4.737761	−0.873264
C	−0.053973	2.714208	−0.586306
N	−1.234445	2.528372	−1.253301
C	−2.110058	3.579198	−1.327587
C	−1.631625	4.911629	−1.118241
C	−0.397869	5.101526	−0.553596
C	0.387555	3.974002	−0.213402
C	−1.037151	0.260212	−3.329769
O	−0.530353	0.134465	−4.370854
C	−3.546700	0.854302	−2.479479
O	−4.547530	1.095899	−3.020010
C	−2.325633	−1.398636	−1.576283
O	−2.586960	−2.533985	−1.518290
O	−5.210369	4.474053	1.374301
H	−4.790614	1.381337	0.644087
H	−2.951113	3.444780	−1.993478
H	−2.284983	5.738348	−1.369000
H	−0.023444	6.099284	−0.353843
H	3.644043	0.276805	0.924142
H	2.456217	−1.876423	0.375905
H	0.176278	−1.753021	−0.606087
H	2.492367	2.425403	0.468938
H	1.331465	4.100169	0.298397
H	−3.024118	0.043161	0.925849
H	−1.954343	1.268825	1.028812
C	−6.163988	5.550026	1.379052
H	−6.434680	5.696415	2.423587

H	-5.721857	6.460596	0.970227
H	-7.047308	5.288360	0.793241

P*ccb*

C	2.417431	3.141243	0.477526
C	1.617899	2.029705	0.165552
N	2.093492	0.767791	0.358490
C	3.334696	0.592540	0.865212
C	4.161229	1.652458	1.199720
C	3.689588	2.955450	0.997972
C	0.253391	2.119388	-0.389203
N	-0.252862	0.942064	-0.872164
C	-1.702041	0.871839	-1.087884
C	-2.265238	2.205388	-1.550178
C	-1.668898	3.355121	-1.163806
C	-0.424834	3.322769	-0.443355
C	-2.454822	0.297852	0.133203
C	-3.928617	0.174698	-0.033462
O	-4.482206	0.178080	-1.123168
Re	0.795050	-0.864958	-0.346862
C	-0.491765	-2.126551	-1.026871
O	-1.263454	-2.885441	-1.458926
C	-1.903308	-0.254172	1.227344
N	-0.487061	-0.332876	1.500524
C	1.753763	-2.301750	0.515882
O	2.342370	-3.140030	1.078393
C	1.844106	-1.097639	-1.928947
O	2.486771	-1.230617	-2.892561
O	-4.604844	0.054424	1.127142
C	-6.030356	-0.139802	1.004605
H	-2.536501	-0.728045	1.968120
H	-6.482843	0.714343	0.499406
H	-6.397528	-0.224018	2.024489
H	-6.239774	-1.049548	0.440558
H	-1.884975	0.132970	-1.878992
H	-3.165859	2.192595	-2.151264
H	-2.096569	4.315092	-1.438704
H	4.311957	3.809917	1.236222
H	5.147488	1.460365	1.601650
H	3.655593	-0.433318	0.992838
H	2.040419	4.138254	0.294982
H	0.013957	4.232301	-0.058247
H	-0.332878	-0.943518	2.298649
H	-0.127014	0.583924	1.767917

TS2*ins*

C	-1.219734	3.496650	0.337610
C	-1.177065	2.106478	0.183448
N	-0.094561	1.387787	0.576842
C	0.953551	2.034827	1.118830
C	0.968971	3.410998	1.312467
C	-0.137951	4.156984	0.910360
C	-2.284268	1.329130	-0.404706
N	-2.084966	-0.010533	-0.491211
C	-3.054576	-0.778434	-1.029258
C	-4.254048	-0.259357	-1.497414
C	-4.469718	1.117256	-1.406144
C	-3.475122	1.914978	-0.854096
Re	-0.193222	-0.844226	0.365766
C	-0.588842	-2.696234	0.094832
O	-0.889069	-3.813046	-0.071927
C	1.548588	-1.308899	1.043722
O	2.586643	-1.579090	1.495056
C	-0.940168	-0.961987	2.090909
O	-1.380425	-1.042057	3.175926
C	1.926079	-0.020478	-1.273827
C	3.189652	0.639404	-1.103549
O	3.358827	1.849007	-0.901788
C	1.328592	-0.391462	-2.394349
O	4.241112	-0.237883	-1.038166
C	5.517917	0.328971	-0.702657
H	1.802216	1.423822	1.391942
H	1.838269	3.878781	1.755989
H	-0.159757	5.232625	1.038153
H	-2.086360	4.056924	0.013979
H	-2.852825	-1.840915	-1.074192
H	-4.997206	-0.923671	-1.919606
H	-5.395011	1.560395	-1.754610
H	-3.622783	2.983197	-0.772496
H	1.704518	-0.246384	-3.410459
H	5.833014	1.051853	-1.458144
H	5.475925	0.825576	0.269505
H	6.211562	-0.509879	-0.670214
N	0.038419	-1.050907	-2.263232
H	-0.678653	-0.579941	-2.814867
H	0.078618	-2.008282	-2.614060

P*ins*

C	0.069754	-0.365429	-0.972053
N	-0.969507	0.316055	-0.454963
C	-0.725816	1.427832	0.285622
C	0.581665	1.863094	0.528626
C	1.652162	1.146461	0.003989
C	1.391896	0.012518	-0.763837
Re	-3.092329	-0.284910	-0.704678
C	-2.965456	-1.465467	0.846508
O	-2.897640	-2.196079	1.753511
C	-1.911328	2.104145	0.848060
N	-3.109452	1.516436	0.595288
C	-4.218671	2.045773	1.146052
C	-4.198137	3.181810	1.945386
C	-2.975616	3.809316	2.183451
C	-1.823004	3.259580	1.633724
C	-2.840657	-1.771359	-1.902056
O	-2.691455	-2.690130	-2.601699
C	-4.978366	-0.584962	-0.778206
O	-6.138451	-0.754872	-0.776253
C	-3.060356	1.131259	-2.447069
C	-1.966469	1.012311	-3.415578
O	-1.225866	0.034235	-3.547821
C	-3.977687	2.095791	-2.752359
N	-5.125448	2.402068	-2.055002
O	-1.775061	2.107122	-4.232773
H	-3.834420	2.738941	-3.617816
H	-0.180808	-1.223575	-1.578024
H	2.190685	-0.574506	-1.197646
H	2.668987	1.470197	0.189815
H	0.760046	2.744372	1.129404
H	-0.862026	3.720869	1.814452
H	-2.918003	4.703540	2.792366
H	-5.123166	3.559857	2.361577
H	-5.146805	1.528485	0.942919
H	-5.494731	1.700505	-1.430341
H	-5.838667	2.908157	-2.558917
C	-0.777758	1.964734	-5.252550
H	-0.796218	2.898173	-5.814127
H	0.208968	1.806566	-4.811796
H	-1.010002	1.122796	-5.908541

Figure S1. PCM-B3LYP/6-31+G(d,p) (LANL2DZ for Re) optimized geometries in THF solution of the critical structures involved in the reaction between the complex [Re(NH_2)(CO)$_3$(bipy)] (bipy = 2,2'-bipyridine) and methyl propiolate (HMAD, HC≡CCO_2Me). Relevant distances are given in angstroms.

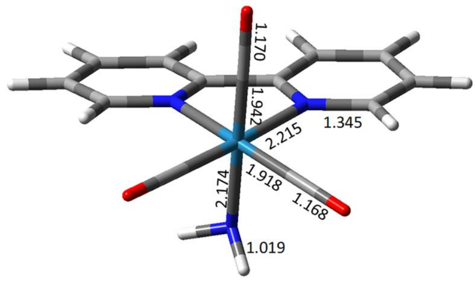

[Re(NH_2)(CO)$_3$(bipy)]

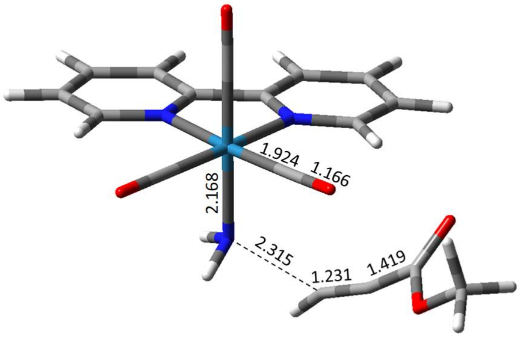

TS1

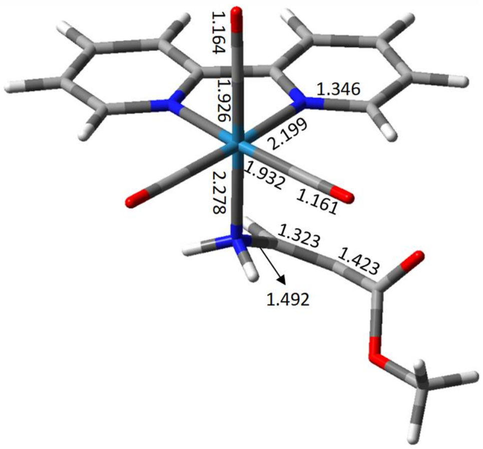

I1

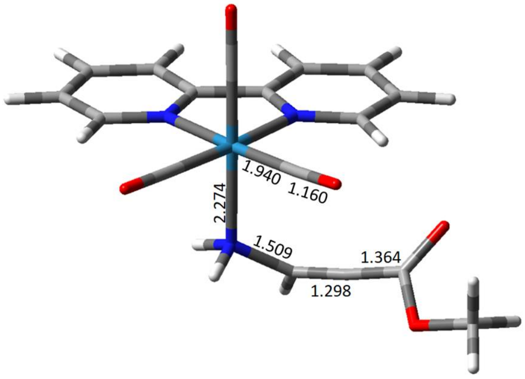

TS1'

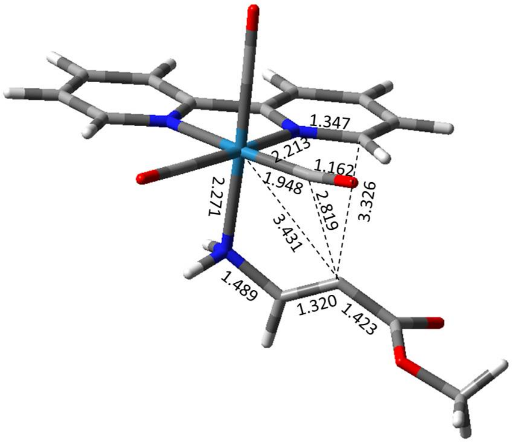

I1'

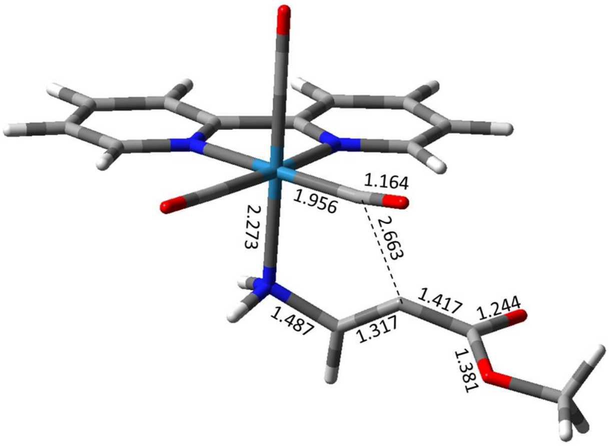

TS2*cco*

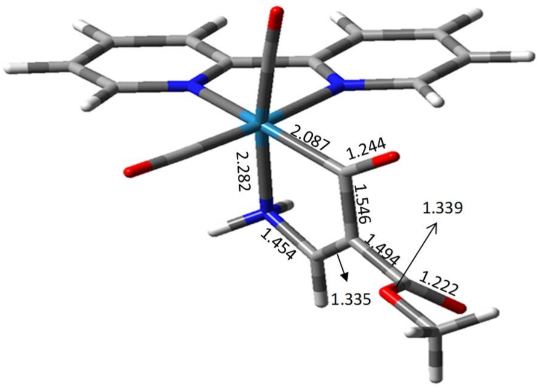

P*cco*

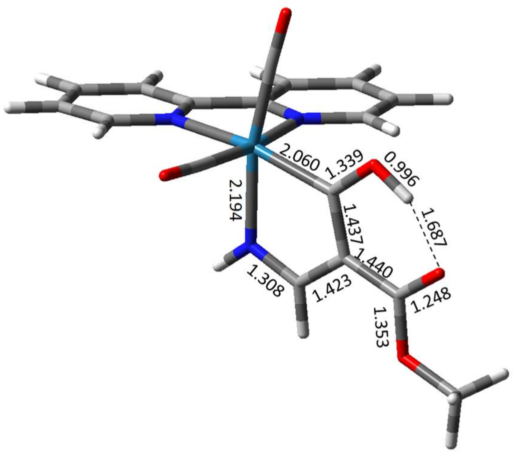

P*ccoh*

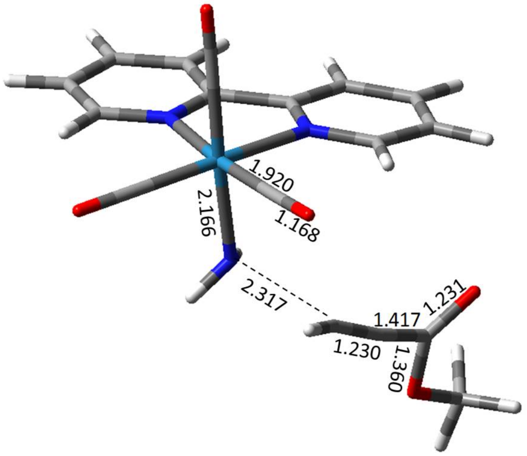

TS1*b*

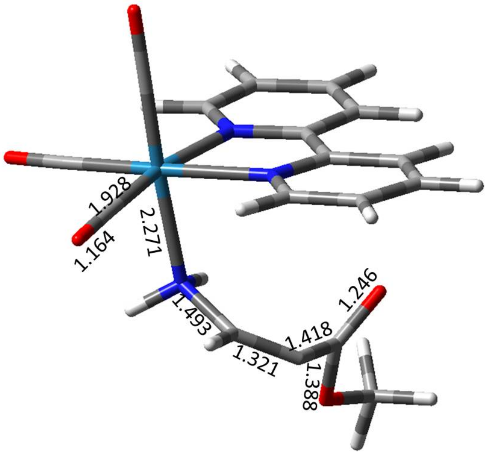

I1*b*

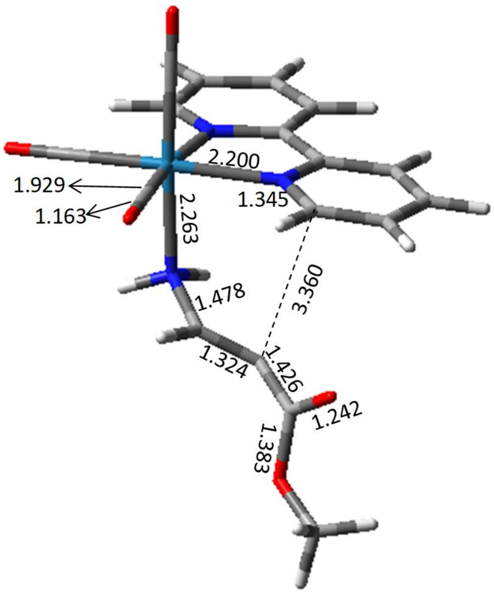

I1'*b*

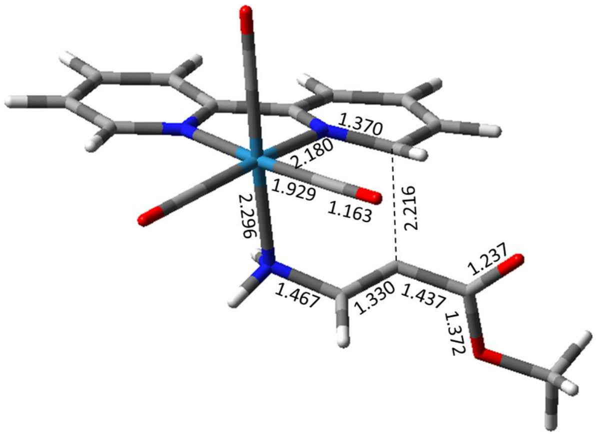

TS2*ccb*

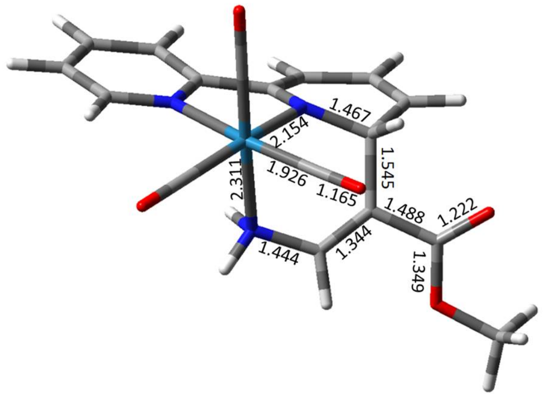

P*ccb*

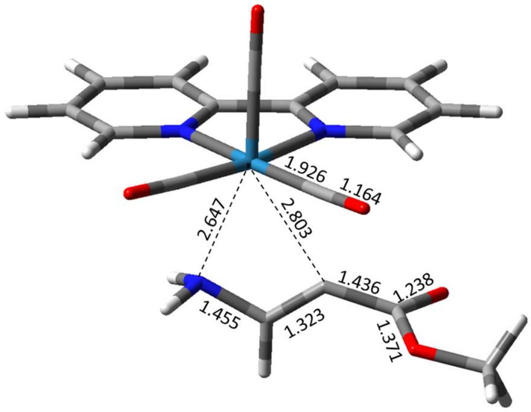

TS2*ins*

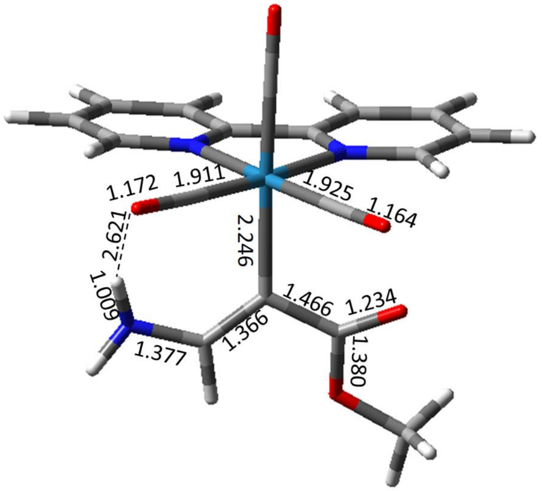

P*ins*

Table S4. PCM-B3LYP/6-31+G(d,p) (LANL2DZ for Re) energies without and with including thermal corrections (E and G, respectively), enthalpies (H), and entropies (S), and CPCM-DLPNO-CCSD(T)/def2-TZVPP//PCM-B3LYP/6-31+G(d,p) (LANL2DZ for Re) energies without and with including thermal corrections (E' and G', respectively) in THF solution of the critical structures involved in the reaction between the complex [Re(NHMe)$(CO)_3$(bipy)] (bipy = 2,2'-bipyridine) and methyl propiolate (HMAD, HC≡CCO_2Me). All the values are given in hartree, except entropies that are in cal/K mol.[a]

Species	E	H	S	G	E'	G'[b]
[Re(NHMe)$(CO)_3$(bipy)]	-1009.897456	-1009.635683	147.755	-1009.705886	-1007.320506	-1007.128936
HMAD	-305.215726	-305.136831	77.228	-305.173524	-304.738441	-304.696239
Reactants	-1315.113182	-1314.772514	224.983	-1314.879410	-1312.058947	-1311.825175
**TS1**	-1315.105239	-1314.764475	174.451	-1314.847362	-1312.055711	-1311.797834
**I1**	-1315.134130	-1314.787568	172.745	-1314.869645	-1312.087766	-1311.823281
**TS1'**	-1315.128900	-1314.784378	176.745	-1314.868355	-1312.078796	-1311.818251
**I1'**	-1315.137138	-1314.791598	180.006	-1314.877125	-1312.086519	-1311.826506
**TS2*cco***	-1315.137121	-1314.792605	176.165	-1314.876307	-1312.086161	-1311.825347
**P*cco***	-1315.164688	-1314.817787	175.257	-1314.901057	-1312.108626	-1311.844995
**P*ccoh***	-1315.203839	-1314.857340	172.321	-1314.939215	-1312.141273	-1311.876649
**TS1*b***	-1315.105575	-1314.765009	180.148	-1314.850603	-1312.057791	-1311.802819
**I1*b***	-1315.138126	-1314.792290	179.419	-1314.877538	-1312.093890	-1311.833302
**I1'*b***	-1315.142792	-1314.796601	179.505	-1314.881889	-1312.094831	-1311.833928
**TS2*ccb***	-1315.130256	-1314.785681	173.529	-1314.868130	-1312.080806	-1311.818680
**P*ccb***	-1315.162456	-1314.815842	170.892	-1314.897039	-1312.121316	-1311.855899
**TS2*ins***	-1315.123952	-1314.780196	176.004	-1314.863821	-1312.071882	-1311.811751
**P*ins***	-1315.186386	-1314.841509	175.445	-1314.924868	-1312.136337	-1311.874819

[a] Thermal magnitudes were computed in THF solution at 298.15 K and 1 atm. [b] For each species, G' was calculated as G' = G – E + E', in which G is the PCM-B3LYP/6-31+G(d,p) (LANL2DZ for Re) energy with including thermal corrections and E and E' are the PCM-B3LYP/6-31+G(d,p) (LANL2DZ for Re) and CPCM-DLPNO-CCSD(T)/def2-TZVPP//PCM-B3LYP/6-31+G(d,p) (LANL2DZ for Re) energies without including thermal corrections, respectively.

Table S5. PCM-B3LYP/6-31+G(d,p) (LANL2DZ for Re) relative energies without and with including thermal corrections (ΔE and ΔG, respectively), enthalpies (ΔH), and entropic contributions (TΔS), and CPCM-DLPNO-CCSD(T)/def2-TZVPP//PCM-B3LYP/6-31+G(d,p) (LANL2DZ for Re) relative energies without and with including thermal corrections (E' and G', respectively) in THF solution of the critical structures involved in the reaction between the complex [Re(NHMe)$(CO)_3$(bipy)] (bipy = 2,2'-bipyridine) and methyl propiolate (HMAD, HC≡CCO_2Me). All the values are given in kcal/mol.[a]

Species	ΔE	ΔH	TΔS	ΔG	ΔE'	ΔG'
[Re(NHMe)$(CO)_3$(bipy)] + HMAD	0.0	0.0	0.0	0.0	0.0	0.0
**TS1**	5.0	5.0	-15.1	20.1	2.0	17.2
**I1**	-13.1	-9.4	-15.6	6.1	-18.1	1.2
**TS1'**	-9.9	-7.4	-14.4	6.9	-12.5	4.3
**I1'**	-15.0	-12.0	-13.4	1.4	-17.3	-0.8
**TS2*cco***	-15.0	-12.6	-14.6	1.9	-17.1	-0.1
**P*cco***	-32.3	-28.4	-14.8	-13.6	-31.2	-12.4
**P*ccoh***	-56.9	-53.2	-15.7	-37.5	-51.7	-32.3
**TS1*b***	4.8	4.7	-13.4	18.1	0.7	14.0
**I1*b***	-15.7	-12.4	-13.6	1.2	-21.9	-5.1
**I1'*b***	-18.6	-15.1	-13.6	-1.6	-22.5	-5.5
**TS2*ccb***	-10.7	-8.3	-15.3	7.1	-13.7	4.1
**P*ccb***	-30.9	-27.2	-16.1	-11.1	-39.1	-19.3
**TS2*ins***	-6.8	-4.8	-14.6	9.8	-8.1	8.4
**P*ins***	-45.9	-43.3	-14.8	-28.5	-48.6	-31.2

[a] Thermal magnitudes were evaluated in THF solution at 298.15 K and 1 atm.

Table S6. PCM-B3LYP/6-31+G(d,p) (LANL2DZ for Re) optimized cartesian coordinates, in Å, for the critical structures involved in the reaction of the complex [Re(NHMe)$(CO)_3$(bipy)] (bipy = 2,2'-bipyridine) towards methyl propiolate (HMAD, HC≡CCO_2Me).

[Re(NHMe)$(CO)_3$(bipy)]

N	0.719724	0.584872	0.071076
Re	-0.235517	-1.342039	-0.082466
C	0.900680	-1.916366	-1.513857
O	1.572475	-2.253720	-2.408640
N	-1.655744	-0.454127	1.375009
C	-2.575893	0.411116	0.880577
C	-3.471037	1.075951	1.726174
C	-3.406906	0.859252	3.098889
C	-2.453530	-0.027664	3.598186
C	-1.604572	-0.667461	2.701295
C	-2.575817	0.584244	-0.586520
N	-1.683288	-0.174003	-1.275794
C	-1.653496	-0.089612	-2.620167
C	-2.492727	0.753416	-3.338738
C	-3.400510	1.548444	-2.638660
C	-3.441458	1.460052	-1.251380
C	0.961573	-2.146112	1.182024
O	1.670259	-2.626539	1.977068
C	-1.268652	-2.987037	-0.206092
O	-1.872311	-3.983051	-0.316152
H	-0.852596	-1.366821	3.043840
H	-2.364130	-0.227520	4.658073
H	-4.090114	1.372697	3.764381
H	-4.065743	2.224440	-3.161643
H	-2.426735	0.782490	-4.418864
H	-0.928083	-0.717582	-3.120563
H	-4.211591	1.751978	1.319620
H	-4.136759	2.070259	-0.691209
H	0.368100	1.194397	-0.665490
C	2.177314	0.685067	0.065370
H	2.668069	0.266543	-0.833124
H	2.510125	1.729365	0.154989
H	2.590014	0.143561	0.924390

TS1

C	1.566833	3.465850	-0.213364
C	1.466281	2.072284	-0.161238
N	0.281081	1.444912	-0.388623
C	-0.818781	2.186504	-0.620480
C	-0.784866	3.576770	-0.669619
C	0.431331	4.227948	-0.474585
C	2.611444	1.195535	0.166571
N	2.352212	-0.137351	0.186376
C	3.341561	-0.994151	0.500186
C	4.631329	-0.571511	0.804025
C	4.908228	0.794518	0.783032
C	3.888575	1.685071	0.461449
Re	0.284515	-0.768324	-0.311112
C	0.763071	-0.954045	-2.181873
O	1.091343	-1.065484	-3.297658
N	-0.066084	-0.366823	1.788904
C	-2.204869	0.413072	2.581995
C	-3.149312	0.575093	1.816298
C	-4.041962	0.707205	0.716850
O	-4.008906	1.618931	-0.110163
O	-4.949594	-0.292923	0.671615
C	-5.804879	-0.307174	-0.488259
C	-1.552753	-1.103956	-0.772370
O	-2.648585	-1.316387	-1.113687
C	0.508442	-2.652650	-0.043393
O	0.671522	-3.795889	0.130987
H	-1.656453	0.394931	3.497573
H	0.326663	0.547796	2.011461
H	-6.463948	-1.160869	-0.349034
H	-6.379974	0.617557	-0.550754
H	-5.199589	-0.429060	-1.387598
H	3.083538	-2.044890	0.494624
H	5.392774	-1.300848	1.048912
H	5.900774	1.163531	1.010450
H	4.086227	2.748062	0.442581
H	2.517360	3.953414	-0.044801
H	0.496755	5.308341	-0.515399
H	-1.700600	4.123745	-0.854295
H	-1.753776	1.656413	-0.748736
C	0.386086	-1.303449	2.810141
H	-0.072817	-2.283269	2.645164
H	1.480010	-1.456473	2.831754
H	0.095510	-0.974763	3.822211

I1

C	-4.049570	0.869670	-1.520344
C	-3.001103	0.229648	-0.856191
N	-2.056568	0.940687	-0.182443
C	-2.117465	2.285143	-0.190603
C	-3.144204	2.978436	-0.827294
C	-4.131134	2.260439	-1.495058
C	-2.830451	-1.237254	-0.850647
N	-1.762957	-1.707032	-0.150546
C	-1.536587	-3.036318	-0.112664
C	-2.350869	-3.954959	-0.762454
C	-3.448862	-3.483265	-1.481480
C	-3.689696	-2.113752	-1.522461
Re	-0.541576	-0.218315	0.930015
C	-1.687834	-0.260119	2.475943
O	-2.374064	-0.283438	3.415355
N	0.749330	-0.184966	-0.972098
C	0.409808	0.835128	-2.025240
C	0.686154	2.126348	-2.006066
C	1.255894	3.006073	-1.060155
O	0.639596	3.740735	-0.262025
O	2.636059	3.120665	-1.160439
C	3.239901	4.082223	-0.285469
C	0.493558	1.205915	1.735431
O	1.129796	2.028153	2.250366
C	0.633334	-1.482027	1.771387
O	1.311450	-2.287161	2.273023
H	-0.109925	0.343285	-2.847332
H	0.535807	-1.087145	-1.398188
H	4.312884	4.004559	-0.459942
H	2.894936	5.093596	-0.513682
H	3.005562	3.856900	0.757956
H	-0.678684	-3.359784	0.461888
H	-2.123354	-5.011184	-0.699588
H	-4.107147	-4.168555	-2.001896
H	-4.534685	-1.731477	-2.077537
H	-4.796537	0.295015	-2.050625
H	-4.944761	2.767522	-2.000630
H	-3.148178	4.060296	-0.801353
H	-1.299351	2.814372	0.282330
C	2.218085	-0.197974	-0.702155
H	2.509132	0.753942	-0.262839
H	2.459876	-1.006816	-0.012610
H	2.767615	-0.331119	-1.637509

TS1'

C	-1.444601	3.497356	0.149104
C	-1.370602	2.105368	0.054755
N	-0.256281	1.430813	0.447074
C	0.806947	2.124153	0.899849
C	0.790295	3.511515	1.020169
C	-0.355856	4.208690	0.647038
C	-2.467701	1.279600	-0.489019
N	-2.255689	-0.064293	-0.492605
C	-3.210597	-0.875999	-0.991750
C	-4.408284	-0.398430	-1.508818
C	-4.635743	0.977682	-1.505916
C	-3.656276	1.820592	-0.991117
Re	-0.367895	-0.780462	0.400689
C	-1.103924	-0.787866	2.177824
O	-1.546814	-0.797023	3.254417
N	0.460716	-0.672814	-1.731953
C	1.429697	0.462154	-1.949956
C	2.583050	0.546479	-1.359397
C	3.737660	0.663142	-0.645222
O	3.890138	1.243461	0.464533
O	4.838573	0.053895	-1.263496
C	6.086034	0.193867	-0.582386
C	1.378022	-1.205420	1.131276
O	2.392552	-1.480457	1.622057
C	-0.737760	-2.657034	0.250197
O	-1.011636	-3.787009	0.156508
H	1.010463	1.173730	-2.667588
H	-0.343851	-0.472488	-2.327565
H	6.825409	-0.289100	-1.222377
H	6.348639	1.245719	-0.441152

H	6.066751	-0.296006	0.395210
H	-2.999169	-1.936865	-0.966234
H	-5.139029	-1.095552	-1.898084
H	-5.559068	1.388965	-1.896015
H	-3.815120	2.890078	-0.983866
H	-2.338437	4.020789	-0.161851
H	-0.402829	5.287498	0.731469
H	1.670400	4.021212	1.390455
H	1.705030	1.564003	1.137220
C	1.048920	-1.950008	-2.228955
H	1.918731	-2.185332	-1.616816
H	0.317579	-2.755223	-2.151176
H	1.367235	-1.840678	-3.269109

I1′

N	-1.926829	-0.898108	-0.060497
C	-1.958415	-1.706403	-1.152966
C	-3.147499	-1.924813	-1.856492
C	-4.318876	-1.305841	-1.430376
C	-4.274644	-0.470083	-0.315871
C	-3.059352	-0.285563	0.334699
C	-0.670206	-2.317964	-1.540609
N	0.391104	-2.023222	-0.742003
C	1.597751	-2.547306	-1.041069
C	1.809415	-3.371179	-2.139483
C	0.726569	-3.676104	-2.964659
C	-0.522051	-3.144726	-2.659822
Re	0.007958	-0.737017	1.017105
C	1.784942	-0.742127	1.725090
O	2.877549	-0.781601	2.137820
N	0.447240	0.940758	-0.481507
C	-0.295409	2.209584	-0.266614
C	-1.133075	2.371123	0.740138
C	-1.939695	3.516904	0.997250
O	-3.097432	3.711549	0.591483
H	-0.059144	2.935737	-1.052454
C	-0.412803	-2.219421	2.151310
O	-0.668608	-3.121705	2.847046
C	-0.535768	0.472880	2.457182
O	-0.809071	0.985455	3.466412
O	-1.360414	4.392840	1.889059
C	-2.183525	5.480094	2.332978
H	-3.160187	-2.571472	-2.723273
H	-1.372248	-3.370001	-3.288922
H	-2.964123	0.383970	1.179077
H	-5.156970	0.042395	0.044798
H	-5.247115	-1.470416	-1.963892
H	0.850470	-4.316583	-3.829620
H	2.799941	-3.762496	-2.333086
H	2.407168	-2.292100	-0.369705
H	0.104557	0.568962	-1.370104
H	-1.549657	6.079643	2.984418
H	-2.532614	6.078996	1.489461
H	-3.049444	5.111035	2.888543
C	1.893941	1.214576	-0.698797
H	2.328569	1.571048	0.234881
H	2.397275	0.295225	-1.002989
H	2.034491	1.975557	-1.472880

TS2*cco*

N	1.154567	0.047451	2.172168
C	-0.006008	0.117725	1.465479
C	0.003933	0.339634	0.083795
C	1.215660	0.488888	-0.582285
C	2.401503	0.412931	0.148904
C	2.325966	0.188877	1.517846
C	-1.248625	-0.042164	2.249133
C	-2.522245	-0.021362	1.670968
C	-3.644271	-0.172508	2.480266
C	-3.470402	-0.327261	3.854448
C	-2.178695	-0.330101	4.369538
N	-1.088010	-0.206180	3.589025
Re	0.996526	-0.332504	4.344948
N	0.862715	1.954514	4.430465
C	0.027904	2.495844	5.531784
C	-0.549630	1.708155	6.419005
C	-1.419510	2.108540	7.475059
O	-2.656777	2.194263	7.418262
C	1.042032	-2.233065	4.139019
O	1.073861	-3.394251	4.014449
C	0.613480	-0.573697	6.255155
O	0.507949	-0.977004	7.343819
C	2.868532	-0.367603	4.731450
O	4.021115	-0.398655	4.924737
O	-0.764839	2.283756	8.672892
C	-1.595762	2.518373	9.818420
H	-0.038359	3.587860	5.474493
H	-2.219525	3.402972	9.675882
H	-0.910545	2.669459	10.650941
H	-2.240327	1.657801	10.013594
H	-4.635806	-0.163424	2.043935
H	1.232684	0.662225	-1.651583
H	3.369140	0.520304	-0.324323
H	3.220177	0.116699	2.122834
H	-0.924893	0.398120	-0.466936
H	-2.637281	0.106506	0.603310
H	-4.313016	-0.435617	4.524842
H	-1.992896	-0.409489	5.432285
H	0.377876	2.205473	3.565911
C	2.171243	2.660820	4.370164
H	2.027596	3.745814	4.351794
H	2.756222	2.393065	5.249779
H	2.709267	2.353945	3.471549

P*cco*

N	-0.147378	0.818871	-1.081196
C	-1.491051	1.268034	-1.401439
C	-2.523609	0.802095	-0.693777
C	-3.910621	1.226542	-1.052042
O	-4.404051	1.120152	-2.165232
Re	-0.234583	-0.525747	0.785210
C	-2.267496	-0.265190	0.390978
O	-3.277522	-0.891528	0.758958
N	1.942407	-0.867824	0.553184
C	2.342661	-1.795642	-0.360069
C	3.699330	-2.009553	-0.635372
C	4.664786	-1.276734	0.045431
C	4.249573	-0.341133	0.996282
C	2.889188	-0.165709	1.213698
C	1.258225	-2.564546	-1.001302
N	-0.006337	-2.208998	-0.644807
C	-1.048937	-2.909024	-1.137158
C	-0.887034	-3.978856	-2.009344
C	0.404745	-4.338232	-2.396693
C	1.484885	-3.625638	-1.885781
C	-0.558066	-1.688797	2.238869
O	-0.805380	-2.414187	3.133440
C	-0.191915	0.942734	1.983116
O	-0.114275	1.848814	2.733920
O	-4.563505	1.741528	-0.001796
C	-5.935165	2.129168	-0.231826
H	-6.285870	2.527422	0.716997
H	-5.988072	2.887954	-1.013718
H	-6.522572	1.258651	-0.525730
H	-1.584450	1.977833	-2.219815
H	-2.028536	-2.590845	-0.803038
H	-1.756495	-4.512629	-2.371163
H	0.570735	-5.164560	-3.078127
H	2.493196	-3.901229	-2.162823
H	3.997966	-2.740502	-1.374876
H	5.716757	-1.434230	-0.158703
H	4.961799	0.248747	1.559786
H	2.522790	0.551175	1.937140
H	0.149225	0.206531	-1.844391
C	0.809909	1.958041	-1.046399
H	1.811357	1.570564	-0.859581
H	0.803275	2.509463	-1.992400
H	0.528647	2.627614	-0.234033

P*ccoh*

C	-1.731207	-1.309902	3.313173
C	-1.354122	-1.241875	1.966828
N	-0.043584	-1.190823	1.609309
C	0.900256	-1.217025	2.570539
C	0.590244	-1.297777	3.923401
C	-0.752527	-1.341369	4.300831
C	-2.320310	-1.233984	0.852485
N	-1.775383	-1.213963	-0.395230
C	-2.606536	-1.194314	-1.459302
C	-3.989773	-1.210538	-1.337629
C	-4.553992	-1.241234	-0.060583
C	-3.707646	-1.252229	1.042425
Re	0.423173	-1.079948	-0.551998
C	0.539603	-1.013195	-2.448526
O	0.557550	-0.996410	-3.624786
N	0.273492	1.110056	-0.262544
C	1.423074	1.690364	-0.063059
C	2.610586	0.897991	-0.095142

C	3.928760	1.451223	0.064973
O	4.978182	0.776417	0.019811
C	2.387289	-0.494146	-0.348700
O	3.463828	-1.290895	-0.402414
C	0.847336	-2.930658	-0.631991
O	1.164940	-4.064217	-0.665433
O	3.973893	2.788631	0.270165
C	5.284471	3.365678	0.427205
H	1.473409	2.765238	0.112807
H	4.276195	-0.729370	-0.271445
H	-2.129861	-1.173665	-2.431285
H	1.925043	-1.173060	2.224681
H	-2.777035	-1.347996	3.585977
H	-4.605143	-1.201368	-2.228499
H	-4.121826	-1.270595	2.041960
H	-5.628833	-1.255268	0.073961
H	1.386216	-1.323962	4.656808
H	-1.034037	-1.400154	5.344999
H	5.116061	4.432962	0.554302
H	5.892551	3.175691	-0.458340
H	5.782409	2.948270	1.303736
C	-0.948011	1.899568	-0.246698
H	-1.608067	1.557430	0.557726
H	-0.736432	2.964028	-0.099934
H	-1.487320	1.767472	-1.189830

TS1*b*

C	1.840367	1.875518	-1.838365
C	0.903787	0.976490	-1.318473
N	-0.216481	1.415133	-0.692096
C	-0.418396	2.738228	-0.561451
C	0.469991	3.684804	-1.058806
C	1.620507	3.242877	-1.710816
C	1.043607	-0.489315	-1.434985
N	0.010371	-1.225908	-0.946781
C	0.046263	-2.567129	-1.065356
C	1.105220	-3.239197	-1.663262
C	2.179632	-2.493800	-2.148931
C	2.148112	-1.107752	-2.030982
Re	-1.571834	-0.129361	0.128240
C	-2.738396	-0.058064	-1.425053
O	-3.452975	-0.053299	-2.348968
N	-0.117184	-0.241868	1.739357
C	1.702018	1.248571	1.969816
C	2.803362	0.886679	1.563016
C	3.943811	0.305930	0.950374
O	4.287024	0.476964	-0.219498
O	4.649292	-0.470205	1.813777
C	5.836009	-1.087853	1.281566
C	-2.729550	1.063896	1.088649
O	-3.406453	1.827742	1.656145
C	-2.525978	-1.642663	0.822440
O	-3.066746	-2.588020	1.242318
H	0.970284	1.858010	2.450365
H	0.536888	-1.003329	1.571955
H	6.256964	-1.658694	2.106544
H	5.583864	-1.747232	0.449449
H	6.542568	-0.328872	0.941760
H	-0.803748	-3.103320	-0.664317
H	1.081966	-4.318571	-1.736034
H	3.029494	-2.981882	-2.611186
H	2.979792	-0.513014	-2.380627
H	2.736391	1.511948	-2.320210
H	2.338042	3.949938	-2.109542
H	0.258402	4.739008	-0.929682
H	-1.322821	3.034820	-0.045850
C	-0.569387	-0.324625	3.125400
H	0.276858	-0.439168	3.816104
H	-1.090224	0.601246	3.400220
H	-1.271123	-1.151384	3.320813

I1*b*

C	-1.687077	2.915687	0.515448
C	-0.860117	1.791269	0.591475
N	0.164389	1.616155	-0.282161
C	0.386337	2.548160	-1.228634
C	-0.388118	3.694300	-1.346454
C	-1.447832	3.878193	-0.458710
C	-1.006497	0.749589	1.627357
N	-0.092374	-0.256466	1.591585
C	-0.133023	-1.217275	2.538747
C	-1.073711	-1.220878	3.559098
C	-2.023112	-0.198948	3.595354
C	-1.990927	0.788772	2.619093
Re	1.366069	-0.215342	-0.055280
C	2.688552	0.733290	0.979752
O	3.490276	1.301020	1.601591
N	-0.299969	-1.281986	-1.217235
C	-1.038161	-0.391087	-2.161736
C	-2.329148	-0.108612	-2.108023
C	-3.306891	-0.497122	-1.156670
O	-3.584343	0.076648	-0.085477
O	-4.082226	-1.565540	-1.586179
C	-5.223988	-1.882588	-0.783335
C	2.433462	-0.016082	-1.648696
O	3.059072	0.132831	-2.618380
C	2.260496	-1.875109	0.336641
O	2.802735	-2.870869	0.602789
H	-0.345230	-0.000083	-2.906225
H	-0.982972	-1.562752	-0.512666
H	-5.753584	-2.669045	-1.320948
H	-4.927302	-2.243700	0.204841
H	-5.870798	-1.011293	-0.660347
H	0.618509	-1.992795	2.466050
H	-1.058989	-2.009930	4.300697
H	-2.778877	-0.173188	4.372051
H	-2.725771	1.581328	2.627378
H	-2.506671	3.038842	1.208785
H	-2.079023	4.755355	-0.524752
H	-0.164084	4.415415	-2.121755
H	1.210628	2.360497	-1.903639
C	0.126056	-2.524370	-1.924982
H	0.845395	-2.259352	-2.700861
H	0.596412	-3.216233	-1.225835
H	-0.739062	-3.002209	-2.391212

I1′*b*

Re	0.755894	-0.817794	-0.396965
N	-0.172318	1.132888	-0.837038
N	2.075937	0.690783	0.526443
C	1.800591	-0.819905	-2.017908
O	2.448198	-0.805562	-2.984210
C	-0.557866	-1.950564	-1.244809
O	-1.339478	-2.619316	-1.785192
C	1.689588	-2.386146	0.210331
O	2.283089	-3.314364	0.588048
C	-1.868647	-0.965160	1.378609
H	-1.969760	-2.039050	1.195585
C	-2.818379	-0.045098	1.462564
C	-4.206677	-0.305916	1.261683
O	-4.833758	-0.229981	0.192726
O	-4.886602	-0.554867	2.439773
C	-6.307697	-0.704723	2.328658
H	-6.759626	0.172848	1.861285
H	-6.673749	-0.817552	3.349116
H	-6.565646	-1.588735	1.740045
C	-1.343869	1.278377	-1.483764
H	-1.838396	0.367885	-1.791301
C	-1.898924	2.524796	-1.752653
H	-2.841322	2.585268	-2.280987
C	-1.226508	3.665053	-1.320492
H	-1.628999	4.653208	-1.509355
C	-0.027751	3.519974	-0.626877
C	0.482726	2.240437	-0.395637
C	1.739237	1.995485	0.342933
C	2.550161	3.022931	0.834859
C	3.713876	2.709220	1.530052
H	4.348598	3.499143	1.913097
C	4.046920	1.368461	1.720593
H	4.940675	1.074719	2.255606
C	3.203876	0.393005	1.202156
H	3.428382	-0.659440	1.317817
H	2.277286	4.057167	0.675452
H	0.500343	4.393069	-0.268859
N	-0.442661	-0.600037	1.525853
H	-0.477741	0.403788	1.715442
C	0.160111	-1.248943	2.720406
H	1.204801	-0.949130	2.818630
H	-0.392805	-0.967475	3.620849
H	0.110357	-2.332003	2.598516

TS2*ccb*

C	-0.344842	3.369235	-0.642911
C	0.309668	2.161668	-0.455653
N	-0.245447	0.969322	-0.835250
C	-1.569305	0.955318	-1.187822
C	-2.207507	2.174576	-1.579338
C	-1.605649	3.370236	-1.287017

C	1.644819	2.067694	0.166596
N	2.082511	0.809350	0.444115
C	3.287391	0.642962	1.030241
C	4.115688	1.707538	1.355639
C	3.680035	3.003373	1.066813
C	2.438769	3.181577	0.469329
Re	0.808839	-0.851529	-0.261535
C	1.789837	-2.292327	0.559433
O	2.390462	-3.144256	1.082906
N	-0.460236	-0.384649	1.608764
C	-1.888148	-0.620533	1.370450
C	-2.619866	0.180405	0.600680
C	-4.007837	-0.015193	0.286311
O	-4.851068	0.258520	1.333364
C	-6.257125	0.242254	1.034186
C	1.842356	-1.038015	-1.868361
O	2.468099	-1.139796	-2.845287
C	-0.455769	-2.140317	-0.938689
O	-1.210690	-2.915690	-1.364083
O	-4.456318	-0.283786	-0.834662
H	-2.223832	-1.543467	1.847391
H	-1.930262	0.035525	-1.625985
H	-3.182704	2.116994	-2.047386
H	-2.082725	4.311122	-1.537119
H	4.301551	3.860475	1.297798
H	5.075780	1.520571	1.819201
H	3.586983	-0.379345	1.221816
H	2.085730	4.175232	0.229570
H	0.116596	4.299976	-0.342587
C	0.012808	-1.007174	2.868836
H	-0.344593	0.622953	1.704388
H	-6.755329	0.535779	1.956836
H	-6.490655	0.949792	0.236099
H	-6.578982	-0.756095	0.731220
H	-0.562725	-0.642807	3.724822
H	-0.099733	-2.089317	2.797508
H	1.068378	-0.770428	3.007878

P*ccb*

C	0.572822	-3.421028	-2.448115
C	0.180444	-2.494304	-1.468949
N	1.092397	-2.009920	-0.580211
C	2.381268	-2.411397	-0.660982
C	2.825621	-3.311022	-1.615678
C	1.895989	-3.829462	-2.525950
C	-1.195916	-1.987762	-1.308919
N	-1.423741	-1.293108	-0.150648
C	-2.632766	-0.464268	-0.076221
C	-3.776676	-1.059296	-0.878936
C	-3.520448	-1.851493	-1.942790
C	-2.171938	-2.242610	-2.253234
C	-2.350092	1.016069	-0.411009
C	-3.518336	1.929418	-0.293083
O	-4.570176	1.610679	0.243102
Re	0.284482	-0.733634	1.026777
C	-0.613897	0.358579	2.333633
O	-1.158113	1.006335	3.135279
C	-1.140237	1.573737	-0.602874
N	0.127793	0.888437	-0.650985
C	1.987679	-0.118967	1.698697
O	3.034786	0.257700	2.055445
C	0.295688	-2.182734	2.274156
O	0.295992	-3.070499	3.029967
O	-3.317616	3.152304	-0.827299
C	-4.392313	4.099936	-0.659707
H	-1.060949	2.651838	-0.699137
H	-5.295088	3.737664	-1.152897
H	-4.042050	5.017422	-1.126758
H	-4.595263	4.257044	0.400523
H	-2.947307	-0.431244	0.975529
H	-4.784167	-0.796203	-0.582857
H	-4.336866	-2.240724	-2.544013
H	2.200934	-4.547089	-3.278854
H	3.868009	-3.601233	-1.639776
H	3.056492	-1.991452	0.073484
H	-0.163572	-3.821992	-3.130477
H	-1.955554	-2.806856	-3.149184
H	0.136758	0.279158	-1.468775
C	1.263893	1.838684	-0.759317
H	1.135153	2.511851	-1.611219
H	1.323797	2.421020	0.159780
H	2.186572	1.273213	-0.877111

TS2*ins*

C	-1.487090	3.461509	0.347471
C	-1.353877	2.077057	0.191946
N	-0.215457	1.434815	0.564107
C	0.801231	2.153868	1.077538
C	0.725180	3.527849	1.270669
C	-0.442582	4.194532	0.901741
C	-2.412223	1.224522	-0.383349
N	-2.101822	-0.091126	-0.509275
C	-3.012622	-0.927496	-1.046823
C	-4.265076	-0.504217	-1.471456
C	-4.599079	0.844336	-1.330518
C	-3.662233	1.714185	-0.784822
Re	-0.152325	-0.782950	0.347156
C	-0.353772	-2.655816	-0.005729
O	-0.525029	-3.788005	-0.232139
C	1.595897	-1.106644	1.086850
O	2.627076	-1.292062	1.594661
C	-0.958842	-1.024944	2.030919
O	-1.438969	-1.174397	3.091786
C	2.028969	0.081076	-1.217433
C	3.317114	0.668674	-0.980269
O	3.530125	1.833785	-0.620153
C	1.412314	-0.134626	-2.367677
O	4.335936	-0.245207	-1.044542
C	5.633779	0.223011	-0.643263
H	1.700764	1.605886	1.318018
H	1.571733	4.054482	1.691697
H	-0.536860	5.265930	1.032989
H	-2.394221	3.963333	0.039001
H	-2.715041	-1.965123	-1.128474
H	-4.959081	-1.219324	-1.894331
H	-5.569740	1.211746	-1.641451
H	-3.902539	2.761999	-0.668015
H	1.802249	0.085565	-3.367460
H	5.982815	1.018274	-1.305235
H	5.608188	0.596552	0.382153
H	6.291027	-0.641973	-0.714800
N	0.087730	-0.725801	-2.324878
H	-0.597450	-0.042654	-2.645838
C	-0.045063	-1.934553	-3.158379
H	-1.079470	-2.283741	-3.128574
H	0.235761	-1.755434	-4.204060
H	0.599027	-2.717826	-2.753998

P*ins*

C	0.054726	-0.408396	-0.943124
N	-0.981145	0.287990	-0.440176
C	-0.732456	1.423349	0.261294
C	0.574576	1.882304	0.456238
C	1.641755	1.155453	-0.061216
C	1.377677	-0.016606	-0.767915
Re	-3.108572	-0.278779	-0.717187
C	-3.045757	-1.470417	0.830369
O	-3.026166	-2.202660	1.738928
C	-1.912016	2.101780	0.833621
N	-3.112617	1.513043	0.594959
C	-4.215336	2.038500	1.162772
C	-4.186728	3.172544	1.964277
C	-2.961255	3.798911	2.191266
C	-1.815225	3.253281	1.624585
C	-2.850411	-1.753786	-1.928044
O	-2.694609	-2.662292	-2.639871
C	-4.997222	-0.539858	-0.860056
O	-6.159290	-0.680967	-0.920843
C	-3.024952	1.163469	-2.435505
C	-1.921256	1.035797	-3.385461
O	-1.181444	0.053444	-3.502021
C	-3.913249	2.165067	-2.726519
N	-5.049816	2.495649	-2.038426
O	-1.712343	2.126491	-4.206805
H	-3.743693	2.819799	-3.579901
H	-0.197968	-1.294168	-1.506688
H	2.173263	-0.618990	-1.187485
H	2.659137	1.497020	0.089285
H	0.756211	2.792658	1.010766
H	-0.852252	3.711630	1.803401
H	-2.896040	4.688486	2.806336
H	-5.106047	3.548036	2.395105
H	-5.144834	1.517816	0.971200
H	-5.381061	1.811332	-1.373608
C	-6.083803	3.317614	-2.650568
C	-0.703494	1.977439	-5.212170
H	-0.735832	2.894364	-5.799512

H	0.283697	1.854554	-4.761230
H	-0.911082	1.114128	-5.847623
H	-6.703589	3.770779	-1.874663
H	-5.611555	4.120136	-3.221187
H	-6.734056	2.749035	-3.328070

Figure S2. PCM-B3LYP/6-31+G(d,p) (LANL2DZ for Re) optimized geometries in THF solution of the critical structures involved in the reaction between the complex [Re(NHMe)(CO)$_3$(bipy)] (bipy = 2,2'-bipyridine) and methyl propiolate (HMAD, HC≡CCO$_2$Me). Relevant distances are given in angstroms.

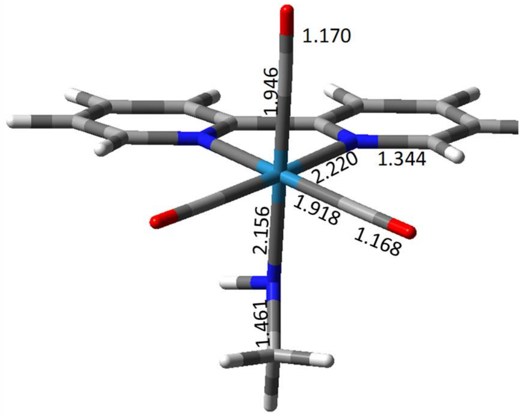

[Re(NHMe)(CO)$_3$(bipy)]

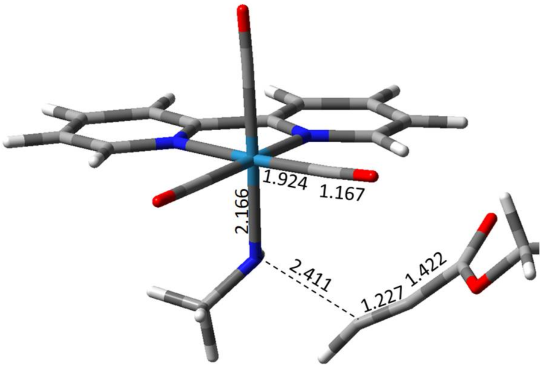

TS1

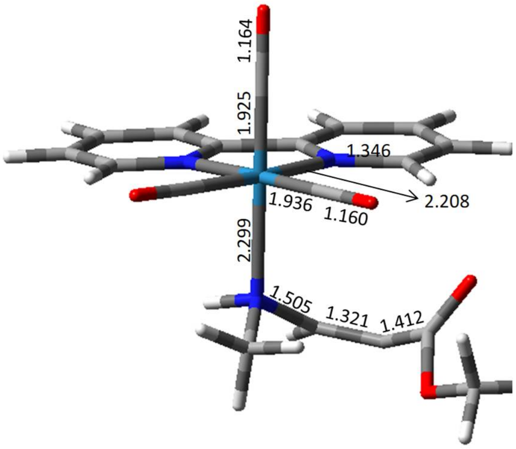

I1

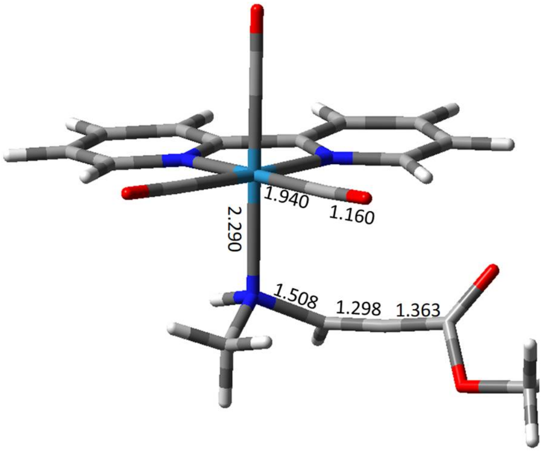

TS1'

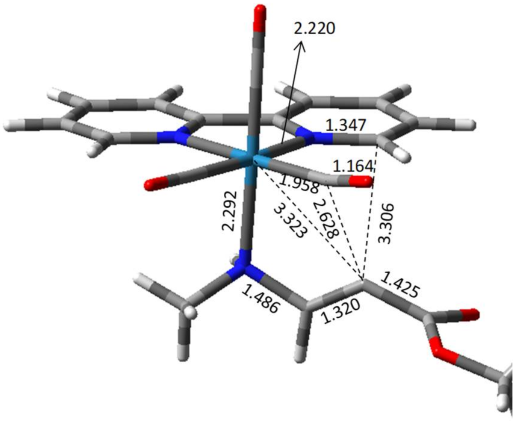

I1'

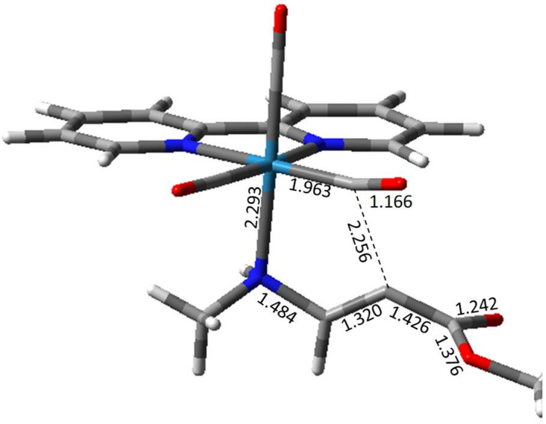

TS2*cco*

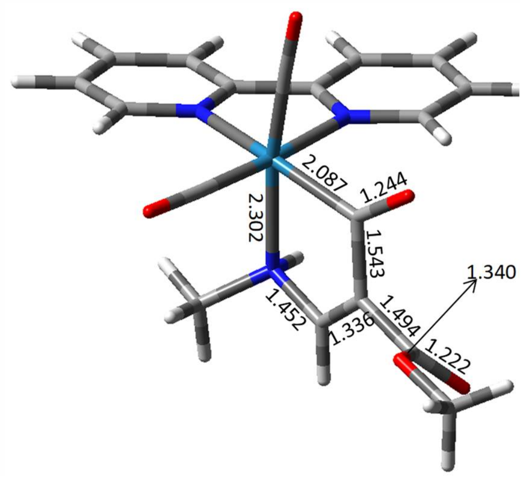

P*cco*

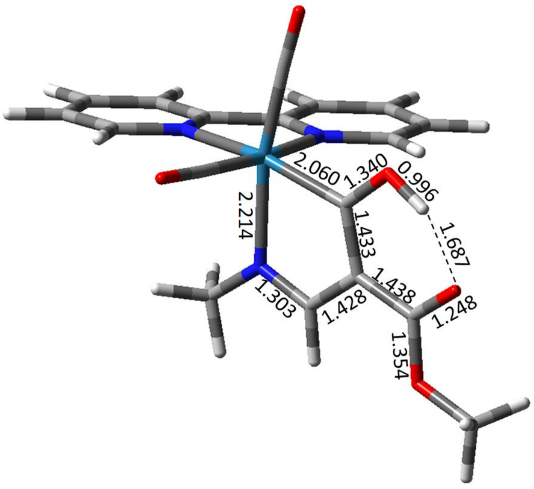

P*ccoh*

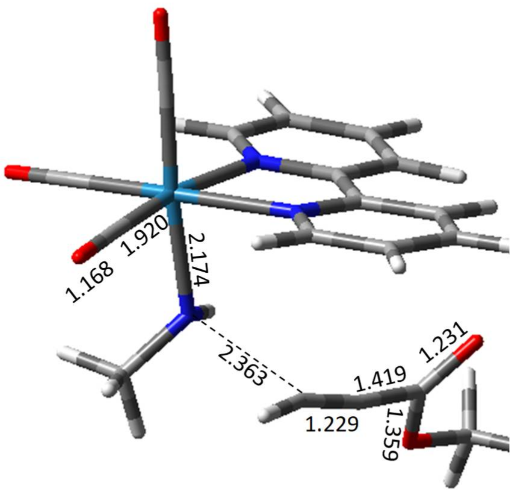

TS1*b*

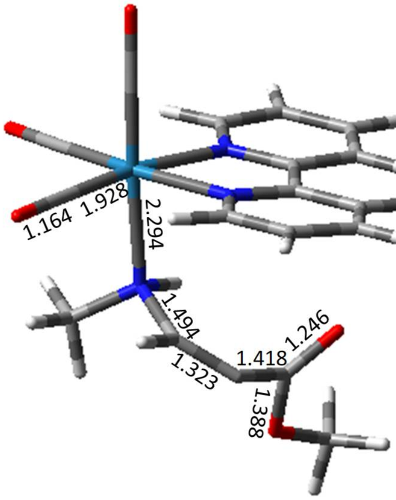

I1*b*

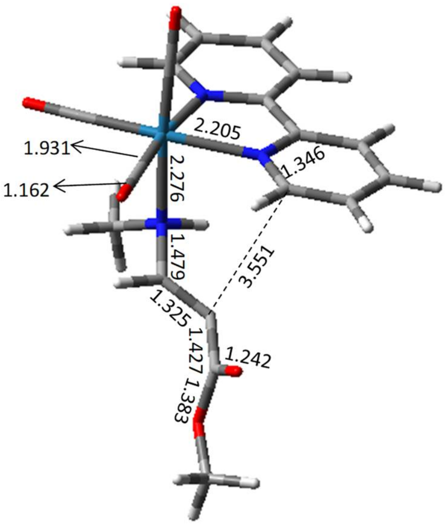

I1'*b*

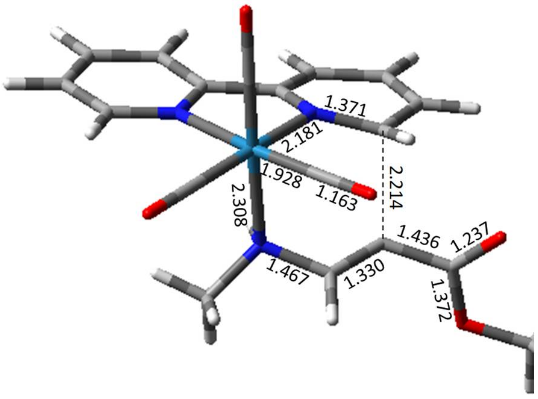

TS2*ccb*

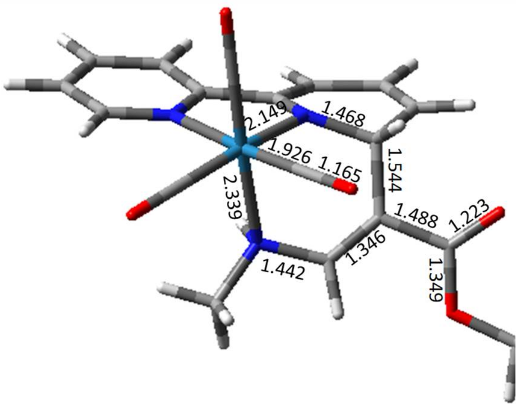

P*ccb*

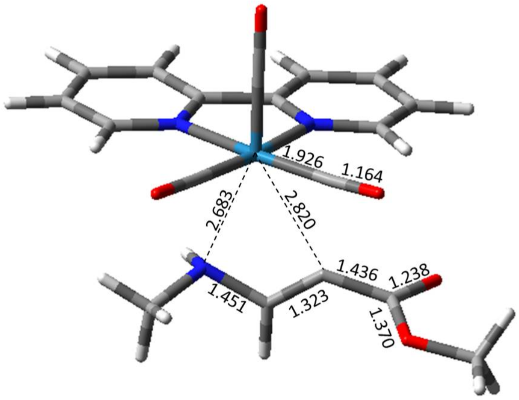

TS2*ins*

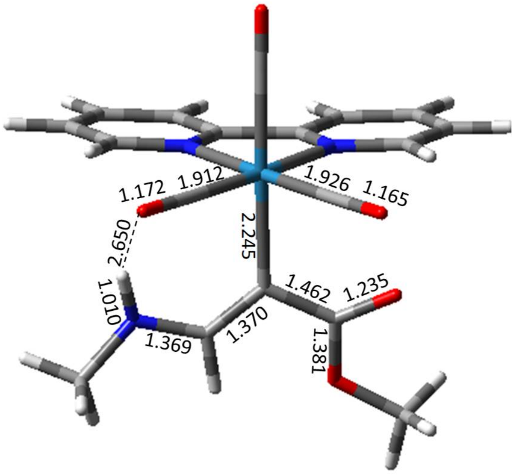

P*ins*

Table S7. PCM-B3LYP/6-31+G(d,p) (LANL2DZ for Re) energies without and with including thermal corrections (E and G, respectively), enthalpies (H), and entropies (S), and CPCM-DLPNO-CCSD(T)/def2-TZVPP//PCM-B3LYP/6-31+G(d,p) (LANL2DZ for Re) energies without and with including thermal corrections (E' and G', respectively) in THF solution of the critical structures involved in the reaction between the complex [Re(OPh)$(CO)_3$(bipy)] (bipy = 2,2'-bipyridine) and methyl propiolate (HMAD, $HC{\equiv}CCO_2Me$). All the values are given in hartree, except entropies that are in cal/K mol.[a]

Species	E	H	S	G	E'	G'[b]
[Re(OPh)$(CO)_3$(bipy)]	-1221.543852	-1221.238633	166.181	-1221.317591	-1218.594171	-1218.367910
HMAD	-305.215726	-305.136831	77.228	-305.173524	-304.738441	-304.696239
Reactants	-1526.759578	-1526.375464	243.409	-1526.491115	-1523.332612	-1523.064149
**TS1**	-1526.724819	-1526.339212	193.723	-1526.431256	-1523.304340	-1523.010777
**I1**	-1526.728962	-1526.341480	194.612	-1526.433947	-1523.308282	-1523.013267
**P*cco***	-1526.751863	-1526.362806	190.501	-1526.453319	-1523.324091	-1523.025547
**TS2*ccb***	-1526.715368	-1526.329296	191.904	-1526.420475	-1523.297168	-1523.002275
**P*ccb***	-1526.747252	-1526.358932	192.520	-1526.450405	-1523.335175	-1523.038328
**TS2*ins***	-1526.727035	-1526.340180	191.252	-1526.431051	-1523.301919	-1523.005935
**P*ins***	-1526.790063	-1526.401199	194.623	-1526.493671	-1523.369479	-1523.073087

[a] Thermal magnitudes were computed in THF solution at 298.15 K and 1 atm. [b] For each species, G' was calculated as G' = G – E + E', in which G is the PCM-B3LYP/6-31+G(d,p) (LANL2DZ for Re) energy with including thermal corrections and E and E' are the PCM-B3LYP/6-31+G(d,p) (LANL2DZ for Re) and CPCM-DLPNO-CCSD(T)/def2-TZVPP//PCM-B3LYP/6-31+G(d,p) (LANL2DZ for Re) energies without including thermal corrections, respectively.

Table S8. PCM-B3LYP/6-31+G(d,p) (LANL2DZ for Re) relative energies without and with including thermal corrections (ΔE and ΔG, respectively), enthalpies (ΔH), and entropic contributions (TΔS), and CPCM-DLPNO-CCSD(T)/def2-TZVPP//PCM-B3LYP/6-31+G(d,p) (LANL2DZ for Re) relative energies without and with including thermal corrections (E' and G', respectively) in THF solution of the critical structures involved in the reaction between the complex [Re(OPh)$(CO)_3$(bipy)] (bipy = 2,2'-bipyridine) and methyl propiolate (HMAD, HC≡CCO_2Me). All the values are given in kcal/mol.[a]

Species	ΔE	ΔH	TΔS	ΔG	ΔE'	ΔG'
[Re(OPh)$(CO)_3$(bipy)] + HMAD	0.0	0.0	0.0	0.0	0.0	0.0
**TS1**	21.8	22.7	-14.9	37.6	17.7	33.5
**I1**	19.2	21.3	-14.6	35.9	15.3	31.9
**P*cco***	4.8	7.9	-15.8	23.7	5.3	24.2
**TS2*ccb***	27.7	29.0	-15.3	44.3	22.2	38.8
**P*ccb***	7.7	10.4	-15.1	25.5	-1.6	16.2
**TS2*ins***	20.4	22.1	-15.6	37.7	19.3	36.5
**P*ins***	-19.1	-16.1	-14.5	-1.6	-23.1	-5.6

[a] Thermal magnitudes were evaluated in THF solution at 298.15 K and 1 atm.

Table S9. PCM-B3LYP/6-31+G(d,p) (LANL2DZ for Re) optimized cartesian coordinates, in Å, for the critical structures involved in the reaction of the complex [Re(OPh)$(CO)_3$(bipy)] (bipy = 2,2'-bipyridine) towards methyl propiolate (HMAD, HC≡CCO_2Me).

[Re(OPh)$(CO)_3$(bipy)]

Re	0.033096	-0.898547	-0.070971
C	-1.042853	-1.618167	-1.493557
O	-1.660572	-2.041318	-2.386037
C	-0.970902	-1.901353	1.227948
O	-1.534264	-2.512482	2.044748
N	1.307097	0.415559	-1.311243
C	1.267695	0.456538	-2.655208
C	2.049008	1.331139	-3.402429
C	2.903124	2.203526	-2.729803
C	2.948543	2.163819	-1.339595
C	2.141322	1.255419	-0.647070
C	2.159432	1.122309	0.825782
N	1.342247	0.173675	1.350119
C	1.328228	-0.016807	2.681817
C	2.125095	0.716806	3.553821
C	2.968075	1.692700	3.025188
C	2.984238	1.895753	1.648705
C	1.242515	-2.380960	-0.253721
O	1.969671	-3.286614	-0.374252
H	0.588501	-0.235810	-3.135861
H	1.979893	1.322976	-4.482186
H	3.525372	2.902547	-3.275489
H	3.607877	2.831157	-0.801763
H	3.634750	2.646258	1.221437
H	3.603733	2.287070	3.670732
H	2.077456	0.521993	4.617567
H	0.655977	-0.780853	3.049357
O	-1.040534	0.935920	0.117388
C	-2.341121	1.209961	0.133623
C	-2.746154	2.568652	0.204304
C	-4.093236	2.924832	0.220569
C	-5.096306	1.947467	0.169983
C	-4.714099	0.604043	0.104361
C	-3.366221	0.234393	0.087134
H	-1.974483	3.331354	0.244866
H	-4.363913	3.975383	0.275553
H	-6.143751	2.227452	0.180940
H	-5.472662	-0.173565	0.065221
H	-3.099037	-0.814075	0.034427

TS1

C	0.600567	-3.522706	-1.845330
C	0.724227	-2.436070	-0.975283
N	-0.373053	-1.831623	-0.445007
C	-1.596803	-2.278402	-0.787272
C	-1.783041	-3.358068	-1.645437
C	-0.665959	-3.997890	-2.175746
C	2.031028	-1.876504	-0.573569
N	1.988754	-0.803430	0.259346
C	3.143812	-0.237271	0.659595
C	4.388356	-0.718838	0.272956
C	4.443492	-1.828255	-0.569894
C	3.253272	-2.410919	-0.995015
Re	0.000175	-0.154297	0.944599
C	0.068985	-1.292100	2.467165
O	0.111859	-1.992313	3.398910
O	0.066723	1.037589	-0.962923
C	-1.242445	1.224965	-1.880570
C	-2.392165	0.925675	-1.434538
C	-3.710161	0.598431	-1.174700
O	-4.226618	-0.533050	-1.281243
C	0.966684	2.089852	-1.114559
C	2.151881	1.887517	-1.825584
C	3.030988	2.959368	-2.015222
C	2.726859	4.222551	-1.500109
C	1.537302	4.413524	-0.787643
C	0.655266	3.349732	-0.593236
O	-4.449193	1.667587	-0.709193
C	-5.746570	1.339076	-0.193519
C	-1.838749	0.267924	1.409204
O	-2.917149	0.462518	1.795310
C	0.585900	1.311994	2.043120
O	0.973394	2.175521	2.721168
H	-0.810607	1.596847	-2.801389
H	-6.368687	0.868930	-0.958003
H	-6.185835	2.286035	0.117841
H	-5.655505	0.666899	0.662862
H	-2.449110	-1.742868	-0.390839
H	-2.789306	-3.673995	-1.887398
H	-0.774064	-4.842797	-2.844866
H	5.394612	-2.238883	-0.887422
H	5.285655	-0.228820	0.627499
H	3.052433	0.616505	1.317080
H	3.276450	-3.273332	-1.646237
H	1.480562	-3.995572	-2.259343
H	2.371108	0.902720	-2.222425
H	-0.274520	3.483215	-0.052183
H	3.950979	2.806633	-2.569563
H	1.295278	5.390333	-0.383500
H	3.409030	5.051494	-1.652504

I1

C	3.102666	-2.273636	-1.631963
C	1.979114	-1.817107	-0.934716
N	2.060484	-0.750009	-0.096154
C	3.250182	-0.136506	0.065643
C	4.403048	-0.546253	-0.592629
C	4.325252	-1.632004	-1.464493
C	0.651902	-2.456088	-1.043301
N	-0.338376	-1.903789	-0.294819
C	-1.576794	-2.429634	-0.356289
C	-1.879918	-3.537931	-1.139464
C	-0.868780	-4.116253	-1.903815
C	0.408374	-3.565467	-1.858588
Re	0.225816	-0.195618	0.996702
C	-1.555962	1.158706	-1.385385
C	-2.509622	0.624486	-0.676072
C	-3.880760	0.416404	-0.969580
O	-4.376184	-0.599782	-1.482895
C	0.957476	1.298486	1.957108
O	1.444717	2.185529	2.534433
C	0.667901	-1.310175	2.460165
O	0.942061	-1.995721	3.365321
C	-1.516968	0.138897	1.810724
O	-2.472732	0.275861	2.457299
O	-4.692166	1.425918	-0.500489
C	-6.103854	1.204588	-0.629440
H	-1.532631	1.644643	-2.358962
H	-6.384397	1.068024	-1.675931
H	-6.579870	2.097292	-0.226018
H	-6.413320	0.322598	-0.063554
H	-1.068225	-4.976889	-2.531179
H	5.201199	-1.980348	-1.998296
H	5.333019	-0.017923	-0.424939
H	3.267754	0.700618	0.750088
H	3.024173	-3.120766	-2.298971
H	1.204259	-3.995498	-2.450327
H	-2.891646	-3.921179	-1.151425
H	-2.338739	-1.922803	0.218807
C	0.570918	2.268738	-1.083617
C	1.749970	2.206544	-1.823833
C	0.129479	3.469758	-0.525833
C	2.512339	3.367143	-1.990686
H	2.058932	1.263836	-2.259400
C	0.895927	4.623633	-0.701499
H	-0.798061	3.488968	0.033488
C	2.091120	4.574281	-1.427124
H	3.431964	3.325432	-2.563696
H	0.562203	5.558682	-0.264964
H	2.684869	5.472035	-1.556987
O	-0.167013	1.090384	-0.889348

P*cco*

C	1.189673	4.938165	-1.065624
C	1.816962	3.790867	-1.560993

C	1.237321	2.532871	-1.379964
C	0.034797	2.450469	-0.684220
C	-0.602176	3.575493	-0.166306
C	-0.015966	4.827879	-0.368372
O	-0.541067	1.173144	-0.510626
C	-1.900134	1.070512	-0.753859
C	-2.599858	0.103108	-0.145039
C	-4.015166	-0.096286	-0.558747
O	-4.550708	-1.180148	-0.705616
C	-1.925504	-0.780080	0.928769
O	-2.688907	-1.510749	1.577696
Re	0.148201	-0.526198	0.949050
C	0.082917	0.623694	2.456180
O	0.083248	1.335924	3.393341
N	2.246279	-0.213776	0.306307
C	2.648088	-0.815016	-0.847310
C	3.937928	-0.622506	-1.355282
C	4.835738	0.188892	-0.668625
C	4.419971	0.796403	0.516693
C	3.122648	0.573810	0.963290
C	1.644383	-1.680465	-1.500106
C	1.905325	-2.413956	-2.663232
C	0.914588	-3.230846	-3.199136
C	-0.318322	-3.308729	-2.551930
C	-0.518499	-2.550355	-1.404311
N	0.431541	-1.745519	-0.887473
C	0.450096	-1.955827	2.108340
O	0.619435	-2.866736	2.837711
O	-4.656396	1.073854	-0.786474
C	-6.014006	0.963094	-1.264422
H	-1.459200	-2.581335	-0.869575
H	-1.116274	-3.937971	-2.924169
H	1.105601	-3.802058	-4.099993
H	2.871277	-2.350612	-3.146212
H	2.756653	1.027770	1.875055
H	5.082372	1.431846	1.090855
H	5.838019	0.339363	-1.050224
H	4.241923	-1.106777	-2.273676
H	-2.282626	1.786961	-1.470241
H	-6.038586	0.416936	-2.208552
H	-6.629641	0.443962	-0.529330
H	-6.357610	1.985748	-1.400406
H	1.701027	1.636818	-1.773554
H	-1.526853	3.474932	0.390040
H	2.752469	3.872534	-2.102835
H	-0.503066	5.712456	0.025592
H	1.640782	5.912104	-1.217831

TS2*ccb*

H	-0.554431	-0.884856	0.167765
C	-0.112952	0.052868	0.476468
C	-0.784675	1.256699	0.231274
C	-2.090647	1.338851	-0.451807
C	-2.710266	0.239986	-1.027961
H	-2.259137	-0.741387	-0.982666
N	-2.660653	2.577115	-0.463612
C	-3.753122	2.798900	-1.262173
C	-4.503183	1.678172	-1.747038
C	-3.970750	0.418379	-1.642457
C	1.114778	0.067751	1.127949
C	1.659415	1.291114	1.524093
C	0.950332	2.451963	1.248922
N	-0.242705	2.442787	0.619176
Re	-1.445156	4.239581	0.213514
O	-0.706987	3.979374	-1.955546
C	-1.574398	3.999978	-3.123968
C	-2.855475	3.713267	-3.052375
C	-3.744335	3.696028	-4.179973
O	-4.235912	4.936230	-4.496692
C	-5.229895	4.970744	-5.534987
C	-2.100381	4.297251	1.992237
O	-2.507002	4.319737	3.084443
C	-2.648631	5.669242	-0.275932
O	-3.385838	6.523431	-0.552046
C	-0.105692	5.553675	0.680289
O	0.717661	6.321461	0.975175
O	-4.149949	2.684273	-4.759661
H	-0.974377	4.255777	-3.993852
H	-4.836969	4.549637	-6.462123
H	-5.474168	6.023195	-5.670191
H	-6.118690	4.410588	-5.236928
H	-4.276142	3.725481	-1.079526
H	-5.454488	1.855296	-2.231559
H	-4.507395	-0.441141	-2.028491
H	1.637607	-0.860466	1.325948
H	2.610432	1.350721	2.036908
H	1.332822	3.423286	1.534564
C	0.631423	4.315765	-2.221410
C	1.602578	3.323038	-2.118628
C	2.938875	3.658666	-2.356422
C	3.285899	4.967490	-2.703873
C	2.295000	5.948239	-2.811232
C	0.957343	5.626093	-2.568963
H	1.311740	2.312594	-1.856568
H	3.704310	2.894777	-2.277246
H	4.323482	5.222210	-2.889446
H	2.559186	6.964887	-3.081638
H	0.175144	6.372604	-2.642561

P*ccb*

C	3.111916	-1.206382	-0.757457
N	1.770427	-1.273433	-0.599811
C	1.025198	-1.997381	-1.480011
C	1.647170	-2.677420	-2.539096
C	3.022669	-2.599702	-2.700272
C	3.775251	-1.846425	-1.790696
Re	0.662992	-0.357748	1.051161
C	-1.531581	1.316495	-0.774325
C	-2.464276	0.396774	-0.466738
C	-2.224216	-1.028756	0.071346
N	-0.809249	-1.407908	-0.051290
C	-0.424137	-2.006098	-1.223489
C	-1.309437	-2.624649	-2.082829
C	-2.675069	-2.755407	-1.656643
C	-3.124902	-2.062889	-0.586983
C	1.214699	-1.641937	2.317107
O	1.543531	-2.460625	3.081843
C	-0.503172	0.436087	2.367939
O	-1.207285	0.911828	3.163970
C	2.109928	0.793811	1.634720
O	3.000729	1.482244	1.938766
C	-3.851953	0.927014	-0.589517
O	-4.721002	0.233254	0.169413
C	-6.094236	0.674204	0.126047
O	-4.189682	1.890685	-1.263927
H	-1.818591	2.293332	-1.146874
H	-6.169581	1.708078	0.463670
H	-6.632053	0.009025	0.797599
H	-6.481368	0.593351	-0.890423
H	-2.468039	-0.996213	1.138682
H	-4.122429	-2.200225	-0.189840
H	-3.323052	-3.460185	-2.169218
H	3.506408	-3.121950	-3.517406
H	4.850772	-1.759809	-1.875462
H	3.647976	-0.619024	-0.023351
H	1.050667	-3.268331	-3.220543
H	-0.971988	-3.094979	-2.995306
C	0.606626	2.306146	-0.952660
C	1.293432	2.332043	-2.160612
C	0.676650	3.343489	-0.028446
C	2.086334	3.447626	-2.448630
H	1.208091	1.500702	-2.850194
C	1.470898	4.452204	-0.330446
H	0.136108	3.268973	0.908899
C	2.174164	4.504964	-1.538298
H	2.629585	3.488858	-3.385954
H	1.541404	5.268954	0.378772
H	2.791540	5.366325	-1.768045
O	-0.163769	1.157700	-0.639369

TS2*ins*

C	-1.826015	-3.394885	-1.291728
C	-1.586067	-2.124129	-0.757868
N	-0.558754	-1.905661	0.105165
C	0.245672	-2.937944	0.430771
C	0.063544	-4.221792	-0.068312
C	-0.995547	-4.455153	-0.945718
C	-2.427379	-0.952513	-1.068381
C	-3.470794	-0.991590	-1.999138
C	-4.215038	0.157752	-2.244955
C	-3.890644	1.328380	-1.561231
C	-2.839653	1.304986	-0.652569
N	-2.126256	0.190407	-0.396722
Re	-0.468880	0.087111	1.070736
C	-1.569815	-0.484493	2.477188
O	-2.260930	-0.824620	3.360290
C	1.064757	-0.237607	2.180827
O	1.989407	-0.477870	2.848419
C	-0.546907	1.924655	1.700903
O	-0.674129	2.965646	2.207155

C	0.266742	2.460916	-0.627893
C	1.080248	1.685209	-1.299184
C	-0.163531	3.800631	-0.870782
O	0.644154	4.737958	-0.283569
C	0.181828	6.097073	-0.344634
O	-1.214925	4.132452	-1.434907
H	-2.536679	2.200897	-0.129514
H	-4.428449	2.252615	-1.728554
H	-5.024954	0.139722	-2.964392
H	-3.694806	-1.906066	-2.531343
H	1.049649	-2.716748	1.119464
H	0.739821	-5.012495	0.230265
H	-1.177480	-5.444479	-1.347770
H	-2.656762	-3.553684	-1.964696
H	1.680939	1.864041	-2.188611
H	0.062452	6.420043	-1.380416
H	-0.773563	6.202389	0.173502
H	0.949124	6.688003	0.152955
C	2.462956	-0.235976	-0.878442
C	2.633236	-1.505780	-1.431237
C	3.536657	0.445893	-0.299718
C	3.894009	-2.106911	-1.393908
H	1.784961	-2.006224	-1.882625
C	4.794186	-0.161691	-0.274768
H	3.382186	1.429872	0.127140
C	4.977216	-1.438370	-0.815608
H	4.029708	-3.093025	-1.824738
H	5.629534	0.362475	0.176138
H	5.956257	-1.904955	-0.790062
O	1.179848	0.313112	-0.884368

Pins

C	-1.169834	-2.962582	-2.267254
C	-1.286202	-1.904789	-1.358031
N	-0.387238	-1.742011	-0.353375
C	0.629789	-2.617226	-0.239556
C	0.798373	-3.689962	-1.106535
C	-0.116680	-3.862713	-2.144880
C	-2.384679	-0.920006	-1.406499
C	-3.382538	-0.939869	-2.386454
C	-4.393839	0.014763	-2.357652
C	-4.386673	0.972598	-1.345347
C	-3.364171	0.943424	-0.403280
N	-2.383045	0.021152	-0.425462
Re	-0.738443	-0.074475	1.059470
C	-1.785459	-1.223201	2.236666
O	-2.405461	-1.891916	2.964793
C	0.821106	-0.332327	2.145867
O	1.769653	-0.519022	2.800115
C	-1.199130	1.463545	2.120891
O	-1.491408	2.376978	2.782997
C	0.391417	1.222738	-0.366386
C	1.671663	1.069418	-0.757580
C	-0.260899	2.433169	-0.907203
O	0.247932	2.875864	-2.097706
C	-0.338482	4.075934	-2.628107
O	-1.201272	3.023888	-0.380971
H	-3.304827	1.680515	0.384967
H	-5.152649	1.735671	-1.281148
H	-5.171754	0.008111	-3.112782
H	-3.370807	-1.689727	-3.166688
H	1.321059	-2.436523	0.572065
H	1.631709	-4.366172	-0.966387
H	-0.017223	-4.685723	-2.842072
H	-1.894691	-3.082732	-3.060619
H	2.205821	1.760009	-1.406226
H	-1.395030	3.917859	-2.853884
H	-0.244939	4.899300	-1.918164
H	0.214655	4.294290	-3.540010
C	3.800657	0.095606	-0.410793
C	4.453905	1.265968	-0.011314
C	4.529512	-1.030117	-0.805813
C	5.851636	1.303284	-0.019838
H	3.880031	2.124441	0.317442
C	5.924183	-0.980075	-0.807651
H	3.997314	-1.923236	-1.112013
C	6.591127	0.186442	-0.417719
H	6.359335	2.208919	0.294308
H	6.488401	-1.852325	-1.119814
H	7.674246	0.222212	-0.421274
O	2.424850	-0.024372	-0.388765

Figure S3. PCM-B3LYP/6-31+G(d,p) (LANL2DZ for Re) optimized geometries in THF solution of the critical structures involved in the reaction between the complex $[Re(OPh)(CO)_3(bipy)]$ (bipy = 2,2'-bipyridine) and methyl propiolate (HMAD, $HC{\equiv}CCO_2Me$). Relevant distances are given in angstroms.

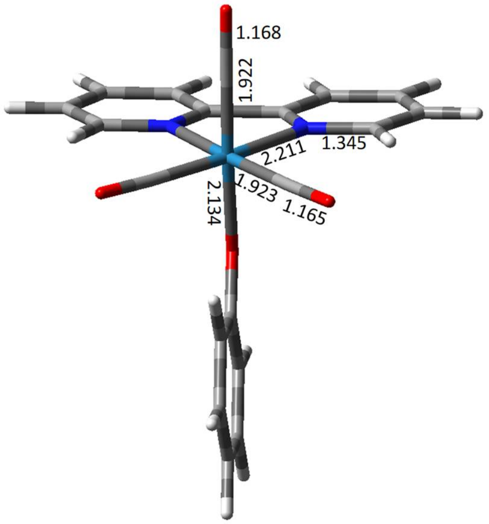

$[Re(OPh)(CO)_3(bipy)]$

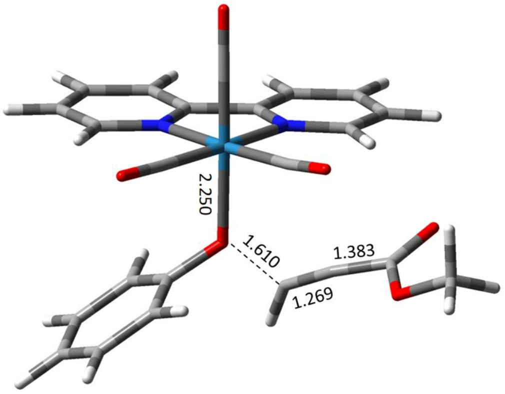

TS1

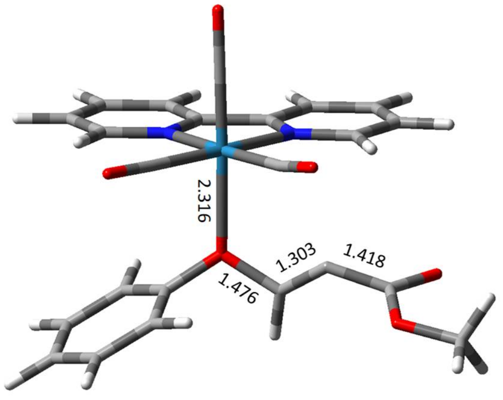

I1

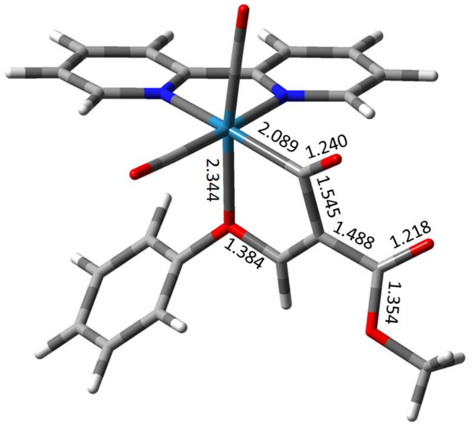

P*cco*

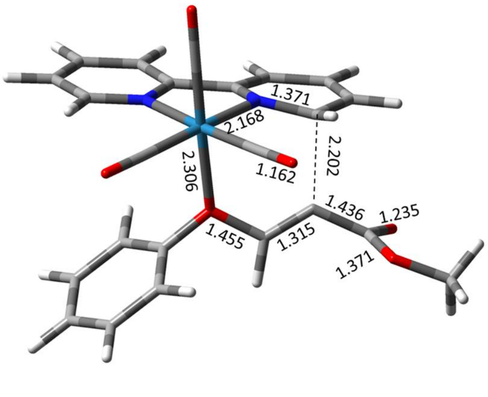

TS2*ccb*

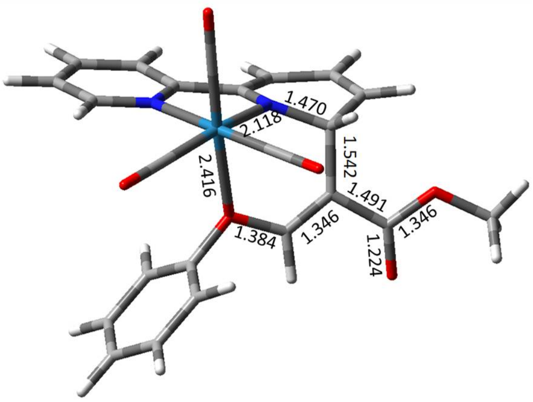

P*ccb*

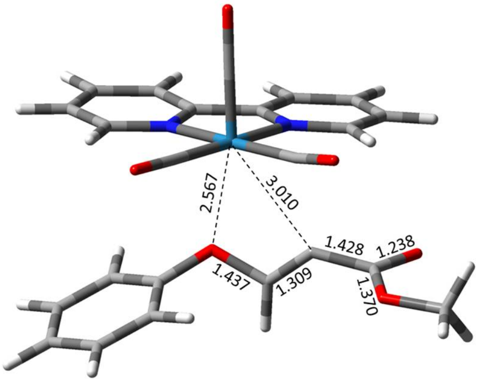

TS2*ins*

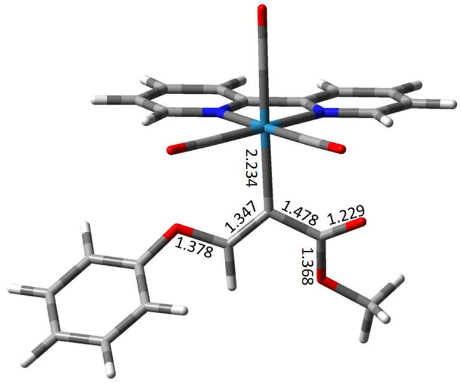

P*ins*

Table S10. PCM-B3LYP/6-31+G(d,p) (LANL2DZ for Re) energies without and with including thermal corrections (E and G, respectively), enthalpies (H), and entropies (S), and CPCM-DLPNO-CCSD(T)/def2-TZVPP//PCM-B3LYP/6-31+G(d,p) (LANL2DZ for Re) energies without and with including thermal corrections (E' and G', respectively) in THF solution of the critical structures involved in the reaction between the complex [Re(PH_2)$(CO)_3$(bipy)] (bipy = 2,2'-bipyridine) and methyl propiolate (HMAD, HC≡CCO_2Me). All the values are given in hartree, except entropies that are in cal/K mol.[a]

Species	E	H	S	G	E'	G'[b]
[Re(PH_2)$(CO)_3$(bipy)]	-1257.202102	-1256.977138	146.178	-1257.046592	-1254.334109	-1254.178599
HMAD	-305.215726	-305.136831	77.228	-305.173524	-304.738441	-304.696239
Reactants	-1562.417828	-1562.113969	223.406	-1562.220116	-1559.072550	-1558.874838
**TS1**	-1562.402073	-1562.097122	178.738	-1562.182046	-1559.050814	-1558.830787
**I1**	-1562.422310	-1562.114717	174.143	-1562.197458	-1559.083274	-1558.858422
**TS1'**	-1562.417049	-1562.111386	172.879	-1562.193526	-1559.075151	-1558.851628
**I1'**	-1562.423524	-1562.116212	176.766	-1562.200199	-1559.083705	-1558.860380
**TS2*cco***	-1562.418924	-1562.113594	175.034	-1562.196759	-1559.075519	-1558.853354
**P*cco***	-1562.443788	-1562.135234	171.074	-1562.216517	-1559.101934	-1558.874663
**P*ccoh***	-1562.458506	-1562.148179	171.400	-1562.229616	-1559.103233	-1558.874343
**TS2*ccb***	-1562.414688	-1562.109110	173.911	-1562.191741	-1559.076249	-1558.853302
**P*ccb***	-1562.443253	-1562.135720	172.478	-1562.217670	-1559.115621	-1558.890038
**TS2*ins***	-1562.397685	-1562.092377	175.443	-1562.175736	-1559.042341	-1558.820392
**P*ins***	-1562.451667	-1562.143819	173.328	-1562.226172	-1559.121256	-1558.895761

[a] Thermal magnitudes were computed in THF solution at 298.15 K and 1 atm. [b] For each species, G' was calculated as G' = G – E + E', in which G is the PCM-B3LYP/6-31+G(d,p) (LANL2DZ for Re) energy with including thermal corrections and E and E' are the PCM-B3LYP/6-31+G(d,p) (LANL2DZ for Re) and CPCM-DLPNO-CCSD(T)/def2-TZVPP//PCM-B3LYP/6-31+G(d,p) (LANL2DZ for Re) energies without including thermal corrections, respectively.

Table S11. PCM-B3LYP/6-31+G(d,p) (LANL2DZ for Re) relative energies without and with including thermal corrections (ΔE and ΔG, respectively), enthalpies (ΔH), and entropic contributions (TΔS), and CPCM-DLPNO-CCSD(T)/def2-TZVPP//PCM-B3LYP/6-31+G(d,p) (LANL2DZ for Re) relative energies without and with including thermal corrections (E' and G', respectively) in THF solution of the critical structures involved in the reaction between the complex [Re(PH_2)$(CO)_3$(bipy)] (bipy = 2,2'-bipyridine) and methyl propiolate (HMAD, HC≡CCO_2Me). All the values are given in kcal/mol.[a]

Species	ΔE	ΔH	TΔS	ΔG	ΔE'	ΔG'
[Re(PH_2)$(CO)_3$(bipy)] + HMAD	0.0	0.0	0.0	0.0	0.0	0.0
**TS1**	9.9	10.6	-13.3	23.9	13.6	27.6
**I1**	-2.8	-0.5	-14.7	14.2	-6.7	10.3
**TS1'**	0.5	1.6	-15.1	16.7	-1.6	14.6
**I1'**	-3.6	-1.4	-13.9	12.5	-7.0	9.1
**TS2*cco***	-0.7	0.2	-14.4	14.7	-1.9	13.5
**P*cco***	-16.3	-13.3	-15.6	2.3	-18.4	0.1
**P*ccoh***	-25.5	-21.5	-15.5	-6.0	-19.3	0.3
**TS2*ccb***	2.0	3.0	-14.8	17.8	-2.3	13.5
**P*ccb***	-16.0	-13.6	-15.2	1.5	-27.0	-9.5
**TS2*ins***	12.6	13.5	-14.3	27.8	19.0	34.2
**P*ins***	-21.2	-18.7	-14.9	-3.8	-30.6	-13.1

[a] Thermal magnitudes were evaluated in THF solution at 298.15 K and 1 atm.

Table S12. PCM-B3LYP/6-31+G(d,p) (LANL2DZ for Re) optimized cartesian coordinates, in Å, for the critical structures involved in the reaction of the complex [Re(PH_2)($CO)_3$(bipy)] (bipy = 2,2'-bipyridine) towards methyl propiolate (HMAD, HC≡CCO_2Me).

[Re(PH_2)($CO)_3$(bipy)]

Re	0.882728	0.017094	0.085533
C	1.068598	0.044679	2.017991
O	1.232328	0.045774	3.173087
C	2.213804	1.383401	-0.144680
O	2.996930	2.233711	-0.306132
C	2.240483	-1.325064	-0.123268
O	3.045911	-2.155683	-0.273091
P	0.516837	-0.070998	-2.472921
C	-2.102874	0.733585	0.045678
C	-0.823376	2.682064	0.082257
C	-3.279970	1.488019	-0.026528
C	-1.954382	3.485595	0.016087
H	0.167503	3.115286	0.123870
C	-3.208557	2.876208	-0.041598
H	-4.241884	0.995564	-0.076488
H	-1.845281	4.562315	0.007359
H	-4.113153	3.468789	-0.099733
C	-2.092049	-0.740359	0.071794
C	-0.781771	-2.669297	0.131608
C	-3.258196	-1.515209	0.060534
C	-1.900888	-3.491227	0.116324
H	0.216389	-3.085877	0.161818
C	-3.165847	-2.902056	0.081986
H	-4.228647	-1.038143	0.035930
H	-1.774653	-4.566089	0.132490
H	-4.062832	-3.508598	0.072057
N	-0.863671	-1.323202	0.114296
N	-0.884525	1.335331	0.093538
H	0.389116	-1.483730	-2.622119
H	1.842472	-0.053938	-2.991238

TS1

C	3.844149	1.749735	0.539021
C	2.588837	1.216743	0.223520
N	2.344016	-0.115178	0.347412
C	3.327785	-0.923350	0.791467
C	4.592704	-0.453930	1.121282
C	4.856254	0.909901	0.990066
C	1.458477	2.036020	-0.253322
N	0.305282	1.361275	-0.511643
C	-0.783943	2.052924	-0.899784
C	-0.764600	3.430283	-1.089938
C	0.421698	4.125986	-0.863836
C	1.540262	3.421141	-0.429916
Re	0.358946	-0.834990	-0.286459
P	-0.423324	-0.429780	2.110537
C	0.620953	-2.685394	0.158602
O	0.811525	-3.794703	0.462197
C	-1.451839	-1.223756	-0.819054
O	-2.543877	-1.451202	-1.153370
C	0.987143	-1.104236	-2.105477
O	1.353746	-1.309332	-3.190986
C	-2.300873	1.066952	2.607046
C	-3.232377	1.065948	1.778447
C	-3.993004	0.817654	0.613371
O	-3.924712	1.469156	-0.434724
O	-4.861076	-0.217652	0.771327
C	-5.675463	-0.533997	-0.373128
H	-1.929773	1.459248	3.531477
H	-5.051378	-0.914497	-1.183192
H	-6.220255	0.348408	-0.711405
H	-6.367279	-1.301664	-0.030996
H	-1.698268	1.492299	-1.047118
H	-1.668942	3.934503	-1.404847
H	0.474855	5.198334	-1.009858
H	5.831368	1.313485	1.233906
H	5.346965	-1.147536	1.470212
H	3.079737	-1.973213	0.875469
H	2.465333	3.944359	-0.229868
H	4.030290	2.809701	0.429346
H	0.728255	-0.567960	2.942774
H	-1.038912	-1.629069	2.544306

I1

P	0.729403	-0.371652	-1.928323
Re	-0.251841	-0.833717	0.344441
C	-1.044051	-1.134495	2.100523
O	-1.501073	-1.334017	3.147365
N	-2.204702	-0.219008	-0.480061
C	-2.553179	1.086848	-0.329796
C	-3.802480	1.550205	-0.757915
C	-4.696589	0.669724	-1.357481
C	-4.322854	-0.663989	-1.522408
C	-3.073122	-1.065797	-1.068857
C	-1.530449	1.959088	0.279961
N	-0.352846	1.357812	0.606859
C	0.656004	2.108672	1.089544
C	0.518970	3.474961	1.321310
C	-0.695209	4.091312	1.032662
C	-1.725971	3.325712	0.493662
C	1.510857	-1.190510	1.062109
O	2.555656	-1.443407	1.498401
C	-0.401545	-2.701195	-0.093000
O	-0.522658	-3.822511	-0.383309
C	1.673269	1.175260	-2.305655
C	2.819722	1.555005	-1.744521
C	3.611533	0.979704	-0.718374
O	4.529115	0.064742	-1.196169
C	5.498600	-0.409706	-0.250747
O	3.625237	1.313279	0.482054
H	1.181903	1.755187	-3.087877
H	6.059203	0.421789	0.182063
H	6.166330	-1.057023	-0.818721
H	5.015419	-0.972061	0.551206
H	1.604742	1.612241	1.259633
H	1.361191	4.032110	1.710290
H	-0.837903	5.151404	1.208332
H	-5.665390	1.021140	-1.692177
H	-4.980560	-1.386807	-1.987762
H	-2.745828	-2.091954	-1.169682
H	-4.072037	2.589390	-0.628578
H	-2.667840	3.789710	0.234826
H	-0.261439	-0.398077	-2.934634
H	1.540835	-1.424603	-2.395541

TS1'

C	3.838019	1.731643	0.509185
C	2.574506	1.209239	0.209004
N	2.334050	-0.126457	0.293279
C	3.333006	-0.951315	0.667679
C	4.607857	-0.493370	0.973777
C	4.864028	0.875408	0.894105
C	1.430428	2.046530	-0.203246
N	0.260527	1.387336	-0.431235
C	-0.838913	2.094904	-0.759364
C	-0.810224	3.477131	-0.921001
C	0.389045	4.156952	-0.726999
C	1.517801	3.433356	-0.350555
Re	0.319667	-0.818957	-0.281161
C	-1.505711	-1.210861	-0.817521
O	-2.565797	-1.474051	-1.209252
P	-0.354532	-0.475431	2.115890
C	-1.886920	0.489652	2.471940
C	-2.846843	0.591091	1.588038
C	-3.849164	0.774898	0.689908
O	-3.922060	1.672542	-0.198206
C	0.615675	-2.689411	0.052458
O	0.834742	-3.809121	0.286373
C	0.883129	-1.015099	-2.134562
O	1.210387	-1.152509	-3.238825
O	-4.868291	-0.180704	0.805633
C	-5.728167	-0.297725	-0.331052
H	-1.896225	0.916658	3.481697
H	-6.214183	0.651937	-0.563943
H	-6.476595	-1.041801	-0.056682
H	-5.170566	-0.640299	-1.208310
H	-1.773307	1.557617	-0.873036

H	-1.722425	3.993531	-1.189523
H	0.447297	5.231562	-0.852695
H	5.845152	1.271133	1.126575
H	5.374357	-1.198963	1.267327
H	3.089380	-2.004409	0.715655
H	4.019463	2.795662	0.441826
H	2.454918	3.944604	-0.178900
H	0.650526	0.130911	2.907588
H	-0.484064	-1.668474	2.860315

I1′

P	0.573353	-0.536142	-1.867559
Re	-0.637611	-0.878684	0.308674
C	-1.232856	-2.610478	-0.276785
O	-1.626481	-3.635745	-0.665227
N	-0.167408	1.241523	0.715916
C	0.959566	1.655337	1.327520
C	1.199325	2.994720	1.617992
C	0.245149	3.943055	1.256917
C	-0.909153	3.522191	0.602141
C	-1.092813	2.162466	0.334766
C	-2.268845	1.632962	-0.383901
N	-2.296736	0.285876	-0.566377
C	-3.331890	-0.263572	-1.233087
C	-4.379761	0.490224	-1.746337
C	-4.363321	1.872460	-1.558925
C	-3.298492	2.445237	-0.871801
C	-1.605558	-1.078027	1.986280
O	-2.174702	-1.225693	2.986608
C	0.921885	-1.696961	1.121388
O	1.806061	-2.220633	1.660074
C	2.312783	-0.015211	-1.879658
C	2.998262	0.118524	-0.737625
C	4.356217	0.550451	-0.660001
O	5.252817	-0.496201	-0.640283
C	6.618830	-0.155570	-0.365723
O	4.749464	1.718246	-0.502971
H	2.705314	0.162648	-2.888588
H	7.007582	0.544147	-1.108569
H	7.169147	-1.094702	-0.409935
H	6.718333	0.290255	0.627267
H	1.688379	0.888811	1.549503
H	2.121454	3.275990	2.109345
H	0.399270	4.994763	1.469081
H	-5.163185	2.494314	-1.942065
H	-5.184666	-0.002800	-2.276167
H	-3.306302	-1.339123	-1.349837
H	-3.265775	3.515897	-0.722655
H	-1.655038	4.244996	0.301747
H	-0.100982	0.389815	-2.700455
H	0.502791	-1.640050	-2.747222

TS2*cco*

C	0.684016	2.192093	0.718529
N	-0.381523	1.438603	0.382105
C	-1.571243	2.049430	0.133138
C	-1.706338	3.437318	0.242671
C	-0.608706	4.208179	0.613922
C	0.609600	3.574380	0.851813
Re	-0.352436	-0.782177	0.253298
C	-0.579853	-2.658616	-0.026536
O	-0.769695	-3.796005	-0.222686
C	-2.683853	1.160100	-0.255493
N	-2.390588	-0.166536	-0.314613
C	-3.363528	-1.034564	-0.659821
C	-4.658760	-0.632248	-0.958539
C	-4.967810	0.727082	-0.903264
C	-3.970089	1.628588	-0.549436
P	0.407848	-0.457925	-2.110468
C	-0.856462	-0.999891	2.105532
O	-1.149636	-1.160395	3.221674
C	1.553894	-1.055761	0.789389
C	2.725030	0.172799	-0.911098
C	4.025586	0.644776	-0.551645
O	4.960053	-0.352676	-0.492935
C	6.248205	0.018254	0.026079
C	2.132013	0.119222	-2.102743
O	2.443804	-1.441526	1.445117
O	4.290640	1.798781	-0.184630
H	2.572065	0.409775	-3.060122
H	6.728455	0.756379	-0.619744
H	6.829890	-0.901310	0.044068
H	6.156381	0.429779	1.032700
H	-0.703969	5.283464	0.707693

H	-5.966903	1.080029	-1.127287
H	-5.401647	-1.372708	-1.226269
H	-3.078848	-2.078024	-0.685271
H	-4.191551	2.686042	-0.499778
H	-2.657470	3.912067	0.045437
H	1.495506	4.129475	1.132146
H	1.619756	1.664699	0.854762
H	-0.299977	0.476696	-2.902560
H	0.346507	-1.540987	-3.015867

P*cco*

C	0.477369	2.534145	0.395670
N	-0.543363	1.682318	0.164302
C	-1.748218	2.190146	-0.214065
C	-1.932121	3.568758	-0.376938
C	-0.876250	4.439904	-0.132951
C	0.352735	3.911947	0.263058
Re	-0.396138	-0.533478	0.371049
C	-0.488346	-2.428092	0.440479
O	-0.591849	-3.600579	0.473941
C	-2.825829	1.204951	-0.426078
N	-2.483026	-0.096403	-0.227038
C	-3.430666	-1.044301	-0.389546
C	-4.739343	-0.750005	-0.749372
C	-5.098031	0.584003	-0.952616
C	-4.129974	1.567384	-0.787567
P	0.479909	-0.574445	-1.935491
C	-0.702009	-0.470322	2.262153
O	-0.843364	-0.450328	3.423748
C	1.686548	-0.521166	0.768941
C	2.643983	-0.159013	-0.406258
C	4.022982	0.274010	-0.021173
O	4.961282	-0.647008	-0.276102
C	6.308442	-0.306165	0.125219
C	2.248182	-0.205312	-1.686167
O	2.294126	-0.736770	1.830667
O	4.253859	1.359712	0.484975
H	2.911097	-0.007935	-2.522308
H	6.911874	-1.177033	-0.118457
H	6.652694	0.570363	-0.424743
H	6.334859	-0.101676	1.195748
H	-1.010654	5.508367	-0.251986
H	-6.110898	0.853400	-1.228770
H	-5.457277	-1.552796	-0.859766
H	-3.111912	-2.063932	-0.215136
H	-4.386190	2.607595	-0.935134
H	-2.892578	3.958688	-0.684884
H	1.205574	4.547037	0.465581
H	1.418658	2.088867	0.691543
H	0.114695	0.372798	-2.918830
H	0.480307	-1.717722	-2.761173

P*ccoh*

C	-0.334588	2.660610	0.307368
N	-1.042604	1.533298	0.092690
C	-2.346918	1.634553	-0.278125
C	-2.954633	2.885641	-0.442902
C	-2.213418	4.043699	-0.237412
C	-0.877388	3.929419	0.150814
Re	-0.208971	-0.495285	0.357339
C	0.236768	-2.345818	0.475513
O	0.453209	-3.497920	0.533167
C	-3.053212	0.357234	-0.478662
N	-2.321865	-0.762614	-0.224906
C	-2.909595	-1.966586	-0.395465
C	-4.225482	-2.112828	-0.812763
C	-4.983171	-0.967742	-1.068633
C	-4.385701	0.276042	-0.903321
P	0.612031	-0.463663	-2.047271
C	2.327636	-0.447544	-1.745843
C	2.768934	-0.030408	-0.486713
C	4.173229	0.286262	-0.242030
O	4.994464	0.079041	-1.285018
C	6.391181	0.371852	-1.071007
C	-0.469197	-0.398510	2.243888
O	-0.578703	-0.358242	3.412055
C	1.778604	0.058176	0.585487
O	2.228725	0.552295	1.744517
O	4.611012	0.714804	0.841664
H	3.055499	-0.678231	-2.517199
H	6.529469	1.439617	-0.891423
H	6.890667	0.069587	-1.988434
H	6.770755	-0.193362	-0.219706
H	-2.672618	5.016253	-0.369906

H	-6.012828	-1.042039	-1.395912
H	-4.640996	-3.105847	-0.929516
H	-2.290167	-2.829020	-0.186327
H	-4.949329	1.177363	-1.103266
H	-3.994056	2.953262	-0.733500
H	-0.261959	4.801704	0.330076
H	0.692499	2.519677	0.617858
H	3.206945	0.743442	1.666840
H	0.511826	-1.489984	-3.017682

TS2*ccb*

P	0.294722	-0.633753	-1.991980
C	2.090257	-0.316686	-1.919641
C	2.714872	0.043707	-0.796570
C	4.119839	0.322181	-0.658831
O	4.845060	-0.777087	-0.287520
C	6.227846	-0.538077	0.030932
Re	-0.930335	-0.886445	0.173089
N	-2.081269	0.925023	-0.353859
C	-1.499253	2.120144	-0.065962
C	-2.193659	3.319032	-0.275752
C	-3.484574	3.290742	-0.788089
C	-4.064411	2.057967	-1.097112
C	-3.329532	0.903815	-0.866059
C	-0.127908	2.060337	0.475862
N	0.346005	0.805457	0.726394
C	1.660354	0.643345	1.085060
C	2.400829	1.781919	1.540100
C	1.893644	3.040836	1.342395
C	0.619896	3.196374	0.751387
C	-1.816225	-0.964248	1.907982
O	-2.344440	-1.035386	2.938247
C	0.272952	-2.299638	0.699946
O	1.009288	-3.135081	1.035889
C	-2.155600	-2.183887	-0.557769
O	-2.912695	-2.937606	-1.023840
O	4.629497	1.445054	-0.712241
H	-0.209986	0.382081	-2.835050
H	1.909743	-0.330167	1.478949
H	3.375916	1.627328	1.983748
H	2.455951	3.916538	1.646121
H	0.218843	4.182634	0.564936
H	-3.739221	-0.074833	-1.079523
H	-5.064928	1.985086	-1.503815
H	-4.028826	4.214676	-0.944905
H	-1.724949	4.261978	-0.029493
H	2.599664	-0.449947	-2.879202
H	6.632361	-1.505294	0.323718
H	6.762766	-0.150045	-0.837791
H	6.314821	0.176663	0.851312
H	0.209326	-1.706690	-2.900249

P*ccb*

Re	-0.958026	-0.890039	0.125644
P	0.163414	-0.367407	-2.030806
N	0.456189	0.700836	0.583303
N	-2.068237	0.989200	-0.185689
C	-1.673519	-1.054363	1.925731
O	-2.095811	-1.166537	3.001272
C	0.196725	-2.402126	0.435204
O	0.894654	-3.311931	0.642220
C	-2.313500	-2.070256	-0.577796
O	-3.147916	-2.750491	-1.029776
C	1.952236	-0.107372	-1.817618
H	2.571387	-0.270019	-2.695050
C	2.569513	0.173459	-0.656564
C	4.072431	0.138531	-0.707987
O	4.745154	0.428001	-1.683826
O	4.601245	-0.291254	0.449356
C	6.043164	-0.367438	0.499584
H	6.408602	-1.065790	-0.254103
H	6.278430	-0.722748	1.499448
H	6.473436	0.619368	0.325098
C	1.896657	0.455788	0.707691
H	2.032186	-0.463498	1.290677
C	2.578451	1.594803	1.448426
H	3.459649	1.368231	2.034476
C	2.089113	2.851178	1.335643
H	2.600603	3.682070	1.813261
C	0.839635	3.089867	0.672770
C	0.040658	1.991291	0.407078
C	-1.371607	2.143335	0.004970
C	-2.007585	3.384486	-0.163058
C	-3.347494	3.437778	-0.516554

H	-3.839957	4.394826	-0.646514
C	-4.054622	2.242408	-0.689830
H	-5.103802	2.233929	-0.954489
C	-3.376655	1.046459	-0.519769
H	-3.877235	0.095286	-0.647781
H	-1.447680	4.296905	-0.011095
H	0.472610	4.097037	0.540435
H	-0.272941	0.828622	-2.640448
H	0.149568	-1.209705	-3.159918

TS2*ins*

Re	0.215785	-0.763031	-0.489843
C	0.948436	-0.831648	-2.223801
O	1.388642	-0.886341	-3.308164
C	0.502109	-2.652080	-0.304988
O	0.724589	-3.790747	-0.188780
C	-1.560079	-1.090555	-1.174205
O	-2.602762	-1.275124	-1.655661
N	0.181222	1.463590	-0.514206
C	-0.867226	2.187563	-0.951829
C	-0.854974	3.576503	-0.995064
C	0.282754	4.251676	-0.557938
C	1.366524	3.512125	-0.095384
C	1.296094	2.115171	-0.088180
N	2.167725	-0.076480	0.349815
C	3.136148	-0.919187	0.761094
C	4.376895	-0.479918	1.202494
C	4.633340	0.892353	1.220827
C	3.637180	1.767600	0.804946
C	2.405624	1.259032	0.372521
P	-0.058506	-1.072715	2.516229
C	-1.684800	-0.285035	2.484397
C	-2.121715	-0.002018	1.253064
C	-3.378292	0.614552	0.907543
O	-3.539118	1.804916	0.611469
O	-4.395747	-0.289884	0.798561
C	-5.650019	0.218216	0.315177
H	-1.743412	1.633653	-1.254409
H	-1.728216	4.105225	-1.354001
H	0.325938	5.333696	-0.570473
H	2.256832	4.016452	0.254036
H	2.895840	-1.973815	0.729766
H	5.120033	-1.200399	1.519475
H	5.590861	1.274685	1.554183
H	3.817573	2.833905	0.810946
H	-2.227180	-0.085534	3.413022
H	-6.073986	0.935083	1.021553
H	-5.521860	0.703255	-0.654205
H	-6.299835	-0.650059	0.222899
H	0.671142	-0.374801	3.519041
H	-0.245455	-2.251754	3.290288

P*ins*

Re	0.183166	-0.394637	0.789317
C	-0.445284	-0.035265	2.603412
O	-0.804117	0.145548	3.696315
C	0.109500	-2.292168	1.102619
O	0.055560	-3.439921	1.294611
C	2.006818	-0.371825	1.395811
O	3.113063	-0.299310	1.758996
N	0.052153	1.738687	0.214169
C	1.047739	2.626693	0.391430
C	0.949220	3.956849	0.001610
C	-0.225327	4.388112	-0.610630
C	-1.262371	3.478201	-0.792212
C	-1.103129	2.154760	-0.368110
N	-1.866756	-0.093311	0.004626
C	-2.819542	-1.045290	0.006057
C	-4.102298	-0.825005	-0.482477
C	-4.410888	0.426486	-1.013109
C	-3.434222	1.416655	-1.009661
C	-2.168921	1.139648	-0.480177
P	-0.900958	-2.810978	-2.162132
C	0.493581	-1.619163	-2.293492
C	0.889383	-0.774330	-1.317501
C	2.090701	0.036206	-1.652455
O	2.095884	1.204004	-2.024620
O	3.237674	-0.653015	-1.434548
C	4.470767	0.080705	-1.561875
H	1.945832	2.251709	0.863225
H	1.780551	4.628328	0.172636
H	-0.338263	5.414734	-0.937158
H	-2.184750	3.797756	-1.257028
H	-2.530952	-2.006650	0.406284

H	-4.830483	-1.625888	-0.452761
H	-5.394474	0.631743	-1.417994
H	-3.656832	2.395787	-1.410390
H	1.040633	-1.642246	-3.238657
H	4.497823	0.630449	-2.503332
H	4.575170	0.775589	-0.725739
H	5.261346	-0.667313	-1.528805
H	-1.551386	-2.506345	-3.392019
H	-0.233912	-3.938932	-2.718097

Figure S4. PCM-B3LYP/6-31+G(d,p) (LANL2DZ for Re) optimized geometries in THF solution of the critical structures involved in the reaction between the complex [Re(PH_2)$(CO)_3$(bipy)] (bipy = 2,2'-bipyridine) and methyl propiolate (HMAD, HC≡CCO_2Me). Relevant distances are given in angstroms.

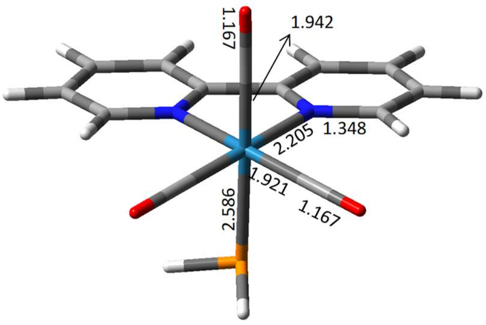

[Re(PH_2)$(CO)_3$(bipy)]

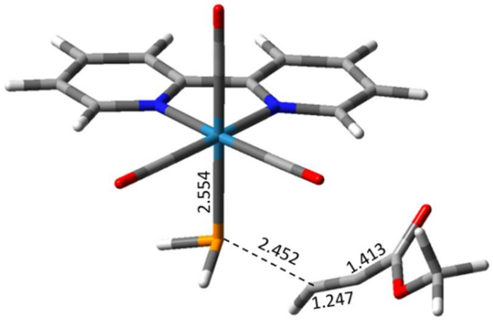

TS1

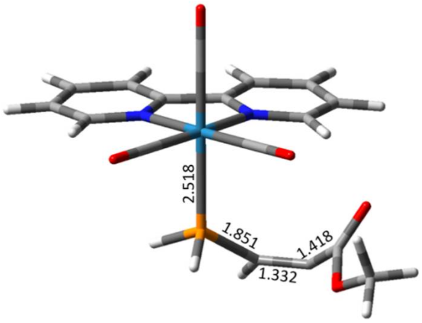

I1

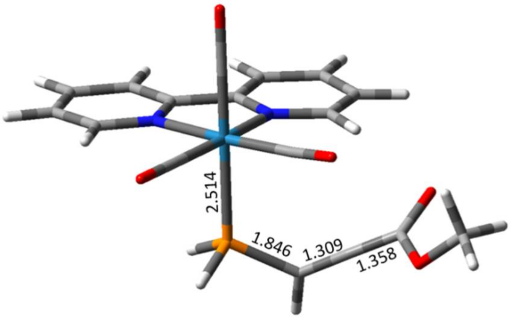

TS1'

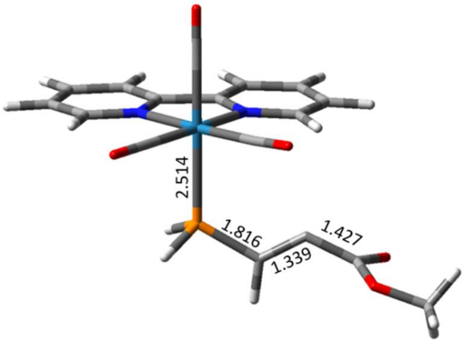

I1'

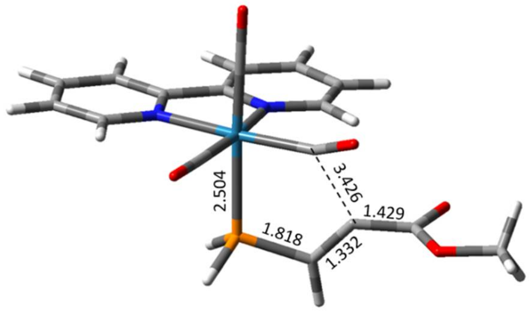

TS2*cco*

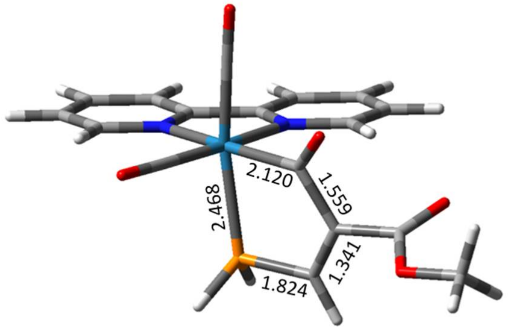

P*cco*

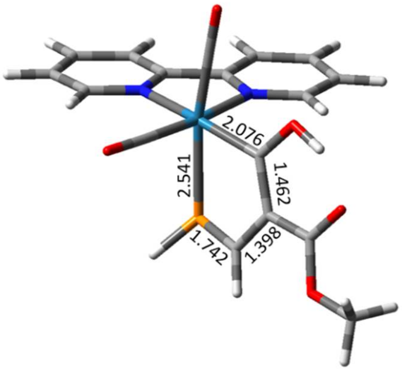

P*ccoh*

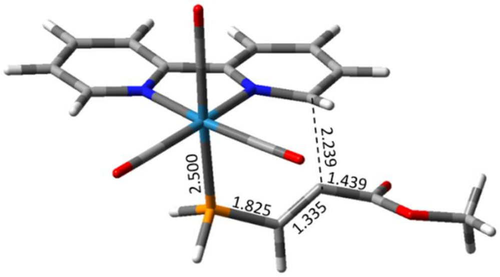

TS2*ccb*

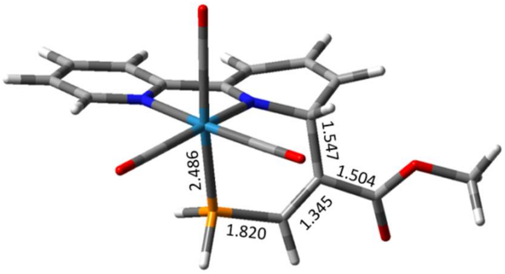

P*ccb*

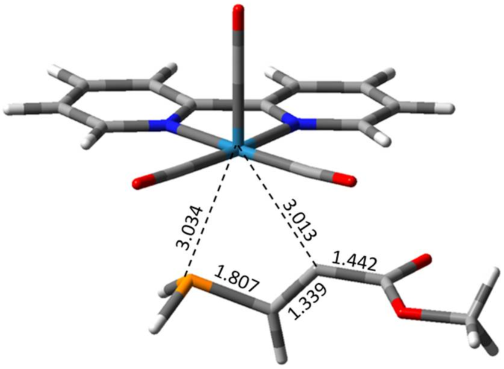

TS2*ins*

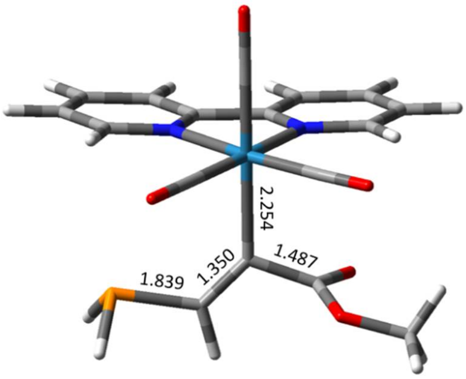

P*ins*

Table S13. PCM-B3LYP/6-31+G(d,p) (LANL2DZ for Re) energies without and with including thermal corrections (E and G, respectively), enthalpies (H), and entropies (S), and CPCM-DLPNO-CCSD(T)/def2-TZVPP//PCM-B3LYP/6-31+G(d,p) (LANL2DZ for Re) energies without and with including thermal corrections (E' and G', respectively) in THF solution of the critical structures involved in the reaction between the complex [Re(PHMe)$(CO)_3$(bipy)] (bipy = 2,2'-bipyridine) and methyl propiolate (HMAD, HC≡CCO_2Me). All the values are given in hartree, except entropies that are in cal/K mol.[a]

Species	E	H	S	G	E'	G'[b]
[Re(PHMe)$(CO)_3$(bipy)]	-1296.517425	-1296.261315	153.272	-1296.334139	-1293.575468	-1293.392182
HMAD	-305.215726	-305.136831	77.228	-305.173524	-304.738441	-304.696239
Reactants	-1601.733151	-1601.398146	230.500	-1601.507663	-1598.313909	-1598.088421
**TS1**	-1601.720346	-1601.384443	184.035	-1601.471884	-1598.287577	-1598.039115
**I1**	-1601.747738	-1601.409798	182.885	-1601.496693	-1598.338068	-1598.087023
**TS1'**	-1601.741702	-1601.405457	181.000	-1601.491456	-1598.328828	-1598.078582
**I1'**	-1601.747727	-1601.410023	186.469	-1601.498620	-1598.337212	-1598.088105
**TS2*cco***	-1601.743863	-1601.407800	183.923	-1601.495188	-1598.329014	-1598.080339
**P*cco***	-1601.768958	-1601.430119	180.257	-1601.515893	-1598.355597	-1598.102532
**P*ccoh***	-1601.778362	-1601.436889	178.895	-1601.521887	-1598.351793	-1598.095318
**TS2*ccb***	-1601.740752	-1601.404236	179.369	-1601.489460	-1598.330413	-1598.079121
**P*ccb***	-1601.769992	-1601.431016	175.632	-1601.514465	-1598.369896	-1598.114369
**TS2*ins***	-1601.719963	-1601.383697	180.347	-1601.469385	-1598.305310	-1598.054732
**P*ins***	-1601.772592	-1601.433876	183.334	-1601.520984	-1598.369194	-1598.117586

[a] Thermal magnitudes were computed in THF solution at 298.15 K and 1 atm. [b] For each species, G' was calculated as G' = G – E + E', in which G is the PCM-B3LYP/6-31+G(d,p) (LANL2DZ for Re) energy with including thermal corrections and E and E' are the PCM-B3LYP/6-31+G(d,p) (LANL2DZ for Re) and CPCM-DLPNO-CCSD(T)/def2-TZVPP//PCM-B3LYP/6-31+G(d,p) (LANL2DZ for Re) energies without including thermal corrections, respectively.

Table S14. PCM-B3LYP/6-31+G(d,p) (LANL2DZ for Re) relative energies without and with including thermal corrections (ΔE and ΔG, respectively), enthalpies (ΔH), and entropic contributions (TΔS), and CPCM-DLPNO-CCSD(T)/def2-TZVPP//PCM-B3LYP/6-31+G(d,p) (LANL2DZ for Re) relative energies without and with including thermal corrections (E' and G', respectively) in THF solution of the critical structures involved in the reaction between the complex [Re(PHMe)$(CO)_3$(bipy)] (bipy = 2,2'-bipyridine) and methyl propiolate (HMAD, HC≡CCO_2Me). All the values are given in kcal/mol.[a]

Species	ΔE	ΔH	TΔS	ΔG	ΔE'	ΔG'
[Re(PHMe)$(CO)_3$(bipy)] + HMAD	0.0	0.0	0.0	0.0	0.0	0.0
**TS1**	8.0	8.6	-13.9	22.5	16.5	30.9
**I1**	-9.2	-7.3	-14.2	6.9	-15.2	0.9
**TS1'**	-5.4	-4.6	-14.8	10.2	-9.4	6.2
**I1'**	-9.1	-7.5	-13.1	5.7	-14.6	0.2
**TS2*cco***	-6.7	-6.1	-13.9	7.8	-9.5	5.1
**P*cco***	-22.5	-20.1	-15.0	-5.2	-26.2	-8.9
**P*ccoh***	-28.4	-24.3	-15.4	-8.9	-23.8	-4.3
**TS2*ccb***	-4.8	-3.8	-15.2	11.4	-10.4	5.8
**P*ccb***	-23.1	-20.6	-16.4	-4.3	-35.1	-16.3
**TS2*ins***	8.3	9.1	-15.0	24.0	5.4	21.1
**P*ins***	-24.7	-22.4	-14.1	-8.4	-34.7	-18.3

[a] Thermal magnitudes were evaluated in THF solution at 298.15 K and 1 atm.

Table S15. PCM-B3LYP/6-31+G(d,p) (LANL2DZ for Re) optimized cartesian coordinates, in Å, for the critical structures involved in the reaction of the complex [Re(PHMe)(CO)$_3$(bipy)] (bipy = 2,2'-bipyridine) towards methyl propiolate (HMAD, HC≡CCO$_2$Me).

[Re(PHMe)(CO)$_3$(bipy)]

Re	-0.054487	0.088586	-0.039942
C	-0.003331	0.082409	1.904935
O	0.094731	0.087466	3.067683
C	0.699174	1.852553	-0.127887
O	1.128807	2.936133	-0.202692
C	1.702155	-0.667194	-0.203168
O	2.755916	-1.149829	-0.344529
P	-0.193685	0.002484	-2.616583
C	1.403366	0.728988	-3.297215
C	-3.090469	-0.316644	-0.230611
C	-2.605757	1.957847	-0.068237
C	-4.462633	-0.039160	-0.260579
C	-3.951724	2.298091	-0.100989
H	-1.839195	2.718092	0.007917
C	-4.900237	1.277946	-0.191504
H	-5.180623	-0.845612	-0.331843
H	-4.241791	3.339631	-0.053128
H	-5.959434	1.504226	-0.209943
C	-2.544471	-1.683064	-0.298468
C	-0.628151	-3.011582	-0.231404
C	-3.345720	-2.820027	-0.460096
C	-1.369863	-4.175403	-0.381871
H	0.449174	-3.041782	-0.140291
C	-2.757494	-4.078123	-0.503856
H	-4.417919	-2.718466	-0.555656
H	-0.863352	-5.132168	-0.406723
H	-3.367991	-4.963804	-0.629716
N	-1.192514	-1.786658	-0.188190
N	-2.173453	0.681474	-0.118518
H	0.152485	-1.371651	-2.793845
H	1.451581	0.591837	-4.381174
H	1.406250	1.803466	-3.094440
H	2.298842	0.293638	-2.846444

TS1

C	3.817203	1.745398	0.785245
C	2.594276	1.227940	0.340486
N	2.363761	-0.112034	0.333164
C	3.328630	-0.944684	0.774417
C	4.559784	-0.492546	1.229864
C	4.809489	0.880720	1.232405
C	1.488534	2.070271	-0.150428
N	0.381016	1.405895	-0.580480
C	-0.677379	2.116819	-1.016352
C	-0.674755	3.506289	-1.068876
C	0.461528	4.194633	-0.647244
C	1.550728	3.467414	-0.178548
Re	0.417782	-0.793615	-0.436080
P	-0.431055	-0.450561	1.951230
C	-1.677988	-1.780377	2.357102
C	0.641006	-2.650109	-0.003312
O	0.799232	-3.762060	0.313608
C	-1.379054	-1.146851	-1.033500
O	-2.468958	-1.352541	-1.392064
C	1.113899	-1.028372	-2.238781
O	1.512622	-1.223559	-3.315635
C	-2.080195	1.441662	2.313215
C	-3.039681	1.367525	1.530438
C	-3.873932	0.989220	0.447997
O	-3.843324	1.493612	-0.677098
O	-4.749212	0.007328	0.786950
C	-5.603188	-0.457247	-0.277082
H	-1.580894	1.920550	3.127406
H	-5.002118	-0.941077	-1.048530
H	-6.159421	0.375007	-0.710196
H	-6.280224	-1.170960	0.187683
H	-1.554640	1.557133	-1.313485
H	-1.555530	4.024763	-1.425201
H	0.498203	5.276608	-0.673509
H	5.759511	1.272317	1.576120
H	5.300244	-1.205609	1.569022
H	3.092630	-2.000053	0.747818
H	2.438000	3.980964	0.165554
H	3.994051	2.812391	0.777757
H	0.644693	-0.918387	2.767845
H	-2.601125	-1.557994	1.814614
H	-1.901410	-1.776110	3.426961
H	-1.329524	-2.775510	2.070555

I1

P	0.743193	-0.528168	-1.796035
Re	-0.286265	-0.738723	0.494794
C	-1.107355	-0.867053	2.260818
O	-1.573035	-0.971731	3.317890
N	-2.237828	-0.307604	-0.443577
C	-2.636404	0.992291	-0.467195
C	-3.880356	1.353214	-0.997804
C	-4.721794	0.373007	-1.511932
C	-4.301763	-0.956965	-1.487161
C	-3.057322	-1.253326	-0.946302
C	-1.673159	1.970502	0.074219
N	-0.485423	1.461809	0.504108
C	0.468891	2.304863	0.942826
C	0.268572	3.680963	1.012993
C	-0.956494	4.207840	0.614895
C	-1.933487	3.341892	0.129947
C	1.469173	-0.935449	1.285735
O	2.508696	-1.092018	1.778241
C	-0.360086	-2.647235	0.281483
O	-0.439634	-3.799238	0.123787
C	1.599273	1.017432	-2.312256
C	2.715654	1.502994	-1.767235
C	3.496634	1.117727	-0.651350
O	4.465834	0.175804	-0.954927
C	5.444467	-0.063691	0.067977
O	3.466127	1.633879	0.483726
H	1.119911	1.489048	-3.171982
H	5.972542	0.857322	0.326526
H	6.140934	-0.786243	-0.356935
H	4.974228	-0.471449	0.965216
H	1.427664	1.873204	1.204144
H	1.070440	4.313680	1.370972
H	-1.148639	5.273097	0.665123
H	-5.685170	0.643520	-1.926965
H	-4.919224	-1.755581	-1.877295
H	-2.695686	-2.272191	-0.906047
H	-4.184049	2.390309	-1.017520
H	-2.886361	3.733288	-0.199437
H	-0.247333	-0.682671	-2.792379
C	1.920274	-1.887518	-2.202465
H	2.831850	-1.717281	-1.625100
H	1.493138	-2.857221	-1.939130
H	2.168258	-1.865999	-3.264856

TS1'

C	1.406726	3.533697	0.057934
C	1.375840	2.139072	-0.017542
N	0.237725	1.479048	-0.368263
C	-0.880730	2.186248	-0.618707
C	-0.910657	3.576402	-0.556916
C	0.252292	4.262732	-0.217353
C	2.546957	1.292227	0.277897
C	3.798778	1.813390	0.623841
C	4.856575	0.950612	0.886438
C	4.640916	-0.425220	0.802177
C	3.376903	-0.882245	0.453337
N	2.347559	-0.050323	0.192792
Re	0.350868	-0.722749	-0.462378
C	0.933797	-0.720499	-2.323357
O	1.266776	-0.748899	-3.434220
C	-1.464052	-1.086802	-1.038738
O	-2.526880	-1.317153	-1.444297
P	-0.347140	-0.635721	1.953368
C	-1.889424	0.263605	2.415055
C	-2.877422	0.513171	1.590621

C	-3.818768	0.779917	0.648419
O	-4.805687	-0.213747	0.560352
C	-5.628975	-0.159320	-0.606521
C	0.694939	-2.609635	-0.318773
O	0.952043	-3.739279	-0.194191
O	-3.884006	1.800530	-0.098568
H	-1.896411	0.519101	3.482742
H	-6.072467	0.829769	-0.736563
H	-6.413114	-0.901244	-0.449092
H	-5.055572	-0.417886	-1.502733
H	-1.786620	1.638737	-0.845484
H	-1.844482	4.085985	-0.755658
H	0.264383	5.344054	-0.158743
H	5.830106	1.345132	1.151065
H	5.432276	-1.136938	0.998446
H	3.167904	-1.940790	0.369261
H	3.943480	2.883417	0.683421
H	2.318047	4.044368	0.338546
H	0.643028	0.000567	2.742199
C	-0.461107	-2.264667	2.815031
H	-0.677929	-2.121550	3.875609
H	-1.266335	-2.843561	2.359017
H	0.475214	-2.815949	2.704398

I1′

P	-0.032278	-0.019349	-0.059972
C	-0.013034	-0.001186	1.784670
Re	2.218138	0.011488	-1.188235
C	3.098691	0.126859	0.516234
O	3.620615	0.244889	1.551802
N	1.114652	0.219313	-3.090646
C	0.759528	-0.822029	-3.867858
C	0.127473	-0.651860	-5.095707
C	-0.166575	0.638923	-5.527983
C	0.179000	1.716978	-4.717896
C	0.823049	1.482803	-3.499362
C	1.236664	2.566540	-2.585953
N	1.896443	2.177897	-1.462431
C	2.318851	3.117978	-0.592816
C	2.106728	4.475756	-0.794055
C	1.425516	4.883759	-1.940812
C	0.985948	3.919688	-2.841334
C	3.933090	0.085717	-2.113726
O	4.962902	0.116258	-2.647997
C	2.323687	-1.923654	-1.110672
O	2.454261	-3.075726	-1.062168
C	-1.220229	-1.306777	-0.527704
C	-0.936599	-2.238176	-1.448080
C	-1.849060	-3.252382	-1.867057
O	-1.744518	-4.413733	-1.129023
C	-2.494250	-5.535492	-1.615156
O	-2.601705	-3.204316	-2.854548
H	-2.169582	-1.223983	0.021843
H	-3.563365	-5.313799	-1.637428
H	-2.292863	-6.347758	-0.917828
H	-2.171122	-5.812531	-2.621032
H	0.969753	-1.805229	-3.472291
H	-0.132266	-1.521449	-5.685035
H	-0.660823	0.807870	-6.477477
H	1.238728	5.933588	-2.131895
H	2.471409	5.188398	-0.065625
H	2.842998	2.756427	0.281831
H	0.453608	4.215834	-3.734354
H	-0.044280	2.726256	-5.035274
H	-0.716584	1.199159	-0.299219
H	0.520255	-0.882373	2.145451
H	-1.034906	-0.016719	2.170506
H	0.497134	0.894042	2.146434

TS2*cco*

C	0.983914	2.163340	0.484906
N	-0.171946	1.512815	0.244537
C	-1.270874	2.228874	-0.115329
C	-1.223209	3.623399	-0.216113
C	-0.033056	4.291781	0.052863
C	1.091123	3.547820	0.408164
Re	-0.439927	-0.682793	0.463944
C	-0.913781	-2.534573	0.482023
O	-1.248364	-3.655456	0.467961
C	-2.492980	1.445802	-0.383332
N	-2.382128	0.099419	-0.226841
C	-3.466668	-0.671810	-0.448115
C	-4.695503	-0.150032	-0.831094
C	-4.815854	1.229692	-1.001311
C	-3.702422	2.031039	-0.776093
P	0.344126	-0.838299	-1.913531
C	0.089488	-2.407229	-2.849461
C	-0.974000	-0.527280	2.316902
O	-1.287827	-0.460848	3.436942
C	1.402161	-1.123295	1.085545
C	2.754371	-0.341745	-0.803747
C	4.139346	-0.122441	-0.537692
O	4.411549	1.192975	-0.229861
C	5.767250	1.481697	0.146188
C	2.117372	-0.454326	-1.969160
O	2.241676	-1.504805	1.805044
O	5.012387	-0.993813	-0.438710
H	2.567587	-0.341790	-2.961353
H	6.054121	0.920020	1.037797
H	5.792162	2.551661	0.349206
H	6.455858	1.236697	-0.665377
H	0.013513	5.371629	-0.017763
H	-5.757092	1.674012	-1.301455
H	-5.532895	-0.817082	-0.990703
H	-3.331039	-1.735525	-0.300080
H	-3.771648	3.103111	-0.901690
H	-2.105711	4.180365	-0.499615
H	2.042536	4.019611	0.617356
H	1.841310	1.544339	0.718351
H	-0.246771	0.081564	-2.812931
H	0.487311	-3.235225	-2.260403
H	0.603573	-2.369274	-3.812591
H	-0.977135	-2.574490	-3.017278

P*cco*

C	-4.138190	1.588569	-0.744131
C	-2.830380	1.219127	-0.403080
N	-2.484226	-0.086580	-0.240625
C	-3.433499	-1.030479	-0.418474
C	-4.746735	-0.728969	-0.753784
C	-5.108469	0.609424	-0.922584
C	-1.747391	2.198817	-0.190775
N	-0.534438	1.681790	0.148003
C	0.497176	2.527401	0.351461
C	0.378227	3.906026	0.222247
C	-0.861738	4.444197	-0.121455
C	-1.932689	3.580674	-0.322690
Re	-0.397617	-0.537536	0.340922
C	1.679618	-0.517310	0.762116
O	2.273163	-0.722203	1.834296
C	-0.482492	-2.431987	0.369655
O	-0.571636	-3.607189	0.364091
P	0.499745	-0.612588	-1.958071
C	2.263615	-0.236293	-1.687343
C	2.649121	-0.164822	-0.404843
C	4.017032	0.286541	-0.007124
O	4.216348	1.355629	0.545986
C	-0.730637	-0.514694	2.231257
O	-0.890454	-0.533592	3.390976
O	4.984683	-0.591235	-0.306542
C	6.319806	-0.227522	0.117723
H	2.939397	-0.056640	-2.518793
H	6.955187	-1.056111	-0.185144
H	6.629700	0.698153	-0.368584
H	6.340597	-0.097676	1.200445
H	-0.994343	5.514472	-0.227571
H	-6.123814	0.884930	-1.182942
H	-5.463590	-1.530047	-0.879453
H	-3.111019	-2.053161	-0.273264
H	-4.396174	2.631073	-0.870116
H	-2.904002	3.978651	-0.582280
H	1.243558	4.533433	0.394653
H	1.445146	2.079707	0.621605
H	0.164198	0.354886	-2.934239
C	0.460558	-2.147408	-2.975794
H	1.162759	-2.083835	-3.808122
H	0.716385	-2.996074	-2.339612
H	-0.549624	-2.291603	-3.364429

P*ccoh*

C	-4.337374	0.284027	-0.976367
C	-3.015283	0.355242	-0.518516
N	-2.304748	-0.769649	-0.227487
C	-2.907817	-1.968312	-0.385931

C	-4.215160	-2.104199	-0.832710
C	-4.947770	-0.954004	-1.135891
C	-2.306636	1.627906	-0.300327
N	-1.014309	1.518675	0.107793
C	-0.316131	2.641236	0.374129
C	-0.861626	3.912110	0.244135
C	-2.183423	4.035229	-0.188530
C	-2.909066	2.881900	-0.462422
Re	-0.200859	-0.519416	0.381382
C	1.794882	0.048830	0.596377
O	2.229452	0.602104	1.739035
C	0.233958	-2.367467	0.535228
O	0.448167	-3.519975	0.616106
P	0.644424	-0.605844	-2.005235
C	2.339278	-0.547538	-1.715441
C	2.778966	-0.063152	-0.464841
C	4.165128	0.321415	-0.249417
O	4.605894	0.795820	0.816493
C	-0.463037	-0.392187	2.265927
O	-0.569657	-0.331343	3.433719
O	4.980240	0.126683	-1.304400
C	6.364913	0.479862	-1.119998
H	3.065849	-0.808635	-2.479353
H	6.459420	1.545007	-0.904723
H	6.851945	0.235344	-2.061364
H	6.799475	-0.095350	-0.301400
H	-2.642904	5.009628	-0.303604
H	-5.969556	-1.020014	-1.488720
H	-4.643641	-3.093433	-0.935108
H	-2.307215	-2.833772	-0.138004
H	-4.884052	1.189188	-1.203707
H	-3.937646	2.956116	-0.787548
H	-0.259482	4.780243	0.478314
H	0.700545	2.494512	0.714536
H	3.202890	0.810686	1.651749
C	0.381685	-1.874599	-3.336408
H	1.232937	-1.861670	-4.021510
H	0.284430	-2.876584	-2.912031
H	-0.525466	-1.643928	-3.896643

TS2*ccb*

C	-0.181658	-2.379313	2.770647
P	-0.279834	-0.829850	1.782965
C	-2.061672	-0.451551	1.739747
C	-2.713007	-0.017918	0.657451
C	-4.116154	0.291160	0.586204
O	-4.877772	-0.779480	0.200333
C	-6.270933	-0.509888	-0.034718
Re	0.975915	-0.771281	-0.379467
N	2.029244	0.999293	0.412792
C	1.402936	2.198263	0.266667
C	2.020320	3.379920	0.697193
C	3.280251	3.330920	1.279985
C	3.912489	2.094106	1.425040
C	3.252985	0.957420	0.979856
C	0.063717	2.158771	-0.352699
N	-0.353926	0.925335	-0.758526
C	-1.650628	0.761009	-1.170246
C	-2.413733	1.913489	-1.539630
C	-1.952167	3.162090	-1.206394
C	-0.712814	3.293724	-0.542032
C	1.903250	-0.593683	-2.086850
O	2.459378	-0.511993	-3.101845
C	-0.164584	-2.151099	-1.094051
O	-0.873981	-2.964512	-1.529677
C	2.246550	-2.099369	0.202366
O	3.033450	-2.873353	0.579618
O	-4.603276	1.418887	0.712718
H	0.232425	0.102996	2.713887
H	-1.864803	-0.181286	-1.650389
H	-3.367512	1.773616	-2.031878
H	-2.529845	4.047203	-1.448059
H	-0.358127	4.268634	-0.237950
H	3.703036	-0.023034	1.069159
H	4.894488	2.004672	1.871973
H	3.763130	4.242319	1.612199
H	1.514943	4.328134	0.575252
H	-2.554351	-0.631767	2.702859
H	-6.703756	-1.458212	-0.348385
H	-6.755848	-0.156065	0.877270
H	-6.391781	0.240898	-0.817896
H	-0.583411	-3.206256	2.182178
H	-0.757397	-2.281523	3.693827
H	0.862160	-2.593594	3.011220

P*ccb*

Re	-0.039548	-0.051280	-0.029459
P	-0.063617	0.024685	2.459061
N	2.058616	0.029560	0.531509
N	0.580601	-2.154264	0.229148
C	0.284554	-0.148527	-1.947786
O	0.475611	-0.194142	-3.092080
C	-0.371581	1.841393	-0.163112
O	-0.559171	2.989405	-0.242816
C	-1.920983	-0.407227	-0.264969
O	-3.055081	-0.662996	-0.381140
C	1.216254	1.146050	3.109458
H	1.051161	1.501602	4.124952
C	2.280031	1.626476	2.442546
C	3.072689	2.675190	3.169775
O	3.247988	2.705851	4.377002
O	3.545407	3.613731	2.332826
C	4.315508	4.675437	2.938524
H	3.690537	5.246343	3.626381
H	4.647947	5.299040	2.112319
H	5.165773	4.257092	3.478359
C	2.701756	1.260412	0.998451
H	2.335375	2.088220	0.379907
C	4.210498	1.170904	0.840335
H	4.761719	2.081227	0.642539
C	4.820864	-0.030042	0.978022
H	5.903586	-0.103078	0.928569
C	4.045817	-1.231132	1.094965
C	2.699555	-1.150774	0.779629
C	1.876301	-2.361030	0.591532
C	2.360374	-3.668307	0.763558
C	1.515937	-4.752594	0.577537
H	1.884778	-5.762404	0.715020
C	0.185550	-4.523044	0.208157
H	-0.509708	-5.336796	0.048778
C	-0.235220	-3.214301	0.036945
H	-1.251710	-2.985296	-0.257269
H	3.394100	-3.821215	1.040812
H	4.527876	-2.183026	1.265193
C	-1.531103	0.461840	3.476060
H	0.314217	-1.213358	3.024790
H	-1.906099	1.437621	3.162825
H	-1.272730	0.494925	4.536500
H	-2.314556	-0.280622	3.316493

TS2*ins*

Re	0.000000	0.000000	0.000000
C	0.000000	0.000000	1.884281
O	0.020506	0.000000	3.056490
C	-0.045162	-1.917491	0.001205
O	-0.121258	-3.081429	0.024959
C	1.926434	-0.034048	-0.052950
O	3.089662	-0.034258	-0.033213
N	-0.308180	2.214054	0.000019
C	0.703673	3.100445	0.053425
C	0.496844	4.472062	0.141520
C	-0.810509	4.953523	0.161150
C	-1.861872	4.044729	0.096606
C	-1.586235	2.675156	0.023720
N	-2.208936	0.359110	-0.055945
C	-3.120963	-0.634096	-0.070726
C	-4.488613	-0.400840	-0.034087
C	-4.942184	0.918900	0.012707
C	-4.009309	1.948675	0.022249
C	-2.641447	1.644905	-0.010485
P	-0.878878	-0.587308	-2.848409
C	0.425317	0.500488	-3.462013
C	1.231113	0.978637	-2.506758
C	2.371644	1.841033	-2.698606
O	2.390872	3.066302	-2.530925
O	3.516499	1.142144	-2.963485
C	4.732783	1.907416	-3.001762
H	1.702667	2.691441	0.013583
H	1.349023	5.137567	0.184303
H	-1.010768	6.016008	0.223849
H	-2.884033	4.397005	0.111348
H	-2.726797	-1.641389	-0.109698
H	-5.175798	-1.237133	-0.042424
H	-6.002220	1.141661	0.043685
H	-4.340856	2.977555	0.057104
H	0.527178	0.703528	-4.533289
H	4.708650	2.633963	-3.816481

H	4.885873	2.433427	-2.057741
H	5.528627	1.183139	-3.165853
H	-2.095301	-0.127208	-3.366699
C	-0.705155	-2.126503	-3.896071
H	0.184595	-2.650420	-3.615433
H	-1.554572	-2.759177	-3.744039
H	-0.646083	-1.850628	-4.928207

Pins

C	-2.171179	1.174046	-0.457208
N	-1.875914	-0.082045	-0.030975
C	-2.833749	-1.027542	-0.074979
C	-4.119365	-0.773738	-0.539393
C	-4.429709	0.509655	-0.984891
C	-3.444069	1.490719	-0.944508
Re	0.152004	-0.414268	0.797481
C	0.884361	-0.801140	-1.293977
C	2.045816	0.047923	-1.672856
O	3.230293	-0.571657	-1.443253
C	4.415639	0.204538	-1.704867
C	-1.085981	2.167337	-0.336130
N	0.065368	1.721302	0.230632
C	1.082842	2.585379	0.402725
C	1.007757	3.921592	0.029304
C	-0.166833	4.386482	-0.558034
C	-1.222481	3.499095	-0.741376
C	-0.531291	-0.039298	2.587432
O	-0.935057	0.152918	3.662875
C	0.061828	-2.309279	1.131253
O	0.017040	-3.451948	1.353713
C	1.954588	-0.416033	1.459654
O	3.046074	-0.370062	1.869408
C	0.503086	-1.675569	-2.250343
P	-0.831717	-2.931916	-2.130704
O	1.992794	1.194995	-2.101605
H	1.979371	2.185464	0.856794
H	1.856230	4.573313	0.195985
H	-0.263191	5.420439	-0.867320
H	-2.142406	3.840955	-1.195166
H	-2.546059	-2.010459	0.268793
H	-4.851442	-1.570829	-0.548757
H	-5.419772	0.745639	-1.356305
H	-3.667602	2.494106	-1.279629
H	1.050350	-1.706070	-3.199338
H	4.463592	0.484104	-2.758220
H	4.421522	1.106323	-1.091159
H	5.251118	-0.441182	-1.442296
H	-1.526712	-2.573407	-3.322598
C	0.071908	-4.395564	-2.861426
H	-0.650385	-5.162040	-3.153709
H	0.669433	-4.116110	-3.733079
H	0.731621	-4.820665	-2.101736

Figure S5. PCM-B3LYP/6-31+G(d,p) (LANL2DZ for Re) optimized geometries in THF solution of the critical structures involved in the reaction between the complex [Re(PHMe)(CO)$_3$(bipy)] (bipy = 2,2'-bipyridine) and methyl propiolate (HMAD, HC≡CCO$_2$Me). Relevant distances are given in angstroms.

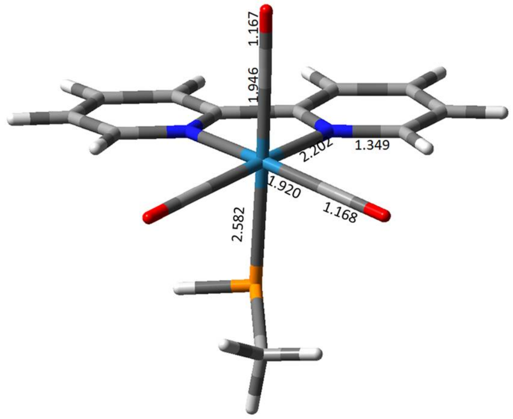

[Re(PHMe)(CO)$_3$(bipy)]

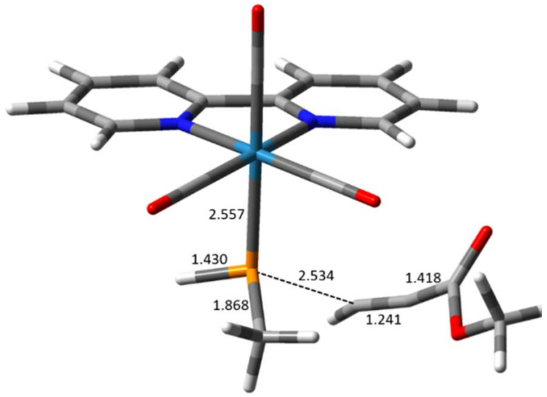

TS1

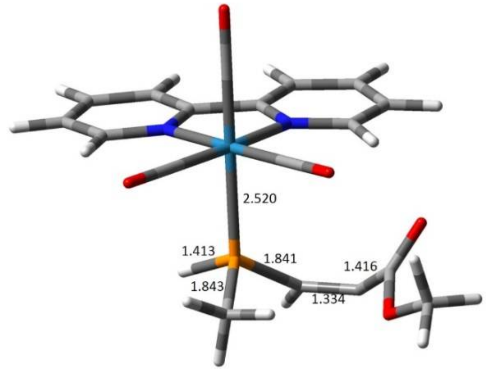

I1

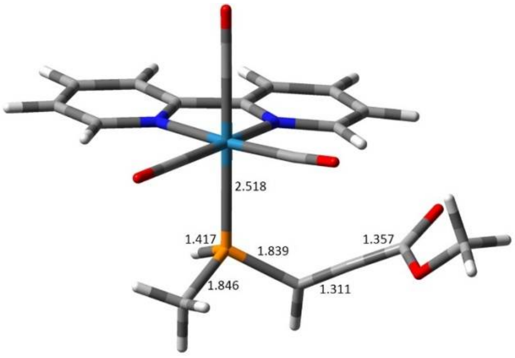

TS1'

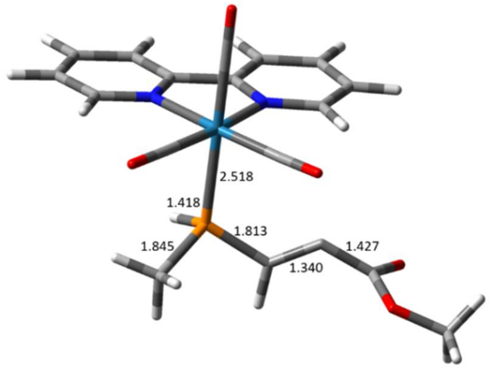

I1'

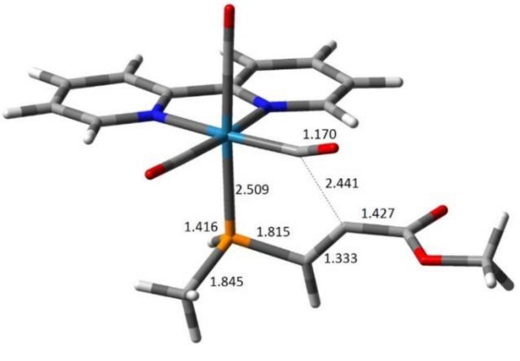

TS2*cco*

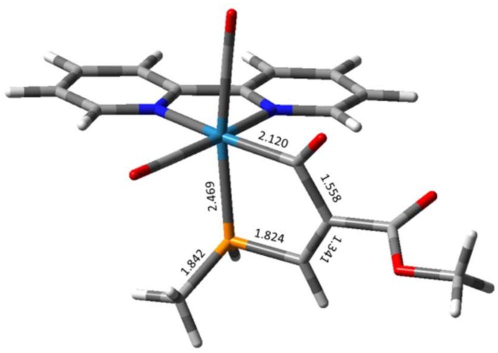

P*cco*

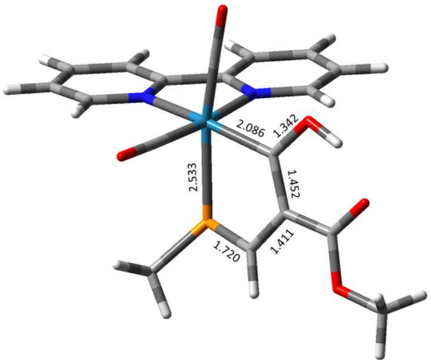

P*ccoh*

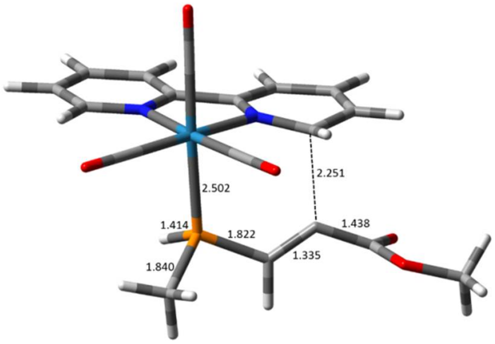

TS2*ccb*

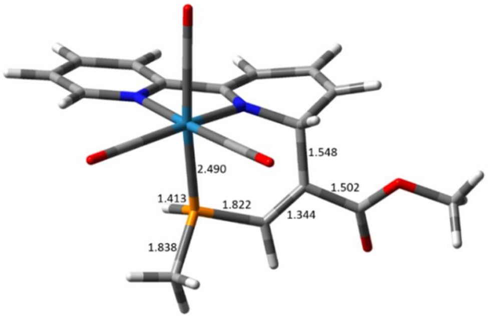

P*ccb*

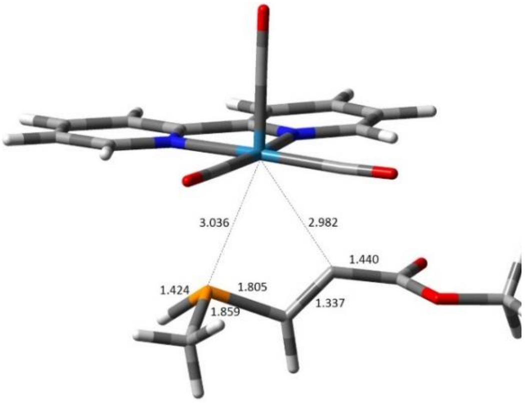

TS2*ins*

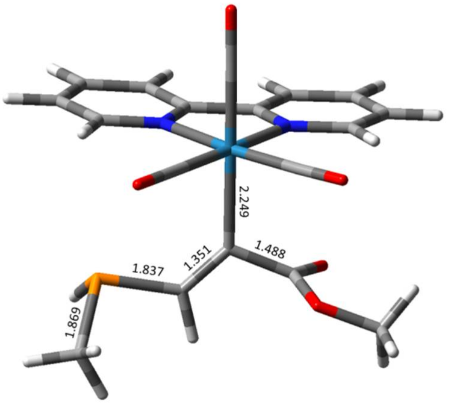

P*ins*

Table S16. PCM-B3LYP/6-31+G(d,p) (LANL2DZ for Re) energies without and with including thermal corrections (E and G, respectively), enthalpies (H), and entropies (S), and CPCM-DLPNO-CCSD(T)/def2-TZVPP//PCM-B3LYP/6-31+G(d,p) (LANL2DZ for Re) energies without and with including thermal corrections (E' and G', respectively) in THF solution of the critical structures involved in the reaction between the complex [Re(PMe_2)$(CO)_3$(bipy)] (bipy = 2,2'-bipyridine) and methyl propiolate (HMAD, HC≡CCO_2Me). All the values are given in hartree, except entropies that are in cal/K mol.[a]

Species	E	H	S	G	E'	G'[b]
[Re(PMe_2)$(CO)_3$(bipy)]	-1335.834013	-1335.546852	157.866	-1335.621859	-1332.820218	-1332.608064
HMAD	-305.215726	-305.136831	77.228	-305.173524	-304.738441	-304.696239
Reactants	-1641.049739	-1640.683683	235.094	-1640.795383	-1637.558659	-1637.304303
**TS1**	-1641.039020	-1640.672437	191.000	-1640.763188	-1637.552485	-1637.276653
**I1**	-1641.072772	-1640.704034	186.260	-1640.792532	-1637.593244	-1637.313004
**TS1'**	-1641.066478	-1640.699790	186.033	-1640.788180	-1637.584741	-1637.306443
**I1'**	-1641.072294	-1640.703936	191.999	-1640.795161	-1637.592468	-1637.315335
**TS2*cco***	-1641.068865	-1640.702165	186.262	-1640.790664	-1637.584592	-1637.306391
**P*cco***	-1641.094019	-1640.724653	190.147	-1640.814999	-1637.610816	-1637.331796
**TS2*ccb***	-1641.066111	-1640.699251	186.349	-1640.787792	-1637.586703	-1637.308384
**P*ccb***	-1641.095369	-1640.726098	183.659	-1640.813361	-1637.626942	-1637.344934
**TS2*ins***	-1641.042884	-1640.676107	187.779	-1640.765326	-1637.560470	-1637.282912
**P*ins***	-1641.093855	-1640.725559	191.222	-1640.816415	-1637.620091	-1637.342651

[a] Thermal magnitudes were computed in THF solution at 298.15 K and 1 atm. [b] For each species, G' was calculated as G' = G – E + E', in which G is the PCM-B3LYP/6-31+G(d,p) (LANL2DZ for Re) energy with including thermal corrections and E and E' are the PCM-B3LYP/6-31+G(d,p) (LANL2DZ for Re) and CPCM-DLPNO-CCSD(T)/def2-TZVPP//PCM-B3LYP/6-31+G(d,p) (LANL2DZ for Re) energies without including thermal corrections, respectively.

Table S17. PCM-B3LYP/6-31+G(d,p) (LANL2DZ for Re) relative energies without and with including thermal corrections (ΔE and ΔG, respectively), enthalpies (ΔH), and entropic contributions (TΔS), and CPCM-DLPNO-CCSD(T)/def2-TZVPP//PCM-B3LYP/6-31+G(d,p) (LANL2DZ for Re) relative energies without and with including thermal corrections (E' and G', respectively) in THF solution of the critical structures involved in the reaction between the complex [Re(PMe_2)$(CO)_3$(bipy)] (bipy = 2,2'-bipyridine) and methyl propiolate (HMAD, HC≡CCO_2Me). All the values are given in kcal/mol.[a]

Species	ΔE	ΔH	TΔS	ΔG	ΔE'	ΔG'
[Re(PMe_2)$(CO)_3$(bipy)] + HMAD	0.0	0.0	0.0	0.0	0.0	0.0
**TS1**	6.7	7.1	-13.1	20.2	3.9	17.4
**I1**	-14.5	-12.8	-14.6	1.8	-21.7	-5.5
**TS1'**	-10.5	-10.1	-14.6	4.5	-16.4	-1.3
**I1'**	-14.2	-12.7	-12.8	0.1	-21.2	-6.9
**TS2*cco***	-12.0	-11.6	-14.6	3.0	-16.3	-1.3
**P*cco***	-27.8	-25.7	-13.4	-12.3	-32.7	-17.3
**TS2*ccb***	-10.3	-9.8	-14.5	4.8	-17.6	-2.6
**P*ccb***	-28.6	-26.6	-15.3	-11.3	-42.8	-25.5
**TS2*ins***	4.3	4.8	-14.1	18.9	-1.1	13.4
**P*ins***	-27.7	-26.3	-13.1	-13.2	-38.5	-24.1

[a] Thermal magnitudes were evaluated in THF solution at 298.15 K and 1 atm.

Table S18. PCM-B3LYP/6-31+G(d,p) (LANL2DZ for Re) optimized cartesian coordinates, in Å, for the critical structures involved in the reaction of the complex [Re(PMe_2)$(CO)_3$(bipy)] (bipy = 2,2'-bipyridine) towards methyl propiolate (HMAD, HC≡CCO_2Me).

[Re(PMe_2)$(CO)_3$(bipy)]

Re	0.733304	-0.003278	-0.358621
C	0.677571	-0.111129	-2.299871
O	0.720815	-0.143847	-3.465832
C	2.065197	-1.380886	-0.239637
O	2.848802	-2.241605	-0.140124
C	2.106724	1.335190	-0.340063
O	2.917173	2.173044	-0.264428
P	0.771846	0.193031	2.235651
C	-2.212915	-0.683808	0.135592
C	-0.948360	-2.641802	0.059826
C	-3.371324	-1.423651	0.404989
C	-2.058153	-3.428657	0.337192
H	0.028749	-3.083776	-0.084815
C	-3.296572	-2.806937	0.510125
H	-4.320856	-0.922603	0.536333
H	-1.946872	-4.503062	0.413174
H	-4.185221	-3.387493	0.726621
C	-2.203984	0.783479	0.013490
C	-0.918237	2.687542	-0.400814
C	-3.354068	1.572644	0.144099
C	-2.023421	3.519692	-0.289396
H	0.063523	3.089067	-0.613227
C	-3.267554	2.950959	-0.007680
H	-4.307595	1.111290	0.361645
H	-1.902752	4.588165	-0.418355
H	-4.151539	3.568436	0.092375
N	-0.993395	1.347499	-0.252288
N	-1.011790	-1.297994	-0.042423
C	2.558498	-0.033353	2.759660
C	0.654098	2.025145	2.628777
H	3.222550	0.690487	2.274032
H	2.642957	0.095328	3.844118
H	2.899523	-1.040358	2.507174
H	1.422291	2.614899	2.115670
H	-0.329520	2.413827	2.354554
H	0.781387	2.165604	3.707387

I1

C	1.469054	3.547798	0.095948
C	1.422657	2.153147	-0.008882
N	0.307101	1.517703	-0.462462
C	-0.778109	2.252820	-0.776684
C	-0.792597	3.640226	-0.691587
C	0.354415	4.301928	-0.254949
C	2.557991	1.283278	0.342523
C	3.780141	1.770253	0.824034
C	4.808538	0.883479	1.119677
C	4.597039	-0.482882	0.923599
C	3.363989	-0.904937	0.446844
N	2.359151	-0.050524	0.159972
Re	0.399431	-0.675263	-0.615933
C	1.029770	-0.665496	-2.457994
O	1.385183	-0.739090	-3.565635
P	-0.408652	-0.730684	1.830433
C	0.675865	-2.562671	-0.410810
O	0.870121	-3.696038	-0.207276
C	-1.415152	-0.982827	-1.183882
O	-2.518812	-1.156044	-1.517723
C	-2.121563	1.136781	2.452441
C	-3.064958	1.152783	1.652540
C	-3.915190	0.923499	0.540173
O	-4.762293	-0.117317	0.742960
C	-5.646340	-0.424830	-0.353516
O	-3.915362	1.588810	-0.497716
H	-1.609777	1.481872	3.323661
H	-5.066640	-0.775137	-1.208826
H	-6.223958	0.455885	-0.636491
H	-6.300760	-1.211534	0.016443
H	-1.662241	1.715656	-1.093395
H	-1.693150	4.177425	-0.959882
H	0.380668	5.382214	-0.180684
H	5.757436	1.251576	1.491336
H	5.367518	-1.212920	1.135778
H	3.157593	-1.954251	0.280264
H	2.366417	4.038947	0.446761
H	3.925786	2.832599	0.968150
C	0.906198	-1.578396	2.863331
C	-1.716573	-2.056471	1.951752
H	-1.971035	-2.225209	3.002955
H	-2.619010	-1.735647	1.426186
H	-1.367365	-3.000266	1.520866
H	0.559296	-1.663902	3.897464
H	1.111340	-2.586244	2.485361
H	1.834815	-1.003788	2.855717

TS1

P	0.739348	-0.563294	-1.823604
Re	-0.324094	-0.732458	0.468646
C	-1.136347	-0.849392	2.240037
O	-1.596884	-0.951703	3.300056
N	-2.288762	-0.283610	-0.436453
C	-2.671809	1.021316	-0.456110
C	-3.915159	1.398801	-0.975795
C	-4.783916	0.428762	-1.463201
C	-4.383947	-0.907522	-1.434887
C	-3.131861	-1.218465	-0.920771
C	-1.695076	1.987989	0.080432
N	-0.497844	1.468622	0.468534
C	0.473957	2.301516	0.887611
C	0.276072	3.675716	0.996058
C	-0.964121	4.210904	0.659295
C	-1.956779	3.356137	0.185933
C	1.437739	-0.930801	1.242592
O	2.484547	-1.084439	1.721167
C	-0.417163	-2.640150	0.262028
O	-0.509942	-3.791283	0.101327
C	1.612632	0.984342	-2.309703
C	2.724645	1.489084	-1.772928
C	3.521898	1.119590	-0.664872
O	4.512543	0.202461	-0.978237
C	5.474982	-0.049137	0.056213
O	3.485156	1.625676	0.475198
H	1.126843	1.451917	-3.171337
H	6.032714	0.858690	0.300881
H	6.151243	-0.801060	-0.350250
H	4.985488	-0.424133	0.957045
H	1.439786	1.862283	1.110307
H	1.090109	4.301729	1.337874
H	-1.156517	5.273310	0.750895
H	-5.749990	0.711889	-1.863029
H	-5.021460	-1.699851	-1.805658
H	-2.783807	-2.242046	-0.884708
H	-4.197988	2.441956	-1.003395
H	-2.922819	3.752892	-0.096102
C	1.979050	-1.899790	-2.090038
C	-0.452689	-0.817033	-3.205182
H	2.845118	-1.701084	-1.454253
H	1.550580	-2.871873	-1.835072
H	2.300591	-1.900290	-3.133823
H	0.072416	-0.723201	-4.159171
H	-0.903959	-1.809029	-3.137604
H	-1.246565	-0.068059	-3.157765

TS1'

C	3.835604	1.747386	0.417622
C	2.561573	1.253269	0.116670
N	2.329331	-0.084920	0.047327
C	3.346793	-0.938772	0.281532
C	4.631530	-0.509060	0.588093
C	4.881207	0.862191	0.653754
C	1.397212	2.123593	-0.133847
N	0.228628	1.485703	-0.419898
C	-0.886560	2.215919	-0.614496
C	-0.882859	3.606696	-0.561102

C	0.312415	4.270832	-0.297271
C	1.463628	3.518063	-0.077670
Re	0.300995	-0.716286	-0.548389
C	-1.529186	-1.048518	-1.094276
O	-2.597549	-1.264338	-1.493985
C	0.618128	-2.609346	-0.447542
O	0.858604	-3.745711	-0.350066
P	-0.362670	-0.677752	1.893152
C	0.908792	0.068424	3.003157
C	0.843803	-0.693089	-2.420708
O	1.157423	-0.711443	-3.537850
C	-1.905119	0.220114	2.362016
C	-2.902506	0.507378	1.561383
C	-3.838801	0.802858	0.623528
O	-3.895847	1.840945	-0.101452
C	-0.568725	-2.347598	2.648230
O	-4.834903	-0.179705	0.511859
C	-5.653809	-0.090930	-0.655675
H	-1.898961	0.445465	3.438806
H	-6.166504	0.872152	-0.714211
H	-6.386565	-0.894111	-0.561920
H	-5.061186	-0.238659	-1.563554
H	0.351552	5.352259	-0.254417
H	5.871140	1.236304	0.883748
H	5.412603	-1.237075	0.765443
H	3.110429	-1.992451	0.211014
H	4.007542	2.814018	0.466365
H	2.401356	4.010421	0.142442
H	-1.815123	4.135089	-0.713631
H	-1.816232	1.689150	-0.790585
H	-1.379178	-2.875939	2.143400
H	0.352173	-2.925334	2.540089
H	-0.810116	-2.245115	3.709018
H	0.520075	0.107953	4.024349
H	1.822656	-0.529478	2.991003
H	1.143474	1.083185	2.674072

I1′

P	0.539642	-0.739325	-1.767118
Re	-0.681119	-0.758081	0.453888
C	-1.328159	-2.527377	0.084107
O	-1.753610	-3.579961	-0.182624
N	-0.172368	1.385750	0.604286
C	0.951099	1.850463	1.184179
C	1.233484	3.210050	1.276701
C	0.336699	4.122578	0.727366
C	-0.819041	3.646721	0.114095
C	-1.059589	2.270256	0.074203
C	-2.273241	1.683150	-0.527521
N	-2.340969	0.325314	-0.519300
C	-3.421964	-0.279440	-1.052041
C	-4.480821	0.428082	-1.605740
C	-4.424195	1.821905	-1.611642
C	-3.309502	2.452342	-1.069626
C	-1.616335	-0.737338	2.163339
O	-2.163676	-0.758320	3.187237
C	0.878686	-1.516567	1.322749
O	1.756633	-2.010816	1.899285
C	2.238632	-0.097674	-1.761862
C	2.936766	0.088046	-0.635417
C	4.269535	0.577518	-0.544693
O	5.214310	-0.429196	-0.492683
C	6.553389	-0.020941	-0.184607
O	4.612957	1.763797	-0.394060
H	2.618736	0.081141	-2.779759
H	6.927381	0.693662	-0.920971
H	7.149631	-0.931720	-0.207940
H	6.604835	0.434766	0.807847
H	1.642238	1.103906	1.548406
H	2.148464	3.531394	1.756824
H	0.531243	5.187742	0.769453
H	-5.232832	2.408969	-2.029476
H	-5.327479	-0.108169	-2.014740
H	-3.427360	-1.360789	-1.019581
H	-3.249812	3.531723	-1.060570
H	-1.523983	4.339259	-0.324718
C	0.668599	-2.406941	-2.546715
C	-0.309206	0.214147	-3.100153
H	1.249162	-3.061616	-1.894515
H	-0.324048	-2.840274	-2.690676
H	1.172401	-2.326903	-3.513998
H	0.295816	0.183554	-4.010200
H	-1.291765	-0.215976	-3.307712
H	-0.434800	1.254609	-2.793348

TS2*cco*

C	-3.968812	1.637307	-0.539531
C	-2.683365	1.165854	-0.247703
N	-2.384193	-0.158359	-0.338232
C	-3.346455	-1.016197	-0.736269
C	-4.634949	-0.607800	-1.054829
C	-4.954554	0.746595	-0.949285
C	-1.574467	2.050704	0.160806
N	-0.384652	1.437618	0.403504
C	0.683329	2.188581	0.738099
C	0.611758	3.571242	0.871886
C	-0.606603	4.207333	0.638638
C	-1.708235	3.438336	0.276793
Re	-0.355302	-0.776626	0.265064
C	1.532886	-1.049485	0.831307
O	2.431045	-1.418395	1.484343
C	-0.547863	-2.657360	-0.016340
O	-0.695547	-3.801624	-0.212513
P	0.392455	-0.460150	-2.113588
C	2.107492	0.135771	-2.095025
C	2.734291	0.176584	-0.919756
C	4.033467	0.647087	-0.572500
O	4.302278	1.793452	-0.178269
C	-0.897018	-0.987165	2.110970
O	-1.215372	-1.147380	3.220842
O	4.977940	-0.347491	-0.552768
C	6.269988	0.019690	-0.045319
H	2.528704	0.446969	-3.058139
H	6.740971	0.769086	-0.685323
H	6.855736	-0.897942	-0.047456
H	6.189928	0.416989	0.968733
H	-0.698826	5.282823	0.731008
H	-5.951570	1.103128	-1.178246
H	-5.365493	-1.341641	-1.370597
H	-3.058402	-2.057800	-0.788096
H	-4.196145	2.690882	-0.451431
H	-2.658627	3.914242	0.077595
H	1.500130	4.127082	1.142890
H	1.617480	1.656567	0.869304
C	-0.558402	0.766677	-3.111586
C	0.339852	-1.934875	-3.218503
H	0.790577	-1.702125	-4.186901
H	0.883356	-2.759362	-2.754535
H	-0.698246	-2.239476	-3.371884
H	-0.105423	0.885345	-4.099267
H	-1.587935	0.420396	-3.231607
H	-0.567873	1.733064	-2.604152

P*cco*

C	-1.926913	3.583585	-0.330358
C	-1.744840	2.201601	-0.193711
N	-0.529926	1.682498	0.135346
C	0.506857	2.525861	0.322022
C	0.389550	3.904639	0.195992
C	-0.852588	4.445085	-0.137508
C	-2.830669	1.222780	-0.393619
C	-4.143974	1.592599	-0.713676
C	-5.112324	0.611992	-0.894723
C	-4.743975	-0.727116	-0.749260
C	-3.427766	-1.028477	-0.426601
N	-2.479482	-0.083927	-0.245895
Re	-0.393684	-0.536199	0.335311
C	-0.728391	-0.516317	2.226863
O	-0.887732	-0.537590	3.387169
C	1.682397	-0.514578	0.759233
C	2.650338	-0.163210	-0.408207
C	4.022247	0.282274	-0.016244
O	4.977419	-0.621932	-0.274081
C	6.315610	-0.264647	0.144787
C	-0.469947	-2.430275	0.351562
O	-0.551021	-3.606363	0.330360
P	0.493306	-0.588554	-1.974783
C	0.489434	-2.147594	-2.954092
O	2.275024	-0.712561	1.834027
C	2.256100	-0.220357	-1.688413
O	4.236707	1.368045	0.498010
H	2.925963	-0.022966	-2.522393
H	6.936187	-1.120614	-0.108767
H	6.649754	0.627982	-0.385466
H	6.329730	-0.079193	1.219476
H	-0.983620	5.515384	-0.243508
H	-6.131496	0.887352	-1.140633

H	-5.459419	-1.529032	-0.878237
H	-3.101040	-2.051715	-0.293541
H	-4.407417	2.635941	-0.822121
H	-2.898331	3.983295	-0.587082
H	1.258158	4.529668	0.359371
H	1.457038	2.074798	0.577244
C	0.008954	0.663635	-3.237940
H	1.179120	-2.076117	-3.798117
H	0.776687	-2.980130	-2.310129
H	-0.520575	-2.327328	-3.330373
H	0.714758	0.658815	-4.072452
H	-0.989349	0.427609	-3.613496
H	-0.010431	1.657012	-2.785199

TS2*ccb*

C	-0.689161	3.295669	-0.667689
C	0.078986	2.154548	-0.477777
N	-0.356844	0.920921	-0.863484
C	-1.658476	0.766627	-1.262511
C	-2.416638	1.922589	-1.628259
C	-1.939710	3.169833	-1.311054
C	1.428774	2.188005	0.118733
N	2.045217	0.984174	0.267078
C	3.275892	0.934794	0.818067
C	3.950812	2.068282	1.248017
C	3.328244	3.310102	1.102199
C	2.061994	3.367045	0.533788
Re	0.951779	-0.786865	-0.465552
C	2.199803	-2.116749	0.153858
O	2.966139	-2.893325	0.568296
P	-0.287800	-0.832121	1.716894
C	0.367995	0.299743	3.014493
C	-2.066002	-0.441895	1.660108
C	-2.720102	-0.001267	0.582020
C	-4.119623	0.322610	0.518863
O	-4.596264	1.456443	0.635058
C	-0.240753	-2.468308	2.556925
O	-4.895992	-0.746219	0.154224
C	-6.289582	-0.468704	-0.066107
C	1.865586	-0.666265	-2.186308
O	2.414311	-0.621902	-3.208212
C	-0.214743	-2.171215	-1.126957
O	-0.936426	-2.991607	-1.528756
H	-1.887892	-0.177360	-1.731702
H	-3.378908	1.787432	-2.105107
H	-2.513598	4.057843	-1.550878
H	-0.318684	4.269989	-0.381204
H	3.717809	-0.049136	0.908815
H	4.937356	1.972955	1.683494
H	3.822783	4.219058	1.423796
H	1.563781	4.319151	0.411578
H	-2.559226	-0.610999	2.627338
H	-6.730644	-1.413616	-0.378889
H	-6.764344	-0.116781	0.852318
H	-6.414724	0.285741	-0.845031
H	0.796240	-2.743641	2.763354
H	-0.800814	-2.429684	3.494973
H	-0.680295	-3.222224	1.901167
H	1.418643	0.077251	3.215007
H	0.285936	1.331344	2.667930
H	-0.212354	0.183928	3.933511

P*ccb*

Re	0.979684	-0.811073	-0.324418
P	-0.138048	-0.459945	1.871533
N	-0.478107	0.731940	-0.782027
N	2.024265	1.103081	0.017773
C	1.708048	-0.931985	-2.131024
O	2.136065	-1.028680	-3.206238
C	-0.122922	-2.355963	-0.650142
O	-0.796710	-3.286601	-0.851698
C	2.366890	-1.947785	0.385288
O	3.217958	-2.600590	0.850194
C	-1.918204	-0.143125	1.648931
H	-2.529662	-0.295038	2.537780
C	-2.554924	0.175412	0.507573
C	-4.055558	0.187603	0.598650
O	-4.694631	0.535720	1.578125
O	-4.627555	-0.278662	-0.524400
C	-6.071348	-0.321310	-0.531744
H	-6.429780	-1.014035	0.230672
H	-6.345521	-0.666505	-1.525555
H	-6.474287	0.673779	-0.339695
C	-1.911379	0.443426	-0.876984
H	-2.028931	-0.496922	-1.428651
C	-2.628991	1.544245	-1.641072
H	-3.506057	1.281124	-2.218128
C	-2.174603	2.817514	-1.559114
H	-2.713251	3.622720	-2.050676
C	-0.931912	3.106550	-0.905849
C	-0.102158	2.033522	-0.621259
C	1.299230	2.233269	-0.204001
C	1.886510	3.495700	-0.019490
C	3.204564	3.594005	0.401249
H	3.658823	4.566594	0.551169
C	3.937232	2.422315	0.623877
H	4.969347	2.448968	0.947983
C	3.312207	1.203720	0.413411
H	3.838202	0.268975	0.562950
H	1.303429	4.387119	-0.204907
H	-0.593315	4.126016	-0.791069
C	-0.144197	-1.868467	3.050482
C	0.424201	0.949679	2.916364
H	0.885441	-2.079252	3.348503
H	-0.741544	-1.636236	3.935521
H	-0.550128	-2.751691	2.554401
H	1.488192	0.829673	3.133721
H	0.275897	1.888178	2.380379
H	-0.141075	0.973245	3.851242

TS2*ins*

C	-2.264596	1.340831	-0.583710
N	-2.101842	0.101207	-0.057159
C	-3.133116	-0.766612	-0.102838
C	-4.361764	-0.446470	-0.662855
C	-4.537969	0.827244	-1.207378
C	-3.479439	1.726200	-1.167534
Re	-0.144516	-0.372567	0.924045
N	-0.008891	1.723531	0.147380
C	1.075188	2.504972	0.309398
C	1.129094	3.819313	-0.140478
C	0.021103	4.349417	-0.798322
C	-1.104957	3.550234	-0.965921
C	-1.100445	2.239770	-0.476742
C	-0.770373	0.266951	2.583406
O	-1.148480	0.652879	3.624651
C	-0.554959	-2.164738	1.460621
O	-0.860577	-3.243155	1.786539
C	1.641779	-0.571061	1.616601
O	2.706852	-0.662123	2.076772
C	1.972451	-0.425376	-1.113117
C	3.234782	0.269685	-1.135125
O	4.275870	-0.506457	-0.704436
C	5.536696	0.162361	-0.536632
C	1.462002	-1.183282	-2.090700
P	-0.152060	-1.900966	-1.711993
C	-1.270283	-1.434029	-3.120343
C	0.010017	-3.722535	-2.036611
O	3.393729	1.470191	-1.388614
H	1.923061	2.052814	0.803338
H	2.029633	4.399424	0.012813
H	0.030006	5.365859	-1.173261
H	-1.978829	3.943172	-1.467339
H	-2.951322	-1.743011	0.327423
H	-5.157453	-1.181325	-0.668099
H	-5.482265	1.114710	-1.653807
H	-3.594431	2.717117	-1.584858
H	1.947967	-1.383111	-3.052378
H	5.895072	0.561465	-1.487897
H	5.447473	0.978666	0.183112
H	6.222994	-0.596082	-0.163906
H	0.707390	-4.166214	-1.323623
H	-0.965322	-4.201265	-1.912978
H	0.367020	-3.909181	-3.053442
H	-0.845776	-1.731854	-4.083589
H	-2.236041	-1.931098	-2.990034
H	-1.434878	-0.354173	-3.116108

P*ins*

Re	0.264844	-1.163147	-0.661440
N	1.533629	0.644338	-0.777339
N	1.090872	-0.861082	1.370453
C	1.735586	-2.385144	-1.064340
O	2.579625	-3.145745	-1.320397

C	-0.279044	-1.195192	-2.503064
O	-0.567173	-1.178191	-3.633938
C	-0.913129	-2.639473	-0.293186
O	-1.625718	-3.532551	-0.067013
C	1.734251	1.348890	-1.905987
C	2.501276	2.507400	-1.945736
H	2.634526	3.029643	-2.884058
C	3.071364	2.971383	-0.762126
H	3.667995	3.875691	-0.748086
C	2.862542	2.252366	0.411177
C	2.093878	1.084653	0.380194
C	1.866191	0.237663	1.567336
C	2.443123	0.499203	2.815518
C	2.242820	-0.391075	3.866133
H	2.687128	-0.199179	4.835642
C	1.472897	-1.531217	3.645137
H	1.295169	-2.257945	4.427379
C	0.912631	-1.725902	2.387949
H	0.296864	-2.589274	2.178162
H	3.052730	1.380212	2.963741
H	3.298200	2.597308	1.338845
H	1.260791	0.965190	-2.799290
C	-1.409050	0.260221	-0.172401
C	-2.215474	0.410492	0.900370
C	-1.682143	1.190876	-1.297834
O	-1.062168	2.216885	-1.556893
O	-2.689191	0.746742	-2.091127
C	-2.942641	1.505977	-3.288967
H	-3.810432	1.039327	-3.751557
H	-3.149718	2.548788	-3.045926
H	-2.081386	1.448926	-3.958000
H	-2.984989	1.192697	0.882564
P	-2.220934	-0.587864	2.440529
C	-4.029372	-0.447813	2.866613
C	-1.565176	0.670345	3.656262
H	-4.197251	-0.854814	3.868344
H	-4.374327	0.592587	2.846099
H	-4.622913	-1.030940	2.158486
H	-0.493380	0.815417	3.498302
H	-2.075567	1.633959	3.554265
H	-1.713769	0.297788	4.674602

Figure S6. PCM-B3LYP/6-31+G(d,p) (LANL2DZ for Re) optimized geometries in THF solution of the critical structures involved in the reaction between the complex [Re(PMe_2)$(CO)_3$(bipy)] (bipy = 2,2'-bipyridine) and methyl propiolate (HMAD, HC≡CCO_2Me). Relevant distances are given in angstroms.

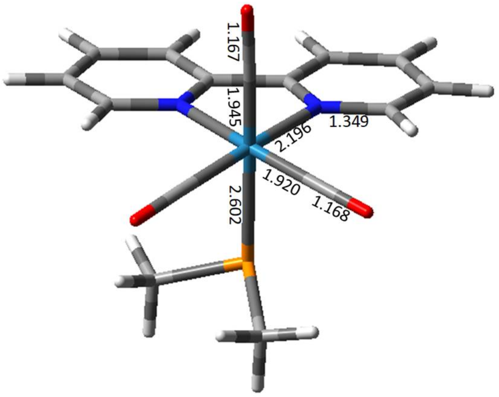

[Re(PMe_2)$(CO)_3$(bipy)]

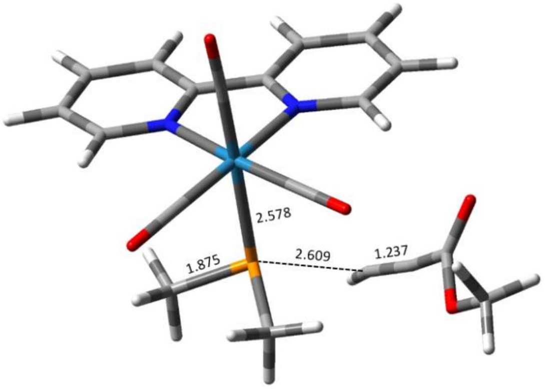

TS1

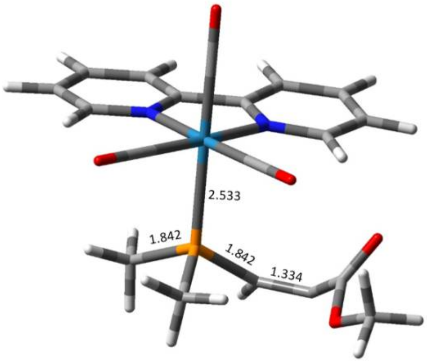

I1

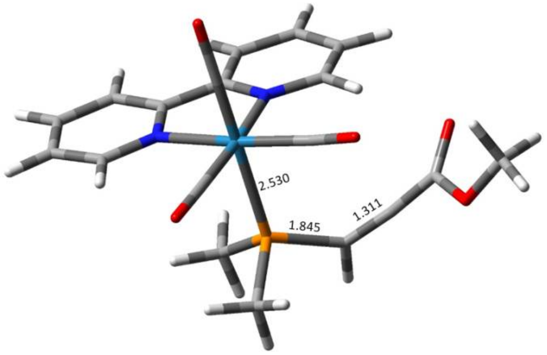

TS1'

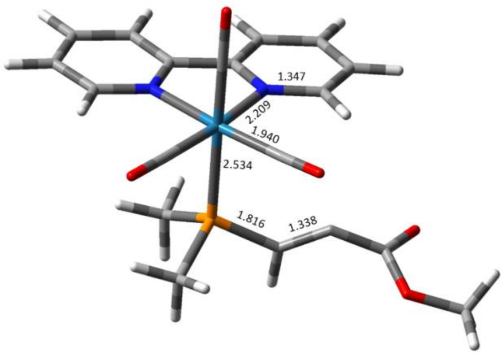

I1'

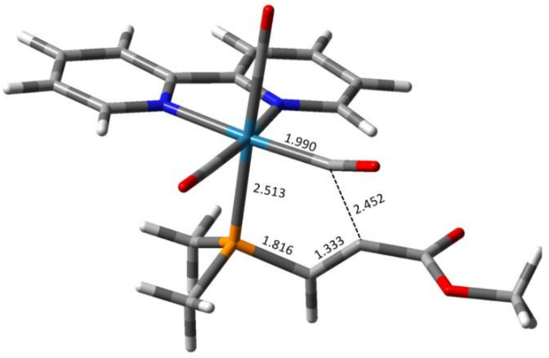

TS2*cco*

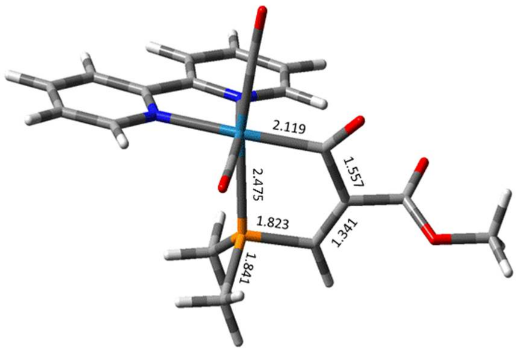

P*cco*

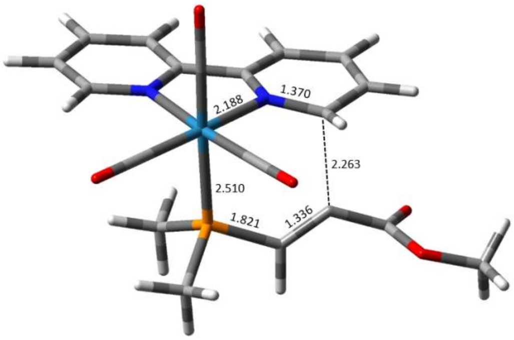

TS2*ccb*

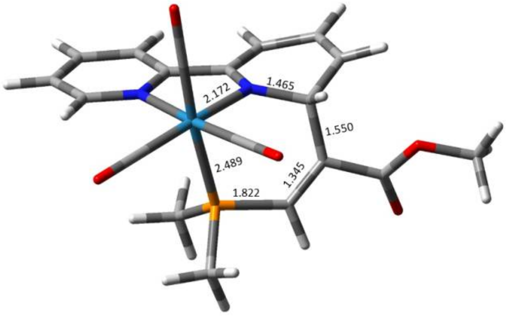

P*ccb*

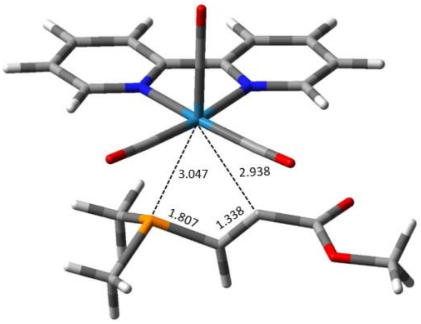

TS2*ins*

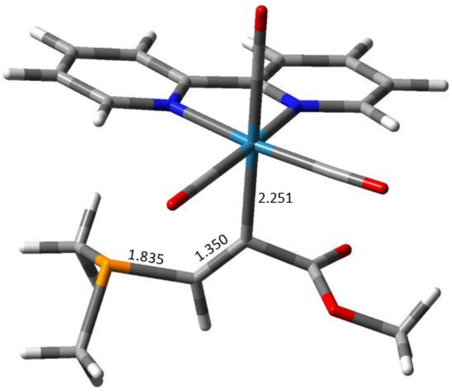

P*ins*

Table S19. PCM-B3LYP/6-31+G(d,p) (LANL2DZ for Re) energies without and with including thermal corrections (E and G, respectively), enthalpies (H), and entropies (S), and CPCM-DLPNO-CCSD(T)/def2-TZVPP//PCM-B3LYP/6-31+G(d,p) (LANL2DZ for Re) energies without and with including thermal corrections (E' and G', respectively) in THF solution of the critical structures involved in the reaction between the complex [Re(PHPh)(CO)$_3$(bipy)] (bipy = 2,2'-bipyridine) and methyl propiolate (HMAD, HC≡CCO$_2$Me). All the values are given in hartree, except entropies that are in cal/K mol.[a]

Species	E	H	S	G	E'	G'[b]
[Re(PHPh)(CO)$_3$(bipy)]	-1488.258510	-1487.944989	165.672	-1488.023705	-1484.956566	-1484.721761
HMAD	-305.215726	-305.136831	77.228	-305.173524	-304.738441	-304.696239
Reactants	-1793.474236	-1793.081820	242.900	-1793.197229	-1789.695007	-1789.418000
**TS1**	-1793.459196	-1793.066744	201.581	-1793.162522	-1789.686638	-1789.389964
**I1**	-1793.480134	-1793.085245	197.762	-1793.179208	-1789.709987	-1789.409061
**TS1'**	-1793.476942	-1793.083241	193.427	-1793.175144	-1789.704418	-1789.402620
**I1'**	-1793.482814	-1793.087951	198.734	-1793.182376	-1789.712812	-1789.412374
**TS2*cco***	-1793.479471	-1793.086091	196.231	-1793.179327	-1789.705021	-1789.404877
**P*cco***	-1793.504175	-1793.108501	199.136	-1793.203117	-1789.730844	-1789.429786
**P*ccoh***	-1793.516560	-1793.118472	193.883	-1793.210592	-1789.730664	-1789.424696
**TS2*ccb***	-1793.475322	-1793.081700	192.366	-1793.173099	-1789.705252	-1789.403029
**P*ccb***	-1793.503194	-1793.107369	192.407	-1793.198788	-1789.742869	-1789.438463
**TS2*ins***	-1793.456995	-1793.063717	204.238	-1793.160757	-1789.686377	-1789.390139
**P*ins***	-1793.510249	-1793.115008	199.226	-1793.209667	-1789.746483	-1789.445901

[a] Thermal magnitudes were computed in THF solution at 298.15 K and 1 atm. [b] For each species, G' was calculated as G' = G – E + E', in which G is the PCM-B3LYP/6-31+G(d,p) (LANL2DZ for Re) energy with including thermal corrections and E and E' are the PCM-B3LYP/6-31+G(d,p) (LANL2DZ for Re) and CPCM-DLPNO-CCSD(T)/def2-TZVPP//PCM-B3LYP/6-31+G(d,p) (LANL2DZ for Re) energies without including thermal corrections, respectively.

Table S20. PCM-B3LYP/6-31+G(d,p) (LANL2DZ for Re) relative energies without and with including thermal corrections (ΔE and ΔG, respectively), enthalpies (ΔH), and entropic contributions (TΔS), and CPCM-DLPNO-CCSD(T)/def2-TZVPP//PCM-B3LYP/6-31+G(d,p) (LANL2DZ for Re) relative energies without and with including thermal corrections (E' and G', respectively) in THF solution of the critical structures involved in the reaction between the complex [Re(PHPh)$(CO)_3$(bipy)] (bipy = 2,2'-bipyridine) and methyl propiolate (HMAD, HC≡CCO_2Me). All the values are given in kcal/mol.[a]

Species	ΔE	ΔH	TΔS	ΔG	ΔE'	ΔG'
[Re(PHPh)$(CO)_3$(bipy)] + HMAD	0.0	0.0	0.0	0.0	0.0	0.0
**TS1**	9.4	9.5	-12.3	21.8	5.3	17.6
**I1**	-3.7	-2.1	-13.5	11.3	-9.4	5.6
**TS1'**	-1.7	-0.9	-14.8	13.9	-5.9	9.7
**I1'**	-5.4	-3.8	-13.2	9.3	-11.2	3.5
**TS2*cco***	-3.3	-2.7	-13.9	11.2	-6.3	8.2
**P*cco***	-18.8	-16.7	-13.0	-3.7	-22.5	-7.4
**P*ccoh***	-26.6	-23.0	-14.6	-8.4	-22.4	-4.2
**TS2*ccb***	-0.7	0.1	-15.1	15.1	-6.4	9.4
**P*ccb***	-18.2	-16.0	-15.1	-1.0	-30.0	-12.8
**TS2*ins***	10.8	11.4	-11.5	22.9	5.4	17.5
**P*ins***	-22.6	-20.8	-13.0	-7.8	-32.3	-17.5

[a] Thermal magnitudes were evaluated in THF solution at 298.15 K and 1 atm.

Table S21. PCM-B3LYP/6-31+G(d,p) (LANL2DZ for Re) optimized cartesian coordinates, in Å, for the critical structures involved in the reaction of the complex [Re(PHPh)(CO)$_3$(bipy)] (bipy = 2,2'-bipyridine) towards methyl propiolate (HMAD, HC≡CCO$_2$Me).

[Re(PHPh)(CO)$_3$(bipy)]

Re	-0.074771	-0.176251	0.194627
P	-0.010011	-0.623843	2.745729
N	2.113970	-0.028929	0.377046
N	0.676638	-2.242355	0.102223
C	-0.051970	0.039308	-1.733654
O	-0.091071	0.172217	-2.891562
C	-0.449379	1.688947	0.459398
O	-0.621296	2.830585	0.632557
C	-1.953230	-0.586292	0.223306
O	-3.082273	-0.874063	0.258956
C	2.780930	1.135927	0.499996
C	4.161131	1.201154	0.636465
H	4.644248	2.165100	0.732148
C	4.891193	0.011728	0.645450
H	5.969709	0.022147	0.746958
C	4.210774	-1.194242	0.523264
C	2.816215	-1.193492	0.396940
C	2.015410	-2.424107	0.263938
C	2.570021	-3.709780	0.293863
C	1.746135	-4.818856	0.142609
H	2.165401	-5.817431	0.165334
C	0.374993	-4.622565	-0.031086
H	-0.307149	-5.453240	-0.156251
C	-0.116274	-3.323996	-0.042397
H	-1.172283	-3.125027	-0.167243
H	3.633563	-3.842140	0.437900
H	4.759815	-2.125879	0.523598
C	-1.515365	0.020943	3.593477
C	-1.713162	1.409674	3.733287
C	-2.437156	-0.834105	4.229409
C	-2.779235	1.919192	4.477226
H	-1.024402	2.100560	3.256314
C	-3.503112	-0.325648	4.976743
H	-2.319231	-1.910037	4.141299
C	-3.680005	1.054630	5.108948
H	-2.904290	2.994114	4.567256
H	-4.197140	-1.010684	5.453882
H	-4.505903	1.449610	5.689911
H	-0.420282	-1.987638	2.740953
H	2.176383	2.033265	0.484986

TS1

C	4.249248	0.491879	-1.464673
C	3.067147	0.064094	-0.847125
N	2.378529	0.881588	-0.006529
C	2.853192	2.122914	0.229345
C	4.021403	2.602138	-0.347456
C	4.733541	1.770538	-1.212254
C	2.488333	-1.276446	-1.058119
N	1.317734	-1.524402	-0.410994
C	0.711390	-2.714425	-0.590493
C	1.256316	-3.717813	-1.386549
C	2.469281	-3.483045	-2.031208
C	3.087008	-2.247001	-1.867905
Re	0.540902	0.070708	0.909866
P	-0.648566	0.840952	-1.211253
C	-1.777604	2.273981	-1.024873
C	-1.781174	3.314608	-1.976502
C	-2.734312	4.333822	-1.930934
C	-3.710988	4.339870	-0.930671
C	-3.720180	3.317512	0.022006
C	-2.765922	2.298318	-0.022829
C	0.039315	1.664447	1.857750
O	-0.223972	2.651735	2.417763
C	-1.061921	-0.793580	1.539725
O	-2.025679	-1.315876	1.938053
C	1.536734	-0.594599	2.439813
O	2.115836	-0.963895	3.379440
C	-2.093806	-1.027122	-2.496973
C	-2.605394	-1.878818	-1.772151
C	-3.004431	-2.756627	-0.730515
O	-2.347930	-3.725529	-0.346314
O	-4.205336	-2.424181	-0.208123
C	-4.658249	-3.240065	0.890117
H	-1.876098	-0.519300	-3.411709
H	-2.788700	1.513540	0.729418
H	-4.471676	3.313727	0.807590
H	-2.710729	5.123821	-2.675785
H	-1.032667	3.322693	-2.763625
H	-3.999432	-3.102343	1.748904
H	-4.674287	-4.292513	0.601741
H	-5.662136	-2.887388	1.114103
H	-0.233118	-2.869396	-0.086643
H	0.726352	-4.655867	-1.491693
H	2.925215	-4.243495	-2.654101
H	5.647451	2.111358	-1.686295
H	4.357273	3.605780	-0.120955
H	2.269496	2.735848	0.903208
H	4.025010	-2.040405	-2.364611
H	4.782464	-0.165407	-2.135086
H	-4.453764	5.131500	-0.894745
H	0.364226	1.509238	-1.963702

I1

C	3.238080	-1.380022	0.070616
C	2.380999	-1.540637	-1.029313
C	2.709648	-2.483563	-2.018037
C	3.873486	-3.247968	-1.907244
C	4.723383	-3.078162	-0.810458
C	4.402608	-2.142617	0.177309
P	0.884857	-0.490383	-1.207743
Re	-0.507466	-0.153115	0.880040
C	-1.665417	0.138095	2.419644
O	-2.328382	0.320495	3.353335
N	-2.089235	-1.322455	-0.122264
C	-2.990283	-0.632750	-0.870359
C	-4.063345	-1.286649	-1.486816
C	-4.215091	-2.660184	-1.331262
C	-3.285308	-3.358887	-0.561021
C	-2.239851	-2.654580	0.021596
C	-2.758479	0.820486	-0.977801
N	-1.663744	1.300150	-0.324365
C	-1.373653	2.612921	-0.407269
C	-2.179730	3.508051	-1.105685
C	-3.314483	3.032959	-1.756475
C	-3.598047	1.670700	-1.701114
C	0.832736	0.991701	1.683316
O	1.635640	1.636479	2.218761
C	0.315178	-1.660742	1.745251
O	0.772159	-2.607215	2.245663
C	1.354737	0.933154	-2.306177
C	1.882689	2.104001	-1.971539
C	2.225218	2.804864	-0.800867
O	3.527712	2.579546	-0.393538
C	4.027710	3.466345	0.616804
O	1.532034	3.670085	-0.223465
H	1.186962	0.654911	-3.349623
H	4.007951	4.502427	0.270202
H	5.056103	3.153811	0.795927
H	3.441501	3.383581	1.533645
H	-0.458284	2.949736	0.066143
H	-1.902545	4.553310	-1.136876
H	-3.963236	3.703060	-2.307935
H	-5.043618	-3.174394	-1.803014
H	-3.359843	-4.428070	-0.408296
H	-1.499590	-3.157679	0.629191
H	2.056518	-2.622947	-2.873115
H	4.114386	-3.974323	-2.676067
H	5.627032	-3.671488	-0.725271
H	5.056115	-2.007517	1.031752
H	2.999809	-0.659640	0.846633
H	-4.774678	-0.728696	-2.079645
H	-4.464232	1.276891	-2.214781
H	0.167568	-1.291653	-2.123918

TS1′

C	2.031271	-3.137147	-1.977275
C	2.022413	-2.111954	-1.018577
C	2.987590	-2.122155	0.002308
C	3.949624	-3.132046	0.050818
C	3.956024	-4.146065	-0.912483
C	2.993217	-4.149171	-1.924358
P	0.845649	-0.711686	-1.148761
C	1.763599	0.628204	-2.044458
C	2.124710	1.752073	-1.481052
C	2.455674	2.935510	-0.900880
O	3.724910	2.922039	-0.309104
C	3.996412	4.017121	0.569865
Re	-0.449497	-0.046253	0.908110
C	0.264073	-1.504127	1.940218
O	0.666667	-2.407958	2.554067
N	-1.462511	1.383114	-0.444583
C	-2.600288	0.949857	-1.055146
C	-3.345670	1.799722	-1.876944
C	-2.910364	3.105404	-2.091091
C	-1.730070	3.527246	-1.486031
C	-1.033763	2.639985	-0.670431
C	-2.972763	-0.456114	-0.801921
N	-2.149832	-1.148287	0.030512
C	-2.427452	-2.440199	0.299865
C	-3.523881	-3.101397	-0.237482
C	-4.373177	-2.400724	-1.094193
C	-4.092845	-1.068434	-1.376612
C	-1.548418	0.513671	2.415489
O	-2.195414	0.839788	3.321149
C	0.993628	1.082160	1.549117
O	1.820660	1.753886	2.009819
O	1.747171	3.983026	-0.847606
H	-0.007205	-1.240739	-2.147520
H	1.998133	0.334632	-3.075085
H	3.944954	4.974433	0.044999
H	5.007938	3.850893	0.940965
H	3.292078	4.029997	1.406600
H	-0.097121	2.946196	-0.221369
H	-1.330455	4.521166	-1.636783
H	-3.481313	3.772473	-2.725957
H	-5.238103	-2.882381	-1.534158
H	-3.697465	-4.140076	0.013499
H	-1.745558	-2.945743	0.970182
H	-4.737854	-0.510871	-2.041345
H	-4.253738	1.450158	-2.348978
H	1.289494	-3.144537	-2.769694
H	2.989630	-4.934091	-2.674055
H	4.706430	-4.929077	-0.873209
H	4.694104	-3.128530	0.840428
H	2.990747	-1.338179	0.754106

I1′

P	-0.200492	0.025074	-0.000132
C	-0.112631	-0.097479	1.830133
C	1.098936	-0.384175	2.478124
C	1.157806	-0.449432	3.873223
C	0.007083	-0.227096	4.633687
C	-1.203505	0.061833	3.995348
C	-1.263785	0.125376	2.602396
Re	-2.062683	-1.232150	-1.151547
C	-2.653343	-2.052522	0.482880
O	-2.985399	-2.598550	1.456877
N	-1.125890	-0.564513	-3.043613
C	-1.477731	0.558097	-3.699825
C	-0.923483	0.905634	-4.928353
C	0.040197	0.073717	-5.492285
C	0.428833	-1.071995	-4.802970
C	-0.172213	-1.373744	-3.577116
C	0.176196	-2.568601	-2.782845
N	-0.530824	-2.746152	-1.635158
C	-0.266432	-3.818926	-0.862361
C	0.711609	-4.751728	-1.182071
C	1.444839	-4.576811	-2.355428
C	1.168377	-3.479444	-3.163410
C	-3.434530	-2.198707	-2.138786
O	-4.266489	-2.766005	-2.715253
C	-3.296708	0.234759	-0.849486
O	-4.092521	1.058020	-0.659839
C	0.015847	1.779799	-0.427989
C	-0.870904	2.423844	-1.195401
C	-0.747719	3.772860	-1.637963
O	-1.390590	4.680392	-0.823576
C	-1.447228	6.027955	-1.313646
O	-0.233149	4.144968	-2.706418
H	0.930434	2.214180	-0.002737
H	-0.444859	6.448553	-1.421276
H	-2.010545	6.588619	-0.568764
H	-1.953539	6.069905	-2.280771
H	-2.196152	1.193271	-3.202010
H	-1.243329	1.816035	-5.417934
H	0.494078	0.315068	-6.446553
H	2.215977	-5.283293	-2.639647
H	0.886099	-5.591558	-0.522546
H	-0.862036	-3.920417	0.035165
H	1.997014	-0.556272	1.893791
H	2.099910	-0.671588	4.362983
H	0.051951	-0.281177	5.715987
H	-2.099734	0.232747	4.581760
H	-2.208413	0.341649	2.113335
H	1.723975	-3.328323	-4.078539
H	1.183616	-1.725033	-5.218691
H	1.054363	-0.557502	-0.304106

TS2*cco*

C	-1.820615	-2.151092	-0.689962
N	-0.509062	-1.906060	-0.500184
C	0.407975	-2.659601	-1.165049
C	0.008385	-3.685209	-2.028520
C	-1.346621	-3.943414	-2.209603
C	-2.278095	-3.161136	-1.529119
Re	0.285229	-0.380961	0.910906
C	1.249213	0.856694	1.999371
O	1.894154	1.584581	2.649807
C	1.823799	-2.317817	-0.922263
N	2.045796	-1.281579	-0.069256
C	3.316633	-0.902948	0.178101
C	4.414138	-1.538812	-0.386682
C	4.196593	-2.613142	-1.249554
C	2.889343	-3.002986	-1.518344
P	0.088387	1.217286	-1.017662
C	0.962923	2.830423	-1.043533
C	1.677469	3.245765	-2.178492
C	2.308029	4.493465	-2.202151
C	2.220021	5.341783	-1.095273
C	1.510081	4.934757	0.039154
C	0.884327	3.687351	0.066255
C	0.315625	-1.656293	2.362684
O	0.318617	-2.408279	3.252358
C	-1.453360	0.304348	1.618347
C	-2.537032	1.053530	-0.404068
C	-3.955922	1.214530	-0.329336
O	-4.640720	0.160992	-0.890157
C	-6.070342	0.197160	-0.742607
C	-1.680010	1.547749	-1.296324
O	-2.267134	0.567803	2.417267
O	-4.551725	2.108519	0.281276
H	-1.934986	2.147165	-2.175150
H	-6.351116	0.209925	0.312194
H	-6.435268	-0.708717	-1.224900
H	-6.488206	1.080289	-1.231891
H	-1.666942	-4.737018	-2.874067
H	5.026981	-3.137236	-1.706358
H	5.412029	-1.193762	-0.148975
H	3.440894	-0.073505	0.861371
H	1.741139	2.595930	-3.045193
H	2.856019	4.804640	-3.085480
H	2.705702	6.311888	-1.113179
H	1.441044	5.587256	0.901987
H	0.336847	3.381331	0.951264
H	2.702089	-3.833040	-2.186309
H	0.745527	-4.274293	-2.556202
H	-3.343133	-3.319505	-1.643315
H	-2.506774	-1.494311	-0.170790
H	0.562179	0.719670	-2.254516

P*cco*

C	0.351964	-0.294718	0.871806
N	-0.219741	0.379354	-0.147562
C	-0.014170	-0.055662	-1.420603
C	0.770761	-1.185956	-1.678207
C	1.357043	-1.875457	-0.622653
C	1.145288	-1.419317	0.678820
Re	-1.468459	2.206207	0.113810

C	-2.567409	3.753177	0.102663
O	-3.242195	4.716974	0.044733
C	-0.656939	0.738904	-2.484621
N	-1.376111	1.814633	-2.063381
C	-1.981756	2.586996	-2.990145
C	-1.898085	2.336935	-4.353728
C	-1.158674	1.236905	-4.793026
C	-0.536029	0.431723	-3.846060
P	-3.391276	0.691735	0.457536
H	-3.356221	-0.607518	-0.098987
C	0.073620	3.345577	0.191315
O	0.997563	4.059563	0.273943
C	-1.442292	2.098419	2.228440
C	-2.330069	1.014573	2.910194
C	-2.021700	0.732012	4.345962
O	-2.879315	1.311204	5.196249
C	-2.607077	1.118544	6.604233
C	-3.277755	0.339433	2.244540
C	-5.150976	1.076377	0.121805
C	-5.950795	0.175320	-0.598429
C	-7.290892	0.473095	-0.860005
C	-7.844638	1.668498	-0.394685
C	-7.052473	2.570437	0.322695
C	-5.710394	2.281575	0.576375
O	-0.773951	2.752513	3.047527
O	-1.070230	0.051870	4.692797
H	-3.930870	-0.389044	2.715493
H	-2.672951	0.059071	6.855301
H	-3.372857	1.686762	7.126153
H	-1.613044	1.494323	6.846651
H	1.968440	-2.749469	-0.813414
H	-1.065120	1.013633	-5.849150
H	-2.400374	2.997494	-5.049218
H	-2.538074	3.432822	-2.607529
H	-5.530310	-0.759763	-0.954626
H	-7.898988	-0.229975	-1.418826
H	-8.885840	1.898450	-0.593468
H	-7.476412	3.501387	0.682506
H	-5.103183	2.996610	1.120109
H	0.044861	-0.423803	-4.163646
H	0.923953	-1.521700	-2.694321
H	1.581408	-1.919945	1.533722
H	0.161239	0.088149	1.866586

P*ccoh*

C	-4.370376	0.340595	-0.948500
C	-3.032526	0.398080	-0.538757
N	-2.309203	-0.734959	-0.319821
C	-2.911575	-1.928501	-0.514530
C	-4.234118	-2.050771	-0.919039
C	-4.982017	-0.892221	-1.140862
C	-2.317670	1.664217	-0.303522
N	-1.018841	1.542570	0.081613
C	-0.306788	2.658831	0.337593
C	-0.841513	3.934915	0.217093
C	-2.171680	4.070599	-0.184377
C	-2.913926	2.924052	-0.443542
Re	-0.203921	-0.498751	0.297626
C	1.770764	0.065809	0.577835
O	2.183227	0.582151	1.742281
C	0.274738	-2.344698	0.327904
O	0.540510	-3.487820	0.308393
P	0.687930	-0.494766	-2.094753
C	2.387860	-0.483602	-1.724975
C	2.788900	-0.025076	-0.464907
C	4.179182	0.330513	-0.193121
O	4.587094	0.765715	0.900083
C	-0.496363	-0.473721	2.179581
O	-0.615920	-0.479388	3.347861
O	5.022864	0.154418	-1.224147
C	6.411040	0.460602	-0.977512
H	3.134887	-0.754718	-2.464957
H	6.525355	1.512375	-0.714098
H	6.923289	0.240490	-1.911201
H	6.796855	-0.161053	-0.168966
H	-2.623053	5.049918	-0.290005
H	-6.016671	-0.948166	-1.455886
H	-4.662403	-3.035835	-1.054688
H	-2.299372	-2.802103	-0.331721
H	-4.928270	1.252972	-1.110772
H	-3.947872	3.008694	-0.749604
H	-0.223446	4.796425	0.435322
H	0.715417	2.505614	0.657290
H	3.160959	0.783145	1.687034

C	0.491122	-1.868285	-3.292551
C	-0.439520	-1.752777	-4.341418
C	-0.593739	-2.777986	-5.277273
C	0.183368	-3.936988	-5.182652
C	1.103155	-4.070759	-4.138088
C	1.248642	-3.051566	-3.193758
H	-1.032725	-0.848386	-4.434909
H	-1.310838	-2.668362	-6.084010
H	0.069438	-4.730221	-5.913056
H	1.699331	-4.973189	-4.050825
H	1.938975	-3.175781	-2.366452

TS2*ccb*

C	-1.585586	3.313291	0.170065
C	-1.175360	2.668777	-1.007947
C	-1.236629	3.364335	-2.225605
C	-1.701772	4.681678	-2.265559
C	-2.115856	5.313201	-1.090441
C	-2.055417	4.627259	0.126638
P	-0.618964	0.922308	-0.962720
C	-2.162547	-0.041741	-1.138472
C	-2.444556	-1.135180	-0.426399
C	-3.663103	-1.896379	-0.521769
O	-4.629469	-1.458467	0.342628
C	-5.828339	-2.251390	0.400161
Re	0.963104	0.233497	0.860123
N	2.343837	-0.398195	-0.745591
C	2.150079	-1.644913	-1.255711
C	3.013747	-2.154992	-2.233932
C	4.074467	-1.384192	-2.692535
C	4.258698	-0.103620	-2.166299
C	3.373518	0.348168	-1.197554
C	0.992768	-2.391581	-0.724250
N	0.300842	-1.762453	0.268168
C	-0.889411	-2.292280	0.696793
C	-1.191615	-3.657436	0.388762
C	-0.443669	-4.317271	-0.553741
C	0.642906	-3.658760	-1.170715
C	2.200524	-0.429227	2.213357
O	2.931567	-0.801788	3.033496
C	-0.377751	0.534899	2.209056
O	-1.195862	0.684444	3.023635
C	1.627592	2.030377	1.092439
O	2.046940	3.111527	1.204725
O	-3.813524	-2.920587	-1.193949
H	-0.032890	0.846538	-2.246113
H	-1.278350	-1.865997	1.608729
H	-2.039737	-4.127283	0.869778
H	-0.675767	-5.341175	-0.825095
H	1.212905	-4.154893	-1.943525
H	3.480241	1.332255	-0.758745
H	5.068849	0.535319	-2.493370
H	4.747133	-1.775697	-3.446502
H	2.855673	-3.149666	-2.626341
H	-0.917157	2.880415	-3.142845
H	-1.741102	5.210535	-3.212032
H	-2.478370	6.334688	-1.120582
H	-2.372008	5.115211	1.041898
H	-1.542382	2.794035	1.122013
H	-2.851485	0.395883	-1.869719
H	-6.458149	-1.773529	1.148654
H	-6.332052	-2.260703	-0.568555
H	-5.599820	-3.277484	0.693588

P*ccb*

Re	-0.039644	0.138648	-0.181691
P	-0.061372	0.141565	2.322799
N	2.041542	0.114844	0.444373
N	0.488375	-1.994456	0.026733
C	0.307781	0.061164	-2.090771
O	0.514758	0.016447	-3.232961
C	-0.268149	2.048468	-0.288826
O	-0.382657	3.206044	-0.363359
C	-1.933285	-0.117032	-0.459499
O	-3.075662	-0.314955	-0.597208
C	1.158544	1.337878	2.957575
H	0.931197	1.774166	3.927186
C	2.245202	1.783385	2.302534
C	2.978953	2.909761	2.974494
O	3.044780	3.077937	4.181261
O	3.533412	3.747865	2.082779
C	4.224894	4.892445	2.629245

H	3.530007	5.511764	3.197995
H	4.609387	5.435037	1.769025
H	5.037749	4.564591	3.277488
C	2.731175	1.323462	0.908397
H	2.450761	2.146578	0.238498
C	4.239855	1.153591	0.844228
H	4.845688	2.038402	0.696921
C	4.785370	-0.075554	0.989888
H	5.864915	-0.196040	0.994893
C	3.954708	-1.244747	1.040314
C	2.626767	-1.097831	0.680661
C	1.762294	-2.266671	0.422452
C	2.189320	-3.598753	0.551526
C	1.313358	-4.639753	0.282522
H	1.641018	-5.667955	0.382808
C	0.007763	-4.343123	-0.125793
H	-0.711192	-5.120918	-0.350547
C	-0.358690	-3.012548	-0.242777
H	-1.354682	-2.731412	-0.559207
H	3.204708	-3.808352	0.857629
H	4.389850	-2.219157	1.207246
C	-1.519564	0.398047	3.395740
C	-2.088666	1.673905	3.532080
C	-2.107273	-0.692981	4.053879
C	-3.223123	1.855999	4.325432
H	-1.647040	2.527180	3.027244
C	-3.247800	-0.508551	4.839419
H	-1.672094	-1.683173	3.960833
C	-3.805646	0.765038	4.977802
H	-3.652374	2.846334	4.430418
H	-3.694213	-1.357140	5.346079
H	-4.687936	0.908031	5.592161
H	0.392466	-1.096591	2.826888

TS2*ins*

Re	0.711578	-0.538933	-1.026702
C	1.440540	-2.168088	-1.637245
O	1.893051	-3.170421	-2.040720
C	0.003611	-0.237840	-2.783627
O	-0.449485	-0.105028	-3.850576
C	2.317701	0.415715	-1.505614
O	3.310048	0.949735	-1.791187
N	1.258177	-1.105862	1.067271
C	2.466264	-0.862001	1.607675
C	2.828231	-1.308097	2.872845
C	1.896813	-2.023067	3.623131
C	0.641809	-2.270614	3.076340
C	0.346941	-1.808234	1.789040
N	-1.062378	-1.555647	-0.137604
C	-2.219230	-1.739998	-0.804806
C	-3.295546	-2.433655	-0.269763
C	-3.179830	-2.957948	1.018120
C	-1.995568	-2.761818	1.718361
C	-0.944612	-2.053656	1.120913
P	-1.238982	1.728274	-0.629570
C	-2.942098	1.850181	0.064105
C	-3.317532	1.030689	1.143388
C	-4.615951	1.078766	1.655040
C	-5.565973	1.930941	1.083409
C	-5.209633	2.740034	0.000973
C	-3.906555	2.701578	-0.502692
C	-0.065063	2.595762	0.438404
C	1.125935	1.992076	0.513941
C	2.288867	2.471392	1.215017
O	2.630274	2.139113	2.356760
O	3.094261	3.253337	0.433547
C	4.359189	3.625629	1.004988
H	3.151920	-0.287513	1.001695
H	3.815038	-1.085723	3.256554
H	2.138732	-2.381902	4.616262
H	-0.095774	-2.821279	3.643588
H	-2.270220	-1.310632	-1.796775
H	-4.198733	-2.552910	-0.854056
H	-3.996197	-3.508704	1.469584
H	-1.887703	-3.158272	2.718438
H	-2.595842	0.348648	1.583911
H	-4.889052	0.444873	2.492001
H	-6.575917	1.963173	1.477806
H	-5.941403	3.403742	-0.447377
H	-3.636648	3.339991	-1.338182
H	-0.336520	3.546232	0.906060
H	4.217940	4.187270	1.930436
H	4.963294	2.739547	1.212752
H	4.847206	4.246739	0.255885
H	-1.439359	2.632811	-1.704283

P*ins*

Re	3.356908	-2.466848	2.271343
N	3.259643	-4.575083	1.607996
N	4.959070	-2.703811	0.759341
C	4.613030	-2.898669	3.701745
O	5.356366	-3.133274	4.567202
C	1.893594	-2.535697	3.514363
O	1.019412	-2.616286	4.282132
C	3.535908	-0.589901	2.665897
O	3.641732	0.534519	2.951483
C	2.403376	-5.485025	2.110125
C	2.350856	-6.797803	1.659259
C	3.208946	-7.187460	0.632334
C	4.088602	-6.250083	0.101249
C	4.103009	-4.947522	0.610909
C	5.049548	-3.913336	0.146670
C	6.019458	-4.156847	-0.831624
C	6.922155	-3.154152	-1.170826
C	6.835680	-1.923737	-0.521995
C	5.838912	-1.738935	0.430041
H	1.750776	-5.140605	2.901713
H	1.648244	-7.489033	2.106529
H	3.194600	-8.201705	0.250020
H	4.763566	-6.532100	-0.694808
H	6.075993	-5.122371	-1.314717
H	7.680224	-3.335083	-1.923920
H	7.519552	-1.114620	-0.744234
H	5.725323	-0.794926	0.944440
C	1.969932	-2.053617	0.551519
C	1.973310	-1.121182	-0.427280
P	3.209322	0.220005	-0.632788
H	1.185136	-1.130598	-1.185382
H	3.696198	-0.130983	-1.923674
C	2.136852	1.604923	-1.224273
C	1.051731	2.039590	-0.443972
H	0.800599	1.510383	0.470253
C	0.286825	3.142453	-0.832975
C	0.597675	3.836977	-2.006212
C	1.677259	3.416802	-2.788669
C	2.436449	2.308442	-2.402783
H	-0.549518	3.459583	-0.218830
H	0.004520	4.693253	-2.308171
H	1.924131	3.944324	-3.704408
H	3.263619	1.984647	-3.026934
C	0.852858	-3.029950	0.426591
O	0.853770	-4.022220	-0.292396
O	-0.188868	-2.736873	1.240223
C	-1.307865	-3.645068	1.200342
H	-1.734935	-3.678626	0.196343
H	-2.030592	-3.246881	1.909376
H	-0.995602	-4.649137	1.493054

Figure S7. PCM-B3LYP/6-31+G(d,p) (LANL2DZ for Re) optimized geometries in THF solution of the critical structures involved in the reaction between the complex [Re(PHPh)$(CO)_3$(bipy)] (bipy = 2,2'-bipyridine) and methyl propiolate (HMAD, HC≡CCO_2Me). Relevant distances are given in angstroms.

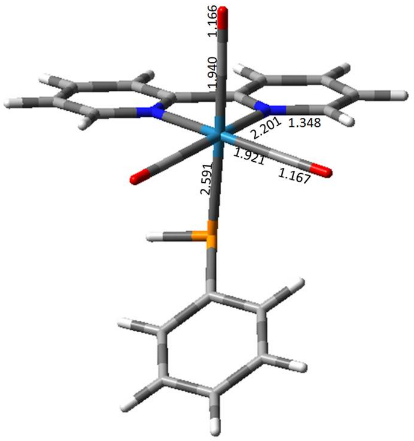

[Re(PHPh)$(CO)_3$(bipy)]

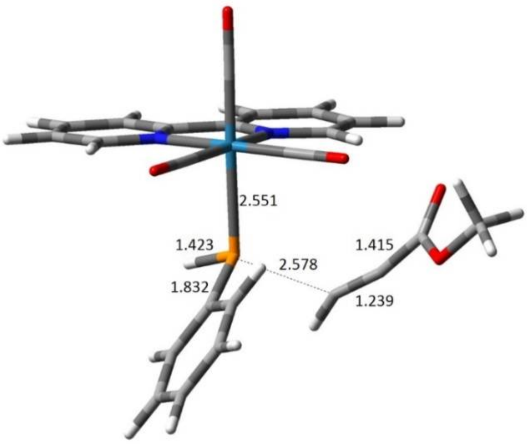

TS1

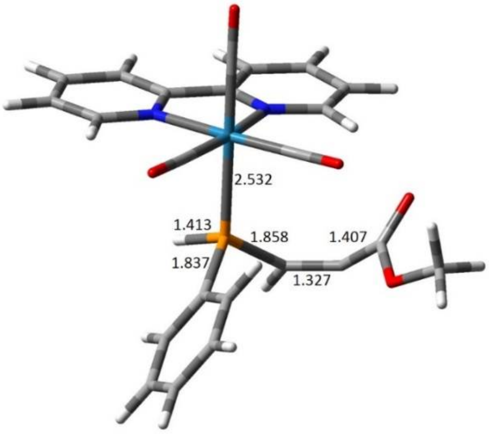

I1

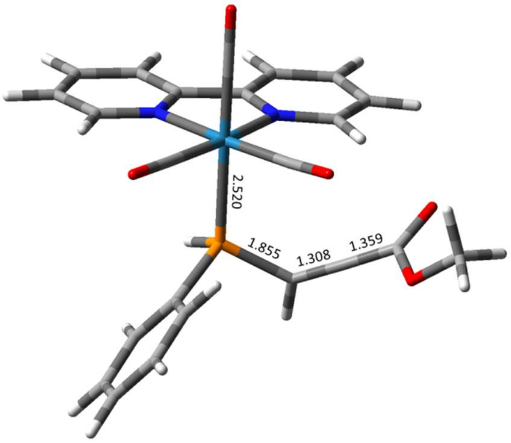

TS1'

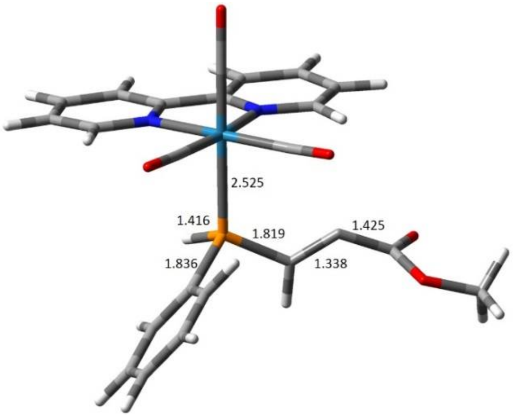

I1'

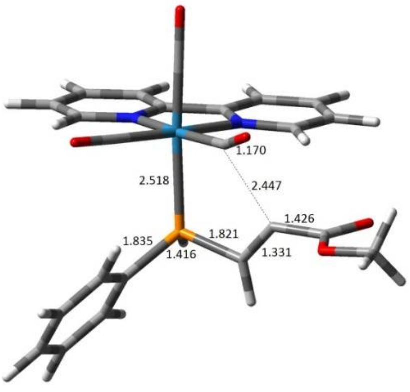

TS2*cco*

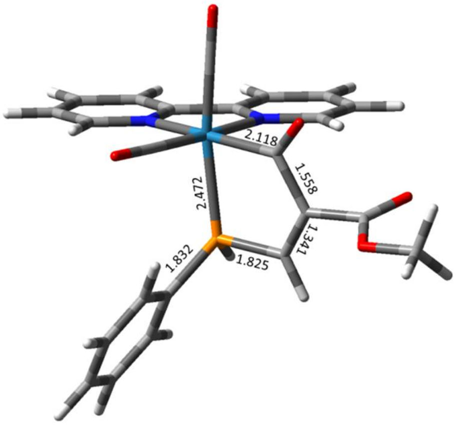

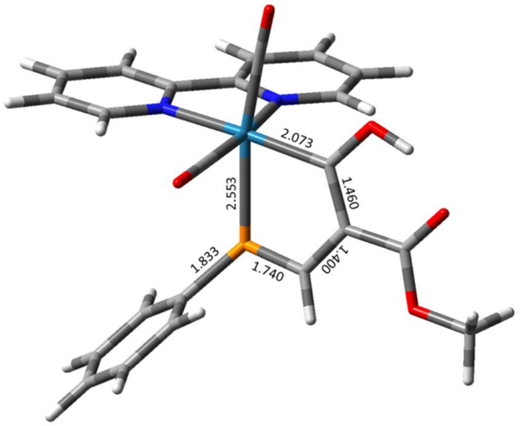

P*cco*

P*ccoh*

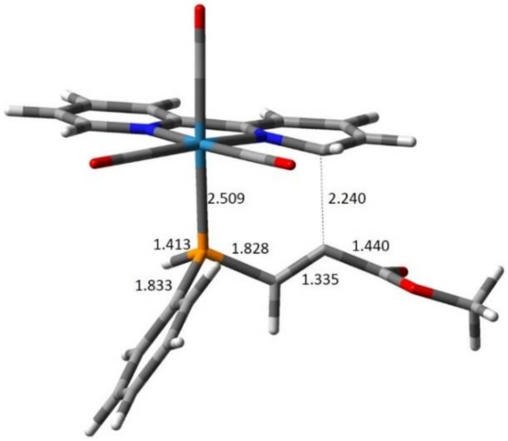

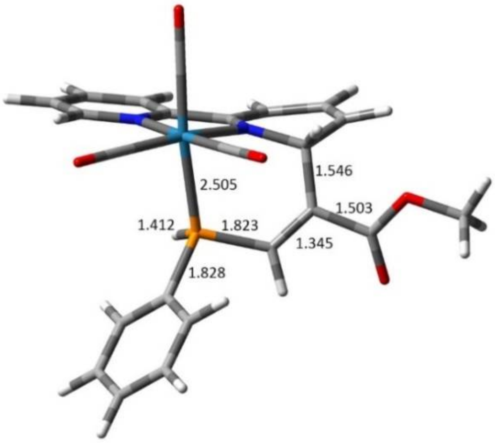

TS2*ccb*

P*ccb*

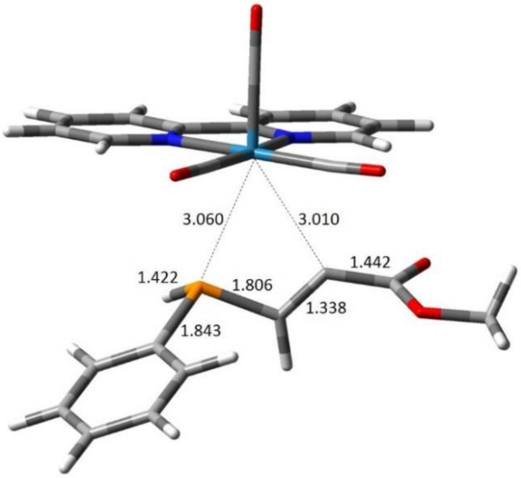

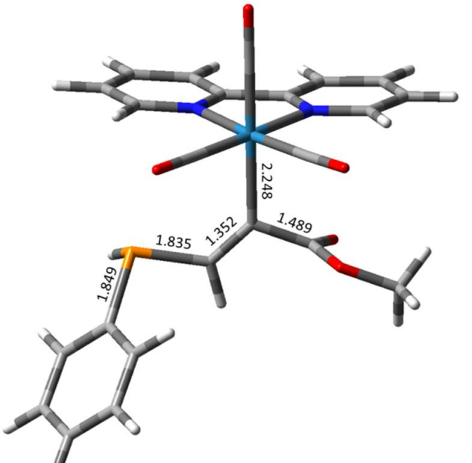

TS2*ins*

P*ins*

Table S22. PCM-B3LYP/6-31+G(d,p) (LANL2DZ for Re) energies without and with including thermal corrections (E and G, respectively). enthalpies (H), and entropies (S), and CPCM-DLPNO-CCSD(T)/def2-TZVPP//PCM-B3LYP/6-31+G(d,p) (LANL2DZ for Re) energies without and with including thermal corrections (E' and G', respectively) in THF solution of the critical structures involved in the reaction between the complex [Re(PMePh)(CO)$_3$(bipy)] (bipy = 2,2'-bipyridine) and methyl propiolate (HMAD, HC≡CCO$_2$Me). All the values are given in hartree, except entropies that are in cal/K mol.[a]

Species	E	H	S	G	E'	G'[b]
[Re(PMePh)(CO)$_3$(bipy)]	-1527.573697	-1527.229924	173.825	-1527.312514	-1524.201817	-1523.940634
HMAD	-305.215726	-305.136831	77.228	-305.173524	-304.738441	-304.696239
Reactants	-1832.789423	-1832.366755	251.053	-1832.486038	-1828.940258	-1828.636873
**TS1**	-1832.776582	-1832.352572	205.017	-1832.449982	-1828.933637	-1828.607037
**I1**	-1832.804632	-1832.379028	201.774	-1832.474897	-1828.966458	-1828.636723
**TS1'**	-1832.800227	-1832.375792	201.293	-1832.471433	-1828.959445	-1828.630651
**I1'**	-1832.806265	-1832.380441	204.218	-1832.477471	-1828.968119	-1828.639325
**TS2*cco***	-1832.802690	-1832.380031	200.229	-1832.475167	-1828.960832	-1828.633309
**P*cco***	-1832.828433	-1832.401548	201.675	-1832.497370	-1828.986408	-1828.655345
**TS2*ccb***	-1832.799072	-1832.376035	197.814	-1832.470023	-1828.960264	-1828.631215
**P*ccb***	-1832.828216	-1832.401869	199.010	-1832.496425	-1829.000484	-1828.668693
**TS2*ins***	-1832.779017	-1832.355020	201.643	-1832.450827	-1828.937727	-1828.609537
**P*ins***	-1832.830510	-1832.404727	208.881	-1832.503973	-1828.997050	-1828.670513

[a] Thermal magnitudes were computed in THF solution at 298.15 K and 1 atm. [b] For each species. G' was calculated as G' = G – E + E', in which G is the PCM-B3LYP/6-31+G(d,p) (LANL2DZ for Re) energy with including thermal corrections and E and E' are the PCM-B3LYP/6-31+G(d,p) (LANL2DZ for Re) and CPCM-DLPNO-CCSD(T)/def2-TZVPP//PCM-B3LYP/6-31+G(d,p) (LANL2DZ for Re) energies without including thermal corrections, respectively.

Table S23. PCM-B3LYP/6-31+G(d,p) (LANL2DZ for Re) relative energies without and with including thermal corrections (ΔE and ΔG, respectively), enthalpies (ΔH), and entropic contributions (TΔS), and CPCM-DLPNO-CCSD(T)/def2-TZVPP//PCM-B3LYP/6-31+G(d,p) (LANL2DZ for Re) relative energies without and with including thermal corrections (E' and G', respectively) in THF solution of the critical structures involved in the reaction between the complex [Re(PMePh)$(CO)_3$(bipy)] (bipy = 2,2'-bipyridine) and methyl propiolate (HMAD, $HC{\equiv}CCO_2Me$). All the values are given in kcal/mol.[a]

Species	ΔE	ΔH	TΔS	ΔG	ΔE'	ΔG'
[Re(PMePh)$(CO)_3$(bipy)] + HMAD	0.0	0.0	0.0	0.0	0.0	0.0
**TS1**	8.1	8.9	-13.7	22.6	4.2	18.7
**I1**	-9.5	-7.7	-14.7	7.0	-16.4	0.1
**TS1'**	-6.8	-5.7	-14.8	9.2	-12.0	3.9
**I1'**	-10.6	-8.6	-14.0	5.4	-17.5	-1.5
**TS2*cco***	-8.3	-8.3	-15.2	6.8	-12.9	2.2
**P*cco***	-24.5	-21.8	-14.7	-7.1	-29.0	-11.6
**TS2*ccb***	-6.1	-5.8	-15.9	10.0	-12.6	3.6
**P*ccb***	-24.3	-22.0	-15.5	-6.5	-37.8	-20.0
**TS2*ins***	6.5	7.4	-14.7	22.1	1.6	17.2
**P*ins***	-25.8	-23.8	-12.6	-11.3	-35.6	-21.1

[a] Thermal magnitudes were evaluated in THF solution at 298.15 K and 1 atm.

Table S24. PCM-B3LYP/6-31+G(d,p) (LANL2DZ for Re) optimized cartesian coordinates, in Å, for the critical structures involved in the reaction of the complex [Re(PMePh)(CO)$_3$(bipy)] (bipy = 2,2'-bipyridine) towards methyl propiolate (HMAD, HC≡CCO$_2$Me).

[Re(PMePh)(CO)$_3$(bipy)]

Re	-0.196033	-0.285721	0.063379
P	-0.263485	-0.450719	2.672590
N	1.967358	-0.000301	0.317811
N	0.656552	-2.308800	0.203442
C	-0.123010	-0.297507	-1.874888
O	-0.148525	-0.304738	-3.041326
C	-0.658672	1.578854	0.080871
O	-0.886699	2.722847	0.088445
C	-2.057617	-0.755125	0.114586
O	-3.178834	-1.066018	0.203278
C	2.574740	1.204113	0.340601
C	3.943898	1.353206	0.514368
H	4.377611	2.344768	0.519801
C	4.729328	0.209894	0.676189
H	5.801777	0.286033	0.809389
C	4.110420	-1.033716	0.663020
C	2.723102	-1.118248	0.489175
C	1.995054	-2.397362	0.435679
C	2.621139	-3.641993	0.579595
C	1.873681	-4.807965	0.470411
H	2.350344	-5.774579	0.579394
C	0.504716	-4.707760	0.213833
H	-0.119278	-5.586892	0.112863
C	-0.061581	-3.446429	0.092426
H	-1.119464	-3.324249	-0.098499
H	3.683273	-3.697781	0.775249
H	4.699839	-1.932110	0.785150
C	-1.403634	0.847090	3.325239
C	-0.951516	2.183815	3.345845
C	-2.672077	0.586164	3.878777
C	-1.731435	3.209677	3.877708
H	0.027735	2.419432	2.937788
C	-3.454407	1.612992	4.419042
H	-3.061741	-0.425149	3.889664
C	-2.992455	2.931072	4.419428
H	-1.356427	4.227979	3.873367
H	-4.428893	1.377937	4.836856
H	-3.600322	3.727492	4.836345
H	1.932467	2.064514	0.206105
C	-1.233096	-2.007983	3.053104
H	-1.368095	-2.108602	4.134125
H	-2.217899	-2.048462	2.575930
H	-0.650356	-2.865750	2.710747

TS1

C	-4.390235	-0.128456	-1.358525
C	-3.169653	0.156836	-0.735160
N	-2.494396	-0.802500	-0.045661
C	-3.023621	-2.040659	0.036412
C	-4.232298	-2.382892	-0.554793
C	-4.928537	-1.407343	-1.269633
C	-2.531835	1.485340	-0.775218
N	-1.351178	1.596703	-0.106171
C	-0.703279	2.778456	-0.121998
C	-1.199507	3.895720	-0.783856
C	-2.413734	3.795088	-1.460942
C	-3.082772	2.575266	-1.457397
Re	-0.595564	-0.216366	0.903385
P	0.576861	-0.724732	-1.341090
C	-0.074596	-1.966708	1.500204
O	0.217353	-3.048466	1.821342
C	1.054299	0.508999	1.583636
O	2.046667	0.960841	1.994872
C	-1.510834	0.160191	2.573839
O	-2.039475	0.331328	3.597910
C	1.738895	1.371122	-2.486975
C	2.388470	2.105324	-1.733003
C	2.943142	2.702844	-0.571187
O	2.409269	3.599476	0.085164
O	4.155707	2.183013	-0.253981
C	4.783095	2.722357	0.926668
H	1.368908	1.084851	-3.447038
H	4.156588	2.538137	1.799898
H	4.954194	3.794241	0.811041
H	5.729868	2.192701	1.013855
H	0.246039	2.829768	0.395201
H	-0.631469	4.816611	-0.765510
H	-2.830196	4.645088	-1.987993
H	-5.873929	-1.636914	-1.746298
H	-4.610737	-3.391779	-0.453274
H	-2.452570	-2.766459	0.599534
H	-4.023824	2.473064	-1.980807
H	-4.914689	0.641282	-1.908043
C	-0.518278	-1.817337	-2.388121
H	-0.868573	-2.714116	-1.867171
H	-1.393486	-1.240832	-2.695367
H	0.019430	-2.128667	-3.288036
C	2.028445	-1.825848	-1.047956
C	3.228009	-1.258012	-0.570042
C	2.020579	-3.206514	-1.319942
C	4.368393	-2.039093	-0.379926
H	3.279946	-0.194895	-0.360055
C	3.163987	-3.989295	-1.128155
H	1.118215	-3.685277	-1.683609
C	4.345329	-3.409981	-0.659704
H	5.280086	-1.572091	-0.020515
H	3.128673	-5.052272	-1.345812
H	5.233948	-4.015387	-0.516519

I1

C	3.398787	-1.050670	-0.316162
C	2.298518	-1.584757	-1.009701
C	2.358245	-2.910993	-1.463403
C	3.497056	-3.689790	-1.232901
C	4.585524	-3.153619	-0.543026
C	4.532895	-1.831497	-0.087944
P	0.814319	-0.523553	-1.270214
Re	-0.476562	-0.119243	0.883269
C	-1.513397	0.224851	2.495896
O	-2.111138	0.427548	3.469462
N	-2.215922	-1.165933	0.009448
C	-3.126779	-0.405162	-0.653342
C	-4.309049	-0.968537	-1.148016
C	-4.557007	-2.324480	-0.964794
C	-3.609645	-3.099106	-0.295068
C	-2.457530	-2.482336	0.173900
C	-2.781881	1.021506	-0.807961
N	-1.603008	1.414146	-0.248384
C	-1.209488	2.696143	-0.379897
C	-1.984352	3.645973	-1.041312
C	-3.200524	3.260829	-1.597372
C	-3.598456	1.931017	-1.485046
C	0.987342	0.913106	1.617606
O	1.861933	1.478575	2.130079
C	0.314189	-1.681884	1.676322
O	0.755810	-2.657116	2.134144
C	1.402180	0.916713	-2.283081
C	1.992505	2.042344	-1.905418
C	2.375359	2.730419	-0.747092
O	3.667614	2.435846	-0.338822
C	4.225518	3.317598	0.644777
O	1.732806	3.637814	-0.171082
H	1.232609	0.690740	-3.341869
H	4.243135	4.347706	0.280108
H	5.242433	2.963087	0.810306
H	3.655078	3.275945	1.574211
H	-0.241327	2.967091	0.028332
H	-1.620380	4.662310	-1.117296
H	-3.827519	3.975418	-2.117022

H	-5.471814	-2.768179	-1.339066
H	-3.754622	-4.159396	-0.129360
H	-1.702327	-3.044789	0.706780
H	1.525129	-3.348114	-2.001767
H	3.529779	-4.712071	-1.593740
H	5.468945	-3.757438	-0.365557
H	5.378264	-1.406188	0.442482
H	3.384679	-0.018355	0.021065
H	-5.030904	-0.354171	-1.667670
H	-4.533963	1.609586	-1.922366
C	-0.174812	-1.448015	-2.520468
H	-0.541225	-2.396545	-2.124991
H	-1.032837	-0.832731	-2.799634
H	0.429343	-1.634105	-3.411606

TS1′

C	3.031931	-1.881085	0.106420
C	2.115052	-2.007697	-0.951194
C	2.254566	-3.085359	-1.840524
C	3.292787	-4.008765	-1.679820
C	4.200474	-3.870583	-0.627587
C	4.069235	-2.800316	0.263448
P	0.833469	-0.701333	-1.167900
C	1.722790	0.676556	-2.034240
C	2.101243	1.792653	-1.467087
C	2.443448	2.961536	-0.866563
O	3.739437	2.945520	-0.331119
C	4.049856	4.019205	0.560187
Re	-0.444600	-0.047776	0.931221
C	1.010101	1.078615	1.549003
O	1.839019	1.752868	2.003010
N	-2.188238	-1.127077	0.108896
C	-3.051557	-0.411623	-0.660939
C	-4.211971	-1.000613	-1.176762
C	-4.489614	-2.335246	-0.902725
C	-3.595973	-3.061651	-0.115489
C	-2.462360	-2.421858	0.367880
C	-2.674243	0.992550	-0.918981
C	-3.453011	1.860587	-1.689154
C	-3.013371	3.163182	-1.913266
C	-1.796143	3.563066	-1.369542
C	-1.065917	2.658380	-0.603811
N	-1.497408	1.403625	-0.368287
C	0.287952	-1.522024	1.924361
O	0.696464	-2.438161	2.515952
C	-1.492048	0.483924	2.483379
O	-2.102173	0.783393	3.423939
O	1.729716	4.002103	-0.757073
H	1.918596	0.408519	-3.082529
H	3.858381	4.989897	0.098298
H	5.112220	3.915773	0.784666
H	3.468918	3.941334	1.484819
H	-0.102684	2.950990	-0.202374
H	-1.394832	4.554724	-1.530547
H	-3.609623	3.844685	-2.508152
H	-5.386591	-2.798978	-1.295196
H	-3.765255	-4.103680	0.124634
H	-1.747498	-2.947864	0.986257
H	-4.890540	-0.423258	-1.789134
H	-4.390543	1.527614	-2.113264
C	-0.253321	-1.375624	-2.494902
H	1.563956	-3.210235	-2.666386
H	3.388726	-4.834635	-2.376428
H	5.005522	-4.586591	-0.503462
H	4.770294	-2.683808	1.082544
H	2.941171	-1.054667	0.803624
H	0.313529	-1.520040	-3.417517
H	-0.702642	-2.322586	-2.189186
H	-1.047774	-0.652491	-2.688242

I1′

P	-0.831487	0.680263	-1.015729
C	-1.715209	2.296457	-0.909913
C	-1.650145	3.278213	-1.909056
C	-2.383527	4.464280	-1.788422
C	-3.193309	4.679371	-0.671963
C	-3.259701	3.707378	0.331954
C	-2.526921	2.527060	0.213733
Re	0.703659	0.207432	0.957297
C	0.568394	2.030100	1.552899
O	0.518645	3.142806	1.893881
N	1.090537	-1.767551	0.039993
C	0.370442	-2.872305	0.314915
C	0.696335	-4.121191	-0.205927
C	1.798078	-4.232740	-1.050293
C	2.533809	-3.089299	-1.352307
C	2.160497	-1.863342	-0.793918
C	2.886056	-0.604348	-1.055996
N	2.424947	0.493548	-0.398738
C	3.040399	1.677619	-0.591368
C	4.131219	1.828400	-1.437793
C	4.608401	0.709080	-2.119716
C	3.977556	-0.515241	-1.927639
C	1.880708	-0.207405	2.449664
O	2.554866	-0.447336	3.364090
C	-0.827847	-0.250272	2.053776
O	-1.703454	-0.499004	2.774283
C	-2.151344	-0.540658	-1.300449
C	-2.413962	-1.539022	-0.450109
C	-3.393188	-2.553234	-0.653968
O	-4.604648	-2.264647	-0.059583
C	-5.574807	-3.320224	-0.055906
O	-3.206028	-3.659055	-1.191162
H	-2.686974	-0.353923	-2.243295
H	-5.871124	-3.586632	-1.073163
H	-6.431872	-2.929571	0.491405
H	-5.183536	-4.209573	0.443095
H	-0.503581	-2.725655	0.933396
H	0.086353	-4.978452	0.047375
H	2.079801	-5.189127	-1.474166
H	5.454600	0.787943	-2.791022
H	4.588308	2.802594	-1.554714
H	2.638970	2.518569	-0.041436
H	-1.033615	3.129048	-2.787792
H	-2.324567	5.213243	-2.571896
H	-3.765329	5.596497	-0.581067
H	-3.884290	3.866132	1.204045
H	-2.590768	1.777459	0.997124
H	4.329912	-1.392874	-2.452035
H	3.389752	-3.155185	-2.009147
C	0.045746	0.764003	-2.634651
H	0.788466	1.565053	-2.643698
H	0.553611	-0.190336	-2.790167
H	-0.665766	0.913259	-3.450297

TS2*cco*

C	2.502035	-3.005025	-1.768322
C	2.096947	-1.938314	-0.959625
N	0.845303	-1.896721	-0.429019
C	-0.019824	-2.891406	-0.708835
C	0.325538	-3.978940	-1.504361
C	1.611552	-4.039415	-2.038953
C	2.969625	-0.789549	-0.644908
N	2.417370	0.181122	0.132193
C	3.155321	1.268877	0.435733
C	4.461239	1.439337	-0.006272
C	5.041339	0.440767	-0.789416
C	4.285283	-0.681321	-1.110558
Re	0.392897	-0.196806	0.927434
C	-1.424679	-0.762640	1.493924
O	-2.296453	-1.103790	2.194472
P	-0.578094	0.965956	-1.102557
C	-2.185062	0.196813	-1.474179
C	-2.603783	-0.782323	-0.674737
C	-3.738319	-1.637950	-0.759133
O	-4.809491	-1.188992	-0.028739
C	-5.910063	-2.104086	0.092690
C	-0.880189	2.781098	-0.997421
C	-0.208781	3.718327	-1.798612
C	-0.470320	5.086020	-1.663450
C	-1.410372	5.532464	-0.732294
C	-2.086539	4.605875	0.068848
C	-1.820053	3.242558	-0.059353
C	0.400521	0.782560	-2.655437
C	0.241988	1.379680	1.998262
O	0.194163	2.350345	2.647854
C	1.110472	-1.142337	2.452399
O	1.536118	-1.686087	3.391071
O	-3.757647	-2.755908	-1.300186
H	-2.698100	0.561824	-2.370942
H	-6.340765	-2.326341	-0.886291
H	-6.642357	-1.600870	0.722841
H	-5.585674	-3.038337	0.557228
H	1.916761	-4.871634	-2.661898
H	6.060761	0.532784	-1.143493
H	5.004961	2.333496	0.270919

H	2.673036	2.012912	1.055665
H	0.518768	3.394277	-2.534263
H	0.058421	5.797319	-2.289364
H	-1.614835	6.593009	-0.629382
H	-2.819389	4.944635	0.793266
H	-2.347570	2.530849	0.568276
H	4.715380	-1.467894	-1.715366
H	3.499730	-3.027144	-2.185635
H	-0.406831	-4.751866	-1.697582
H	-1.017871	-2.778840	-0.303755
H	-0.126509	1.230829	-3.500593
H	1.385649	1.243665	-2.554258
H	0.535083	-0.283312	-2.848518

P*cco*

Re	0.419046	-0.264335	0.853806
P	-0.684532	0.905178	-1.045766
N	1.374842	-1.605854	-0.644117
N	2.408939	0.598995	0.414210
C	0.983641	-1.386139	2.302620
O	1.283162	-2.078115	3.198856
C	-1.425853	-1.305630	0.847039
O	-1.874014	-2.119482	1.673870
C	-0.203733	1.002930	2.120754
O	-0.545374	1.800159	2.918045
C	-2.190911	-0.104110	-1.250375
H	-2.871777	0.087123	-2.075792
C	-2.376862	-1.092855	-0.365145
C	-3.502992	-2.068055	-0.502511
O	-3.350564	-3.207260	-0.910578
O	-4.685011	-1.562544	-0.120354
C	-5.810761	-2.469064	-0.171802
H	-5.951212	-2.838153	-1.188072
H	-6.668381	-1.883142	0.149472
H	-5.641453	-3.308397	0.503457
C	1.437444	-3.581696	-2.011501
C	2.723291	-3.257283	-2.445479
H	3.253110	-3.897523	-3.140676
C	3.320595	-2.095834	-1.967594
C	2.628138	-1.280553	-1.063338
C	3.195712	-0.045386	-0.490935
C	4.463017	0.443177	-0.834379
C	4.940969	1.602023	-0.233951
H	5.920692	1.988147	-0.487730
C	4.133425	2.253364	0.700866
H	4.460408	3.156360	1.200946
C	2.882800	1.724572	0.990969
H	2.224899	2.197733	1.708376
C	-1.324255	2.625639	-0.923986
C	-0.975019	3.626303	-1.843491
H	-0.311348	3.407703	-2.672001
C	-1.482323	4.922133	-1.704889
H	-1.203291	5.685608	-2.423048
C	-2.350241	5.228367	-0.654949
H	-2.747625	6.232174	-0.551466
C	-2.702473	4.236368	0.265966
H	-3.375347	4.466754	1.084794
C	-2.190681	2.944955	0.135514
H	-2.463273	2.185463	0.860267
H	5.070295	-0.078173	-1.562096
H	4.319054	-1.831396	-2.288823
C	0.799851	-2.730834	-1.117137
H	0.931089	-4.475687	-2.352013
H	-0.198274	-2.945322	-0.756782
C	0.074180	0.824073	-2.724384
H	-0.641535	1.122520	-3.493365
H	0.953046	1.469236	-2.778573
H	0.386637	-0.206395	-2.902489

TS2*ccb*

C	0.149076	3.619913	-2.087438
C	-0.313636	2.876700	-0.990983
C	-0.785116	3.562033	0.142346
C	-0.802563	4.956981	0.171502
C	-0.338032	5.689177	-0.926289
C	0.138523	5.018023	-2.053337
P	-0.329388	1.037507	-0.984816
C	-2.109673	0.633674	-1.092569
C	-2.682577	-0.396919	-0.469240
C	-4.053088	-0.811983	-0.570882
O	-4.856254	-0.265431	0.394294
C	-6.203963	-0.766351	0.440820
Re	0.896615	-0.047177	0.941239
C	-0.416069	0.639202	2.171778
O	-1.219954	1.032165	2.917250
N	-0.221386	-1.811898	0.280604
C	0.342627	-2.592239	-0.685868
C	-0.319803	-3.686541	-1.226327
C	-1.595646	-4.021478	-0.721810
C	-2.187216	-3.215021	0.218026
C	-1.534026	-2.009401	0.623862
C	1.713362	-2.211169	-1.081757
N	2.194028	-1.062266	-0.533238
C	3.431168	-0.638644	-0.865622
C	4.249211	-1.330504	-1.746968
C	3.773150	-2.518568	-2.305979
C	2.499502	-2.959747	-1.968488
C	1.988867	1.516192	1.224494
O	2.664571	2.454601	1.370329
C	1.800591	-0.981682	2.394414
O	2.337516	-1.512944	3.275811
C	0.311395	0.579284	-2.650212
O	-4.472753	-1.687607	-1.334963
H	-1.847959	-1.523478	1.534516
H	-3.166708	-3.438370	0.619992
H	-2.095544	-4.917323	-1.072599
H	0.143933	-4.297560	-1.987988
H	3.761639	0.279651	-0.398234
H	5.233494	-0.945135	-1.979337
H	4.386068	-3.093084	-2.990229
H	2.117418	-3.881830	-2.384995
H	0.520145	3.120469	-2.974554
H	0.501854	5.577027	-2.908998
H	-0.347768	6.772902	-0.901834
H	-1.172853	5.470781	1.052137
H	-1.141035	3.008149	1.004936
H	-2.655847	1.318880	-1.751537
H	-6.684643	-0.236151	1.261134
H	-6.723824	-0.564466	-0.497939
H	-6.208539	-1.842284	0.627317
H	-0.282997	1.051924	-3.435014
H	1.359795	0.866107	-2.755999
H	0.228171	-0.503235	-2.755674

P*ccb*

Re	0.935577	0.222960	0.932727
P	-0.504838	0.945102	-0.988128
N	0.167456	-1.662159	0.180321
N	2.416381	-0.461427	-0.552366
C	2.005906	-0.551681	2.362042
O	2.641896	-1.000320	3.224191
C	-0.470268	0.619475	2.185790
O	-1.326360	0.842479	2.946257
C	1.709896	1.966109	1.225128
O	2.200738	3.018262	1.352029
C	-2.014402	-0.078340	-1.056701
H	-2.841518	0.360534	-1.611032
C	-2.229275	-1.256013	-0.443252
C	-3.639457	-1.771779	-0.533158
O	-4.365675	-1.661115	-1.507019
O	-4.031839	-2.341055	0.619276
C	-5.381859	-2.855165	0.651793
H	-6.092751	-2.058569	0.430411
H	-5.523274	-3.230680	1.661804
H	-5.492888	-3.658300	-0.077922
C	-1.221413	-2.077476	0.398105
H	-1.480746	-1.854700	1.440685
C	-1.384130	-3.573063	0.178305
H	-2.160495	-4.088577	0.729888
C	-0.584566	-4.213182	-0.706837
H	-0.731135	-5.270726	-0.910003
C	0.522923	-3.534475	-1.313665
C	0.891692	-2.311773	-0.776606
C	2.162964	-1.658699	-1.148676
C	3.082485	-2.196610	-2.064088
C	4.246263	-1.505927	-2.368849
H	4.956224	-1.918534	-3.076214
C	4.490417	-0.275261	-1.748489
H	5.385519	0.298268	-1.952543
C	3.553575	0.205522	-0.848149
H	3.698686	1.149061	-0.337267
H	2.879592	-3.151118	-2.529686
H	1.131043	-4.031060	-2.055436
C	-1.205503	2.643492	-0.959674
C	-2.103812	2.991341	0.063490
C	-0.839575	3.614224	-1.903545

C	-2.631223	4.281304	0.132202
H	-2.391120	2.258011	0.809764
C	-1.363748	4.908319	-1.828026
H	-0.147375	3.371016	-2.701109
C	-2.262930	5.243286	-0.813928
H	-3.328331	4.534903	0.923346
H	-1.071641	5.648522	-2.564966
H	-2.672096	6.246224	-0.757178
C	0.201585	0.772505	-2.680727
H	-0.525921	1.084553	-3.432371
H	1.109799	1.371834	-2.771128
H	0.452535	-0.276929	-2.840120

TS2*ins*

Re	0.778554	-0.469847	-1.051184
C	1.674440	-1.943125	-1.815128
O	2.229730	-2.848532	-2.309999
C	0.006410	-0.078105	-2.759808
O	-0.494481	0.112129	-3.797033
C	2.264902	0.699453	-1.441441
O	3.190000	1.358619	-1.690054
N	1.409698	-1.178193	0.974663
C	2.597612	-0.875452	1.529632
C	3.013147	-1.396299	2.748776
C	2.159568	-2.258370	3.434788
C	0.927270	-2.571819	2.871296
C	0.576137	-2.024986	1.632052
N	-0.865356	-1.748968	-0.267551
C	-2.002177	-1.991554	-0.950706
C	-3.013042	-2.808060	-0.463571
C	-2.847397	-3.406645	0.786363
C	-1.678390	-3.162663	1.497275
C	-0.694518	-2.328820	0.949168
P	-1.367378	1.609834	-0.482598
C	-3.040428	1.554639	0.309496
C	-3.306331	0.523998	1.228612
C	-4.547110	0.427838	1.862078
C	-5.555642	1.351861	1.572691
C	-5.309119	2.376133	0.655123
C	-4.064713	2.474082	0.025771
C	-0.255572	2.445589	0.677072
C	0.978343	1.934921	0.727602
C	2.078565	2.429104	1.512435
O	2.402753	2.024861	2.636362
O	2.853573	3.332025	0.836046
C	4.049219	3.762921	1.506878
H	3.220642	-0.188070	0.975640
H	3.979351	-1.117855	3.148668
H	2.444852	-2.680008	4.391139
H	0.250249	-3.238473	3.387596
H	-2.090837	-1.504763	-1.913318
H	-3.905792	-2.963782	-1.055478
H	-3.613859	-4.050772	1.200322
H	-1.530051	-3.618398	2.466746
H	-2.536939	-0.208535	1.453544
H	-4.729465	-0.372968	2.570680
H	-6.522524	1.275081	2.059428
H	-6.084146	3.099655	0.424733
H	-3.897553	3.279580	-0.680014
H	-0.605876	3.324545	1.228280
H	3.807064	4.266649	2.444788
H	4.701543	2.912930	1.717866
H	4.537030	4.454009	0.821381
C	-1.614321	2.838842	-1.849023
H	-2.382300	2.487367	-2.542450
H	-1.895491	3.823480	-1.467833
H	-0.670852	2.927801	-2.390879

P*ins*

C	-5.364553	1.069149	-0.585870
C	-4.154775	0.360947	-0.465570
C	-4.212661	-0.982369	-0.054646
C	-5.436237	-1.595839	0.229063
C	-6.629861	-0.879106	0.108291
C	-6.588512	0.456290	-0.304129
P	-2.523505	1.221736	-0.668881
C	-1.509277	-0.112521	-1.409665
C	-0.380390	-0.653398	-0.899485
C	0.217246	-1.699723	-1.774516
O	-0.099525	-2.955291	-1.377229
C	0.454566	-4.033582	-2.157711
Re	0.775635	-0.259580	0.988731
C	-0.843257	0.004740	1.994587
O	-1.806622	0.164044	2.630148
N	2.579121	-0.338055	-0.290047
C	3.313492	-1.448818	-0.490560
C	4.444944	-1.468481	-1.296522
C	4.830814	-0.291581	-1.936129
C	4.070331	0.857080	-1.742724
C	2.949517	0.813774	-0.906915
C	2.128136	2.002272	-0.602464
N	1.081636	1.801741	0.240765
C	0.320098	2.853369	0.597503
C	0.558704	4.143256	0.135033
C	1.625042	4.357246	-0.736611
C	2.416738	3.274690	-1.107491
C	1.845857	0.138980	2.572382
O	2.469511	0.365887	3.529695
C	0.731160	-2.121791	1.463245
O	0.751541	-3.251537	1.751568
O	0.946969	-1.491054	-2.736688
H	2.979256	-2.340789	0.023244
H	5.003146	-2.388621	-1.413272
H	5.704950	-0.265759	-2.574797
H	4.352532	1.780781	-2.229133
H	3.252547	3.419190	-1.777875
H	1.840270	5.347418	-1.119463
H	-0.083024	4.953139	0.457899
H	-0.503789	2.640472	1.263840
H	-1.887987	-0.469242	-2.375132
C	-2.866077	2.219751	-2.206369
H	-3.293305	-1.551617	0.038418
H	-5.454671	-2.634352	0.542183
H	-7.579482	-1.354308	0.330641
H	-7.508454	1.023024	-0.405936
H	-5.360958	2.107888	-0.899891
H	0.068565	-4.002524	-3.177841
H	0.136734	-4.946113	-1.657899
H	1.542963	-3.967251	-2.182510
H	-3.623324	2.979227	-2.000853
H	-1.947495	2.732627	-2.499578
H	-3.205090	1.591074	-3.035041

Figure S8. PCM-B3LYP/6-31+G(d,p) (LANL2DZ for Re) optimized geometries in THF solution of the critical structures involved in the reaction between the complex [Re(PMePh)(CO)$_3$(bipy)] (bipy = 2,2'-bipyridine) and methyl propiolate (HMAD, HC≡CCO$_2$Me). Relevant distances are given in angstroms.

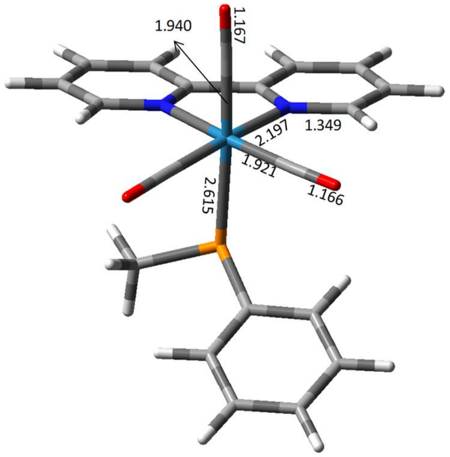

[Re(PMePh)(CO)$_3$(bipy)]

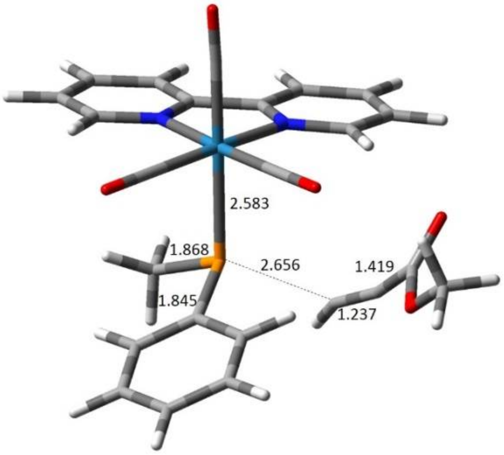

TS1

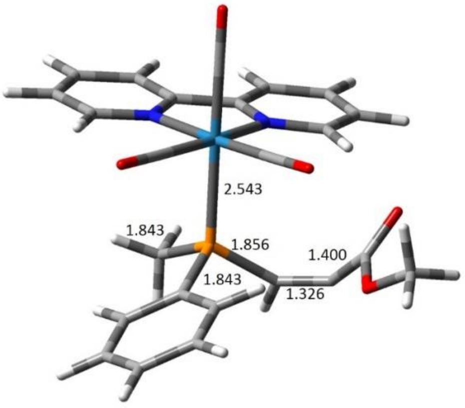

I1

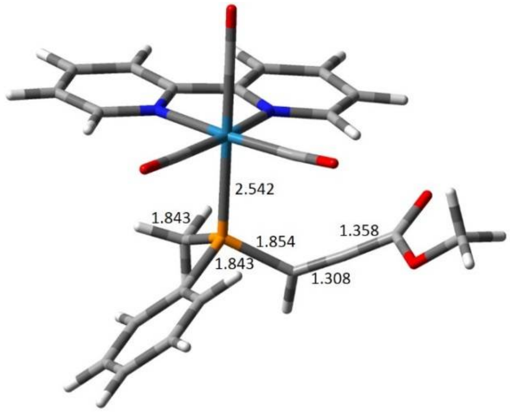

TS1'

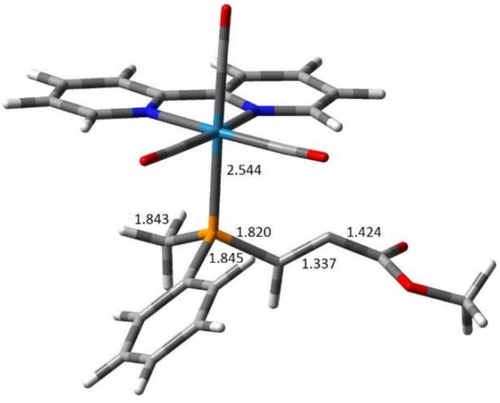

I1'

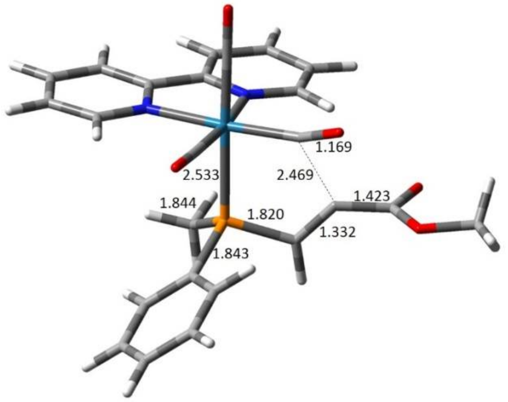

TS2*cco*

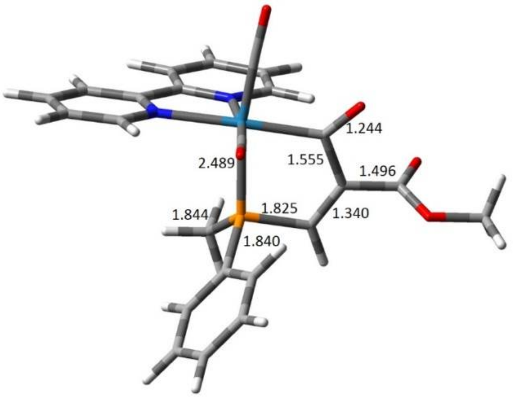

P*cco*

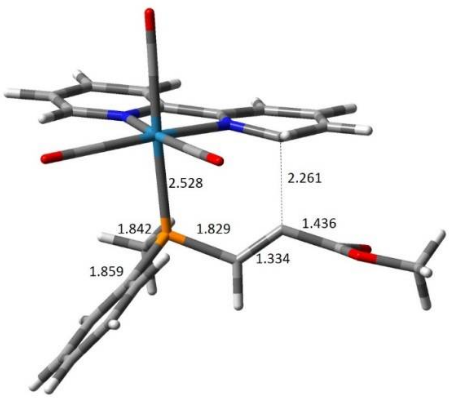

TS2*ccb*

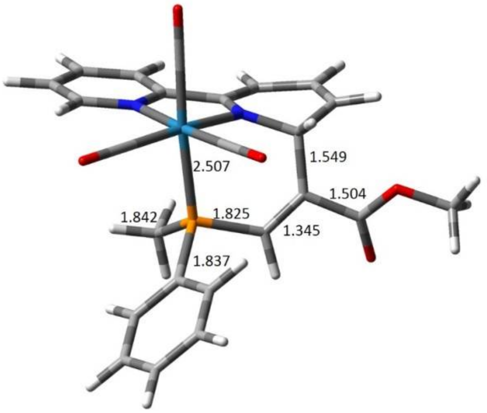

P*ccb*

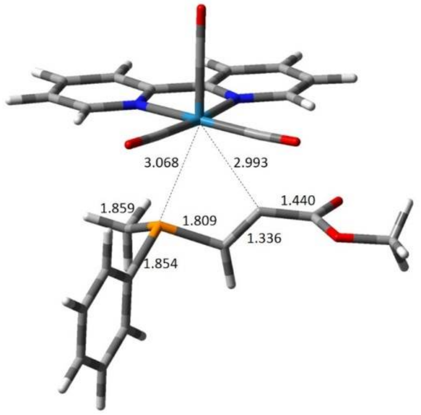

TS2*ins*

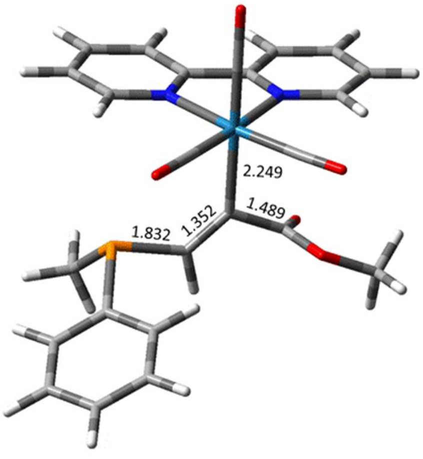

P*ins*

Table S25. PCM-B3LYP/6-31+G(d,p) (LANL2DZ for Re) energies without and with including thermal corrections (E and G, respectively), enthalpies (H), and entropies (S), and CPCM-DLPNO-CCSD(T)/def2-TZVPP//PCM-B3LYP/6-31+G(d,p) (LANL2DZ for Re) energies without and with including thermal corrections (E' and G', respectively) in THF solution of the critical structures involved in the reaction between the complex [Re(SH)$(CO)_3$(bipy)] (bipy = 2,2'-bipyridine) and methyl propiolate (HMAD, HC≡CCO_2Me). All the values are given in hartree, except entropies that are in cal/K mol.[a]

Species	E	H	S	G	E'	G'[b]
[Re(SH)$(CO)_3$(bipy)]	-1313.467257	-1313.250858	145.026	-1313.319764	-1310.591473	-1310.443980
HMAD	-305.215726	-305.136831	77.228	-305.173524	-304.738441	-304.696239
Reactants	-1618.682983	-1618.387689	222.254	-1618.493288	-1615.329914	-1615.140219
**TS1'**	-1618.652647	-1618.356456	176.221	-1618.440186	-1615.300544	-1615.088083
**I1'**	-1618.655485	-1618.357512	178.263	-1618.442211	-1615.305911	-1615.092637
**TS2*cco***	-1618.653722	-1618.357617	174.152	-1618.440362	-1615.301125	-1615.087765
**P*cco***	-1618.675783	-1618.376854	171.881	-1618.458520	-1615.323858	-1615.106595
**P*ccoh***	-1618.730922	-1618.428460	166.589	-1618.507612	-1615.367638	-1615.144328
**TS1*b***	-1618.655810	-1618.359516	174.346	-1618.442353	-1615.309011	-1615.095554
**I1*b***	-1618.656625	-1618.358850	176.536	-1618.442728	-1615.313116	-1615.099219
**I1'*b***	-1618.655995	-1618.357948	175.216	-1618.441199	-1615.309394	-1615.094598
**TS2*ccb***	-1618.646343	-1618.349882	172.254	-1618.431725	-1615.297809	-1615.083191
**P*ccb***	-1618.675067	-1618.376194	169.619	-1618.456786	-1615.336701	-1615.118420
**TS2*ins***	-1618.642618	-1618.346102	173.496	-1618.428536	-1615.291930	-1615.077848
**P*ins***	-1618.699194	-1618.400273	173.759	-1618.482831	-1615.360353	-1615.143990

[a] Thermal magnitudes were computed in THF solution at 298.15 K and 1 atm. [b] For each species, G' was calculated as G' = G – E + E', in which G is the PCM-B3LYP/6-31+G(d,p) (LANL2DZ for Re) energy with including thermal corrections and E and E' are the PCM-B3LYP/6-31+G(d,p) (LANL2DZ for Re) and CPCM-DLPNO-CCSD(T)/def2-TZVPP//PCM-B3LYP/6-31+G(d,p) (LANL2DZ for Re) energies without including thermal corrections, respectively.

Table S26. PCM-B3LYP/6-31+G(d,p) (LANL2DZ for Re) relative energies without and with including thermal corrections (ΔE and ΔG, respectively), enthalpies (ΔH), and entropic contributions (TΔS), and CPCM-DLPNO-CCSD(T)/def2-TZVPP//PCM-B3LYP/6-31+G(d,p) (LANL2DZ for Re) relative energies without and with including thermal corrections (E' and G', respectively) in THF solution of the critical structures involved in the reaction between the complex $[Re(SH)(CO)_3(bipy)]$ (bipy = 2,2'-bipyridine) and methyl propiolate (HMAD, $HC{\equiv}CCO_2Me$). All the values are given in kcal/mol.[a]

Species	ΔE	ΔH	TΔS	ΔG	ΔE'	ΔG'
$[Re(SH)(CO)_3(bipy)]$ + HMAD	0.0	0.0	0.0	0.0	0.0	0.0
**TS1**	19.0	19.6	-13.7	33.3	18.4	32.7
**I1**	17.3	18.9	-13.1	32.1	15.1	29.9
**TS2*cco***	18.4	18.9	-14.3	33.2	18.1	32.9
**P*cco***	4.5	6.8	-15.0	21.8	3.8	21.1
**P*ccoh***	-30.1	-25.6	-16.6	-9.0	-23.7	-2.6
**TS1*b***	17.1	17.7	-14.3	32.0	13.1	28.0
**I1*b***	16.5	18.1	-13.6	31.7	10.5	25.7
**I1'*b***	16.9	18.7	-14.0	32.7	12.9	28.6
**TS2*ccb***	23.0	23.7	-14.9	38.6	20.1	35.8
**P*ccb***	5.0	7.2	-15.7	22.9	-4.3	13.7
**TS2*ins***	25.3	26.1	-14.5	40.6	23.8	39.1
**P*ins***	-10.2	-7.9	-14.5	6.6	-19.1	-2.4

[a] Thermal magnitudes were evaluated in THF solution at 298.15 K and 1 atm.

Table S27. PCM-B3LYP/6-31+G(d,p) (LANL2DZ for Re) optimized cartesian coordinates, in Å, for the critical structures involved in the reaction of the complex [Re(SH)$(CO)_3$(bipy)] (bipy = 2,2'-bipyridine) towards methyl propiolate (HMAD, HC≡CCO_2Me).

[Re(SH)$(CO)_3$(bipy)]

Re	0.878217	0.003129	-0.045953
C	1.111580	-0.076551	1.863876
O	1.275773	-0.127922	3.018624
C	2.226971	1.362471	-0.202432
O	3.024968	2.205564	-0.301976
C	2.223691	-1.339347	-0.326466
O	3.016838	-2.171567	-0.514459
C	-2.110227	0.736140	0.056584
C	-0.814916	2.673606	0.096981
C	-3.282339	1.499153	0.029985
C	-1.941455	3.487596	0.081658
H	0.180392	3.097620	0.117193
C	-3.199649	2.887805	0.041645
H	-4.249222	1.015791	-0.009454
H	-1.824534	4.563455	0.094197
H	-4.101272	3.487244	0.017663
C	-2.108651	-0.740447	0.053174
C	-0.808889	-2.674688	0.004675
C	-3.278572	-1.506500	0.095667
C	-1.932747	-3.491680	0.038420
H	0.187098	-3.096241	-0.031140
C	-3.192396	-2.895017	0.088205
H	-4.246283	-1.025200	0.139765
H	-1.813050	-4.567231	0.027893
H	-4.092331	-3.496498	0.120534
N	-0.885941	-1.330627	0.015539
N	-0.888289	1.329273	0.079541
H	1.661234	0.094086	-3.045030
S	0.409081	0.102252	-2.551868

TS1

C	-1.500540	3.443108	0.339597
C	-1.420828	2.054504	0.202146
N	-0.253042	1.392410	0.428399
C	0.848703	2.096851	0.755214
C	0.829519	3.479524	0.907789
C	-0.366912	4.163376	0.707211
C	-2.574523	1.218730	-0.190495
N	-2.346513	-0.119780	-0.253212
C	-3.354198	-0.942679	-0.604434
C	-4.628454	-0.479750	-0.907113
C	-4.872073	0.892081	-0.850466
C	-3.835633	1.746659	-0.489179
Re	-0.322904	-0.812949	0.273785
S	0.144286	-0.369113	-2.214630
H	0.384969	-1.606727	-2.690616
C	-0.823165	-1.040123	2.113329
O	-1.131539	-1.187496	3.225569
C	1.519556	-1.206677	0.774579
O	2.562220	-1.494842	1.196441
C	-0.616094	-2.682595	-0.054639
O	-0.832280	-3.805778	-0.276027
C	1.932778	0.333246	-2.400110
C	2.718921	0.418134	-1.383661
C	3.866959	0.731461	-0.681250
O	4.061565	1.757212	0.009933
O	4.799421	-0.292473	-0.710262
C	5.844170	-0.210537	0.265418
H	2.029443	0.634217	-3.440276
H	6.376549	0.739973	0.199014
H	6.519381	-1.036148	0.042504
H	5.434360	-0.326948	1.272741
H	1.771277	1.545028	0.875451
H	1.742990	3.994007	1.175470
H	-0.419374	5.239152	0.824527
H	-5.851726	1.292010	-1.082149
H	-5.403214	-1.183955	-1.181665
H	-3.119487	-1.998362	-0.637607
H	-4.007451	2.813214	-0.441779
H	-2.435779	3.958229	0.169438

I1

Re	-0.507457	-0.931903	0.206789
C	-0.865185	-2.759682	-0.267154
O	-1.122979	-3.851414	-0.580960
N	-0.361382	1.250963	0.519867
C	0.706843	1.860010	1.069487
C	0.733797	3.227535	1.323599
C	-0.375830	3.996502	0.981028
C	-1.472377	3.374737	0.389388
C	-1.443937	1.994723	0.168245
C	-2.564073	1.252987	-0.446722
N	-2.396645	-0.091465	-0.559527
C	-3.379751	-0.831178	-1.109446
C	-4.569429	-0.277282	-1.564901
C	-4.750601	1.100732	-1.453960
C	-3.737135	1.870778	-0.892589
C	-1.260031	-1.220693	1.950155
C	-1.712579	-1.408855	3.004953
C	1.232412	-1.469641	0.886659
O	2.222694	-1.847270	1.356971
C	2.177584	0.007235	-2.131704
C	2.772665	0.439590	-1.039767
C	4.113832	0.916057	-0.965053
C	5.010747	-0.070954	-0.618006
C	6.352266	0.360887	-0.351542
O	4.486313	2.094872	-1.074945
H	2.550370	-0.077024	-3.153897
H	6.816175	0.779332	-1.247495
H	6.891109	-0.531188	-0.034830
H	6.369133	1.113458	0.440135
H	1.558643	1.227505	1.273515
H	1.613606	3.668062	1.774600
H	-0.390431	5.064667	1.163173
H	-5.664315	1.569564	-1.798663
H	-5.327903	-0.918548	-1.994784
H	-3.198262	-1.895695	-1.175183
H	-3.858177	2.941190	-0.802324
H	-2.339487	3.957772	0.111008
H	0.462399	-1.655303	-2.699813
S	0.373441	-0.424840	-2.156142

TS2*cco*

C	0.543734	2.154224	0.743445
N	-0.466660	1.368519	0.322144
C	-1.674316	1.932263	0.049998
C	-1.880248	3.306398	0.207261
C	-0.838454	4.109911	0.660969
C	0.395445	3.523487	0.935868
Re	-0.313118	-0.829917	0.056079
C	-0.406412	-2.693141	-0.365183
O	-0.504938	-3.822573	-0.648505
C	-2.731557	1.008078	-0.409116
N	-2.374718	-0.299664	-0.513740
C	-3.296189	-1.197794	-0.916512
C	-4.602522	-0.845130	-1.230234
C	-4.977067	0.494432	-1.127418
C	-4.031768	1.426949	-0.713487
C	-0.818000	-1.214917	1.856085
O	-1.119914	-1.462666	2.956146
C	1.589407	-1.033961	0.596457
C	2.651067	0.334318	-1.092726
C	3.944327	0.824890	-0.736132
O	4.900049	-0.153867	-0.723588
C	6.190305	0.232414	-0.221723
C	2.054421	0.310326	-2.265164
O	2.517023	-1.369023	1.224099
O	4.193285	1.972202	-0.341231
H	2.412736	0.646084	-3.236717
H	6.633323	1.009703	-0.847611
H	6.797245	-0.670338	-0.255548
H	6.112273	0.599607	0.803183
H	-0.988888	5.175059	0.791169
H	-5.986785	0.809368	-1.360852
H	-5.302277	-1.608185	-1.546215
H	-2.962062	-2.224887	-0.977331
H	-4.306022	2.469174	-0.624175
H	-2.841548	3.745664	-0.020016
H	1.238690	4.106011	1.283581
H	1.496396	1.665500	0.902554
H	0.428041	-1.399914	-2.979519
S	0.290430	-0.206606	-2.364427

P*cco*

C	-1.985936	3.566232	-0.238013
C	-1.765575	2.186616	-0.148588
N	-0.553592	1.692648	0.226279
C	0.440179	2.557773	0.516702
C	0.280221	3.936959	0.452419
C	-0.957431	4.451345	0.067120
C	-2.812028	1.185267	-0.435527
C	-4.121008	1.530506	-0.794387
C	-5.058842	0.530906	-1.026132
C	-4.665071	-0.801922	-0.894578
C	-3.352088	-1.079014	-0.535338
N	-2.436342	-0.115074	-0.304021
Re	-0.347876	-0.524652	0.318362
C	-0.699274	-0.622847	2.174424
O	-0.882334	-0.697760	3.331873
C	1.712511	-0.470424	0.692061
C	2.610768	-0.038239	-0.505547
C	4.000755	0.397142	-0.161826
O	4.936037	-0.505705	-0.479947
C	6.293081	-0.168786	-0.107355
C	-0.361981	-2.420113	0.243203
O	-0.410999	-3.594411	0.179683
S	0.435371	-0.292096	-2.097643
C	2.186922	-0.025916	-1.771584
O	2.367593	-0.693867	1.723820
O	4.237166	1.467099	0.372429
H	2.797841	0.213919	-2.633923
H	6.896174	-1.018894	-0.416050
H	6.608703	0.739102	-0.622435
H	6.353430	-0.019185	0.970905
H	-1.120687	5.520515	0.003764
H	-6.075816	0.787349	-1.299057
H	-5.358368	-1.616828	-1.060483
H	-3.005148	-2.097120	-0.412991
H	-4.405820	2.569447	-0.888721
H	-2.952782	3.947377	-0.536783
H	1.112606	4.583110	0.699726
H	1.389077	2.123157	0.804297
H	0.513921	-1.546052	-2.590165

TS1*b*

N	-0.036539	1.530572	0.437924
C	1.043087	1.094251	1.139504
C	2.067534	1.974292	1.501959
C	1.975620	3.317901	1.155921
C	0.857596	3.758816	0.447973
C	-0.118501	2.833004	0.101364
C	1.043045	-0.336908	1.501064
N	-0.026694	-1.055632	1.073898
C	-0.102785	-2.363439	1.386408
C	0.864758	-3.009435	2.145369
C	1.965093	-2.278428	2.591498
C	2.055054	-0.931177	2.260451
Re	-1.526943	0.010439	-0.139040
S	0.077502	-0.321573	-2.117638
C	-2.594222	-1.517642	-0.618494
O	-3.215583	-2.462283	-0.894404
C	-2.646447	1.127571	-1.241843
O	-3.303343	1.820883	-1.905571
C	-2.639205	0.352608	1.393250
O	-3.318448	0.566116	2.313914
C	1.447885	-1.920490	-1.671386
C	2.692108	-1.708066	-1.500141
C	3.689977	-0.704550	-1.532569
O	3.886287	-0.113848	-0.306763
C	4.986596	0.805512	-0.231263
O	4.387640	-0.403726	-2.507007
H	0.777646	-2.762278	-1.634833
H	4.942748	1.230378	0.771081
H	5.936195	0.284500	-0.376748
H	4.892705	1.593028	-0.980528
H	-0.996624	3.130050	-0.456496
H	0.739014	4.794807	0.158391
H	2.762949	4.008676	1.432562
H	2.929668	1.609124	2.043188
H	2.904764	-0.348074	2.586300
H	2.741710	-2.748665	3.182557
H	0.750943	-4.061842	2.371623
H	-0.971069	-2.893697	1.018034
H	-0.702927	-1.018418	-2.963389

P*ccoh*

C	-0.051298	2.464094	0.851580
N	-0.857520	1.502705	0.359843
C	-2.129111	1.826307	0.004168
C	-2.603578	3.136544	0.137387
C	-1.766290	4.124419	0.644440
C	-0.464823	3.781152	1.012410
Re	-0.224197	-0.602137	0.108315
C	0.020899	-2.471498	-0.197229
O	0.113617	-3.624596	-0.387109
C	-2.948848	0.716125	-0.515296
N	-2.338904	-0.499955	-0.536184
C	-3.031096	-1.560834	-1.001492
C	-4.338766	-1.466488	-1.459289
C	-4.971649	-0.222427	-1.437323
C	-4.267893	0.876600	-0.957201
C	-0.492957	-0.960082	1.954376
O	-0.616596	-1.178511	3.102340
C	1.789542	-0.365637	0.389089
C	2.660883	0.065227	-0.705021
C	4.100213	0.241836	-0.511331
O	4.774391	0.668428	-1.591146
C	6.199405	0.838103	-1.434977
C	2.083157	0.280593	-1.951413
O	2.395607	-0.575959	1.560234
O	4.682875	0.018804	0.563733
H	2.707888	0.598997	-2.779588
H	6.408341	1.563823	-0.648543
H	6.551144	1.199916	-2.398210
H	6.667322	-0.115205	-1.186804
H	-2.124233	5.141083	0.752695
H	-5.991106	-0.110423	-1.785694
H	-4.843139	-2.352406	-1.823796
H	-2.505645	-2.507393	-0.997764
H	-4.737985	1.851052	-0.933192
H	-3.617402	3.380303	-0.149313
H	0.223570	4.513211	1.415053
H	0.948939	2.150901	1.120352
H	3.376277	-0.419510	1.461488
S	0.434617	0.044158	-2.256395

I1*b*

N	-0.091661	1.408837	0.800200
C	0.819872	0.840473	1.633306
C	1.736908	1.626996	2.337191
C	1.721045	3.008931	2.182502
C	0.786890	3.581775	1.319499
C	-0.097431	2.747846	0.647290
C	0.764680	-0.630714	1.742248
N	-0.186501	-1.245213	0.991979
C	-0.294708	-2.586812	1.042835
C	0.519131	-3.373869	1.847536
C	1.492514	-2.751232	2.627565
C	1.616276	-1.367586	2.569903
Re	-1.455144	0.044252	-0.261769
S	0.458026	-0.007054	-1.981704
C	-2.431330	-1.350110	-1.166069
O	-2.999508	-2.212482	-1.700859
C	-2.370904	1.368584	-1.324678
O	-2.903270	2.188854	-1.953778
C	-2.810664	0.156775	1.100386
O	-3.633050	0.228814	1.918711
C	1.527422	-1.587135	-1.730005
C	2.784580	-1.553273	-1.372719
C	3.692059	-0.488418	-1.127182
O	3.699237	-0.089225	0.194994
C	4.751109	0.811707	0.566936
O	4.496570	-0.004042	-1.935302
H	0.893566	-2.449199	-1.898043
H	4.573940	1.052585	1.614958
H	5.728842	0.336252	0.452637
H	4.725052	1.720927	-0.036629
H	-0.836841	3.149256	-0.033109
H	0.738539	4.651152	1.161610
H	2.426871	3.627885	2.723244
H	2.459005	1.161653	2.994063
H	2.369321	-0.865440	3.160779
H	2.147185	-3.331716	3.266250
H	0.386707	-4.448188	1.852586
H	-1.063064	-3.029372	0.423038
H	-0.209067	-0.395138	-3.084693

I1′*b*

C	-3.398747	0.849527	-0.865652
N	-2.160977	0.872725	-0.333052
C	-1.694341	2.027862	0.211257
C	-2.486467	3.180347	0.235330
C	-3.763361	3.150628	-0.315882
C	-4.227662	1.963961	-0.882503
Re	-0.824957	-0.875072	-0.255017
S	0.146786	0.013101	-2.470140
C	1.974347	-0.185074	-2.316654
C	2.727430	0.886003	-2.145313
C	4.142844	0.846382	-1.969507
O	4.512311	0.795918	-0.638094
C	5.922028	0.883192	-0.383763
C	-0.317571	1.979311	0.743438
N	0.328981	0.788601	0.610528
C	1.612077	0.693271	1.005130
C	2.293479	1.749783	1.599466
C	1.627030	2.957639	1.780606
C	0.311765	3.075792	1.336167
C	-1.531511	-1.425239	1.448683
O	-1.956688	-1.771858	2.473096
C	0.533828	-2.243776	-0.154034
O	1.363076	-3.054462	-0.083387
C	-1.992225	-2.108295	-1.164691
O	-2.719972	-2.821746	-1.725825
O	5.005299	0.946294	-2.852798
H	2.239999	-1.243263	-2.320594
H	6.458504	0.060425	-0.861543
H	6.029733	0.821771	0.698611
H	6.327868	1.828634	-0.750518
H	2.103239	-0.253605	0.832575
H	3.328701	1.616471	1.883638
H	2.120911	3.802383	2.245491
H	-4.381470	4.040201	-0.305642
H	-5.212167	1.892959	-1.326943
H	-3.720066	-0.093410	-1.287441
H	-2.110110	4.093151	0.675372
H	-0.211719	4.015453	1.444027
H	-0.120123	-1.027050	-3.285661

P*ccb*

Re	-0.903559	-0.869654	0.116741
N	0.411965	0.690410	0.707854
N	-2.010359	0.968243	-0.328708
C	-1.896614	-1.209653	1.689760
O	-2.505100	-1.396839	2.667906
C	0.250264	-2.355315	0.546935
O	0.935518	-3.255165	0.823403
C	-2.062980	-2.066684	-0.868154
O	-2.753060	-2.757100	-1.508462
C	1.722574	-0.184066	-1.733466
H	2.216187	-0.393053	-2.675043
C	2.433419	0.130476	-0.636324
C	3.904330	0.054230	-0.864775
O	4.455652	0.104962	-1.956866
O	4.586287	-0.110057	0.283384
C	6.023112	-0.179260	0.166732
H	6.314461	-1.077221	-0.380017
H	6.392149	-0.217166	1.188957
H	6.400340	0.702883	-0.351998
C	1.861551	0.442172	0.765659
H	2.018210	-0.460885	1.365534
C	2.577472	1.594969	1.451099
H	3.482018	1.379184	2.004534
C	2.090017	2.849393	1.328499
H	2.619397	3.690995	1.765475
C	0.827446	3.074587	0.680865
C	0.016633	1.980140	0.457341
C	-1.367773	2.129193	-0.022112
C	-2.031610	3.359134	-0.158628
C	-3.335680	3.394976	-0.630233
H	-3.851159	4.342757	-0.737112
C	-3.976146	2.194178	-0.959440
H	-4.992256	2.174917	-1.331693
C	-3.281281	1.008599	-0.787824
H	-3.738196	0.053769	-1.013405
H	-1.526484	4.275153	0.115914
H	0.485387	4.079485	0.479444
H	0.067537	-0.641617	-2.634000
S	0.348161	-0.285566	-1.779585

TS2*ccb*

Re	-0.838935	-0.908349	-0.108912
N	0.327418	0.812921	0.552017
N	-2.174482	0.827749	-0.353969
C	-1.580468	-1.318288	1.612212
O	-2.025728	-1.574322	2.655916
C	0.513768	-2.261414	0.143624
O	1.334237	-3.066792	0.312229
C	-1.987471	-2.214206	-0.945022
O	-2.698682	-2.971399	-1.472481
C	2.031230	-0.242234	-2.165213
H	2.475844	-0.750581	-3.019893
C	2.655707	0.331156	-1.152443
C	4.083690	0.421182	-1.001650
O	4.833811	1.210288	-1.581295
O	4.542471	-0.388811	0.002720
C	5.948240	-0.292946	0.295425
H	6.542349	-0.566083	-0.578434
H	6.125545	-0.995016	1.107904
H	6.208380	0.721983	0.602270
C	1.672573	0.742998	0.799663
H	2.048035	-0.238014	1.056467
C	2.346094	1.887370	1.335379
H	3.359075	1.767938	1.699366
C	1.724545	3.107588	1.309876
H	2.227288	3.995078	1.677305
C	0.405391	3.205446	0.806941
C	-0.265349	2.043660	0.461275
C	-1.670778	2.036919	0.011422
C	-2.470247	3.186328	-0.039388
C	-3.782960	3.096868	-0.485206
H	-4.406212	3.981885	-0.527955
C	-4.284464	1.851293	-0.870264
H	-5.299121	1.731879	-1.226570
C	-3.450456	0.745879	-0.785165
H	-3.798742	-0.239785	-1.062840
H	-2.064569	4.139839	0.269132
H	-0.081707	4.168353	0.741296
H	0.077252	-1.280706	-3.102123
S	0.205551	-0.169207	-2.354998

TS2*ins*

C	-1.502844	3.417379	0.297269
C	-1.373255	2.030716	0.164043
N	-0.257941	1.385990	0.597218
C	0.740478	2.104099	1.147321
C	0.667068	3.480878	1.318274
C	-0.476674	4.150150	0.884253
C	-2.415558	1.179422	-0.441320
N	-2.141433	-0.149692	-0.483069
C	-3.048901	-0.986239	-1.024339
C	-4.257232	-0.547727	-1.550308
C	-4.546237	0.817307	-1.513900
C	-3.617718	1.685247	-0.950566
Re	-0.207480	-0.815026	0.371359
C	-0.410698	-2.683522	-0.016160
O	-0.584250	-3.809623	-0.267363
C	1.592545	-1.124046	1.021566
O	2.629601	-1.326073	1.509752
C	-0.928458	-1.102995	2.080979
O	-1.351417	-1.275904	3.160547
C	2.112152	0.104560	-1.186114
C	3.410650	0.664401	-0.975699
O	3.653490	1.810357	-0.575882
C	1.421181	-0.044166	-2.291224
O	4.420737	-0.248547	-1.138938
C	5.740608	0.203700	-0.795414
H	1.622185	1.551462	1.439674
H	1.496675	4.008046	1.771011
H	-0.568385	5.223946	0.997106
H	-2.394907	3.918650	-0.052905
H	-2.786884	-2.036116	-1.029522
H	-4.948590	-1.263692	-1.976327
H	-5.478119	1.199052	-1.913497
H	-3.824062	2.746047	-0.912706
H	1.672750	0.193298	-3.324279
H	6.044474	1.035511	-1.434945
H	5.779784	0.522850	0.247593
H	6.393834	-0.652620	-0.954384
S	-0.251332	-0.627545	-2.115810
H	-0.823286	-0.676105	-3.293354

Pins

C	-2.179090	1.139635	-0.487661
N	-1.871848	-0.094184	-0.008297
C	-2.826342	-1.044294	0.006833
C	-4.115033	-0.823534	-0.463584
C	-4.428414	0.427916	-0.992264
C	-3.450567	1.416765	-1.001695
Re	0.180419	-0.378333	0.780388
C	0.901846	-0.734579	-1.320102
C	2.126123	0.035478	-1.650301
O	3.253110	-0.698268	-1.464696
C	4.507137	-0.001014	-1.590811
C	-1.117164	2.159817	-0.375233
N	0.036827	1.752555	0.215600
C	1.024079	2.647954	0.401379
C	0.912947	3.980524	0.023962
C	-0.262197	4.405059	-0.591463
C	-1.284253	3.483125	-0.796496
C	-0.465925	-0.044177	2.592271
O	-0.836228	0.127943	3.683228
C	0.132908	-2.281164	1.080764
O	0.102314	-3.429212	1.273992
C	2.000512	-0.340683	1.392546
O	3.106748	-0.263987	1.755025
C	0.499189	-1.571702	-2.289493
S	-0.958366	-2.608940	-2.215887
O	2.173879	1.212776	-1.989971
H	1.924629	2.278246	0.872164
H	1.736385	4.658381	0.207794
H	-0.385445	5.434133	-0.906455
H	-2.205180	3.796879	-1.268140
H	-2.533330	-2.006731	0.404475
H	-4.844118	-1.622536	-0.423417
H	-5.416841	0.634246	-1.384653
H	-3.679155	2.396421	-1.397755
H	1.021786	-1.662438	-3.240252
H	4.546813	0.556321	-2.526887
H	4.637037	0.682670	-0.749216
H	5.274647	-0.772787	-1.568725
H	-0.594337	-3.448448	-3.200617

Figure S9. PCM-B3LYP/6-31+G(d,p) (LANL2DZ for Re) optimized geometries in THF solution of the critical structures involved in the reaction between the complex [Re(SH)(CO)$_3$(bipy)] (bipy = 2,2'-bipyridine) and methyl propiolate (HMAD, HC≡CCO$_2$Me). Relevant distances are given in angstroms.

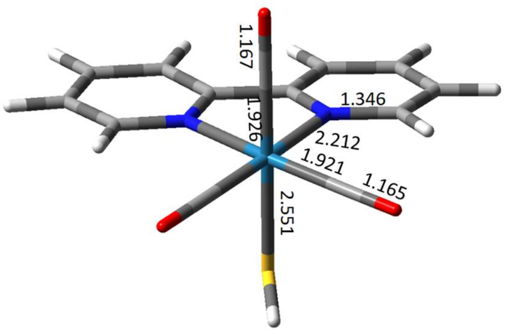

[Re(SH)(CO)$_3$(bipy)]

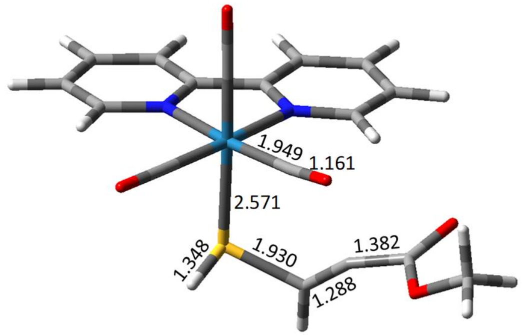

TS1

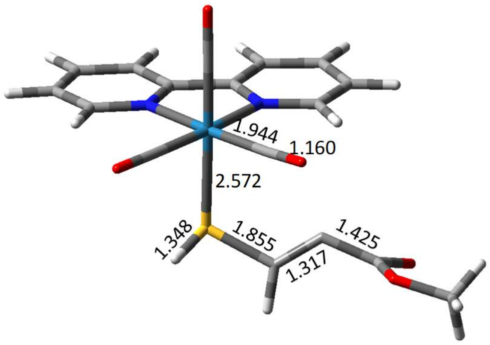

I1

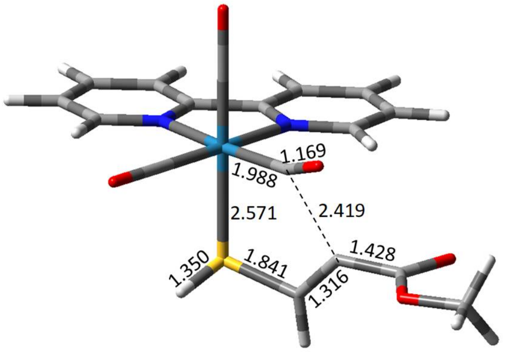

TS2*cco*

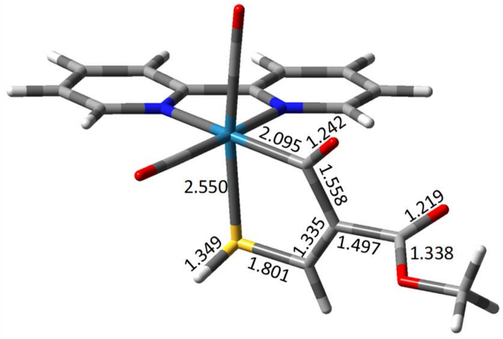

P*cco*

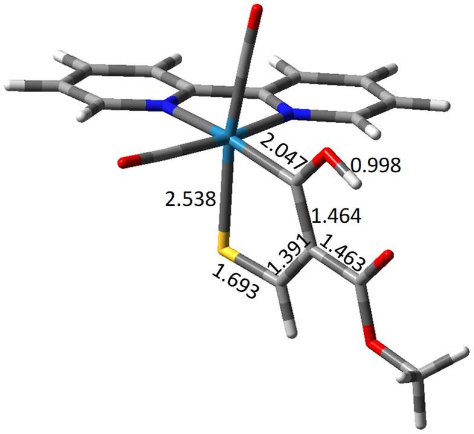

P*ccoh*

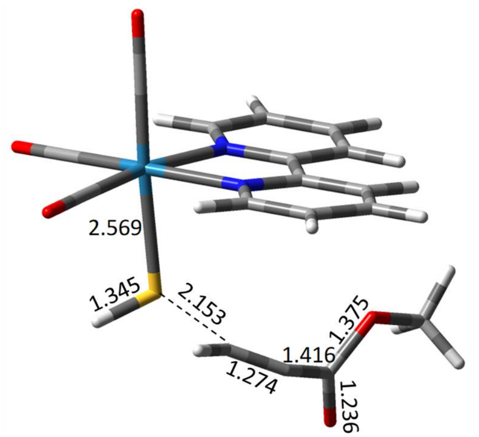

TS1*b*

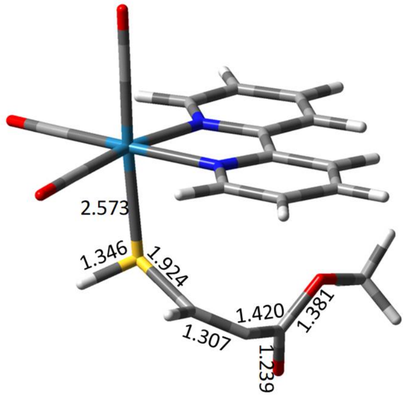

I1*b*

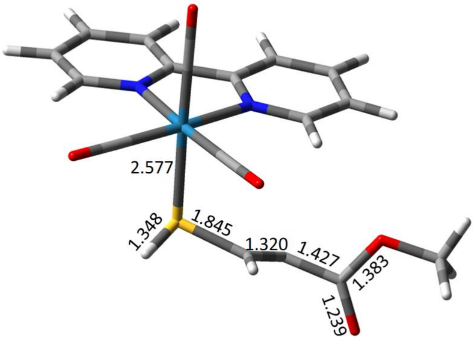

I1'*b*

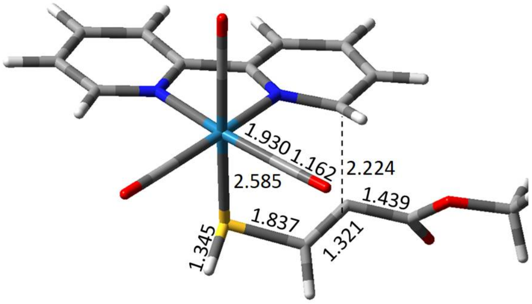

TS2*ccb*

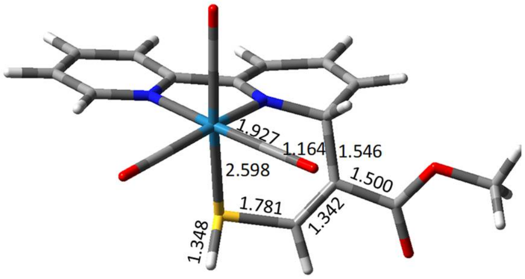

P*ccb*

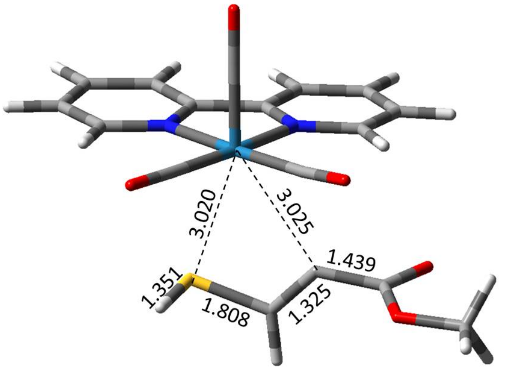

TS2*ins*

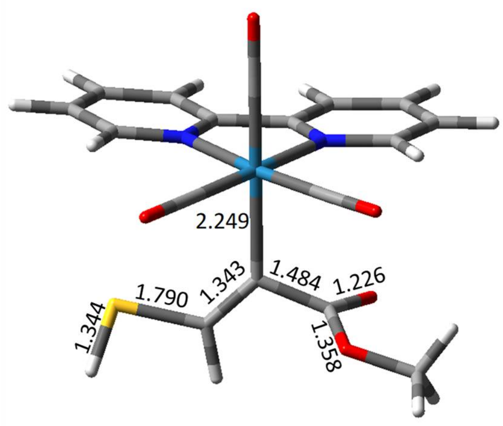

P*ins*

Table S28. PCM-B3LYP/6-31+G(d,p) (LANL2DZ for Re) energies without and with including thermal corrections (E and G, respectively), enthalpies (H), and entropies (S), and CPCM-DLPNO-CCSD(T)/def2-TZVPP//PCM-B3LYP/6-31+G(d,p) (LANL2DZ for Re) energies without and with including thermal corrections (E' and G', respectively) in THF solution of the critical structures involved in the reaction between the complex [Re(SMe)(CO)$_3$(bipy)] (bipy = 2,2'-bipyridine) and methyl propiolate (HMAD, HC≡CCO$_2$Me). All the values are given in hartree, except entropies that are in cal/K mol.[a]

Species	E	H	S	G	E'	G'[b]
[Re(SMe)(CO)$_3$(bipy)]	-1352.773939	-1352.525603	151.188	-1352.597437	-1349.822364	-1349.645862
HMAD	-305.215726	-305.136831	77.228	-305.173524	-304.738441	-304.696239
Reactants	-1657.989665	-1657.662434	228.416	-1657.770961	-1654.560805	-1654.342101
**TS1**	-1657.972361	-1657.644525	181.903	-1657.730953	-1654.546712	-1654.305304
**I1**	-1657.974249	-1657.644837	179.022	-1657.729896	-1654.551547	-1654.307194
**TS1'**	-1657.967887	-1657.640670	179.208	-1657.725818	-1654.542525	-1654.300456
**I1'**	-1657.971803	-1657.643036	185.363	-1657.731108	-1654.550524	-1654.309829
**TS2*cco***	-1657.970655	-1657.642958	177.697	-1657.727388	-1654.545938	-1654.302671
**P*cco***	-1657.994157	-1657.664081	179.612	-1657.749420	-1654.569387	-1654.324650
**TS1*b***	-1657.968339	-1657.640699	186.590	-1657.729353	-1654.547582	-1654.308596
**I1*b***	-1657.973151	-1657.643793	181.855	-1657.730198	-1654.557467	-1654.314514
**I1'*b***	-1657.972898	-1657.643928	184.597	-1657.731636	-1654.553393	-1654.312131
**TS2*ccb***	-1657.963925	-1657.636449	179.502	-1657.721736	-1654.542145	-1654.299956
**P*ccb***	-1657.993908	-1657.664097	177.852	-1657.748600	-1654.582424	-1654.337116
**TS2*ins***	-1657.958334	-1657.631284	174.858	-1657.714365	-1654.534887	-1654.290918
**P*ins***	-1658.016403	-1657.686591	182.354	-1657.773233	-1654.599799	-1654.356629

[a] Thermal magnitudes were computed in THF solution at 298.15 K and 1 atm. [b] For each species, G' was calculated as G' = G – E + E', in which G is the PCM-B3LYP/6-31+G(d,p) (LANL2DZ for Re) energy with including thermal corrections and E and E' are the PCM-B3LYP/6-31+G(d,p) (LANL2DZ for Re) and CPCM-DLPNO-CCSD(T)/def2-TZVPP//PCM-B3LYP/6-31+G(d,p) (LANL2DZ for Re) energies without including thermal corrections, respectively.

Table S29. PCM-B3LYP/6-31+G(d,p) (LANL2DZ for Re) relative energies without and with including thermal corrections (ΔE and ΔG, respectively), enthalpies (ΔH), and entropic contributions (TΔS), and CPCM-DLPNO-CCSD(T)/def2-TZVPP//PCM-B3LYP/6-31+G(d,p) (LANL2DZ for Re) relative energies without and with including thermal corrections (E' and G', respectively) in THF solution of the critical structures involved in the reaction between the complex [Re(SMe)$(CO)_3$(bipy)] (bipy = 2,2'-bipyridine) and methyl propiolate (HMAD, HC≡CCO_2Me). All the values are given in kcal/mol.[a]

Species	ΔE	ΔH	TΔS	ΔG	ΔE'	ΔG'
[Re(SMe)$(CO)_3$(bipy)] + HMAD	0.0	0.0	0.0	0.0	0.0	0.0
**TS1**	10.9	11.2	-13.9	25.1	8.8	23.1
**I1**	9.7	11.0	-14.7	25.8	5.8	21.9
**TS1'**	13.7	13.7	-14.7	28.3	11.5	26.1
**I1'**	11.2	12.2	-12.8	25.0	6.5	20.3
**TS2*cco***	11.9	12.2	-15.1	27.3	9.3	24.7
**P*cco***	-2.8	-1.0	-14.6	13.5	-5.4	11.0
**TS1*b***	13.4	13.6	-12.5	26.1	8.3	21.0
**I1*b***	10.4	11.7	-13.9	25.6	2.1	17.3
**I1'*b***	10.5	11.6	-13.1	24.7	4.7	18.8
**TS2*ccb***	16.2	16.3	-14.6	30.9	11.7	26.4
**P*ccb***	-2.7	-1.0	-15.1	14.0	-13.6	3.1
**TS2*ins***	19.7	19.5	-16.0	35.5	16.3	32.1
**P*ins***	-16.8	-15.2	-13.7	-1.4	-24.5	-9.1

[a] Thermal magnitudes were evaluated in THF solution at 298.15 K and 1 atm.

Table S30. PCM-B3LYP/6-31+G(d,p) (LANL2DZ for Re) optimized cartesian coordinates, in Å, for the critical structures involved in the reaction of the complex [Re(SMe)(CO)$_3$(bipy)] (bipy = 2,2'-bipyridine) towards methyl propiolate (HMAD, HC≡CCO$_2$Me).

[Re(SMe)(CO)$_3$(bipy)]

N	-0.906411	1.333727	0.054045
C	-2.125991	0.737394	0.096835
C	-3.300188	1.495926	0.146594
C	-3.222405	2.884575	0.146362
C	-1.966771	3.488338	0.088691
C	-0.838061	2.678530	0.043376
C	-2.122684	-0.738570	0.076533
C	-3.293492	-1.503419	0.104834
C	-3.209487	-2.891182	0.066715
C	-1.951137	-3.487529	-0.006708
C	-0.826054	-2.671744	-0.029430
N	-0.900432	-1.328051	0.017914
Re	0.863700	0.007559	-0.019636
S	0.477897	0.040700	-2.525307
C	1.031818	-0.018124	1.903443
O	1.156195	-0.033559	3.064420
C	2.209893	1.367472	-0.183144
O	3.003862	2.212011	-0.307922
C	2.215912	-1.341445	-0.219794
O	3.013594	-2.178788	-0.367332
H	0.154872	3.105636	-0.003219
H	-4.264344	1.006953	0.183665
H	-1.853952	4.564730	0.079238
H	-4.124994	3.481945	0.186722
H	0.168793	-3.092978	-0.087253
H	-4.259855	-1.019954	0.154446
H	-1.833491	-4.562748	-0.045460
H	-4.109411	-3.493456	0.090200
C	2.146290	0.054864	-3.303763
H	2.018330	0.069340	-4.387852
H	2.710840	0.940713	-3.004015
H	2.714628	-0.836387	-3.028159

TS1

C	-1.631218	3.446561	0.144239
C	-1.521644	2.053994	0.115086
N	-0.385212	1.428307	0.527002
C	0.650814	2.172365	0.958654
C	0.597436	3.561825	1.019353
C	-0.563385	4.210393	0.608017
C	-2.610433	1.174505	-0.354250
C	-3.852461	1.654097	-0.784583
C	-4.828826	0.756311	-1.203064
C	-4.543027	-0.608804	-1.189271
C	-3.291549	-1.021553	-0.749543
N	-2.344222	-0.157422	-0.335916
Re	-0.367461	-0.779607	0.421637
S	0.409833	-0.486763	-1.988307
C	1.683417	-1.775369	-2.254384
C	1.435635	-1.133153	1.017831
O	2.518758	-1.369858	1.365616
C	-0.604713	-2.661438	0.114857
O	-0.785252	-3.791994	-0.100727
C	-1.030226	-0.958980	2.224400
O	-1.418223	-1.089962	3.314087
C	1.807300	1.193581	-2.216017
C	2.871904	1.313625	-1.540831
C	3.739094	0.985527	-0.481717
O	4.706373	0.081173	-0.831261
C	5.658071	-0.235511	0.200006
O	3.705433	1.488113	0.652032
H	1.305362	1.704023	-3.019741
H	6.194950	0.660243	0.518791
H	6.346741	-0.948139	-0.250848
H	5.153861	-0.681715	1.059389
H	1.557133	1.648724	1.235311
H	1.461617	4.108329	1.373947
H	-0.638802	5.290431	0.638291
H	-5.794419	1.118649	-1.535381
H	-5.269491	-1.344975	-1.508435
H	-3.027025	-2.069908	-0.716117
H	-4.058608	2.715714	-0.786733
H	-2.538198	3.930786	-0.189904
H	1.267932	-2.754446	-2.014790
H	1.985008	-1.752831	-3.301465
H	2.548396	-1.569933	-1.620437

I1

C	-1.519583	3.427950	0.270016
C	-1.493668	2.035797	0.159311
N	-0.449718	1.310917	0.647661
C	0.587778	1.955338	1.215004
C	0.614785	3.340092	1.357881
C	-0.459546	4.088428	0.886586
C	-2.564853	1.262555	-0.499502
C	-3.678359	1.854990	-1.105746
C	-4.630084	1.055328	-1.729366
C	-4.452592	-0.327980	-1.732889
C	-3.329459	-0.856462	-1.109628
N	-2.403678	-0.086952	-0.503586
Re	-0.602629	-0.885414	0.492151
C	1.079332	-1.419254	1.286645
O	2.080289	-1.781422	1.748619
S	0.460316	-0.668183	-1.825591
C	1.641771	-2.055571	-1.979328
C	-0.973644	-2.743063	0.157122
O	-1.230421	-3.856869	-0.066201
C	-1.471860	-1.000414	2.209226
O	-1.978707	-1.085109	3.252119
C	1.649746	0.834601	-1.989692
C	2.764217	1.050471	-1.341375
C	3.518136	0.495582	-0.286042
O	4.447048	-0.436058	-0.702522
C	5.350397	-0.902375	0.311579
O	3.478421	0.850436	0.906341
H	1.214422	1.453332	-2.767354
H	5.937604	-0.075876	0.718593
H	6.004147	-1.617366	-0.186422
H	4.801401	-1.386317	1.122224
H	1.432210	1.352753	1.526205
H	1.473724	3.806926	1.822249
H	-0.472851	5.167859	0.982803
H	-5.491881	1.505140	-2.207633
H	-5.163894	-0.991568	-2.207433
H	-3.152949	-1.923160	-1.089241
H	-3.797253	2.929572	-1.100216
H	-2.356703	3.992718	-0.116506
H	2.075239	-2.024400	-2.978278
H	2.428580	-1.964899	-1.231187
H	1.089179	-2.984674	-1.841207

TS1'

Re	0.330603	-0.750548	-0.404535
C	0.844381	-0.843705	-2.256571
O	1.149896	-0.922338	-3.376062
N	2.352251	-0.093629	0.182063
C	2.568159	1.247037	0.243460
C	3.828800	1.758664	0.569248
C	4.880458	0.887279	0.830626
C	4.648288	-0.486641	0.768077
C	3.373921	-0.933527	0.442604
C	1.404887	2.104039	-0.058374
N	0.254924	1.454900	-0.388897
C	-0.849558	2.175953	-0.661391
C	-0.858853	3.567015	-0.629228
C	0.315180	4.240927	-0.302929
C	1.458794	3.499401	-0.016206
C	-1.504051	-1.117821	-0.932909
O	-2.554710	-1.377770	-1.352314
C	0.650436	-2.635827	-0.216674
O	0.897261	-3.766139	-0.078972
C	-2.772643	0.467345	1.390939
C	-3.811423	0.838950	0.576258
O	-4.784172	-0.148513	0.458497
C	-5.691983	0.010974	-0.636018
O	-3.910605	1.909422	-0.076541

H	-2.011711	0.448572	3.415931
H	-6.200050	0.976113	-0.595867
H	-6.416677	-0.797494	-0.537169
H	-5.164065	-0.079657	-1.590342
H	-1.757772	1.633800	-0.883798
H	-1.784188	4.087301	-0.839233
H	0.344650	5.322884	-0.266461
H	5.861460	1.273989	1.078523
H	5.433636	-1.204941	0.964540
H	3.152022	-1.990933	0.379455
H	3.986290	2.827624	0.614270
H	2.379587	4.001666	0.247980
S	-0.203619	-0.497393	2.088676
C	-1.936026	0.244574	2.346943
C	-0.491888	-2.166775	2.779495
H	0.452458	-2.711553	2.779957
H	-0.848605	-2.052847	3.803069
H	-1.230884	-2.696056	2.178583

I1′

C	-3.692937	1.861811	-0.895371
C	-2.530938	1.220900	-0.451986
N	-2.414208	-0.132062	-0.508226
C	-3.433747	-0.856983	-1.009995
C	-4.610910	-0.279215	-1.468508
C	-4.743084	1.107627	-1.407384
C	-1.380241	1.945510	0.125204
N	-0.351746	1.172936	0.565403
C	0.714445	1.762326	1.139518
C	0.803713	3.140607	1.305944
C	-0.234266	3.942070	0.837482
C	-1.336009	3.338434	0.237628
Re	-0.539988	-1.006940	0.261137
S	0.320101	-0.509768	-2.104093
C	2.016214	0.187748	-2.060990
C	2.691941	0.379396	-0.942748
C	3.975766	0.983444	-0.830427
O	4.212054	2.187140	-0.635313
C	-0.934895	-2.830078	-0.197822
O	-1.213268	-3.921488	-0.497099
C	-1.287239	-1.283255	2.011851
O	-1.730929	-1.467423	3.071349
C	1.197231	-1.575701	0.923532
O	2.178989	-1.984743	1.386572
O	5.003836	0.063651	-0.820938
C	6.307792	0.586532	-0.532708
H	2.305177	0.428612	-3.087487
H	6.611708	1.318035	-1.284680
H	6.978110	-0.271708	-0.550097
H	6.328415	1.061164	0.451222
H	1.515463	1.104419	1.442515
H	1.680794	3.563741	1.777552
H	-0.190254	5.020492	0.933844
H	-5.647341	1.595243	-1.750635
H	-5.400628	-0.908610	-1.858044
H	-3.291257	-1.928984	-1.032989
H	-3.779853	2.937463	-0.834450
H	-2.149746	3.945069	-0.135086
C	0.645140	-2.114058	-2.924939
H	1.086036	-1.913883	-3.902457
H	1.326024	-2.710255	-2.317984
H	-0.304843	-2.633848	-3.050410

TS2*cco*

C	-4.000479	1.457235	-0.737495
C	-2.714777	1.017666	-0.402670
N	-2.377881	-0.296307	-0.497935
C	-3.303179	-1.178129	-0.927002
C	-4.592405	-0.802747	-1.282456
C	-4.947322	0.542782	-1.186463
C	-1.650889	1.924687	0.074339
N	-0.453123	1.343082	0.351856
C	0.566408	2.111943	0.781243
C	0.437245	3.482747	0.977697
C	-0.787387	4.087571	0.701091
C	-1.838934	3.300478	0.240767
Re	-0.334920	-0.857611	0.103874
S	0.278608	-0.299440	-2.324178
C	1.988590	0.323722	-2.220304
C	2.621925	0.321573	-1.063152
C	3.898571	0.858889	-0.723828
O	4.107653	2.001741	-0.290781
C	-0.480772	-2.729369	-0.262054
O	-0.618758	-3.865507	-0.499434
C	-0.856273	-1.188459	1.913247
O	-1.164642	-1.408757	3.017070
C	1.556505	-1.096823	0.647671
O	2.494705	-1.444302	1.250571
O	4.901543	-0.073375	-0.775507
C	6.185052	0.356453	-0.294593
H	2.321667	0.713511	-3.182793
H	6.582041	1.163708	-0.913915
H	6.828569	-0.519017	-0.359655
H	6.114891	0.701350	0.739145
H	-0.923482	5.153721	0.836096
H	-5.943832	0.875507	-1.450807
H	-5.294403	-1.553411	-1.622335
H	-2.986214	-2.211692	-0.975420
H	-4.260078	2.503727	-0.653030
H	-2.792766	3.755368	0.011145
H	1.287550	4.052889	1.329096
H	1.509570	1.606953	0.943934
C	0.510985	-1.868713	-3.239326
H	0.879415	-1.641229	-4.239578
H	1.216621	-2.504876	-2.706067
H	-0.459672	-2.360991	-3.309828

P*cco*

N	-2.458854	-0.036138	-0.285260
C	-2.791555	1.270177	-0.471057
C	-4.059468	1.638156	-0.938967
C	-5.001328	0.655812	-1.223924
C	-4.656129	-0.682231	-1.024374
C	-3.383547	-0.981732	-0.555098
C	-1.744216	2.253184	-0.128691
C	-1.935464	3.637111	-0.217831
C	-0.913747	4.499522	0.165215
C	0.289407	3.960168	0.620766
C	0.425880	2.577915	0.667283
N	-0.563339	1.735588	0.305466
Re	-0.386444	-0.481704	0.363654
S	0.345636	-0.238020	-2.061220
C	2.097590	-0.039075	-1.778845
C	2.559274	-0.038381	-0.522675
C	3.971867	0.371296	-0.250073
O	4.256797	1.401291	0.335851
C	-0.709818	-0.588558	2.226552
O	-0.871424	-0.671468	3.387141
C	1.682140	-0.428424	0.700231
O	2.347470	-0.633117	1.729833
C	-0.399586	-2.377063	0.293221
O	-0.437220	-3.553149	0.240442
O	4.878743	-0.496919	-0.721941
C	6.265001	-0.152390	-0.492967
H	2.706537	0.151885	-2.656705
H	6.840180	-0.952580	-0.952096
H	6.493842	0.807337	-0.957999
H	6.463229	-0.097321	0.577745
H	-1.053385	5.572426	0.105907
H	-5.983586	0.929594	-1.588924
H	-5.356143	-1.483511	-1.223050
H	-3.074652	-2.004063	-0.379808
H	-4.308229	2.681426	-1.077851
H	-2.874916	4.037603	-0.573799
H	1.113851	4.589279	0.930936
H	1.348704	2.119159	1.000440
C	0.301892	-1.829297	-2.967695
H	0.882527	-1.729328	-3.884753
H	0.692849	-2.628639	-2.340314
H	-0.742833	-2.018396	-3.213282

TS1*b*

C	2.048551	-0.945506	2.195497
C	1.021881	-0.337047	1.466924
N	-0.066564	-1.042326	1.066314
C	-0.150889	-2.349481	1.379996
C	0.825345	-3.006925	2.117648
C	1.947037	-2.290178	2.532195
C	1.024954	1.096194	1.113845
N	-0.058711	1.539562	0.422525
C	-0.133578	2.841134	0.082013
C	0.846933	3.762122	0.426587
C	1.959389	3.318927	1.141398
C	2.051848	1.973035	1.479043
Re	-1.583946	0.040430	-0.113347
C	-2.646709	0.404228	1.454179
O	-3.302148	0.631415	2.389774
S	-0.054020	-0.278580	-2.132396

C	-2.667874	-1.486644	-0.548231
O	-3.284860	-2.441000	-0.803620
C	-2.722000	1.156829	-1.196513
O	-3.392192	1.845318	-1.853110
C	1.513545	-1.886561	-1.606174
C	2.725407	-1.604780	-1.432754
C	3.792408	-0.675270	-1.444702
O	4.498542	-0.401269	-2.418774
O	4.022228	-0.120491	-0.212728
C	5.140444	0.779808	-0.141342
H	0.812641	-2.702453	-1.600928
H	5.188215	1.102228	0.897493
H	6.063882	0.270150	-0.422786
H	4.990612	1.638493	-0.798206
H	-1.011443	3.138717	-0.475546
H	0.733575	4.798320	0.136204
H	2.745076	4.008724	1.427136
H	2.912090	1.608214	2.023271
H	2.920586	-0.377713	2.484627
H	2.733309	-2.771209	3.101269
H	0.705173	-4.057094	2.351152
H	-1.035106	-2.869269	1.034930
C	-0.982358	-1.298425	-3.336254
H	-0.430953	-1.318157	-4.276799
H	-1.098644	-2.320165	-2.964098
H	-1.969532	-0.865780	-3.501319

I1*b*

C	1.622524	-1.309909	2.637188
C	0.781577	-0.601776	1.775587
N	-0.147365	-1.243109	1.020073
C	-0.230106	-2.586387	1.083897
C	0.578708	-3.346414	1.919103
C	1.517251	-2.693782	2.717496
C	0.822729	0.867355	1.638903
N	-0.109919	1.412561	0.814074
C	-0.131166	2.748684	0.638939
C	0.758806	3.602516	1.276972
C	1.715779	3.053298	2.130285
C	1.752218	1.673770	2.303384
Re	-1.451568	0.010203	-0.228911
C	-2.804218	0.076391	1.145349
O	-3.632161	0.109439	1.960039
S	0.442941	-0.011411	-1.960432
C	1.449151	-1.578530	-1.739405
C	2.715942	-1.608222	-1.383735
C	3.634823	-0.558502	-1.116500
O	4.426155	-0.042326	-1.920065
C	-2.402610	-1.402007	-1.131875
O	-2.954588	-2.266034	-1.681468
C	-2.409002	1.319063	-1.272457
O	-2.963434	2.136202	-1.887543
O	3.676612	-0.215820	0.222214
C	4.730281	0.678612	0.601747
H	0.809497	-2.436056	-1.937295
H	4.615939	0.828198	1.675105
H	5.707991	0.241695	0.386148
H	4.645266	1.633070	0.078458
H	-0.886437	3.131177	-0.033972
H	0.695039	4.668436	1.103582
H	2.429224	3.686678	2.643222
H	2.497846	1.227908	2.947051
H	2.352440	-0.786322	3.238198
H	2.162774	-3.252217	3.385656
H	0.467649	-4.423129	1.937725
H	-0.975136	-3.049989	0.451824
C	-0.351877	-0.368476	-3.568672
H	0.416878	-0.364186	-4.340705
H	-0.843990	-1.341679	-3.533390
H	-1.088264	0.409470	-3.768800

I1′*b*

C	0.319019	3.110883	1.275058
C	-0.320417	1.998409	0.725555
N	0.323460	0.803821	0.615624
C	1.609755	0.716360	1.001103
C	2.301927	1.790025	1.551993
C	1.639132	3.003160	1.707271
C	-1.704952	2.035014	0.214112
N	-2.169160	0.876048	-0.323782
C	-3.420617	0.838890	-0.822762
C	-4.267403	1.939886	-0.806420
C	-3.803958	3.130945	-0.248698
C	-2.512499	3.176104	0.265991

Re	-0.823627	-0.868896	-0.238206
C	-1.990629	-2.111672	-1.133657
O	-2.716941	-2.828544	-1.693405
S	0.159129	-0.010185	-2.455259
C	1.972986	-0.175748	-2.275077
C	2.740186	0.887795	-2.094354
C	4.155521	0.822929	-1.922044
O	5.017125	0.954742	-2.802963
C	-1.530668	-1.397595	1.476065
O	-1.955145	-1.726939	2.506371
C	0.534671	-2.235405	-0.126079
O	1.360375	-3.049540	-0.048428
O	4.529261	0.710873	-0.595592
C	5.935103	0.829684	-0.334044
H	2.250257	-1.234521	-2.288568
H	6.496106	0.030114	-0.822914
H	6.040453	0.753863	0.747776
H	6.314687	1.792525	-0.683435
H	2.097333	-0.235967	0.846611
H	3.340753	1.663901	1.826307
H	2.142882	3.861823	2.134982
H	-4.436033	4.009823	-0.211779
H	-5.264429	1.855500	-1.219226
H	-3.741737	-0.107491	-1.237030
H	-2.138533	4.092210	0.702436
H	-0.202777	4.053796	1.362730
C	-0.164851	-1.327105	-3.680942
H	0.269999	-1.027931	-4.634353
H	0.275559	-2.265140	-3.339665
H	-1.243433	-1.444376	-3.782893

TS2*ccb*

C	-3.438376	0.751079	-0.770916
N	-2.172434	0.822993	-0.308934
C	-1.654180	2.035777	0.023976
C	-2.419770	3.200854	-0.112450
C	-3.719279	3.121696	-0.596732
C	-4.241972	1.870946	-0.932586
Re	-0.845771	-0.920723	-0.043642
S	0.169935	-0.222445	-2.306867
C	1.989594	-0.231240	-2.129743
C	2.630915	0.339964	-1.125757
C	4.052911	0.466550	-0.988636
O	4.549129	-0.381613	-0.032173
C	5.954860	-0.262654	0.246679
C	-0.259779	2.034618	0.505754
N	0.323543	0.801690	0.606539
C	1.663464	0.725951	0.867221
C	2.343037	1.863737	1.402891
C	1.729197	3.089114	1.369701
C	0.413797	3.192688	0.860705
C	-1.570722	-1.315235	1.692853
O	-2.001974	-1.568702	2.742805
C	0.516349	-2.265618	0.197494
O	1.350087	-3.060650	0.351895
C	-1.999994	-2.241828	-0.844870
O	-2.710852	-3.014832	-1.350600
O	4.773128	1.311448	-1.529319
H	2.431480	-0.715554	-3.003176
H	6.542443	-0.431776	-0.657242
H	6.171242	-1.029915	0.987895
H	6.186274	0.727648	0.644314
H	2.040521	-0.259884	1.101629
H	3.355646	1.738844	1.766762
H	2.236576	3.975869	1.732791
H	-4.313989	4.020259	-0.710022
H	-5.248783	1.758557	-1.313137
H	-3.802707	-0.238109	-1.015323
H	-1.996161	4.158885	0.157483
H	-0.069245	4.157018	0.788883
C	-0.007569	-1.651994	-3.428214
H	0.558942	-1.451880	-4.338203
H	0.352686	-2.558904	-2.943412
H	-1.065246	-1.752856	-3.668703

P*ccb*

C	-3.385343	1.043950	-0.606015
N	-2.083716	0.999817	-0.243194
C	-1.408404	2.161537	-0.025560
C	-2.054831	3.396584	-0.193969
C	-3.384733	3.436926	-0.584088
C	-4.069991	2.233789	-0.791747
Re	-0.920049	-0.854958	0.010678
S	0.167726	-0.130072	-2.202036

C	1.910656	-0.080719	-1.869232
C	2.535270	0.216623	-0.716560
C	1.880136	0.510612	0.655184
N	0.435351	0.734602	0.543127
C	-0.000195	2.023202	0.390121
C	0.775604	3.127686	0.688557
C	2.035778	2.896291	1.335767
C	2.551732	1.648407	1.406564
C	-1.658811	-1.202281	1.745193
O	-2.100607	-1.421605	2.799862
C	0.291206	-2.336824	0.229510
O	1.023910	-3.231528	0.369849
C	-2.210608	-2.039872	-0.805443
O	-2.998690	-2.720793	-1.333115
C	4.027753	0.120800	-0.806493
O	4.590388	-0.167712	0.379444
C	6.031571	-0.261065	0.387613
O	4.677901	0.259829	-1.832128
H	2.509878	-0.301119	-2.748246
H	6.359317	-1.055435	-0.283531
H	6.301530	-0.487293	1.415558
H	6.468353	0.687046	0.071288
H	2.030923	-0.411218	1.231834
H	3.449175	1.427226	1.969165
H	2.536538	3.725129	1.827640
H	-3.884480	4.388638	-0.722012
H	-5.110788	2.215750	-1.087446
H	-3.873721	0.088498	-0.750976
H	-1.508972	4.313109	-0.016705
H	0.391817	4.130794	0.573237
C	0.135449	-1.475788	-3.437665
H	0.740528	-1.187363	-4.297367
H	0.501793	-2.400022	-2.993668
H	-0.905318	-1.592879	-3.738532

TS2*ins*

C	-3.596949	1.872251	-0.812297
C	-2.410357	1.270321	-0.374488
N	-2.224846	-0.071129	-0.478475
C	-3.211551	-0.826050	-1.002745
C	-4.410206	-0.291004	-1.453620
C	-4.605629	1.087800	-1.358129
C	-1.297197	2.028235	0.227894
C	-1.343438	3.409181	0.447442
C	-0.262849	4.043883	1.051968
C	0.849360	3.284872	1.410367
C	0.839508	1.920547	1.149718
N	-0.207206	1.297129	0.577932
Re	-0.293395	-0.903446	0.246363
C	-0.941880	-1.162157	1.995417
O	-1.332923	-1.338392	3.085139
C	-0.632031	-2.752218	-0.135435
O	-0.885026	-3.866316	-0.372459
C	1.509096	-1.329024	0.800996
O	2.568500	-1.581175	1.207928
C	1.978329	0.167967	-1.421486
C	1.510820	-0.126648	-2.628337
S	-0.074866	-0.956369	-2.741746
C	3.178215	0.901978	-1.123438
O	4.286325	0.100814	-1.050619
C	5.495171	0.731988	-0.597158
O	3.234957	2.102781	-0.829610
H	1.693480	1.302551	1.385892
H	1.718515	3.733517	1.872965
H	-0.288626	5.111839	1.232936
H	-2.214480	3.980732	0.157898
H	-3.019117	-1.889321	-1.060938
H	-5.166413	-0.945077	-1.868305
H	-5.526376	1.543104	-1.703246
H	-3.728879	2.942125	-0.726446
H	1.955255	0.126876	-3.593367
H	5.787628	1.540631	-1.270034
H	5.366354	1.132350	0.410239
H	6.251836	-0.050983	-0.597744
C	0.411466	-2.640756	-3.274585
H	-0.487696	-3.258172	-3.282293
H	0.834322	-2.602418	-4.278805
H	1.135550	-3.056832	-2.573797

P*ins*

C	2.953084	0.868221	-1.962430
C	2.066131	0.263954	-1.064655
N	1.495738	0.974957	-0.056520
C	1.833825	2.269083	0.098497
C	2.728598	2.920658	-0.742390
C	3.285475	2.209561	-1.803805
C	1.722228	-1.171742	-1.117135
C	2.307748	-2.063449	-2.022971
C	1.985771	-3.415725	-1.961105
C	1.073536	-3.847599	-0.999791
C	0.510817	-2.909221	-0.142431
N	0.831847	-1.602438	-0.185969
Re	0.049432	-0.096351	1.242225
C	1.452654	-0.541954	2.522477
O	2.268672	-0.810819	3.311398
C	-0.481066	1.358584	2.378893
O	-0.756160	2.224138	3.113363
C	-1.204099	-1.218097	2.177122
O	-1.936576	-1.907952	2.764556
C	-1.486011	0.323491	-0.338435
C	-1.960424	1.512099	-0.785763
S	-1.492270	3.110328	-0.204062
C	-2.139591	-0.847884	-0.974353
O	-2.621472	-0.625402	-2.235189
C	-3.340180	-1.713739	-2.837612
O	-2.260689	-1.956667	-0.458467
H	-0.219306	-3.194067	0.601037
H	0.792431	-4.889095	-0.910608
H	2.443078	-4.117275	-2.648093
H	3.016928	-1.710821	-2.758388
H	1.368019	2.786459	0.925696
H	2.966357	3.962239	-0.567914
H	3.973923	2.685428	-2.491349
H	3.384695	0.299009	-2.774718
H	-2.719864	1.565125	-1.564068
H	-2.701018	-2.593149	-2.927858
H	-4.219182	-1.971566	-2.243200
H	-3.640078	-1.358456	-3.821970
C	-2.592991	4.159473	-1.215994
H	-2.382862	5.192955	-0.938756
H	-2.389060	4.023062	-2.278524
H	-3.638922	3.936658	-1.003118

Figure S10. PCM-B3LYP/6-31+G(d,p) (LANL2DZ for Re) optimized geometries in THF solution of the critical structures involved in the reaction between the complex [Re(SMe)(CO)$_3$(bipy)] (bipy = 2,2'-bipyridine) and methyl propiolate (HMAD, HC≡CCO$_2$Me). Relevant distances are given in angstroms.

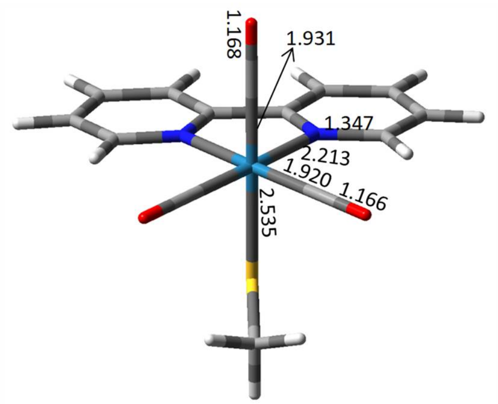

[Re(SMe)(CO)$_3$(bipy)]

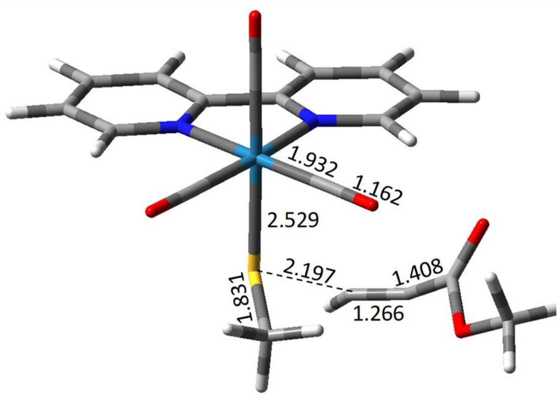

TS1

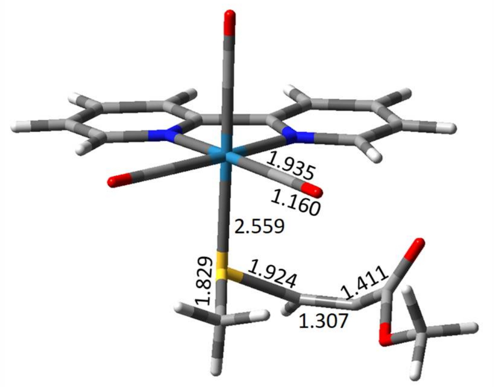

I1

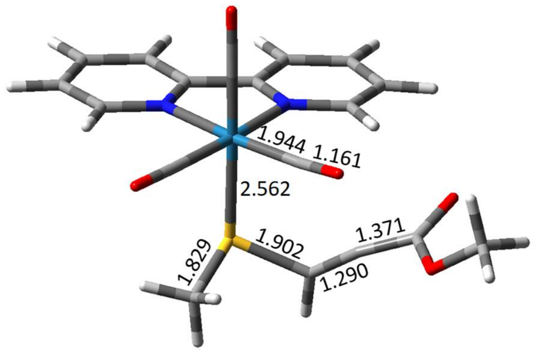

TS1'

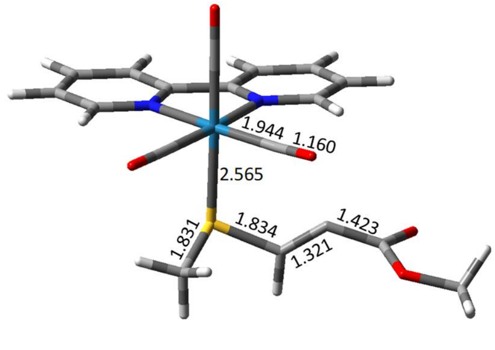

I1'

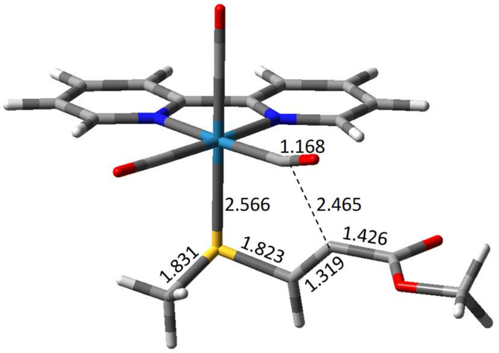

TS2*cco*

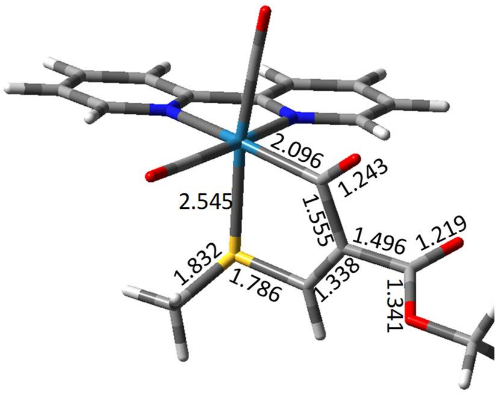

P*cco*

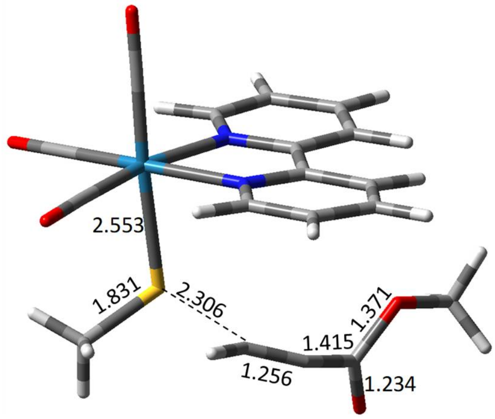

TS1*b*

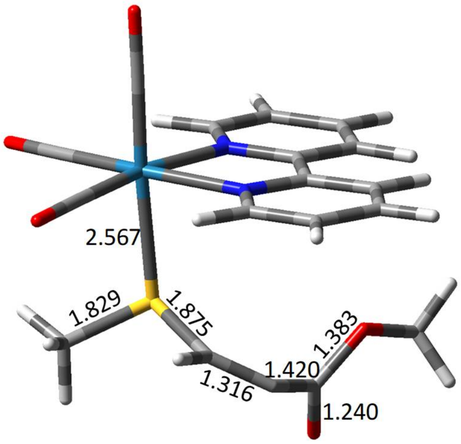

I1*b*

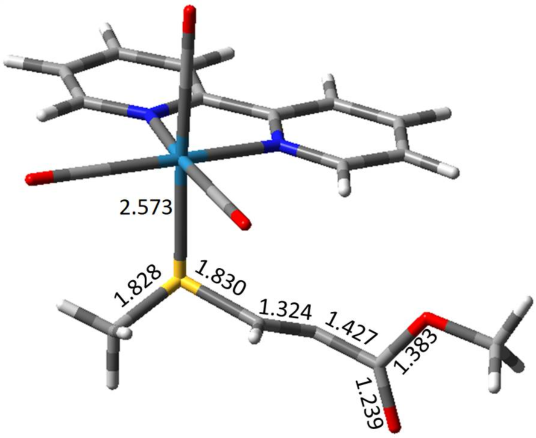

I1’*b*

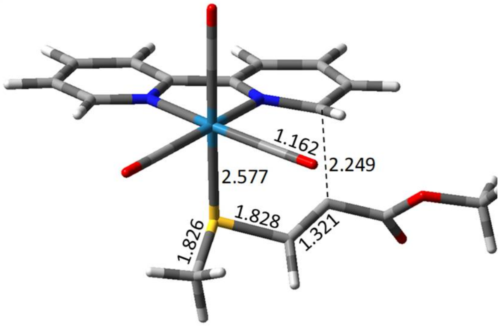

TS2*ccb*

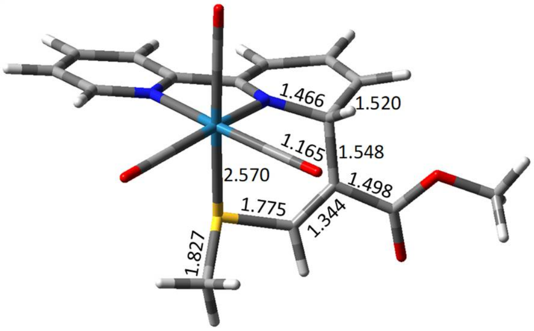

P*ccb*

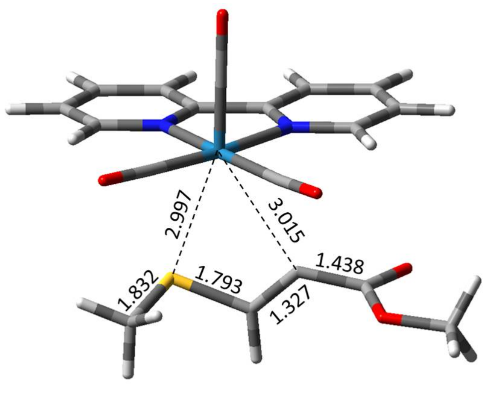

TS2*ins*

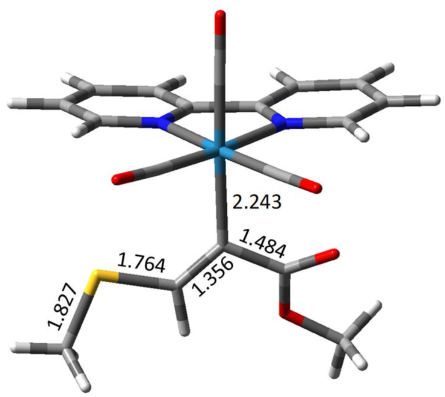

P*ins*

Table S31. PCM-B3LYP/6-31+G(d,p) (LANL2DZ for Re) energies without and with including thermal corrections (E and G, respectively), enthalpies (H), and entropies (S), and CPCM-DLPNO-CCSD(T)/def2-TZVPP//PCM-B3LYP/6-31+G(d,p) (LANL2DZ for Re) energies without and with including thermal corrections (E' and G', respectively) in THF solution of the critical structures involved in the reaction between the complex [Re(SPh)(CO)$_3$(bipy)] (bipy = 2,2'-bipyridine) and methyl propiolate (HMAD, HC≡CCO$_2$Me). All the values are given in hartree, except entropies that are in cal/K mol.[a]

Species	E	H	S	G	E'	G'[b]
[Re(SPh)(CO)$_3$(bipy)]	-1544.517318	-1544.212797	167.654	-1544.292454	-1541.206649	-1540.981785
HMAD	-305.215726	-305.136831	77.228	-305.173524	-304.738441	-304.696239
Reactants	-1849.733044	-1849.349628	244.882	-1849.465978	-1845.945090	-1845.678024
**TS1**	-1849.704532	-1849.320573	194.878	-1849.413166	-1845.920327	-1845.628961
**I1**	-1849.707892	-1849.322739	197.422	-1849.416541	-1845.927807	-1845.636456
**TS2*cco***	-1849.706710	-1849.322593	193.042	-1849.414313	-1845.924246	-1845.631849
**P*cco***	-1849.730807	-1849.344449	198.625	-1849.438822	-1845.947038	-1845.655053
**TS1*b***	-1849.705850	-1849.321682	197.287	-1849.415419	-1845.927613	-1845.637182
**I1*b***	-1849.706932	-1849.321500	201.175	-1849.417084	-1845.931873	-1845.642025
**I1'*b***	-1849.707902	-1849.322452	199.055	-1849.417030	-1845.929124	-1845.638252
**TS2*ccb***	-1849.699592	-1849.315636	197.010	-1849.409242	-1845.918949	-1845.628600
**P*ccb***	-1849.729014	-1849.343167	194.057	-1849.435369	-1845.957541	-1845.663896
**TS2*ins***	-1849.696649	-1849.312351	196.991	-1849.405948	-1845.913288	-1845.622587
**P*ins***	-1849.753435	-1849.367037	198.838	-1849.461511	-1845.979051	-1845.687127

[a] Thermal magnitudes were computed in THF solution at 298.15 K and 1 atm. [b] For each species, G' was calculated as G' = G – E + E', in which G is the PCM-B3LYP/6-31+G(d,p) (LANL2DZ for Re) energy with including thermal corrections and E and E' are the PCM-B3LYP/6-31+G(d,p) (LANL2DZ for Re) and CPCM-DLPNO-CCSD(T)/def2-TZVPP//PCM-B3LYP/6-31+G(d,p) (LANL2DZ for Re) energies without including thermal corrections, respectively.

Table S32. PCM-B3LYP/6-31+G(d,p) (LANL2DZ for Re) relative energies without and with including thermal corrections (ΔE and ΔG, respectively), enthalpies (ΔH), and entropic contributions (TΔS), and CPCM-DLPNO-CCSD(T)/def2-TZVPP//PCM-B3LYP/6-31+G(d,p) (LANL2DZ for Re) relative energies without and with including thermal corrections (E' and G', respectively) in THF solution of the critical structures involved in the reaction between the complex [Re(SPh)$(CO)_3$(bipy)] (bipy = 2,2'-bipyridine) and methyl propiolate (HMAD, HC≡CCO_2Me). All the values are given in kcal/mol.[a]

Species	ΔE	ΔH	TΔS	ΔG	ΔE'	ΔG'
[Re(SPh)$(CO)_3$(bipy)] + HMAD	0.0	0.0	0.0	0.0	0.0	0.0
**TS1**	17.9	18.2	-14.9	33.1	15.5	30.8
**I1**	15.8	16.9	-14.2	31.0	10.8	26.1
**TS2*cco***	16.5	17.0	-15.5	32.4	13.1	29.0
**P*cco***	1.4	3.2	-13.8	17.0	-1.2	14.4
**TS1*b***	17.1	17.5	-14.2	31.7	11.0	25.6
**I1*b***	16.4	17.7	-13.0	30.7	8.3	22.6
**I1'*b***	15.8	17.1	-13.7	30.7	10.0	25.0
**TS2*ccb***	21.0	21.3	-14.3	35.6	16.4	31.0
**P*ccb***	2.5	4.1	-15.2	19.2	-7.8	8.9
**TS2*ins***	22.8	23.4	-14.3	37.7	20.0	34.8
**P*ins***	-12.8	-10.9	-13.7	2.8	-21.3	-5.7

[a] Thermal magnitudes were evaluated in THF solution at 298.15 K and 1 atm.

Table S33. PCM-B3LYP/6-31+G(d,p) (LANL2DZ for Re) optimized cartesian coordinates, in Å, for the critical structures involved in the reaction of the complex [Re(SPh)$(CO)_3$(bipy)] (bipy = 2,2'-bipyridine) towards methyl propiolate (HMAD, HC≡CCO_2Me).

[Re(SPh)$(CO)_3$(bipy)]

N	-1.682559	-0.732467	0.973299
C	-2.505600	0.334012	1.147662
C	-3.466346	0.343994	2.164553
C	-3.584440	-0.753780	3.010557
C	-2.729811	-1.840439	2.828480
C	-1.796086	-1.789261	1.800394
C	-2.319887	1.453573	0.202027
C	-3.085928	2.622743	0.247033
C	-2.856845	3.630610	-0.683427
C	-1.865335	3.447384	-1.646000
C	-1.139554	2.261860	-1.638287
N	-1.353850	1.284037	-0.737855
Re	-0.175251	-0.590467	-0.637802
S	1.094247	0.704527	1.148348
C	-1.325981	-1.459295	-1.917078
O	-2.006841	-1.990634	-2.700697
C	0.724169	-2.246519	-0.265068
O	1.239477	-3.258246	-0.003018
C	1.097940	-0.182293	-2.022355
O	1.843415	0.097056	-2.871873
H	-1.113338	-2.609757	1.624782
H	-4.120386	1.195723	2.290709
H	-2.779126	-2.715512	3.463418
H	-4.327165	-0.756799	3.799357
H	-0.358630	2.081605	-2.365384
H	-3.850368	2.746489	1.002500
H	-1.651250	4.201723	-2.392216
H	-3.443090	4.540799	-0.656466
C	2.871495	0.621897	1.047720
C	3.603737	1.569567	1.793358
C	3.592329	-0.335476	0.311639
C	4.998432	1.554455	1.806756
H	3.067201	2.319402	2.365570
C	4.990310	-0.343754	0.324333
H	3.063041	-1.083697	-0.264803
C	5.705320	0.597421	1.069844
H	5.533135	2.296408	2.391656
H	5.520407	-1.095172	-0.252382
H	6.789333	0.585911	1.077759

TS1

Re	0.502483	0.109548	0.913943
C	1.474068	-0.558034	2.432555
O	2.039495	-0.973570	3.360014
N	2.335417	0.849441	-0.066315
C	2.978349	-0.011778	-0.898503
C	4.140922	0.374505	-1.574555
C	4.653416	1.654123	-1.388850
C	3.989131	2.528687	-0.529295
C	2.835715	2.088525	0.107346
C	2.377771	-1.354510	-1.028963
N	1.217824	-1.556698	-0.346700
C	0.606672	-2.753772	-0.431336
C	1.128398	-3.807480	-1.174174
C	2.324908	-3.617516	-1.860897
C	2.953171	-2.376674	-1.788167
C	-1.101472	-0.723237	1.626361
O	-2.010304	-1.233042	2.137533
C	0.100405	1.721477	1.883979
O	-0.092122	2.707393	2.472192
C	-2.280964	-1.430890	-1.178513
C	-2.861503	-2.626561	-0.802130
O	-4.078566	-2.459059	-0.162467
C	-4.682783	-3.658065	0.337283
O	-2.362312	-3.767456	-0.933806
H	-2.191660	-0.058194	-2.868829
H	-4.865198	-4.373936	-0.467297
H	-5.627335	-3.345511	0.781827
H	-4.051704	-4.129176	1.094864
H	-0.330554	-2.864670	0.093481
H	0.583730	-4.741042	-1.212457
H	2.763644	-4.416236	-2.446746
H	5.553970	1.962625	-1.906064
H	4.347828	3.534123	-0.351299
H	2.289366	2.734211	0.781776
H	4.638180	-0.315025	-2.242935
H	3.882217	-2.209676	-2.316205
S	-0.603978	0.902658	-1.261305
C	-1.927995	-0.416498	-1.876792
C	-1.656431	2.341837	-1.031308
C	-2.508737	2.467598	0.073855
C	-1.655870	3.327867	-2.027676
C	-3.335591	3.585270	0.190508
H	-2.524306	1.696344	0.835231
C	-2.492485	4.441758	-1.907759
H	-0.999813	3.225463	-2.884643
C	-3.332053	4.573873	-0.799076
H	-3.988125	3.678670	1.051398
H	-2.485477	5.202935	-2.681230
H	-3.977678	5.439953	-0.704444

I1

Re	0.629271	0.230876	0.877247
C	1.690708	-0.171396	2.425536
O	2.324170	-0.407031	3.372663
N	2.418068	0.503659	-0.385758
C	2.891783	-0.595081	-1.030716
C	4.027646	-0.517767	-1.843545
C	4.688453	0.697098	-1.991357
C	4.195026	1.818696	-1.325250
C	3.059575	1.679140	-0.537259
C	2.140021	-1.847132	-0.812892
N	1.045295	-1.751485	-0.012012
C	0.308436	-2.853430	0.226836
C	0.632580	-4.096931	-0.305788
C	1.754993	-4.206467	-1.122711
C	2.516537	-3.068756	-1.378002
C	-0.974261	-0.206385	1.881813
O	-1.879087	-0.436344	2.571005
C	0.475019	2.057803	1.457891
O	0.423530	3.171421	1.793224
C	-2.310910	-1.386127	-0.594791
C	-3.223485	-2.471108	-0.658803
O	-4.490326	-2.143686	-0.225516
C	-5.404246	-3.239328	-0.075858
O	-2.937942	-3.646278	-0.945271
H	-2.375490	-0.420162	-2.552298
H	-5.552371	-3.758000	-1.025697
H	-6.340325	-2.796026	0.261070
H	-5.038014	-3.954549	0.664746
H	-0.575611	-2.711629	0.830962
H	0.002334	-4.948547	-0.086851
H	2.035235	-5.158121	-1.557995
H	5.570995	0.766214	-2.615043
H	4.673871	2.785985	-1.409927
H	2.643879	2.521905	-0.000782
H	4.392963	-1.398840	-2.353083
H	3.389626	-3.131899	-2.012174
S	-0.656010	0.732128	-1.305239
C	-1.988972	-0.523863	-1.536963
C	-1.610264	2.252928	-1.092379
C	-2.630450	2.329707	-0.137063
C	-1.334851	3.341910	-1.924795
C	-3.371008	3.505710	-0.014317
H	-2.848206	1.472409	0.490349
C	-2.079929	4.519206	-1.793184
H	-0.551388	3.268384	-2.671432
C	-3.099427	4.600593	-0.842516
H	-4.162300	3.565413	0.725524
H	-1.868472	5.362541	-2.441640
H	-3.680219	5.511215	-0.745744

TS2*cco*

C	4.269055	-0.333171	-1.329708
C	3.011926	-0.535870	-0.749354

N	2.414950	0.439435	-0.013495
C	3.059745	1.610619	0.160810
C	4.311829	1.869428	-0.382980
C	4.927446	0.878191	-1.147120
C	2.254601	-1.796534	-0.886711
N	1.023749	-1.817865	-0.308790
C	0.272036	-2.931550	-0.409611
C	0.714014	-4.075058	-1.066213
C	1.979041	-4.067456	-1.650489
C	2.754705	-2.915755	-1.559506
Re	0.432857	-0.012053	0.842479
S	-0.494487	0.829819	-1.413491
C	-1.982079	-0.203128	-1.677973
C	-2.354218	-1.061387	-0.750785
C	-3.380231	-2.048577	-0.741724
O	-3.236268	-3.240521	-1.052853
C	0.148949	1.662674	1.724189
O	0.014759	2.689230	2.263880
C	1.174653	-0.706676	2.458186
O	1.611126	-1.129260	3.454765
C	-1.356224	-0.612646	1.443673
O	-2.245903	-0.904219	2.142266
O	-4.548732	-1.594870	-0.188938
C	-5.569602	-2.584017	0.023884
H	-2.401625	-0.023007	-2.667438
H	-5.868536	-3.044726	-0.919401
H	-6.408037	-2.046289	0.463653
H	-5.218333	-3.360977	0.705931
H	2.356646	-4.939899	-2.170243
H	5.901951	1.042377	-1.590954
H	4.781999	2.828817	-0.208584
H	2.547287	2.351908	0.759101
H	4.728974	-1.111362	-1.922958
H	3.739071	-2.892276	-2.007123
H	0.069727	-4.942561	-1.117116
H	-0.716004	-2.882230	0.028091
C	-1.189108	2.482769	-1.188589
C	-0.495535	3.575960	-1.717625
C	-2.386124	2.670331	-0.488926
C	-0.999936	4.867456	-1.534696
H	0.425121	3.421042	-2.269951
C	-2.890159	3.961652	-0.325337
H	-2.911516	1.812658	-0.082746
C	-2.197463	5.061692	-0.841688
H	-0.460539	5.715909	-1.941287
H	-3.821017	4.107114	0.212313
H	-2.592329	6.062835	-0.708589

P*cco*

C	3.355215	-2.445080	-1.691321
C	2.584351	-1.502643	-1.000392
N	1.402765	-1.852117	-0.424970
C	0.983471	-3.130418	-0.511404
C	1.704586	-4.111737	-1.181517
C	2.911595	-3.760141	-1.785863
C	3.000052	-0.099492	-0.809614
N	2.182947	0.655152	-0.024543
C	2.527244	1.937307	0.218717
C	3.677779	2.521175	-0.296159
C	4.513051	1.756334	-1.112344
C	4.166563	0.434657	-1.370411
Re	0.309253	-0.280751	0.704993
C	-0.446242	1.188769	1.640456
O	-0.866109	2.113307	2.235790
S	-0.828513	0.305694	-1.505169
C	-2.428952	-0.409949	-1.172610
C	-2.579260	-1.185363	-0.093736
C	-3.856281	-1.939909	0.114920
O	-4.164613	-2.952075	-0.490854
C	-1.244640	2.046461	-1.729830
C	-0.637824	2.727307	-2.790688
C	-0.922929	4.080816	-2.990572
C	-1.819163	4.741714	-2.146647
C	-2.428068	4.050018	-1.094609
C	-2.140099	2.701309	-0.877142
C	-1.436789	-1.434250	0.920224
O	-1.670304	-2.376032	1.700609
C	0.960210	-0.951345	2.348593
O	1.323097	-1.386339	3.377058
O	-4.613928	-1.379674	1.067121
C	-5.837378	-2.075154	1.398115
H	-3.189529	-0.234877	-1.925368
H	-6.485367	-2.131271	0.522598
H	-6.300701	-1.485338	2.185156
H	-5.609014	-3.080441	1.751849
H	3.503885	-4.497571	-2.314386
H	5.414171	2.179590	-1.538826
H	3.904997	3.553446	-0.061765
H	1.847957	2.495627	0.849578
H	4.796375	-0.175207	-2.003967
H	4.299518	-2.161056	-2.136542
H	1.324013	-5.124455	-1.218505
H	0.049347	-3.355707	-0.012622
H	0.041030	2.204114	-3.454816
H	-2.599608	2.168290	-0.053095
H	-0.454859	4.610816	-3.812611
H	-3.123853	4.559383	-0.437025
H	-2.043743	5.790222	-2.307756

TS1*b*

C	2.027123	-0.935503	2.263274
C	1.036118	-0.313051	1.497361
N	-0.083351	-0.988526	1.125459
C	-0.225456	-2.278216	1.487734
C	0.733905	-2.957720	2.227348
C	1.878628	-2.269420	2.628676
C	1.132412	1.082403	1.023862
C	2.219586	1.914039	1.307435
C	2.250684	3.205243	0.792786
C	1.182716	3.646940	0.012706
C	0.124447	2.778197	-0.220148
N	0.094899	1.522076	0.264868
Re	-1.590844	0.136000	-0.027489
C	-2.979064	-1.202834	-0.035881
O	-3.794977	-2.030490	-0.014100
C	-0.909325	-0.504952	-1.708859
O	-0.514549	-0.899406	-2.730069
S	-2.330047	0.809193	2.347394
C	-4.048083	1.316603	2.383300
C	-2.741030	1.356195	-0.970413
O	-3.390578	2.123193	-1.557528
C	-1.367097	2.651390	2.962756
C	-0.481387	2.672948	3.874607
C	0.202026	1.893164	4.838812
O	1.434721	1.467608	4.407550
C	2.211355	0.757609	5.385044
O	-0.189805	1.654344	5.986037
H	-1.866416	3.354253	2.315782
H	3.143896	0.495215	4.885802
H	2.412182	1.390019	6.251863
H	1.691596	-0.143906	5.713960
H	-1.134539	-2.768036	1.165324
H	0.575723	-3.997055	2.484460
H	2.644506	-2.762476	3.215727
H	2.902765	-0.382860	2.574841
H	3.028354	1.557608	1.928834
H	3.090439	3.856603	1.002262
H	1.159335	4.643888	-0.408328
H	-0.726187	3.082929	-0.814800
C	-4.523887	2.408610	1.641166
C	-5.859570	2.802733	1.752403
C	-6.731011	2.116310	2.602842
C	-6.257681	1.033370	3.349083
C	-4.922209	0.637962	3.246660
H	-3.860842	2.950758	0.977764
H	-6.217327	3.647890	1.173981
H	-7.767998	2.424115	2.684282
H	-6.924393	0.496386	4.015166
H	-4.552089	-0.196155	3.831425

I1*b*

Re	-1.385779	-0.120255	-0.344443
C	-2.316649	-1.618649	-1.114228
O	-2.862041	-2.554183	-1.540543
N	-0.138653	-1.278294	1.046369
C	-0.236700	-2.612010	1.209931
C	0.553696	-3.315369	2.109584
C	1.494643	-2.615036	2.863151
C	1.611843	-1.241119	2.685440
C	0.783678	-0.589572	1.767680
C	0.822891	0.868435	1.538389
N	-0.091129	1.356926	0.658376
C	-0.132742	2.682875	0.422707
C	0.723010	3.581643	1.046892
C	1.672668	3.089109	1.941469
C	1.722893	1.720823	2.185078
C	-2.786937	0.095097	0.958656
O	-3.638571	0.234156	1.737113
H	2.340198	-0.682156	3.255536
H	2.129957	-3.129799	3.573619

H	0.430535	-4.386222	2.207992
H	-0.978995	-3.116361	0.605683
H	-0.878821	3.022264	-0.283506
H	0.643561	4.638054	0.825831
H	2.364167	3.757261	2.440396
H	2.455279	1.319225	2.871353
H	4.821598	1.550901	0.164300
C	-2.290839	1.073518	-1.562646
O	-2.815727	1.810389	-2.291771
S	0.631863	-0.214122	-1.942492
C	1.663904	-1.782727	-1.595015
C	2.907303	-1.742668	-1.186951
C	3.816819	-0.681156	-0.941601
O	3.814494	-0.271626	0.377387
O	4.632819	-0.208533	-1.746118
C	4.866470	0.629757	0.748058
H	4.708899	0.847571	1.804018
H	5.843783	0.163276	0.603542
H	1.047072	-2.656378	-1.777212
C	0.088991	-0.450822	-3.642659
C	0.437128	0.531733	-4.578199
C	0.077510	0.375223	-5.919401
C	-0.615993	-0.765163	-6.332274
C	-0.953915	-1.748841	-5.397385
C	-0.610389	-1.593434	-4.052668
H	0.990947	1.406458	-4.257148
H	0.347305	1.141533	-6.637846
H	-0.889929	-0.889101	-7.373796
H	-1.489789	-2.637592	-5.712690
H	-0.881744	-2.358853	-3.335987

I1'*b*

C	2.871289	-1.708890	-1.028953
N	1.571460	-1.716181	-0.672341
C	0.717967	-2.566634	-1.302028
C	1.173595	-3.432901	-2.301216
C	2.516623	-3.422706	-2.662772
C	3.383313	-2.542263	-2.015609
Re	0.717263	-0.454264	0.922463
S	0.042893	1.143393	-0.993178
C	-1.635202	1.764883	-0.516680
C	-2.705111	1.270194	-1.106170
C	-4.052989	1.628658	-0.817852
O	-4.631599	0.816715	0.141225
C	-6.032246	1.033881	0.369492
C	-0.691142	-2.509102	-0.866080
N	-0.973299	-1.617484	0.123318
C	-2.247912	-1.474838	0.530484
C	-3.290497	-2.234687	0.009783
C	-3.006801	-3.169147	-0.980843
C	-1.694439	-3.298600	-1.430275
C	1.112821	-1.766625	2.273494
O	1.352476	-2.546751	3.100884
C	-0.253228	0.603602	2.211319
O	-0.850509	1.234261	2.983531
C	2.336911	0.497561	1.358298
O	3.332629	1.048829	1.595605
O	-4.732350	2.485674	-1.400903
H	-1.541736	2.494941	0.290602
H	-6.217282	2.044642	0.739167
H	-6.325716	0.300439	1.119780
H	-6.604042	0.885490	-0.549222
H	-2.434474	-0.723191	1.284225
H	-4.296157	-2.064380	0.368522
H	-3.792217	-3.778165	-1.412069
H	2.878178	-4.090655	-3.435214
H	4.436068	-2.496343	-2.261580
H	3.509142	-1.014385	-0.498934
H	0.485525	-4.107416	-2.791887
H	-1.456805	-4.002968	-2.215459
C	1.039747	2.643097	-0.947346
C	1.640104	3.060844	-2.141178
C	1.179345	3.408791	0.217201
C	2.381904	4.244971	-2.168286
H	1.519191	2.468329	-3.040682
C	1.911795	4.597096	0.178801
H	0.723572	3.082708	1.145580
C	2.517966	5.014362	-1.010082
H	2.848314	4.564081	-3.093979
H	2.014204	5.190618	1.080278
H	3.089075	5.935800	-1.032738

TS2*ccb*

C	2.820573	-2.032532	-0.846575
N	1.505575	-1.872516	-0.589134
C	0.599827	-2.657734	-1.232390
C	1.023928	-3.628134	-2.149813
C	2.378913	-3.785815	-2.413156
C	3.298873	-2.971200	-1.749326
Re	0.700887	-0.430142	0.870049
S	0.210575	1.047413	-1.206253
C	-1.511394	1.676206	-1.048700
C	-2.535975	0.892241	-0.768906
C	-3.917970	1.284890	-0.750268
O	-4.398234	1.404631	0.527305
C	-5.799742	1.705753	0.648956
C	-0.817690	-2.412113	-0.902568
N	-1.037213	-1.469049	0.064385
C	-2.319232	-1.054745	0.303430
C	-3.412970	-1.862040	-0.141518
C	-3.182343	-2.859737	-1.051932
C	-1.861236	-3.106879	-1.493738
C	0.989904	-1.639609	2.330248
O	1.170154	-2.360318	3.225565
C	-0.236465	0.777467	2.044528
O	-0.818269	1.496770	2.749124
C	2.366050	0.463567	1.269944
O	3.386714	0.983438	1.477274
O	-4.665670	1.379430	-1.727069
H	-1.526443	2.739986	-1.287757
H	-6.032232	2.659426	0.171454
H	-5.994139	1.762458	1.718658
H	-6.404774	0.920544	0.191775
H	-2.455753	-0.444888	1.185517
H	-4.408894	-1.627393	0.213036
H	-3.998049	-3.462269	-1.434843
H	2.712325	-4.532373	-3.124202
H	4.364149	-3.056530	-1.921701
H	3.494156	-1.382184	-0.303885
H	0.298257	-4.255065	-2.649391
H	-1.672133	-3.860073	-2.245565
C	1.137448	2.583196	-1.071676
C	2.065513	2.876237	-2.076334
C	0.936237	3.470779	-0.007725
C	2.807434	4.059490	-2.007941
H	2.200539	2.188913	-2.903500
C	1.671903	4.656200	0.044838
H	0.218261	3.238086	0.770182
C	2.608707	4.950556	-0.950920
H	3.529781	4.284730	-2.784510
H	1.515313	5.346026	0.866597
H	3.181241	5.870777	-0.902280

P*ccb*

C	3.481191	-0.652174	-0.827149
N	2.191499	-1.004338	-0.627520
C	1.633400	-1.980450	-1.396017
C	2.398340	-2.620646	-2.384573
C	3.719346	-2.251311	-2.589344
C	4.277112	-1.243679	-1.794128
Re	0.897305	-0.138622	0.927174
S	-0.210949	1.039623	-1.104824
C	-1.942998	0.658831	-0.931033
C	-2.497094	-0.463438	-0.441866
C	-1.767000	-1.684316	0.162638
N	-0.315088	-1.626164	-0.044347
C	0.223323	-2.304648	-1.104800
C	-0.456147	-3.281439	-1.806731
C	-1.730535	-3.703003	-1.295409
C	-2.343467	-3.008264	-0.312081
C	1.654553	-1.247305	2.287799
O	2.110783	-1.930658	3.112851
C	-0.424770	0.496036	2.174526
O	-1.233299	0.872473	2.924313
C	2.074566	1.314185	1.426940
O	2.796530	2.190724	1.694292
C	-3.993814	-0.425426	-0.399970
O	-4.490853	-1.211677	0.570945
C	-5.929904	-1.258424	0.677132
O	-4.700523	0.248545	-1.134511
H	-2.582621	1.452227	-1.303387
H	-6.329793	-0.257779	0.841350
H	-6.134927	-1.902944	1.528369
H	-6.356160	-1.677223	-0.235645
H	-1.951249	-1.603480	1.242458
H	-3.254547	-3.357527	0.156126
H	-2.167599	-4.627919	-1.661237
H	4.310254	-2.741838	-3.353995
H	5.303404	-0.923456	-1.916613
H	3.868327	0.126137	-0.182636

H	1.955466	-3.405345	-2.982146
H	0.000567	-3.804273	-2.634068
C	-0.267355	2.842110	-1.056449
C	-0.126252	3.530708	-2.266089
C	-0.414814	3.536510	0.149747
C	-0.138646	4.928028	-2.267734
H	-0.008496	2.980464	-3.192485
C	-0.424589	4.932710	0.137171
H	-0.514162	2.997194	1.084861
C	-0.286403	5.628543	-1.068117
H	-0.032009	5.464320	-3.204290
H	-0.536224	5.475248	1.069420
H	-0.292044	6.712314	-1.070567

TS2*ins*

C	-2.306046	-3.430388	-1.380073
C	-1.929513	-2.211341	-0.803349
N	-0.807197	-2.110380	-0.046091
C	-0.042800	-3.205811	0.136807
C	-0.357211	-4.440945	-0.414406
C	-1.514196	-4.556312	-1.186403
C	-2.705011	-0.967349	-0.966167
C	-3.866358	-0.891623	-1.741438
C	-4.549289	0.316032	-1.840937
C	-4.046762	1.429712	-1.169819
C	-2.878890	1.295015	-0.428675
N	-2.221736	0.123897	-0.313656
Re	-0.406355	-0.153378	0.929182
C	-1.397206	-0.651056	2.446877
O	-2.019056	-0.935599	3.398149
C	1.219080	-0.657121	1.818435
O	2.199438	-1.017536	2.334044
C	-0.221623	1.651983	1.600768
O	-0.159702	2.705397	2.090218
C	0.354344	2.122957	-0.926216
C	1.181340	1.480776	-1.741233
S	1.404974	-0.280362	-1.490725
C	-0.073412	3.492747	-1.024312
O	0.796304	4.360083	-0.425772
C	0.352142	5.722399	-0.312800
O	-1.162600	3.878339	-1.466515
H	-2.440511	2.144139	0.075715
H	-4.535347	2.394284	-1.224269
H	-5.452933	0.385493	-2.434077
H	-4.231829	-1.765965	-2.262420
H	0.839956	-3.075439	0.748856
H	0.292062	-5.288422	-0.235736
H	-1.795019	-5.504581	-1.628427
H	-3.207694	-3.497476	-1.972914
H	1.694487	1.869701	-2.622016
H	0.201128	6.163470	-1.300034
H	-0.581415	5.776143	0.250592
H	1.146419	6.245307	0.217303
C	3.146765	-0.456156	-1.079110
C	3.800467	-1.630772	-1.474000
C	3.842540	0.526782	-0.363334
C	5.146733	-1.821898	-1.153653
H	3.261006	-2.382514	-2.039714
C	5.192202	0.334405	-0.060213
H	3.332745	1.433337	-0.056827
C	5.847263	-0.838076	-0.450596
H	5.647477	-2.731681	-1.466803
H	5.730652	1.100489	0.487515
H	6.895987	-0.980993	-0.213513

P*ins*

C	-2.418448	-2.869715	-1.714050
C	-2.150004	-1.774486	-0.885899
N	-1.144967	-1.815678	0.027245
C	-0.416361	-2.941941	0.145303
C	-0.635770	-4.064760	-0.644220
C	-1.649435	-4.023124	-1.600579
C	-2.946055	-0.532399	-0.915675
C	-4.024184	-0.343346	-1.785330
C	-4.749924	0.842243	-1.736049
C	-4.378654	1.818305	-0.813427
C	-3.294138	1.572310	0.019274
N	-2.590641	0.425078	-0.019739
Re	-0.852255	-0.009092	1.274238
C	-1.999985	-0.751372	2.667015
O	-2.675153	-1.176819	3.515424
C	0.713367	-0.572773	2.245326
O	1.634664	-0.924653	2.864821
C	-0.791402	1.672240	2.202289
O	-0.793435	2.696026	2.760501
C	0.397006	0.832159	-0.388244
C	1.519713	0.398731	-0.991140
S	2.323657	-1.160093	-0.683743
C	-0.095557	2.121556	-0.941484
O	0.407876	3.190536	-0.276859
C	-0.045220	4.485556	-0.719373
O	-0.895740	2.252898	-1.859964
H	-2.971556	2.306198	0.746024
H	-4.910711	2.757186	-0.736009
H	-5.588439	0.998637	-2.405262
H	-4.293589	-1.114755	-2.493145
H	0.364782	-2.923972	0.893117
H	-0.016546	-4.942454	-0.510493
H	-1.846314	-4.875649	-2.239344
H	-3.219437	-2.821302	-2.439073
H	2.002488	0.961511	-1.790439
H	0.242725	4.654352	-1.758171
H	-1.129667	4.559939	-0.629399
H	0.443740	5.204364	-0.065370
C	4.053542	-0.746958	-0.858737
C	4.945054	-1.802281	-1.113700
C	4.551664	0.556803	-0.721496
C	6.313480	-1.554445	-1.225895
H	4.564275	-2.811690	-1.228272
C	5.922671	0.797659	-0.853623
H	3.879083	1.377923	-0.502473
C	6.809606	-0.251910	-1.102842
H	6.991369	-2.379971	-1.420239
H	6.294699	1.811291	-0.749142
H	7.872016	-0.058962	-1.198603

Figure S11. PCM-B3LYP/6-31+G(d,p) (LANL2DZ for Re) optimized geometries in THF solution of the critical structures involved in the reaction between the complex [Re(SPh)(CO)$_3$(bipy)] (bipy = 2,2'-bipyridine) and methyl propiolate (HMAD, HC≡CCO$_2$Me). Relevant distances are given in angstroms.

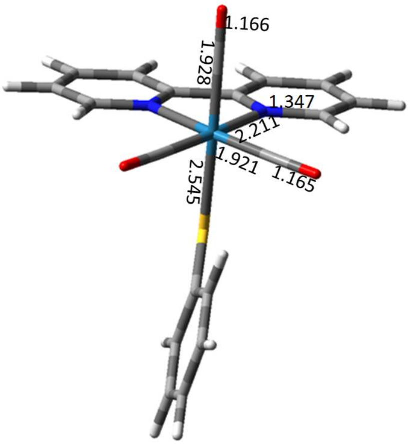

[Re(SPh)(CO)$_3$(bipy)]

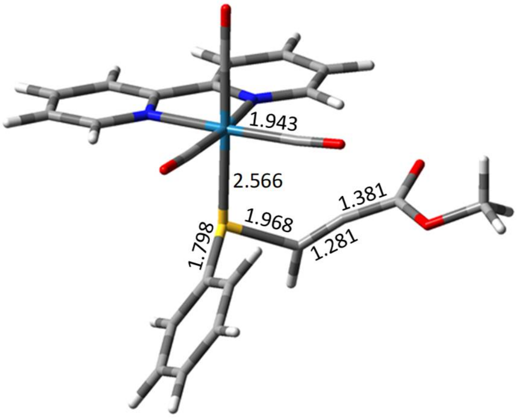

TS1

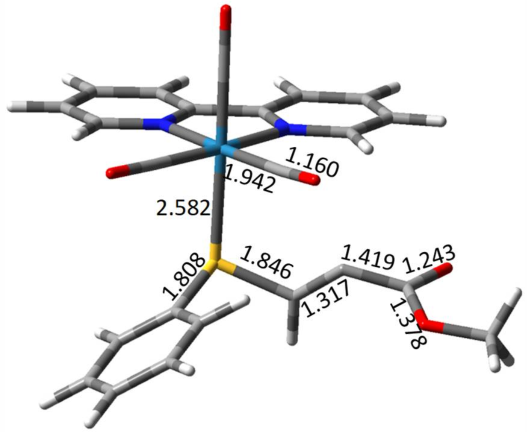

I1

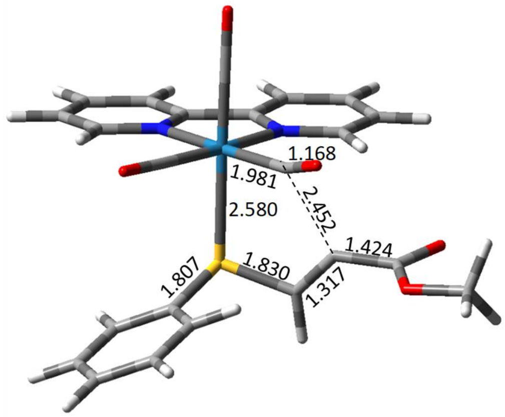

TS2*cco*

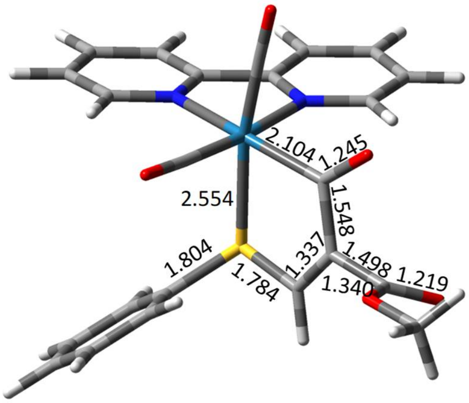

P*cco*

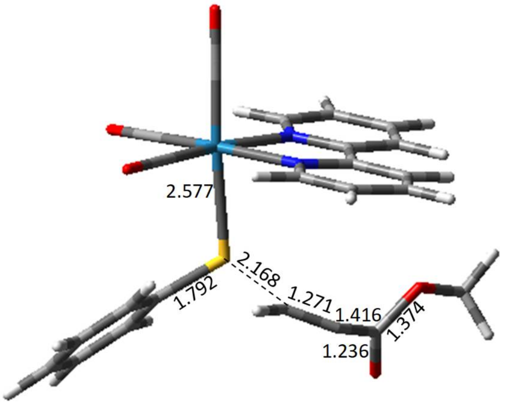

TS1*b*

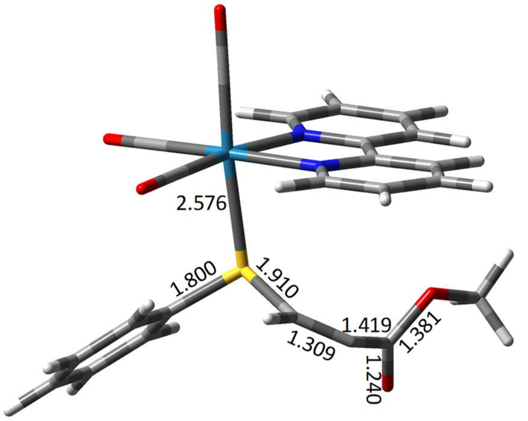

I1*b*

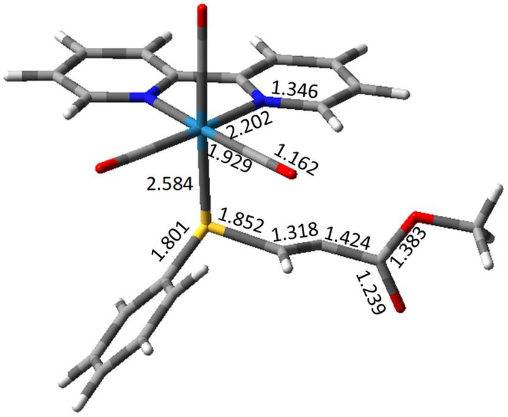

I1'*b*

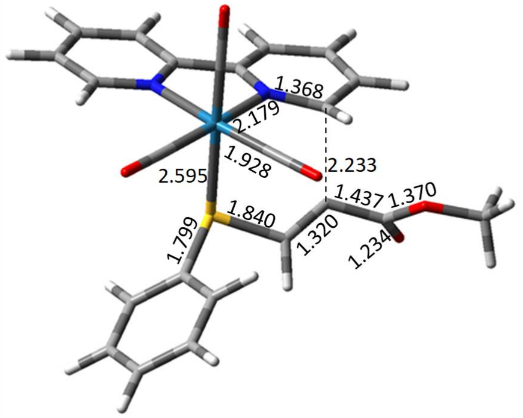

TS2*ccb*

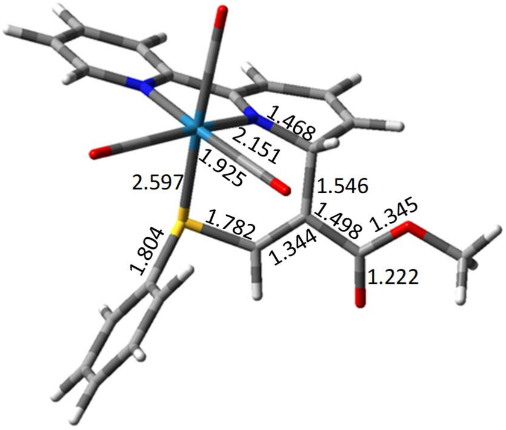

P*ccb*

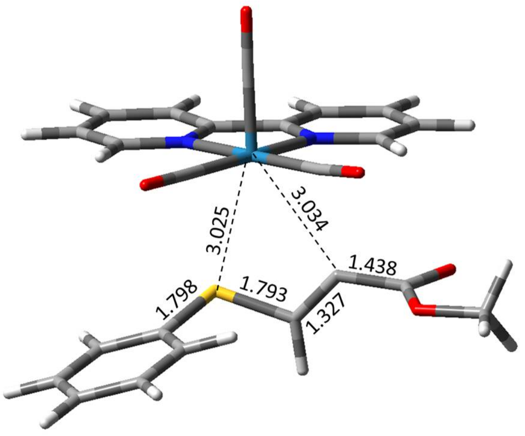

TS2*ins*

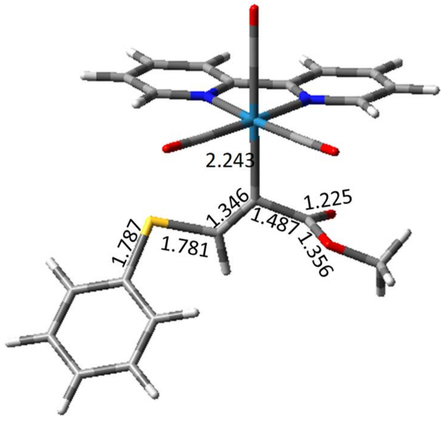

P*ins*

Table S34. PCM-B3LYP/6-31+G(d,p) (LANL2DZ for Re) energies without and with including thermal corrections (E and G, respectively), enthalpies (H), and entropies (S), and CPCM-DLPNO-CCSD(T)/def2-TZVPP//PCM-B3LYP/6-31+G(d,p) (LANL2DZ for Re) energies without and with including thermal corrections (E' and G', respectively) in THF solution of the critical structures involved in the reaction between the complex [Re(NH*p*Tol)$(CO)_3$(bipy)] (bipy = 2,2'-bipyridine) and methyl propiolate (HMAD, HC≡CCO_2Me). All the values are given in hartree, except entropies that are in cal/K mol.[a]

Species	E	H	S	G	E'	G'[b]
[Re(NH*p*Tol)$(CO)_3$(bipy)]	-1240.978786	-1240.631532	173.326	-1240.713884	-1237.965433	-1237.700531
HMAD	-305.215726	-305.136831	77.228	-305.173524	-304.738441	-304.696239
Reactants	-1546.194512	-1545.768363	250.554	-1545.887408	-1542.703874	-1542.396770
**TS1**	-1546.175707	-1545.747959	204.891	-1545.845310	-1542.691548	-1542.361151
**I1**	-1546.187683	-1545.756870	199.801	-1545.851802	-1542.708457	-1542.372576
**TS1'**	-1546.185687	-1545.756465	196.622	-1545.849886	-1542.705475	-1542.369674
**P*cco***	-1546.220617	-1545.788478	197.137	-1545.882144	-1542.735607	-1542.397134
**P*ccoh***	-1546.264387	-1545.832367	194.555	-1545.924806	-1542.770000	-1542.430419
**TS1*b***	-1546.175651	-1545.748042	203.887	-1545.844915	-1542.693438	-1542.362702
**I1*b***	-1546.193132	-1545.762664	205.023	-1545.860077	-1542.716119	-1542.383064
**I1'*b***	-1546.197826	-1545.766373	198.514	-1545.860694	-1542.718239	-1542.381107
**TS2*ccb***	-1546.186256	-1545.756872	194.994	-1545.849520	-1542.703186	-1542.366450
**P*ccb***	-1546.218806	-1545.787138	194.712	-1545.879652	-1542.744076	-1542.404922
**TS2*ins***	-1546.185872	-1545.758129	194.790	-1545.850680	-1542.699519	-1542.364327
**P*ins***	-1546.253169	-1545.821787	208.131	-1545.920677	-1542.764937	-1542.432445

[a] Thermal magnitudes were computed in THF solution at 298.15 K and 1 atm. [b] For each species, G' was calculated as G' = G – E + E', in which G is the PCM-B3LYP/6-31+G(d,p) (LANL2DZ for Re) energy with including thermal corrections and E and E' are the PCM-B3LYP/6-31+G(d,p) (LANL2DZ for Re) and CPCM-DLPNO-CCSD(T)/def2-TZVPP//PCM-B3LYP/6-31+G(d,p) (LANL2DZ for Re) energies without including thermal corrections, respectively.

Table S35. PCM-B3LYP/6-31+G(d,p) (LANL2DZ for Re) relative energies without and with including thermal corrections (ΔE and ΔG, respectively), enthalpies (ΔH), and entropic contributions (TΔS), and CPCM-DLPNO-CCSD(T)/def2-TZVPP//PCM-B3LYP/6-31+G(d,p) (LANL2DZ for Re) relative energies without and with including thermal corrections (E' and G', respectively) in THF solution of the critical structures involved in the reaction between the complex [Re(NH*p*Tol)$(CO)_3$(bipy)] (bipy = 2,2'-bipyridine) and methyl propiolate (HMAD, HC≡CCO_2Me). All the values are given in kcal/mol.[a]

Species	ΔE	ΔH	TΔS	ΔG	ΔE'	ΔG'
[Re(NH*p*Tol)$(CO)_3$(bipy)] + HMAD	0.0	0.0	0.0	0.0	0.0	0.0
**TS1**	11.8	12.8	-13.6	26.4	7.7	22.4
**I1**	4.3	7.2	-15.1	22.3	-2.9	15.2
**TS1'**	5.5	7.5	-16.1	23.5	-1.0	17.0
**P*cco***	-16.4	-12.6	-15.9	3.3	-19.9	-0.2
**P*ccoh***	-43.8	-40.2	-16.7	-23.5	-41.5	-21.1
**TS1*b***	11.8	12.8	-13.9	26.7	6.5	21.4
**I1*b***	0.9	3.6	-13.6	17.2	-7.7	8.6
**I1'*b***	-2.1	1.2	-15.5	16.8	-9.0	9.8
**TS2*ccb***	5.2	7.2	-16.6	23.8	0.4	19.0
**P*ccb***	-15.2	-11.8	-16.6	4.9	-25.2	-5.1
**TS2*ins***	5.4	6.4	-16.6	23.0	2.7	20.4
**P*ins***	-36.8	-33.5	-12.6	-20.9	-38.3	-22.4

[a] Thermal magnitudes were evaluated in THF solution at 298.15 K and 1 atm.

Table S36. PCM-B3LYP/6-31+G(d,p) (LANL2DZ for Re) optimized cartesian coordinates, in Å, for the critical structures involved in the reaction of the complex [Re(NH*p*Tol)(CO)$_3$(bipy)] (bipy = 2,2'-bipyridine) towards methyl propiolate (HMAD, HC≡CCO$_2$Me).

[Re(NH*p*Tol)(CO)$_3$(bipy)]

Re	-2.867449	1.872962	-0.808532
N	-2.270969	4.015881	-0.728136
C	-2.290883	4.621400	0.487060
C	-2.734389	3.780598	1.620210
N	-3.065163	2.500394	1.313425
C	-3.474005	1.675909	2.294832
C	-3.580157	2.082175	3.620352
C	-3.250646	3.397591	3.944775
C	-2.824344	4.253637	2.933642
C	-1.897891	4.726234	-1.808278
C	-1.526933	6.064267	-1.734684
C	-1.536411	6.692921	-0.490061
C	-1.921146	5.962995	0.630532
C	-3.404874	0.047315	-0.573904
O	-3.762085	-1.046407	-0.378494
C	-2.536789	1.565552	-2.675284
O	-2.323595	1.427192	-3.813315
C	-4.700668	2.363776	-1.201170
O	-5.799675	2.676763	-1.444365
N	-0.818737	1.556593	-0.248382
C	0.147468	0.651167	-0.608509
H	-0.419283	2.296544	0.320896
C	-0.124731	-0.492565	-1.401896
C	0.882741	-1.396664	-1.745363
C	2.211834	-1.234910	-1.330697
C	2.491267	-0.102115	-0.546434
C	1.501289	0.809653	-0.193432
H	-1.135612	-0.668894	-1.750170
H	0.625254	-2.257141	-2.359068
C	3.288619	-2.229574	-1.699089
H	3.509360	0.072204	-0.205445
H	1.761257	1.674800	0.412366
H	2.910896	-2.961986	-2.417813
H	4.159820	-1.740698	-2.149677
H	3.651978	-2.785818	-0.826399
H	-1.934069	6.432344	1.604865
H	-1.248991	7.732715	-0.391303
H	-1.237368	6.590342	-2.635316
H	-1.903093	4.196728	-2.751952
H	-3.717670	0.664448	1.997119
H	-3.913601	1.378002	4.372174
H	-3.323009	3.753209	4.965549
H	-2.566231	5.278060	3.165038

TS1

C	2.458569	-1.870630	-1.802507
C	2.105395	-0.891670	-0.854645
C	3.089405	-0.486368	0.065288
C	4.361054	-1.055607	0.044150
C	4.716235	-2.038703	-0.893707
C	3.737821	-2.431809	-1.816286
N	0.835610	-0.265393	-0.888968
Re	-0.480583	-0.063089	0.897597
C	0.726471	-0.926561	2.114896
O	1.419994	-1.490888	2.862236
C	6.106141	-2.628979	-0.922254
N	-1.966394	0.589022	-0.615832
C	-2.724732	-0.384907	-1.189525
C	-3.733112	-0.072140	-2.105058
C	-3.965796	1.258444	-2.442229
C	-3.174742	2.246650	-1.861976
C	-2.184637	1.874030	-0.956716
C	-2.420723	-1.771841	-0.778083
N	-1.419592	-1.915579	0.129605
C	-1.090540	-3.152361	0.551514
C	-1.739447	-4.295673	0.102434
C	-2.772406	-4.158211	-0.824433
C	-3.109549	-2.884789	-1.272102
C	0.245603	1.634495	1.461325
O	0.661333	2.638998	1.876171
C	-1.792868	0.050743	2.307321
O	-2.595253	0.102769	3.150648
C	1.167104	1.317345	-2.061581
C	1.129694	2.511286	-1.689257
C	0.946375	3.696027	-0.960111
O	2.104282	4.153106	-0.398773
C	1.981957	5.332735	0.412883
H	1.405555	0.694249	-2.906881
O	-0.123899	4.311903	-0.836663
H	0.280046	-0.760063	-1.586813
H	2.850318	0.283014	0.790977
H	5.099201	-0.729348	0.771534
H	3.975438	-3.193301	-2.554240
H	1.717079	-2.201506	-2.524989
H	6.610168	-2.495447	0.038489
H	6.080599	-3.699404	-1.146663
H	6.728995	-2.152160	-1.688049
H	1.319332	5.142969	1.259674
H	1.595758	6.167831	-0.173882
H	2.990772	5.549750	0.760580
H	-1.542374	2.623433	-0.510280
H	-3.308251	3.294584	-2.098790
H	-4.747348	1.513210	-3.148117
H	-3.305737	-5.025651	-1.194249
H	-1.438825	-5.264663	0.479704
H	-0.288365	-3.213991	1.275059
H	-4.332094	-0.855822	-2.548125
H	-3.905206	-2.758510	-1.993373

I1

C	-4.054000	0.832920	-1.531396
C	-2.978448	0.225735	-0.879580
N	-2.008977	0.966713	-0.275850
C	-2.080418	2.310124	-0.335665
C	-3.130176	2.971851	-0.968723
C	-4.137856	2.222909	-1.568398
C	-2.814225	-1.239781	-0.802135
N	-1.730693	-1.679245	-0.106894
C	-1.521796	-3.006947	0.010155
C	-2.365392	-3.953175	-0.557742
C	-3.477987	-3.511413	-1.273490
C	-3.701694	-2.144135	-1.395646
Re	-0.449830	-0.143662	0.838550
C	-1.515900	-0.133160	2.434252
O	-2.161079	-0.128947	3.403029
N	0.700647	-0.223188	-1.167262
C	0.678346	0.965578	-2.133651
C	0.947413	2.225531	-1.894733
C	1.280974	3.162983	-0.915717
O	0.506617	3.952078	-0.328614
C	2.036559	-0.806464	-1.047840
C	3.100683	-0.059572	-0.530027
C	4.358140	-0.646986	-0.407237
C	4.594409	-1.979260	-0.784870
C	3.521943	-2.703922	-1.314602
C	2.255271	-2.125511	-1.449956
C	5.958205	-2.602162	-0.611983
O	2.650631	3.269983	-0.704789
C	3.065062	4.353296	0.135986
C	0.624979	1.292868	1.564327
O	1.283756	2.106303	2.065157
C	0.765551	-1.371461	1.682695
O	1.459862	-2.148054	2.201625
H	0.442072	0.576052	-3.124533
H	0.148292	-0.935418	-1.646821
H	2.956412	0.977353	-0.249565
H	5.177779	-0.056580	-0.009212
H	6.746418	-1.945567	-0.991212
H	6.025448	-3.557875	-1.137910
H	6.175245	-2.788807	0.445637
H	3.674682	-3.729993	-1.633829
H	1.441067	-2.708606	-1.870754
H	4.152085	4.291919	0.177549
H	2.761997	5.314581	-0.286508
H	2.641529	4.252261	1.137295
H	-0.651185	-3.305244	0.579642
H	-2.150853	-5.006728	-0.431154
H	-4.161338	-4.218045	-1.728865
H	-4.559453	-1.784261	-1.946771
H	-4.820111	0.231967	-2.001604
H	-4.970418	2.705572	-2.066244
H	-3.138065	4.053522	-0.986914
H	-1.257391	2.868617	0.095138

TS1′

C	-4.054000	0.832920	-1.531396
C	-2.978448	0.225735	-0.879580
N	-2.008977	0.966713	-0.275850
C	-2.080418	2.310124	-0.335665
C	-3.130176	2.971851	-0.968723
C	-4.137856	2.222909	-1.568398
C	-2.814225	-1.239781	-0.802135
N	-1.730693	-1.679245	-0.106894
C	-1.521796	-3.006947	0.010155
C	-2.365392	-3.953175	-0.557742
C	-3.477987	-3.511413	-1.273490
C	-3.701694	-2.144135	-1.395646
Re	-0.449830	-0.143662	0.838550
C	-1.515900	-0.133160	2.434252
O	-2.161079	-0.128947	3.403029
N	0.700647	-0.223188	-1.167262
C	0.678346	0.965578	-2.133651
C	0.947413	2.225531	-1.894733
C	1.280974	3.162983	-0.915717
O	0.506617	3.952078	-0.328614
C	2.036559	-0.806464	-1.047840
C	3.100683	-0.059572	-0.530027
C	4.358140	-0.646986	-0.407237
C	4.594409	-1.979260	-0.784870
C	3.521943	-2.703922	-1.314602
C	2.255271	-2.125511	-1.449956
C	5.958205	-2.602162	-0.611983
O	2.650631	3.269983	-0.704789
C	3.065062	4.353296	0.135986
C	0.624979	1.292868	1.564327
O	1.283756	2.106303	2.065157
C	0.765551	-1.371461	1.682695
O	1.459862	-2.148054	2.201625
H	0.442072	0.576052	-3.124533
H	0.148292	-0.935418	-1.646821
H	2.956412	0.977353	-0.249565
H	5.177779	-0.056580	-0.009212
H	6.746418	-1.945567	-0.991212
H	6.025448	-3.557875	-1.137910
H	6.175245	-2.788807	0.445637
H	3.674682	-3.729993	-1.633829
H	1.441067	-2.708606	-1.870754
H	4.152085	4.291919	0.177549
H	2.761997	5.314581	-0.286508
H	2.641529	4.252261	1.137295
H	-0.651185	-3.305244	0.579642
H	-2.150853	-5.006728	-0.431154
H	-4.161338	-4.218045	-1.728865
H	-4.559453	-1.784261	-1.946771
H	-4.820111	0.231967	-2.001604
H	-4.970418	2.705572	-2.066244
H	-3.138065	4.053522	-0.986914
H	-1.257391	2.868617	0.095138

P*cco*

Re	-0.220811	-0.531882	0.806138
N	1.950834	-0.876985	0.529395
C	2.333944	-1.806768	-0.388746
C	1.236084	-2.561072	-1.025143
N	-0.022230	-2.190393	-0.661831
C	-1.075163	-2.873788	-1.156870
C	-0.929664	-3.940490	-2.035923
C	0.355776	-4.314496	-2.429229
C	1.446323	-3.619506	-1.917130
C	3.685822	-2.045526	-0.664789
C	4.663939	-1.329241	0.016596
C	4.265575	-0.380397	0.959952
C	2.908047	-0.180284	1.177319
C	-0.180179	0.893787	2.058459
O	-0.125784	1.758549	2.855802
C	-0.538496	-1.722826	2.230986
O	-0.786061	-2.462487	3.113922
C	-2.253521	-0.247127	0.456571
H	2.553373	0.546990	1.895822
H	4.987467	0.199884	1.520394
H	5.713960	-1.510570	-0.182026
H	3.970820	-2.786897	-1.399248
H	2.451291	-3.904767	-2.198427
H	0.507997	-5.138180	-3.116355
H	-1.807808	-4.459466	-2.398465
H	-2.051066	-2.547432	-0.818761
O	-3.275771	-0.783739	0.919724
N	-0.143493	0.861620	-1.056395
C	-1.505149	1.266247	-1.385928
C	-2.520506	0.761744	-0.683651
H	0.179263	0.238011	-1.798811
C	0.789983	1.973248	-0.980673
C	0.412518	3.163744	-0.354752
C	1.319023	4.220755	-0.275293
C	2.615916	4.115751	-0.798604
C	2.975134	2.909656	-1.416380
C	2.075219	1.845424	-1.510858
H	-0.578568	3.259040	0.074008
H	1.010756	5.142815	0.207669
C	3.592915	5.260107	-0.678476
H	3.969390	2.798165	-1.837359
H	2.373726	0.921655	-1.998285
H	-1.607606	1.971415	-2.205761
C	-3.935488	1.092551	-1.031710
O	-4.484672	1.920500	-0.132104
O	-4.535311	0.645792	-1.995799
C	-5.880532	2.239424	-0.326961
H	-6.135913	2.923107	0.478744
H	-6.026600	2.714039	-1.297789
H	-6.479694	1.330752	-0.267511
H	4.352613	5.221507	-1.463681
H	3.081798	6.224386	-0.742193
H	4.115484	5.232557	0.284556

P*ccoh*

C	-1.742982	-1.230471	3.297982
C	-1.379122	-1.204804	1.946396
N	-0.074239	-1.121467	1.574299
C	0.875528	-1.083239	2.529502
C	0.579585	-1.120173	3.886804
C	-0.757759	-1.188481	4.278423
C	-2.354957	-1.283403	0.843148
N	-1.829692	-1.221550	-0.410121
C	-2.668321	-1.317741	-1.463191
C	-4.038365	-1.497870	-1.326133
C	-4.582171	-1.566269	-0.042200
C	-3.729901	-1.451731	1.050246
Re	0.367071	-1.000237	-0.594106
C	0.470434	-0.860853	-2.488872
O	0.486389	-0.786984	-3.662074
N	0.287298	1.217316	-0.250217
C	1.472529	1.726764	-0.003346
C	2.618038	0.890448	-0.059359
C	3.959658	1.390373	0.116871
O	4.980848	0.679178	0.034007
C	-0.841584	2.077550	-0.215141
C	-1.777278	2.032829	-1.260649
C	-2.857419	2.913120	-1.277774
C	-3.058209	3.846254	-0.246683
C	-2.135038	3.862835	0.806051
C	-1.044615	2.988672	0.831505
C	-4.218643	4.810409	-0.284127
C	2.342558	-0.481270	-0.378055
O	3.388972	-1.311086	-0.468713
C	0.761187	-2.848366	-0.746605
O	1.061727	-3.982799	-0.841078
O	4.052881	2.713025	0.377225
C	5.384695	3.249528	0.496669
H	1.576759	2.791014	0.201004
H	4.221340	-0.786522	-0.312282
H	-2.207187	-1.258020	-2.440903
H	1.896785	-1.024058	2.178449
H	-2.784923	-1.287433	3.580684
H	-4.657382	-1.583642	-2.209758
H	-4.128666	-1.506105	2.054386
H	-5.646375	-1.709378	0.106130
H	1.383873	-1.093626	4.611580
H	-1.029533	-1.211178	5.326710
H	-1.631120	1.323534	-2.067927
H	-0.347932	3.010455	1.663135
H	-3.559935	2.875076	-2.105645
H	-2.274486	4.563110	1.623967
H	-5.167919	4.292498	-0.452624
H	-4.101823	5.538591	-1.094267
H	-4.296385	5.366230	0.653466
H	5.250369	4.310443	0.697989
H	5.936906	3.103777	-0.432862
H	5.920120	2.767576	1.315264

TS1*b*

C	3.620862	-0.013385	-1.321256
C	2.495780	-0.385451	-0.579778

N	1.440849	-1.002644	-1.169425
C	1.489896	-1.266182	-2.488408
C	2.581138	-0.929725	-3.279986
C	3.665056	-0.286135	-2.684185
C	2.376391	-0.154854	0.873956
N	1.241281	-0.620365	1.461440
C	1.085073	-0.479696	2.792781
C	2.042358	0.123508	3.599762
C	3.203128	0.615940	3.003623
C	3.367923	0.478797	1.629426
Re	-0.281370	-1.494587	0.119213
C	0.364854	-3.291412	0.402459
O	0.742837	-4.376755	0.596182
N	-0.864613	0.661161	-0.050407
C	0.061304	1.762707	-1.538600
C	0.956546	2.574567	-1.241644
C	1.953985	3.341930	-0.610345
O	3.126050	2.994804	-0.420709
C	-2.202460	1.107522	-0.055063
C	-3.116294	0.676497	-1.037597
C	-4.420910	1.170829	-1.069587
C	-4.876976	2.115081	-0.137619
C	-3.965502	2.544944	0.836886
C	-2.657060	2.062575	0.876131
C	-6.285534	2.657732	-0.192879
O	1.501686	4.579552	-0.241655
C	2.480730	5.451574	0.347622
C	-1.421781	-2.141704	-1.282367
O	-2.068298	-2.543976	-2.165896
C	-1.688780	-1.773319	1.404374
O	-2.521484	-1.929888	2.202177
H	-0.560939	1.334967	-2.300502
H	-0.382492	1.149821	0.701524
H	-2.808255	-0.055549	-1.774815
H	-5.102300	0.810273	-1.835621
H	-6.381239	3.449514	-0.945497
H	-6.580995	3.085573	0.768798
H	-7.005291	1.876620	-0.454847
H	-4.284474	3.270280	1.580206
H	-1.974302	2.420917	1.641737
H	1.963967	6.394021	0.520019
H	2.845608	5.041068	1.291440
H	3.322917	5.597767	-0.330853
H	0.164782	-0.865955	3.210914
H	1.870462	0.205879	4.665651
H	3.967686	1.102604	3.597319
H	4.248803	0.873969	1.145145
H	4.444350	0.497540	-0.844677
H	4.530867	0.001366	-3.268662
H	2.569839	-1.163696	-4.336734
H	0.626435	-1.763971	-2.909830

I1*b*

C	3.981291	0.531241	-0.706244
C	2.760447	0.100983	-0.179755
N	1.902116	-0.645842	-0.919508
C	2.228477	-0.951395	-2.190516
C	3.423573	-0.551572	-2.772433
C	4.319681	0.200479	-2.012441
C	2.327897	0.404011	1.198055
N	1.140876	-0.137287	1.582440
C	0.685371	0.097161	2.831470
C	1.385323	0.865409	3.751556
C	2.606323	1.421238	3.366128
C	3.077140	1.189724	2.079051
Re	0.075753	-1.354485	0.083507
C	0.973871	-2.977102	0.591874
O	1.528102	-3.955424	0.888805
N	-0.868517	0.677280	-0.495045
C	-0.382236	1.196602	-1.828315
C	0.349158	2.283455	-1.988270
C	0.878717	3.175946	-1.020740
O	1.941756	3.043598	-0.384074
C	-2.317562	0.742623	-0.352329
C	-3.163418	0.085019	-1.253734
C	-4.546022	0.180629	-1.104871
C	-5.124541	0.914870	-0.056741
C	-4.262962	1.559510	0.837601
C	-2.874720	1.482956	0.690785
C	-6.623316	0.996869	0.102995
O	0.150962	4.348363	-0.907339
C	0.739525	5.378960	-0.105182
C	-0.718954	-2.249296	-1.425310
O	-1.180952	-2.775220	-2.355126
C	-1.441732	-1.843393	1.171468
O	-2.329765	-2.141908	1.860242
H	-0.691630	0.521118	-2.620947
H	-0.480036	1.320153	0.196623
H	-2.752160	-0.497518	-2.069757
H	-5.187816	-0.326890	-1.818901
H	-7.107286	1.302500	-0.829410
H	-6.894143	1.718175	0.877403
H	-7.047828	0.027658	0.384868
H	-4.679423	2.140076	1.654931
H	-2.225805	2.005753	1.387004
H	0.037883	6.211653	-0.135911
H	0.882629	5.046324	0.925250
H	1.705007	5.686798	-0.514309
H	-0.264693	-0.354555	3.085228
H	0.977227	1.019932	4.741734
H	3.180805	2.028387	4.055203
H	4.011481	1.627136	1.756102
H	4.654139	1.125268	-0.104457
H	5.262661	0.527936	-2.433283
H	3.637836	-0.826410	-3.797249
H	1.509498	-1.540999	-2.742998

I1′*b*

C	1.963228	-1.595816	1.187275
C	3.180567	-1.653338	1.868061
C	4.291121	-0.992296	1.346239
C	4.155372	-0.278554	0.160151
C	2.916195	-0.264280	-0.472566
C	0.724858	-2.221796	1.693987
N	-0.391124	-2.025044	0.941019
C	-1.561011	-2.556343	1.349717
C	-1.678842	-3.304011	2.514384
C	-0.541090	-3.508634	3.294365
C	0.668626	-2.960418	2.880235
Re	-0.138470	-0.916881	-0.951373
C	-1.974566	-1.011667	-1.525331
O	-3.086620	-1.114009	-1.851072
N	-0.428741	0.984467	0.331362
C	0.528980	2.063049	-0.065939
C	1.561545	2.357889	0.705845
C	2.596221	3.268973	0.342043
O	3.622101	3.001465	-0.305538
C	-1.790714	1.473155	0.489272
C	-2.497373	1.976194	-0.607179
C	-3.787973	2.475562	-0.433874
C	-4.409552	2.481755	0.824592
C	-3.691571	1.960874	1.908454
C	-2.393583	1.468285	1.749130
C	-5.795979	3.050424	1.005804
H	0.252711	2.480094	-1.035256
C	0.224412	-2.533874	-1.924527
O	0.440874	-3.511491	-2.516810
C	0.281562	0.117587	-2.526044
O	0.540177	0.725892	-3.482475
O	2.436339	4.526123	0.890967
C	3.500426	5.455960	0.649298
H	3.262017	-2.191785	2.802498
H	1.561578	-3.109902	3.471029
H	2.766322	0.298695	-1.382363
H	4.971971	0.286745	-0.267445
H	5.238941	-1.025724	1.870697
H	-0.592570	-4.084954	4.210047
H	-2.641257	-3.712367	2.793789
H	-2.415900	-2.376093	0.711595
H	-0.097445	0.712649	1.259146
H	-1.851133	1.087468	2.608989
H	-4.145369	1.945916	2.894963
H	-4.326133	2.859109	-1.295407
H	-2.045919	1.966345	-1.593428
H	-6.318471	2.569877	1.836877
H	-5.754432	4.124034	1.221518
H	-6.396030	2.924021	0.101342
H	3.190203	6.387492	1.121370
H	4.435236	5.104719	1.092675
H	3.653793	5.609451	-0.421937

TS2*ccb*

N	-1.457488	-1.285119	-0.152413
C	-1.274462	-1.987339	-1.312173
C	-2.326772	-2.282540	-2.164843
C	-3.634345	-1.882717	-1.800981
C	-3.811348	-1.105342	-0.686416
C	-2.666720	-0.678325	0.057231
C	0.116021	-2.396802	-1.588830
N	1.060573	-1.874121	-0.760125

C	2.361286	-2.164235	-0.974629
C	2.782779	-2.990937	-2.006383
C	1.819398	-3.556057	-2.845354
C	0.479181	-3.256814	-2.632869
Re	0.317044	-0.761659	0.997016
C	2.047074	-0.287263	1.703231
O	3.111979	-0.035659	2.102885
N	0.118157	1.080066	-0.450078
C	-1.087306	1.893032	-0.160260
C	-2.312278	1.449621	-0.416776
C	-3.521411	2.181132	-0.156964
O	-4.385698	1.860332	0.667144
C	1.317138	1.883511	-0.612196
C	1.820391	2.622163	0.462080
C	2.958115	3.410583	0.290428
C	3.623844	3.475632	-0.942757
C	3.110231	2.719622	-2.004710
C	1.963946	1.936566	-1.848662
C	4.838298	4.351903	-1.126144
H	-0.819410	2.845068	0.296821
C	0.342944	-2.352546	2.059517
O	0.355031	-3.329003	2.693949
C	-0.525400	0.198211	2.442450
O	-1.038709	0.761911	3.320552
O	-3.724100	3.231107	-1.015013
C	-4.987544	3.906816	-0.893567
H	-2.161478	-2.840170	-3.076667
H	-0.282965	-3.691930	-3.264744
H	-2.829681	-0.280342	1.049061
H	-4.785605	-0.747685	-0.377383
H	-4.479795	-2.189758	-2.406461
H	2.108321	-4.222341	-3.649628
H	3.838699	-3.191658	-2.137042
H	3.069367	-1.719195	-0.287573
H	-0.061003	0.609994	-1.335973
H	1.572457	1.373179	-2.690423
H	3.608211	2.747176	-2.969425
H	3.344265	3.975073	1.133527
H	1.334917	2.567395	1.431180
H	5.368500	4.492796	-0.181028
H	5.533396	3.921270	-1.851526
H	4.555445	5.344209	-1.494836
H	-4.986520	4.670755	-1.670472
H	-5.814148	3.210124	-1.045423
H	-5.086355	4.367953	0.091479

P*ccb*

C	0.587751	-3.424689	-2.460678
C	0.191003	-2.504850	-1.476389
N	1.096316	-2.029493	-0.576182
C	2.381501	-2.447073	-0.639802
C	2.827015	-3.349414	-1.591152
C	1.906786	-3.848500	-2.521211
C	-1.184821	-1.995221	-1.324163
N	-1.406542	-1.267498	-0.185077
C	-2.625539	-0.453719	-0.105950
C	-3.763145	-1.037712	-0.925981
C	-3.508445	-1.860298	-1.966106
C	-2.163844	-2.279287	-2.255351
C	-2.351289	1.031776	-0.413887
C	-3.522810	1.936747	-0.258259
O	-4.560439	1.600973	0.295091
Re	0.289291	-0.733028	1.010771
C	-0.593555	0.390627	2.301287
O	-1.126953	1.056780	3.094888
C	-1.150260	1.605713	-0.611704
N	0.129875	0.938087	-0.693915
C	1.256607	1.846140	-0.789303
C	1.430848	2.844355	0.172385
C	2.512966	3.718989	0.070153
C	3.443217	3.614079	-0.974856
C	3.263220	2.587119	-1.910999
C	2.177004	1.712809	-1.828956
C	4.593598	4.583652	-1.095379
C	2.001991	-0.153510	1.696523
O	3.054357	0.185846	2.069208
C	0.271398	-2.161646	2.268302
O	0.247643	-3.038128	3.037801
O	-3.345628	3.166747	-0.783847
C	-4.426711	4.100476	-0.578461
H	-1.082112	2.683984	-0.692265
H	-5.336974	3.733125	-1.053622
H	-4.099529	5.027389	-1.043213
H	-4.604550	4.244484	0.488566
H	-2.948662	-0.440226	0.944183
H	-4.771290	-0.753141	-0.651642

H	-4.323912	-2.247799	-2.569494
H	2.216411	-4.562332	-3.275645
H	3.865049	-3.654965	-1.598286
H	3.052492	-2.039817	0.105216
H	2.047602	0.931418	-2.571507
H	3.976795	2.470987	-2.720646
H	2.643000	4.490080	0.822666
H	0.736078	2.928268	1.001685
H	-0.142389	-3.810279	-3.158881
H	-1.949575	-2.865197	-3.137642
H	0.134415	0.313830	-1.499837
H	4.882290	4.979968	-0.118949
H	5.466197	4.106092	-1.548353
H	4.325534	5.436452	-1.729496

TS2*ins*

Re	-0.434759	-0.191213	0.945970
N	-0.012809	-2.119462	-0.088598
C	-1.010098	-2.654759	-0.841644
C	-2.215461	-1.819395	-0.995993
N	-2.205047	-0.636337	-0.328224
C	-3.268456	0.183191	-0.439021
C	-4.387343	-0.138362	-1.197763
C	-4.407112	-1.351583	-1.885479
C	-3.307795	-2.197695	-1.784559
C	-0.877032	-3.919564	-1.426610
C	0.298711	-4.639966	-1.249217
C	1.324216	-4.077193	-0.487206
C	1.127094	-2.822606	0.075159
C	1.213013	-0.049074	1.919741
O	2.215936	-0.016940	2.512838
C	-1.198507	-1.081464	2.415847
O	-1.685721	-1.617879	3.338321
C	-1.007557	1.525222	1.624556
H	1.895256	-2.352384	0.675270
H	2.260946	-4.595283	-0.325193
H	0.412237	-5.620376	-1.696318
H	-1.685839	-4.335203	-2.012202
H	-3.295570	-3.139439	-2.316440
H	-5.259047	-1.633768	-2.492655
H	-5.213602	0.558423	-1.251103
H	-3.200583	1.126219	0.084401
O	-1.403064	2.511357	2.100538
N	1.130898	0.428328	-1.140494
C	0.449255	1.643475	-1.571893
C	-0.592889	2.064621	-0.876306
H	0.903099	-0.343207	-1.764264
C	2.552782	0.505783	-1.009262
C	3.158553	1.641398	-0.453456
C	4.543682	1.689639	-0.298753
C	5.365440	0.617061	-0.678069
C	4.745557	-0.511615	-1.230509
C	3.360478	-0.568708	-1.402387
H	2.541735	2.480466	-0.151795
H	4.998500	2.581976	0.122002
C	6.861819	0.678655	-0.489243
H	5.355834	-1.351230	-1.550732
H	2.905419	-1.442656	-1.860419
H	0.865859	2.103599	-2.472098
C	-1.449587	3.182735	-1.148932
O	-0.981528	4.352198	-0.611368
O	-2.561928	3.131165	-1.690572
C	-1.861433	5.485217	-0.699743
H	-1.330357	6.305146	-0.218899
H	-2.073388	5.730174	-1.742810
H	-2.801115	5.286307	-0.180131
H	7.369585	-0.045079	-1.131582
H	7.249489	1.674228	-0.720942
H	7.143616	0.456031	0.545771

P*ins*

Re	1.116427	-0.492853	-0.942727
N	0.511817	-1.480394	0.951651
C	1.212619	-1.150192	2.067492
C	2.362678	-0.247456	1.866427
N	2.565511	0.185140	0.595583
C	3.628672	0.968108	0.334544
C	4.540484	1.345550	1.314538
C	4.336368	0.912048	2.623834
C	3.236142	0.107089	2.900093
C	0.875550	-1.682749	3.317697
C	-0.181497	-2.579633	3.423190
C	-0.871813	-2.942826	2.266711
C	-0.494474	-2.369420	1.059311

C	-0.233342	-1.211264	-2.092085
O	-1.052088	-1.685639	-2.783173
C	2.299009	-1.974974	-1.415180
O	3.005468	-2.849096	-1.725847
C	1.755949	0.552968	-2.428238
H	-1.001331	-2.630258	0.138880
H	-1.688795	-3.652554	2.291558
H	-0.451643	-2.996841	4.385752
H	1.436770	-1.399331	4.197803
H	3.060889	-0.246565	3.906535
H	5.023574	1.189135	3.414403
H	5.383184	1.970530	1.047841
H	3.734691	1.302433	-0.686740
O	2.150442	1.166148	-3.335376
N	-2.343200	0.263632	-0.164601
C	-1.457931	1.301616	-0.004924
C	-0.115422	1.256515	-0.258085
H	-1.973986	-0.587122	-0.563292
C	-3.734011	0.298934	-0.016504
C	-4.430679	1.402866	0.505599
C	-5.820279	1.357649	0.632370
C	-6.566914	0.234136	0.252652
C	-5.859149	-0.862672	-0.260953
C	-4.473134	-0.835909	-0.398011
H	-3.900940	2.293802	0.819838
H	-6.335733	2.222480	1.040616
C	-8.072013	0.215008	0.371358
H	-6.401130	-1.754519	-0.562579
H	-3.950941	-1.697839	-0.803069
H	-1.924662	2.221036	0.331516
C	0.563783	2.549831	-0.104107
O	-0.084728	3.467596	0.691711
O	1.643316	2.857018	-0.614425
C	0.532702	4.757898	0.794980
H	-0.087120	5.325721	1.487591
H	1.550972	4.669934	1.178611
H	0.562746	5.254890	-0.177567
H	-8.445290	-0.804173	0.500535
H	-8.409297	0.811887	1.223101
H	-8.551915	0.625722	-0.524602

Figure S12. PCM-B3LYP/6-31+G(d,p) (LANL2DZ for Re) optimized geometries in THF solution of the critical structures involved in the reaction between the complex [Re(NH*p*Tol)(CO)$_3$(bipy)] (bipy = 2,2'-bipyridine) and methyl propiolate (HMAD, HC≡CCO$_2$Me). Relevant distances are given in angstroms.

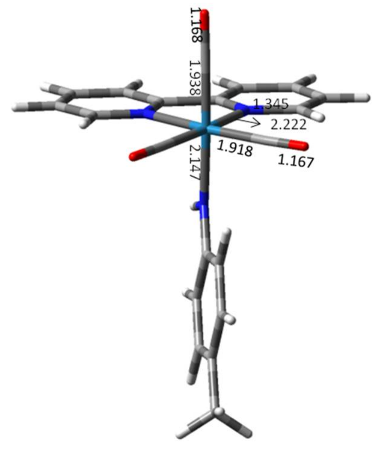

[Re(NH*p*Tol)(CO)$_3$(bipy)]

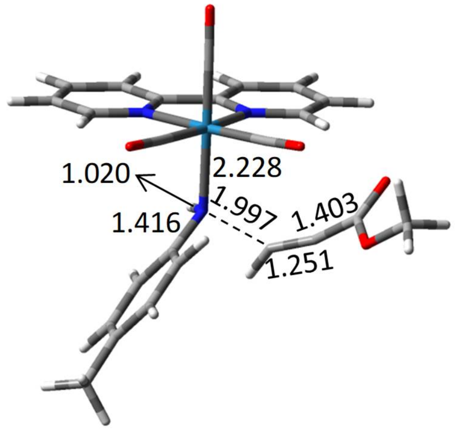

TS1

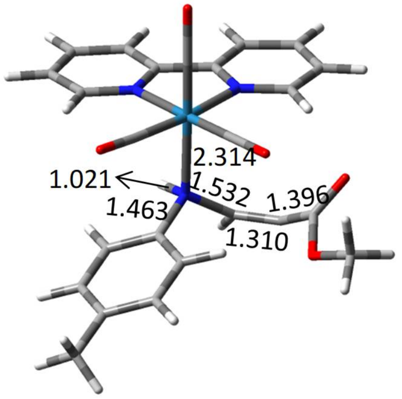

I1

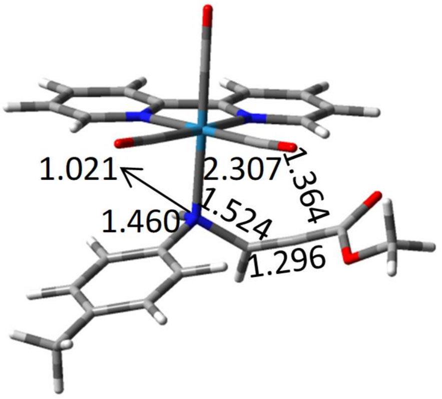

TS1'

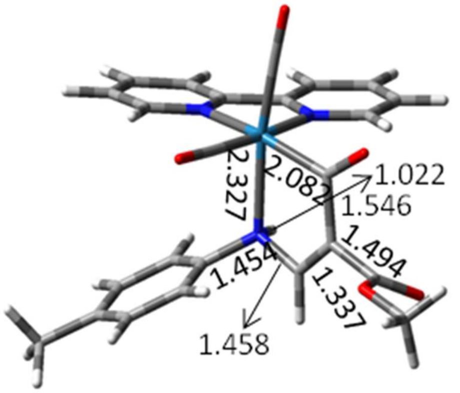

P*cco*

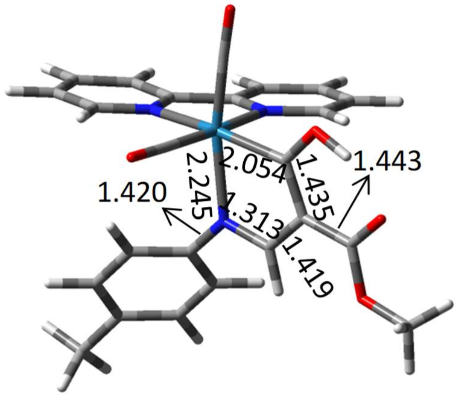

P*ccoh*

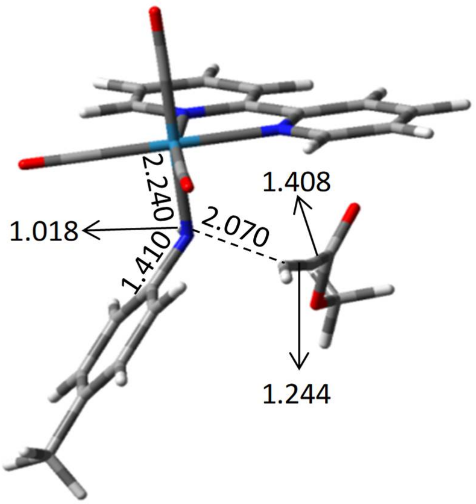

TS1*b*

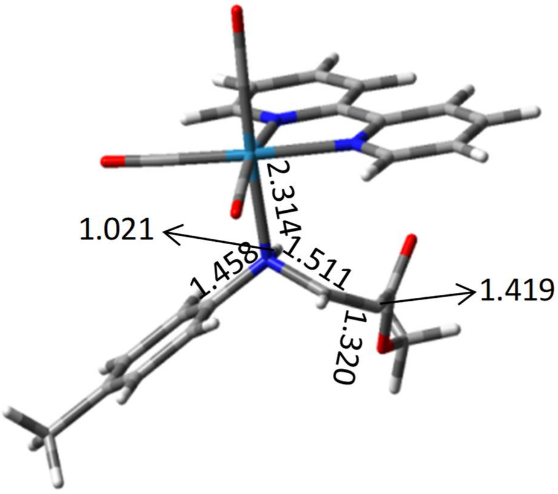

I1*b*

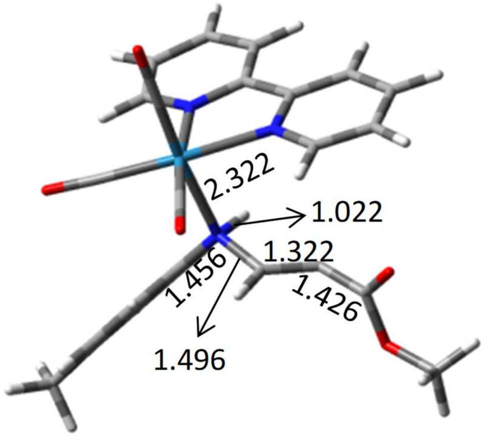

I1'*b*

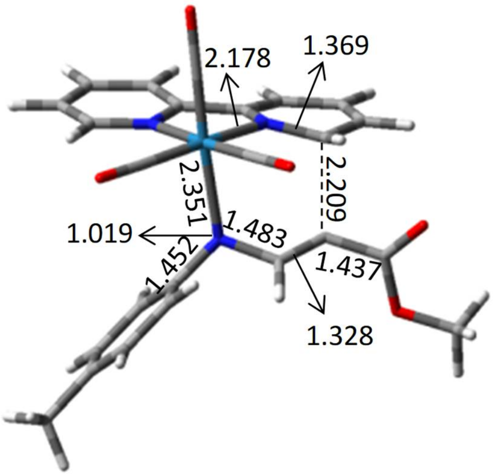

TS2*ccb*

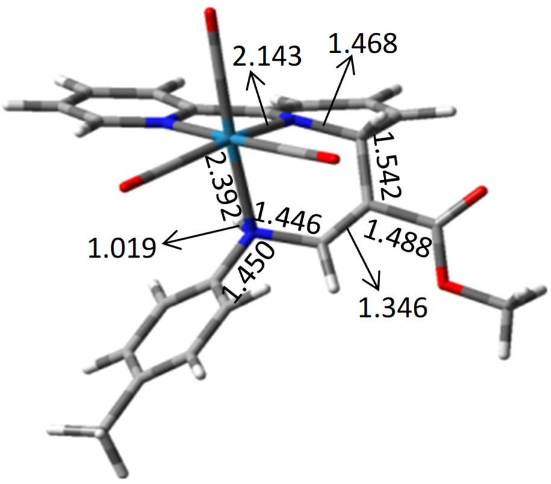

P*ccb*

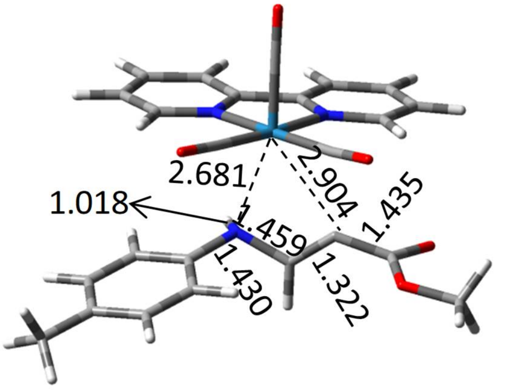

TS2*ins*

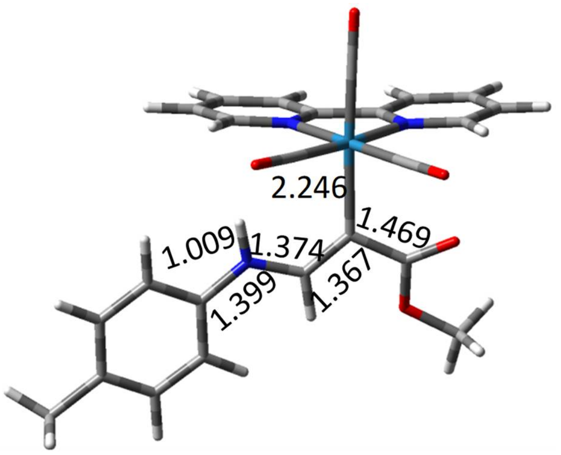

P*ins*

Table S37. PCM-B3LYP/6-31+G(d,p) (LANL2DZ for Re) energies without and with including thermal corrections (E and G, respectively), enthalpies (H), and entropies (S), and CPCM-DLPNO-CCSD(T)/def2-TZVPP//PCM-B3LYP/6-31+G(d,p) (LANL2DZ for Re) energies without and with including thermal corrections (E' and G', respectively) in THF solution of the critical structures involved in the reaction between the complex [Re(OH)(CO)$_3$(bipy)] (bipy = 2,2'-bipyridine) and methyl propiolate (HMAD, HC≡CCO$_2$Me). All the values are given in hartree, except entropies that are in cal/K mol.[a]

Species	E	H	S	G	E'	G'[b]
[Re(OH)(CO)$_3$(bipy)]	-990.486781	-990.267057	141.947	-990.334501	-987.975686	-987.823406
HMAD	-305.215726	-305.136831	77.228	-305.173524	-304.738441	-304.696239
Reactants	-1295.702507	-1295.403888	219.175	-1295.508025	-1292.714128	-1292.519645
**TS1**	-1295.683761	-1295.383431	172.753	-1295.465511	-1292.694888	-1292.476638
**I1**	-1295.687563	-1295.384993	171.763	-1295.466603	-1292.702616	-1292.481656
**TS1'**	-1295.683701	-1295.383415	172.336	-1295.465298	-1292.693387	-1292.474984
**I1'**	-1295.689983	-1295.387778	174.847	-1295.470853	-1292.700735	-1292.481605
**TS2*cco***	-1295.689321	-1295.388222	170.824	-1295.469386	-1292.696835	-1292.476900
**P*cco***	-1295.712691	-1295.409010	171.972	-1295.490719	-1292.717434	-1292.495462
**P*ccoh***	-1295.767429	-1295.462840	165.589	-1295.541517	-1292.764621	-1292.538709
**TS2*ccb***	-1295.677305	-1295.376359	170.141	-1295.457199	-1292.690098	-1292.469992
**P*ccb***	-1295.709972	-1295.406472	167.940	-1295.486265	-1292.730494	-1292.506787
**TS2*ins***	-1295.684273	-1295.383420	171.750	-1295.465023	-1292.693781	-1292.474531
**P*ins***	-1295.745928	-1295.442604	169.724	-1295.523245	-1292.764086	-1292.541403

[a] Thermal magnitudes were computed in THF solution at 298.15 K and 1 atm. [b] For each species, G' was calculated as G' = G – E + E', in which G is the PCM-B3LYP/6-31+G(d,p) (LANL2DZ for Re) energy with including thermal corrections and E and E' are the PCM-B3LYP/6-31+G(d,p) (LANL2DZ for Re) and CPCM-DLPNO-CCSD(T)/def2-TZVPP//PCM-B3LYP/6-31+G(d,p) (LANL2DZ for Re) energies without including thermal corrections, respectively.

Table S38. PCM-B3LYP/6-31+G(d,p) (LANL2DZ for Re) relative energies without and with including thermal corrections (ΔE and ΔG, respectively), enthalpies (ΔH), and entropic contributions (TΔS), and CPCM-DLPNO-CCSD(T)/def2-TZVPP//PCM-B3LYP/6-31+G(d,p) (LANL2DZ for Re) relative energies without and with including thermal corrections (E' and G', respectively) in THF solution of the critical structures involved in the reaction between the complex [Re(OH)(CO)$_3$(bipy)] (bipy = 2,2'-bipyridine) and methyl propiolate (HMAD, HC≡CCO$_2$Me). All the values are given in kcal/mol.[a]

Species	ΔE	ΔH	TΔS	ΔG	ΔE'	ΔG'
[Re(OH)(CO)$_3$(bipy)] + HMAD	0.0	0.0	0.0	0.0	0.0	0.0
**TS1**	11.8	12.8	-13.8	26.7	12.1	27.0
**I1**	9.4	11.9	-14.1	26.0	7.2	23.8
**TS1'**	11.8	12.8	-14.0	26.8	13.0	28.0
**I1'**	7.9	10.1	-13.2	23.3	8.4	23.9
**TS2*cco***	8.3	9.8	-14.4	24.2	10.9	26.8
**P*cco***	-6.4	-3.2	-14.1	10.9	-2.1	15.2
**P*ccoh***	-40.7	-37.0	-16.0	-21.0	-31.7	-12.0
**TS2*ccb***	15.8	17.3	-14.6	31.9	15.1	31.2
**P*ccb***	-4.7	-1.6	-15.3	13.7	-10.3	8.1
**TS2*ins***	11.4	12.8	-14.1	27.0	12.8	28.3
**P*ins***	-27.2	-24.3	-14.7	-9.6	-31.3	-13.7

[a] Thermal magnitudes were evaluated in THF solution at 298.15 K and 1 atm.

Table S39. PCM-B3LYP/6-31+G(d,p) (LANL2DZ for Re) optimized cartesian coordinates, in Å, for the critical structures involved in the reaction of the complex [Re(OH)$(CO)_3$(bipy)] (bipy = 2,2'-bipyridine) towards methyl propiolate (HMAD, HC≡CCO_2Me).

[Re(OH)$(CO)_3$(bipy)]

Re	-0.533906	-0.676015	-0.873981
C	-1.740368	-1.240499	-2.268898
O	-2.475493	-1.576700	-3.114823
C	0.613745	0.156879	-2.164596
O	1.306011	0.690829	-2.937478
C	0.469680	-2.299182	-1.060025
O	1.065747	-3.298188	-1.155389
N	-1.875117	-1.325259	0.765820
C	-1.869776	-2.561474	1.295143
C	-2.615108	-2.900981	2.419151
C	-3.396431	-1.916956	3.022735
C	-3.411842	-0.638190	2.473942
C	-2.643980	-0.364699	1.337192
C	-2.618031	0.961892	0.683919
N	-1.774303	1.090260	-0.370324
C	-1.704902	2.268171	-1.015164
C	-2.472723	3.369610	-0.653206
C	-3.351941	3.244830	0.421611
C	-3.425274	2.028483	1.093902
H	-1.240624	-3.291041	0.801567
H	-2.571903	-3.910424	2.807002
H	-3.983989	-2.137251	3.905518
H	-4.008205	0.138334	2.933701
H	-1.011928	2.319758	-1.845126
H	-2.377654	4.295053	-1.206200
H	-3.972669	4.076224	0.732105
H	-4.107772	1.912315	1.924938
O	0.546226	0.032027	0.801106
H	1.497947	-0.042323	0.671209

TS1

Re	-0.308916	-0.768480	-1.416320
C	-1.041069	-1.828912	-2.835113
O	-1.489295	-2.475915	-3.695916
C	-0.250798	0.766090	-2.573211
O	-0.258539	1.712631	-3.252576
C	1.477907	-1.228676	-1.987398
O	2.547386	-1.496390	-2.355883
N	-0.644357	-2.345750	0.088471
C	0.237639	-3.323567	0.367869
C	-0.020543	-4.315427	1.309707
C	-1.230379	-4.289097	1.998422
C	-2.137191	-3.267705	1.727002
C	-1.821163	-2.300979	0.769139
C	-2.726226	-1.184325	0.427319
N	-2.294485	-0.349119	-0.552188
C	-3.071513	0.685786	-0.923393
C	-4.306906	0.943065	-0.340532
C	-4.756207	0.095252	0.671071
C	-3.959008	-0.978424	1.055061
H	1.185129	-3.293651	-0.153705
H	0.724924	-5.077233	1.496883
H	-1.467095	-5.045720	2.737463
H	-3.077376	-3.222764	2.259638
H	-2.681228	1.316461	-1.711112
H	-4.893159	1.789124	-0.675542
H	-5.712420	0.262117	1.152278
H	-4.293681	-1.646476	1.836656
O	0.358986	0.384363	0.319239
H	0.995416	1.051967	0.030974
C	1.249738	-0.280819	1.755079
C	2.344419	-0.897510	1.650458
C	3.342258	-1.491849	0.845260
O	3.355751	-2.669600	0.468146
O	4.366902	-0.635402	0.550911
C	5.433797	-1.189347	-0.239537
H	0.538417	0.070341	2.478514
H	5.056005	-1.515877	-1.210086
H	5.897019	-2.034018	0.274310
H	6.152995	-0.381165	-0.360800

I1

Re	-0.155493	-0.857428	0.395703
C	-0.951547	-1.249546	2.084777
O	-1.442023	-1.479011	3.115671
C	-0.271303	-2.716894	-0.100514
O	-0.378983	-3.831751	-0.413980
C	1.598013	-1.177594	1.145096
O	2.651171	-1.368360	1.593939
N	-0.256460	1.312903	0.727651
C	0.721392	2.033564	1.307641
C	0.600525	3.400669	1.541126
C	-0.566157	4.049017	1.148106
C	-1.568234	3.312044	0.520563
C	-1.390506	1.943098	0.316196
C	-2.389028	1.094167	-0.365500
N	-2.057594	-0.216929	-0.503006
C	-2.901000	-1.043642	-1.150679
C	-4.112980	-0.615103	-1.678604
C	-4.472127	0.723106	-1.523111
C	-3.600317	1.584072	-0.863210
H	1.632049	1.507550	1.558814
H	1.416746	3.933583	2.011232
H	-0.694951	5.112359	1.313645
H	-2.472577	3.802907	0.188385
H	-2.586148	-2.074745	-1.239463
H	-4.752838	-1.321129	-2.191760
H	-5.412569	1.094286	-1.912885
H	-3.860252	2.626178	-0.738605
O	0.674476	-0.342506	-1.631022
H	1.465065	-0.881309	-1.783411
C	1.050757	1.049040	-2.084850
C	2.203047	1.615277	-1.825731
C	3.255442	1.068032	-1.031285
O	3.560685	1.400838	0.122263
O	4.007827	0.123565	-1.702142
C	5.156180	-0.375469	-0.994922
H	0.198831	1.416560	-2.641656
H	4.850514	-0.869290	-0.069998
H	5.630113	-1.086911	-1.670073
H	5.846451	0.436504	-0.756675

TS1'

Re	-0.223288	-0.817737	0.244119
C	-0.769403	-1.155149	2.037571
O	-1.111468	-1.350621	3.134802
C	-0.406657	-2.687156	-0.177766
O	-0.559737	-3.808189	-0.451337
C	1.614423	-1.139693	0.773857
O	2.685346	-1.360334	1.162315
N	-0.267661	1.372164	0.482730
C	0.779510	2.109548	0.898073
C	0.699229	3.489876	1.060881
C	-0.500551	4.133813	0.772670
C	-1.578211	3.378029	0.316820
C	-1.439102	1.995148	0.178273
C	-2.533201	1.122773	-0.294963
N	-2.243229	-0.203802	-0.379619
C	-3.200172	-1.061146	-0.790208
C	-4.479563	-0.646024	-1.137126
C	-4.784290	0.712792	-1.060375
C	-3.802453	1.602250	-0.635459
H	1.714379	1.592864	1.072983
H	1.572248	4.033978	1.396362
H	-0.598107	5.206708	0.890120
H	-2.513858	3.861515	0.073023
H	-2.917611	-2.104658	-0.832873
H	-5.211747	-1.375261	-1.458870
H	-5.770090	1.076950	-1.323256
H	-4.025843	2.658033	-0.563703
O	0.259316	-0.301190	-1.870340
H	0.079393	-1.055431	-2.455781
C	1.427561	0.482474	-2.415961
C	2.524533	0.648786	-1.773620
C	3.575918	0.774033	-0.892714
O	3.719485	1.650785	-0.008821
O	4.558384	-0.189425	-1.076435

C	5.650954	-0.138276	-0.153411
H	1.114856	0.818530	-3.401635
H	5.300211	-0.276125	0.871853
H	6.311344	-0.958075	-0.436456
H	6.185921	0.812072	-0.223190

I1′

Re	-0.336602	-0.496719	-1.452815
C	-1.221111	-1.133212	-3.007169
O	-1.779058	-1.528187	-3.953461
C	-0.024508	1.184413	-2.327660
O	0.128139	2.219353	-2.842332
C	1.348505	-1.172585	-2.163952
O	2.263195	-1.597211	-2.739867
N	-0.892493	-2.255846	-0.243616
C	-0.147409	-3.373164	-0.153312
C	-0.548592	-4.480446	0.586474
C	-1.758074	-4.421243	1.276418
C	-2.520880	-3.259319	1.204253
C	-2.066914	-2.183672	0.434839
C	-2.812382	-0.914187	0.304478
N	-2.232275	0.041662	-0.469561
C	-2.857294	1.225470	-0.626348
C	-4.078168	1.515754	-0.029091
C	-4.683479	0.539258	0.761064
C	-4.043387	-0.684948	0.927383
H	0.799930	-3.354708	-0.674685
H	0.083851	-5.357979	0.621588
H	-2.102484	-5.262360	1.866509
H	-3.456155	-3.191235	1.743034
H	-2.356547	1.949642	-1.255475
H	-4.537414	2.482680	-0.189734
H	-5.638273	0.723673	1.238875
H	-4.500770	-1.455635	1.532550
O	0.489128	0.212913	0.519315
H	0.360523	1.147508	0.735393
C	1.782363	-0.233667	1.033708
C	2.402714	-1.214465	0.434514
C	3.616767	-1.844013	0.821993
O	3.740795	-2.856011	1.529926
O	4.724899	-1.281116	0.222805
C	5.968969	-1.960926	0.439323
H	2.010014	0.339340	1.932198
H	5.925944	-2.982942	0.055579
H	6.717154	-1.386479	-0.106167
H	6.219820	-1.992526	1.501721

TS2*cco*

Re	-0.169599	-0.832914	0.281301
C	-0.754583	-1.136404	2.053015
O	-1.109958	-1.324695	3.152738
C	0.079663	-2.719678	0.068702
O	0.190929	-3.873516	-0.077956
C	1.692635	-0.653175	0.920689
O	2.643713	-0.699311	1.599196
N	-0.677608	1.317053	0.283318
C	0.153872	2.285361	0.712117
C	-0.200183	3.629822	0.710862
C	-1.459784	3.989827	0.235119
C	-2.315288	2.994785	-0.228664
C	-1.902094	1.658848	-0.196722
C	-2.744520	0.542190	-0.673667
N	-2.199023	-0.699628	-0.571147
C	-2.918289	-1.764060	-0.981145
C	-4.195199	-1.648089	-1.516305
C	-4.759943	-0.377906	-1.631332
C	-4.028110	0.723977	-1.200420
H	1.128087	1.960475	1.050898
H	0.503507	4.368564	1.071681
H	-1.771793	5.027611	0.218625
H	-3.293925	3.257780	-0.606095
H	-2.444269	-2.730229	-0.869264
H	-4.725967	-2.536750	-1.832754
H	-5.752333	-0.246428	-2.045655
H	-4.450245	1.715975	-1.280369
O	0.387668	-0.357336	-1.858606
H	0.139830	-0.994468	-2.543619
C	1.715703	0.163299	-2.113235
C	2.489650	0.405380	-1.087240
C	3.781140	1.014028	-1.057063
O	4.019690	2.216194	-0.883064
O	4.802688	0.100162	-1.115982
C	6.127889	0.625620	-0.938788
H	1.850259	0.327948	-3.180190
H	6.206690	1.164898	0.007172
H	6.788972	-0.239753	-0.939276
H	6.389484	1.300855	-1.756716

P*cco*

Re	0.183847	-0.886140	-1.370689
C	0.180482	-2.180180	-2.719088
O	0.205246	-3.008298	-3.558612
C	0.722717	0.396766	-2.659027
O	1.021325	1.195095	-3.472092
C	2.173021	-1.241756	-0.816853
O	3.018164	-2.040151	-1.244625
N	-0.659783	-2.281330	0.133032
C	0.054671	-3.265003	0.714375
C	-0.502801	-4.161212	1.619523
C	-1.855070	-4.036120	1.938874
C	-2.598870	-3.024918	1.338177
C	-1.980016	-2.156020	0.432735
C	-2.700378	-1.082785	-0.280674
N	-1.981413	-0.420977	-1.228915
C	-2.593346	0.548199	-1.942337
C	-3.921129	0.907613	-1.744737
C	-4.656613	0.246431	-0.759446
C	-4.038339	-0.759728	-0.024757
H	1.097872	-3.321574	0.429829
H	0.114229	-4.935647	2.056829
H	-2.325395	-4.716571	2.638679
H	-3.652091	-2.921583	1.561176
H	-1.984364	1.036507	-2.692160
H	-4.360871	1.689755	-2.350717
H	-5.691894	0.504453	-0.570458
H	-4.590558	-1.287026	0.741730
O	0.454709	0.521195	0.403384
H	-0.039625	1.324453	0.616861
C	1.747938	0.538005	0.880083
C	2.620095	-0.338048	0.360177
C	3.976642	-0.414141	0.960657
O	4.610228	-1.438417	1.141118
O	4.449560	0.808103	1.311189
C	5.744079	0.821316	1.947637
H	1.941086	1.246321	1.675678
H	5.947137	1.866815	2.168665
H	6.498469	0.417299	1.271609
H	5.723186	0.229524	2.864495

P*ccoh*

Re	-0.195264	-0.660467	0.453265
C	-0.616580	-0.742846	2.288076
O	-0.827777	-0.769031	3.447400
C	0.077845	-2.547306	0.487409
O	0.189620	-3.715121	0.514598
C	1.796607	-0.317819	0.735550
O	2.471633	-0.303825	1.884945
N	-0.810600	1.452707	0.293410
C	-0.071580	2.478773	0.757763
C	-0.470164	3.804819	0.634388
C	-1.677303	4.083139	-0.007112
C	-2.446658	3.026876	-0.484573
C	-1.997140	1.712572	-0.315824
C	-2.762366	0.531108	-0.757283
N	-2.210538	-0.674298	-0.451465
C	-2.863851	-1.795086	-0.821267
C	-4.081542	-1.772713	-1.490147
C	-4.657114	-0.539195	-1.797440
C	-3.987469	0.622102	-1.427454
H	0.860968	2.209811	1.237150
H	0.157151	4.593866	1.028797
H	-2.017420	5.103934	-0.130253
H	-3.386893	3.222449	-0.981886
H	-2.385670	-2.731230	-0.561904
H	-4.563663	-2.704865	-1.755815
H	-5.607855	-0.481969	-2.314082
H	-4.415098	1.589361	-1.655535
O	0.532332	-0.327875	-1.610765
H	3.429725	-0.109384	1.692737
C	1.776128	-0.057111	-1.645812
C	2.546627	-0.031323	-0.470100
C	3.971538	0.231586	-0.457901
O	4.655061	0.247601	0.581185
O	4.522053	0.461175	-1.664555
C	5.938150	0.738447	-1.683482
H	2.227362	0.148429	-2.619441
H	6.496990	-0.121182	-1.311767
H	6.178021	0.930057	-2.726462

H	6.161033	1.611743	-1.069642

TS2*ccb*

Re	-0.796397	-0.906877	0.124734
C	-1.793892	-1.288834	1.697851
O	-2.407731	-1.511540	2.663111
C	-1.770821	-2.275933	-0.824532
O	-2.376305	-3.072467	-1.421479
C	0.528516	-2.215142	0.641707
O	1.319726	-2.996604	0.977232
N	0.240391	0.861506	0.845804
C	1.565473	0.791688	1.195849
C	2.244202	1.990151	1.591738
C	1.677436	3.205414	1.304102
C	0.410338	3.255782	0.678280
C	-0.281644	2.069136	0.486546
C	-1.635508	2.018427	-0.100375
N	-2.104264	0.778762	-0.405295
C	-3.335975	0.648756	-0.939915
C	-4.160525	1.736019	-1.192458
C	-3.691127	3.014012	-0.881150
C	-2.422343	3.153303	-0.331415
H	1.875188	-0.144655	1.635090
H	3.218756	1.912609	2.055955
H	2.189392	4.127623	1.555639
H	-0.024652	4.204082	0.394483
H	-3.657793	-0.361242	-1.158147
H	-5.142583	1.578606	-1.619132
H	-4.308147	3.887177	-1.056750
H	-2.044681	4.132417	-0.070281
O	0.381567	-0.243191	-1.688051
H	0.065967	-0.585439	-2.533623
C	1.809574	-0.069342	-1.759873
C	2.532012	0.196181	-0.692705
C	3.951139	0.413146	-0.690686
O	4.513026	1.511467	-0.638971
O	4.670152	-0.752361	-0.607415
C	6.094237	-0.606021	-0.479554
H	2.133073	-0.164368	-2.794837
H	6.505587	-0.068486	-1.336011
H	6.488598	-1.620459	-0.442294
H	6.348160	-0.066856	0.435448

P*ccb*

Re	-0.680470	-0.488910	-1.528901
C	-1.581413	-1.415543	-2.909409
O	-2.139693	-2.006544	-3.746728
C	-1.436420	1.177410	-2.160625
O	-1.891043	2.201560	-2.488209
C	0.828159	-0.240666	-2.706175
O	1.728435	-0.100906	-3.431232
N	0.135157	-2.057800	-0.351127
C	1.594718	-2.230189	-0.272206
C	1.944832	-3.547409	0.401340
C	1.132224	-4.041602	1.361373
C	-0.125816	-3.406659	1.641391
C	-0.595390	-2.482847	0.729653
C	-1.953770	-1.922531	0.827219
N	-2.241152	-0.924621	-0.053867
C	-3.460945	-0.342223	-0.029877
C	-4.452396	-0.721596	0.859499
C	-4.178689	-1.759331	1.758765
C	-2.928637	-2.360366	1.738265
H	1.979678	-2.259022	-1.297329
H	2.858869	-4.046028	0.106430
H	1.395949	-4.952444	1.890810
H	-0.727550	-3.726622	2.479803
H	-3.629249	0.443883	-0.754494
H	-5.413212	-0.223694	0.840830
H	-4.933645	-2.094640	2.460933
H	-2.704559	-3.174155	2.414397
O	0.409769	0.523659	0.284962
H	0.261382	1.468085	0.431886
C	1.700510	0.184153	0.631606
C	2.267232	-1.013860	0.404559
C	3.707137	-1.048915	0.787999
O	4.251327	-0.280042	1.570231
O	4.377473	-2.023194	0.146317
C	5.779748	-2.147622	0.464130
H	2.257291	0.981208	1.109722
H	5.911232	-2.269695	1.539941
H	6.322815	-1.263337	0.127577
H	6.117820	-3.031257	-0.072029

TS2*ins*

Re	-0.200785	-0.823951	0.375518
C	-0.920912	-1.107135	2.086296
O	-1.343406	-1.276760	3.166567
C	-0.388506	-2.696405	-0.000543
O	-0.552701	-3.825416	-0.244882
C	1.601112	-1.114312	1.029129
O	2.639342	-1.304883	1.519424
N	-0.269289	1.377944	0.587644
C	0.722790	2.107533	1.134100
C	0.638069	3.484706	1.296426
C	-0.510678	4.141968	0.857302
C	-1.530356	3.397264	0.274033
C	-1.389411	2.010897	0.149541
C	-2.424244	1.147431	-0.451381
N	-2.139316	-0.179649	-0.484637
C	-3.039494	-1.026876	-1.021448
C	-4.250877	-0.601457	-1.551138
C	-4.550972	0.761386	-1.523460
C	-3.630017	1.640314	-0.964762
H	1.608691	1.563894	1.430616
H	1.462976	4.021389	1.746566
H	-0.611191	5.215665	0.963399
H	-2.426145	3.889096	-0.079992
H	-2.768970	-2.074606	-1.019885
H	-4.936032	-1.325641	-1.973271
H	-5.485566	1.133070	-1.926196
H	-3.844988	2.699619	-0.933671
O	0.090852	-0.532677	-2.148466
H	-0.102882	-1.214722	-2.809888
C	1.423918	-0.056457	-2.290453
C	2.112686	0.104732	-1.185716
C	3.406416	0.676386	-0.977708
O	3.639605	1.826736	-0.584823
O	4.424016	-0.229334	-1.134427
C	5.739873	0.235732	-0.792626
H	1.674463	0.176619	-3.324755
H	6.400160	-0.616232	-0.945725
H	6.037549	1.065991	-1.437065
H	5.775542	0.561662	0.248409

P*ins*

Re	-1.066646	0.285236	0.672449
C	-1.072842	1.460905	2.227711
O	-1.105464	2.158298	3.163559
C	-2.711148	1.051302	0.046825
O	-3.706150	1.528118	-0.334289
C	-2.079589	-1.030011	1.642002
O	-2.689685	-1.808435	2.260262
N	0.968830	-0.459723	1.132583
C	1.218305	-1.520434	1.923342
C	2.507358	-1.950029	2.218543
C	3.586383	-1.263429	1.663764
C	3.335169	-0.176448	0.832349
C	2.013928	0.210970	0.580887
C	1.662720	1.367281	-0.267834
N	0.336010	1.624853	-0.401169
C	-0.053152	2.678677	-1.142946
C	0.849270	3.523617	-1.777832
C	2.213640	3.264356	-1.651420
C	2.622225	2.173935	-0.890849
H	0.351481	-2.037502	2.308924
H	2.649588	-2.804172	2.867983
H	4.605571	-1.567923	1.870350
H	4.157735	0.367770	0.388645
H	-1.119820	2.832937	-1.226102
H	0.482813	4.361820	-2.356508
H	2.947388	3.899515	-2.133409
H	3.675482	1.957019	-0.777343
O	-1.678976	0.589905	-2.687604
H	-1.910088	0.634914	-3.623778
C	-1.260848	-0.677900	-2.380848
C	-0.943873	-1.044044	-1.120796
C	-0.543929	-2.453971	-0.973788
O	-0.555560	-3.091557	0.077993
O	-0.117726	-3.059003	-2.129893
C	0.202714	-4.454928	-2.031065
H	-1.224788	-1.354161	-3.231809
H	1.033986	-4.611112	-1.340778
H	-0.659879	-5.025177	-1.681923
H	0.480819	-4.763440	-3.037870

Figure S13. PCM-B3LYP/6-31+G(d,p) (LANL2DZ for Re) optimized geometries in THF solution of the critical structures involved in the reaction between the complex [Re(OH)$(CO)_3$(bipy)] (bipy = 2,2'-bipyridine) and methyl propiolate (HMAD, HC≡CCO_2Me). Relevant distances are given in angstroms.

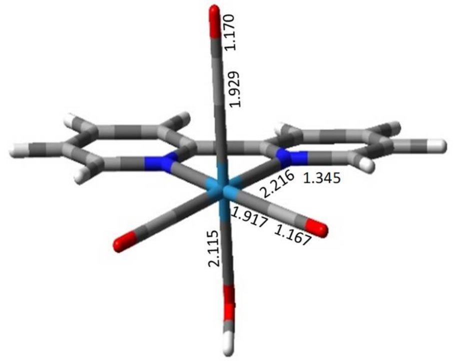

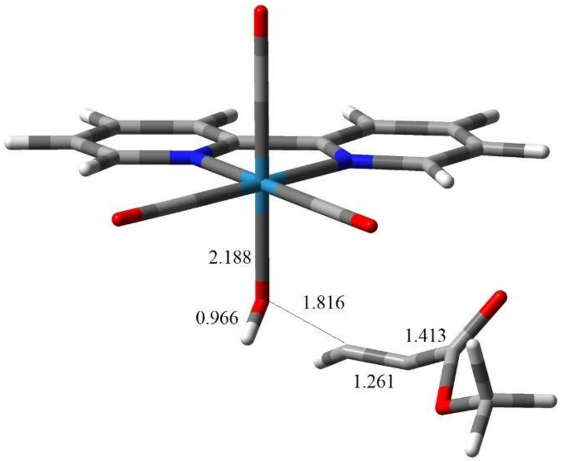

[Re(OH)$(CO)_3$(bipy)]

TS1

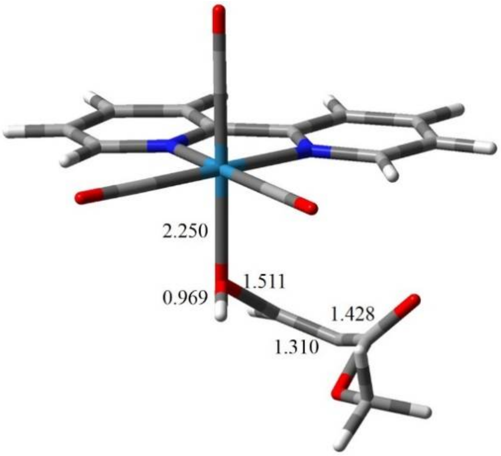

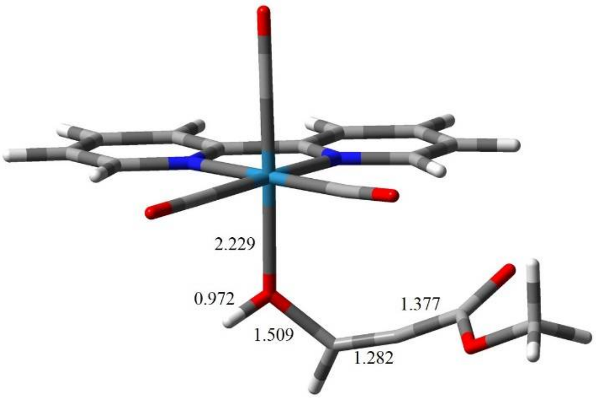

I1

TS1'

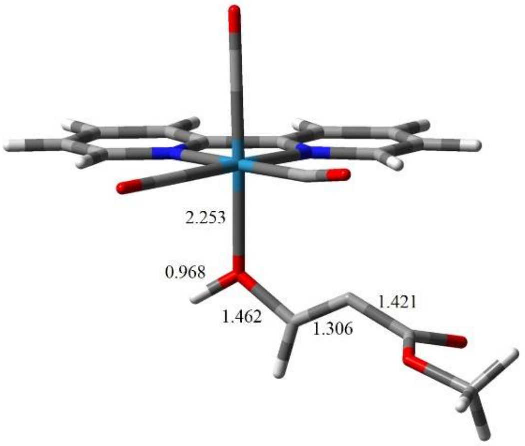

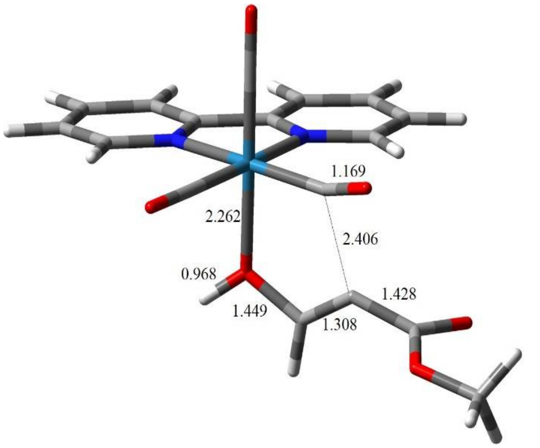

I1'

TS2*cco*

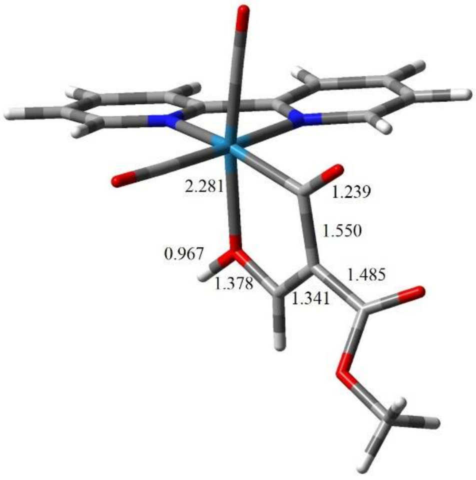

P*cco*

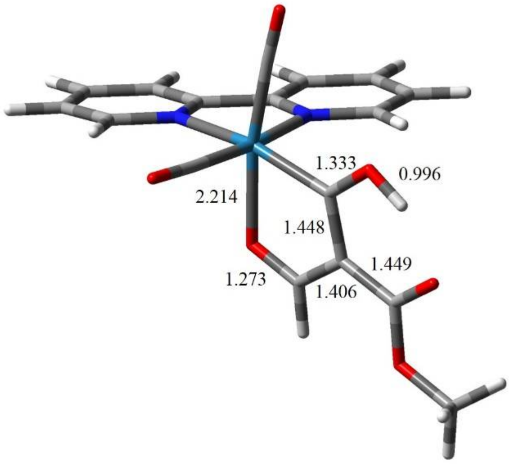

P*ccoh*

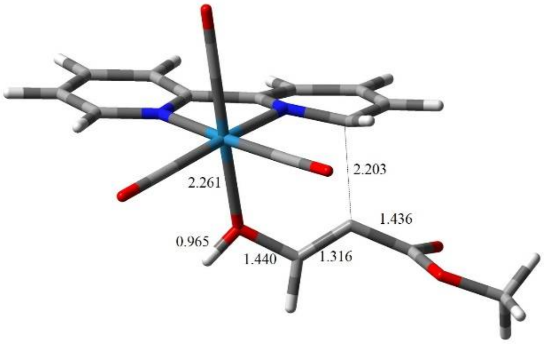

TS2*ccb*

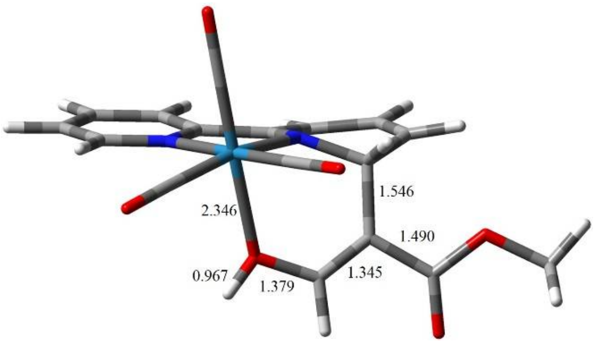

P*ccb*

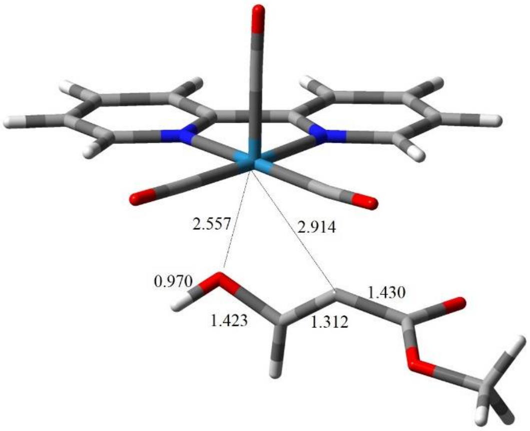

TS2*ins*

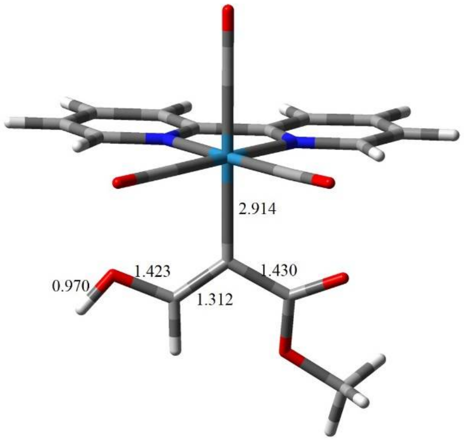

P*ins*

Table S40. PCM-B3LYP/6-31+G(d,p) (LANL2DZ for Re) energies without and with including thermal corrections (E and G, respectively), enthalpies (H), and entropies (S), and CPCM-DLPNO-CCSD(T)/def2-TZVPP//PCM-B3LYP/6-31+G(d,p) (LANL2DZ for Re) energies without and with including thermal corrections (E' and G', respectively) in THF solution of the critical structures involved in the reaction between the complex [Re(OMe)$(CO)_3$(bipy)] (bipy = 2,2'-bipyridine) and methyl propiolate (HMAD, HC≡CCO_2Me). All the values are given in hartree, except entropies that are in cal/K mol.[a]

Species	E	H	S	G	E'	G'[b]
[Re(OMe)$(CO)_3$(bipy)]	-1029.781957	-1029.532513	148.293	-1029.602972	-1027.194607	-1027.015622
HMAD	-305.215726	-305.136831	77.228	-305.173524	-304.738441	-304.696239
Reactants	-1334.997683	-1334.669344	225.521	-1334.776496	-1331.933048	-1331.711861
**TS1**	-1334.978019	-1334.647773	176.286	-1334.731532	-1331.917071	-1331.670584
**I1**	-1334.983003	-1334.651302	183.045	-1334.738272	-1331.924440	-1331.679709
**TS1'**	-1334.982629	-1334.651885	175.771	-1334.735399	-1331.919587	-1331.672357
**P*cco***	-1335.013437	-1334.680028	181.449	-1334.766240	-1331.943278	-1331.696081
**TS2*ccb***	-1334.975442	-1334.644176	172.717	-1334.726240	-1331.914127	-1331.664925
**P*ccb***	-1335.009546	-1334.676037	173.088	-1334.758277	-1331.955284	-1331.704015
**TS2*ins***	-1334.984538	-1334.654107	179.758	-1334.739516	-1331.918621	-1331.673599
**P*ins***	-1335.044406	-1334.712736	176.410	-1334.796554	-1331.985836	-1331.737984

[a] Thermal magnitudes were computed in THF solution at 298.15 K and 1 atm. [b] For each species, G' was calculated as G' = G – E + E', in which G is the PCM-B3LYP/6-31+G(d,p) (LANL2DZ for Re) energy with including thermal corrections and E and E' are the PCM-B3LYP/6-31+G(d,p) (LANL2DZ for Re) and CPCM-DLPNO-CCSD(T)/def2-TZVPP//PCM-B3LYP/6-31+G(d,p) (LANL2DZ for Re) energies without including thermal corrections, respectively.

Table S41. PCM-B3LYP/6-31+G(d,p) (LANL2DZ for Re) relative energies without and with including thermal corrections (ΔE and ΔG, respectively), enthalpies (ΔH), and entropic contributions (TΔS), and CPCM-DLPNO-CCSD(T)/def2-TZVPP//PCM-B3LYP/6-31+G(d,p) (LANL2DZ for Re) relative energies without and with including thermal corrections (E' and G', respectively) in THF solution of the critical structures involved in the reaction between the complex $[Re(OMe)(CO)_3(bipy)]$ (bipy = 2,2'-bipyridine) and methyl propiolate (HMAD, $HC{\equiv}CCO_2Me$). All the values are given in kcal/mol.[a]

Species	ΔE	ΔH	TΔS	ΔG	ΔE'	ΔG'
$[Re(OMe)(CO)_3(bipy)]$ + HMAD	0.0	0.0	0.0	0.0	0.0	0.0
**TS1**	12.3	13.5	-14.7	28.2	10.0	25.9
**I1**	9.2	11.3	-12.7	24.0	5.4	20.2
**TS1'**	9.4	11.0	-14.8	25.8	8.4	24.8
**P*cco***	-9.9	-6.7	-13.1	6.4	-6.4	9.9
**TS2*ccb***	14.0	15.8	-15.7	31.5	11.9	29.5
**P*ccb***	-7.4	-4.2	-15.6	11.4	-14.0	4.9
**TS2*ins***	8.2	9.6	-13.6	23.2	9.1	24.0
**P*ins***	-29.3	-27.2	-14.6	-12.6	-33.1	-16.4

[a] Thermal magnitudes were evaluated in THF solution at 298.15 K and 1 atm.

Table S42. PCM-B3LYP/6-31+G(d,p) (LANL2DZ for Re) optimized cartesian coordinates, in Å, for the critical structures involved in the reaction of the complex [Re(OMe)(CO)$_3$(bipy)] (bipy = 2,2'-bipyridine) towards methyl propiolate (HMAD, HC≡CCO$_2$Me).

[Re(OMe)(CO)$_3$(bipy)]

Re	0.853439	0.011207	0.081903
C	1.042769	0.004725	2.005785
O	1.164547	-0.000337	3.169166
C	2.199153	1.373638	-0.052975
O	2.996659	2.223456	-0.119841
C	2.212654	-1.336861	-0.055497
O	3.017935	-2.179805	-0.119269
C	-2.131014	0.728149	0.000018
C	-0.862031	2.676811	0.145359
C	-3.305184	1.479156	-0.113966
C	-1.991419	3.481429	0.038347
H	0.125327	3.108859	0.245935
C	-3.236213	2.868806	-0.097050
H	-4.262677	0.986678	-0.218455
H	-1.887501	4.558442	0.059596
H	-4.139253	3.460124	-0.187036
C	-2.121026	-0.750348	0.008963
C	-0.823087	-2.679833	0.148821
C	-3.286607	-1.518634	-0.075425
C	-1.941955	-3.501245	0.060523
H	0.171302	-3.097565	0.239640
C	-3.197863	-2.907205	-0.053933
H	-4.253162	-1.039634	-0.155367
H	-1.821269	-4.576617	0.082447
H	-4.094187	-3.511209	-0.123476
N	-0.903402	-1.337837	0.118754
N	-0.923001	1.333683	0.120981
O	0.372230	0.029844	-1.958021
C	1.369225	-0.078205	-2.935003
H	2.122939	0.727335	-2.868970
H	1.921108	-1.033387	-2.876855
H	0.918916	-0.022047	-3.938422

TS1

C	-3.689061	1.617801	-0.961506
C	-2.489553	1.133850	-0.428304
N	-2.216087	-0.196089	-0.421446
C	-3.116588	-1.054359	-0.935900
C	-4.324649	-0.637310	-1.482284
C	-4.614813	0.726495	-1.494390
C	-1.453838	2.008873	0.159221
N	-0.324683	1.388628	0.593495
C	0.662979	2.126230	1.133836
C	0.563654	3.505365	1.290630
C	-0.592825	4.149154	0.858797
C	-1.608981	3.392156	0.281149
Re	-0.259313	-0.808850	0.392784
C	1.537142	-1.153592	1.015287
O	2.613035	-1.383418	1.390219
C	1.311805	1.158060	-2.053751
C	2.469232	1.375856	-1.611218
C	3.526522	0.996107	-0.749900
O	3.683279	1.394974	0.409365
C	-0.478878	-2.693617	0.093417
O	-0.661413	-3.832022	-0.079345
C	-0.934793	-1.036461	2.175253
O	-1.340860	-1.172238	3.260105
O	4.425904	0.157740	-1.345429
C	5.532141	-0.249879	-0.520218
H	0.538621	1.544112	-2.689794
H	6.117066	0.615851	-0.203120
H	6.135027	-0.904352	-1.147235
H	5.168937	-0.785749	0.358970
H	1.565417	1.601009	1.417719
H	1.387074	4.051833	1.731620
H	-0.704986	5.221859	0.964690
H	-5.545380	1.092008	-1.911833
H	-5.013614	-1.369359	-1.883727
H	-2.851366	-2.102552	-0.905379
H	-3.898459	2.678536	-0.965238
H	-2.512300	3.875033	-0.065757
O	0.383954	-0.393219	-1.651453
C	1.075055	-1.417451	-2.362381
H	2.075017	-1.587881	-1.943464
H	0.507758	-2.352594	-2.323891
H	1.186577	-1.120016	-3.410831

I1

Re	-0.523352	-0.908058	0.356119
C	-1.715208	-1.145201	1.828595
O	-2.437149	-1.295390	2.729562
N	-1.990639	0.272262	-0.786418
C	-2.019963	1.610303	-0.542427
C	-2.928834	2.439515	-1.205738
C	-3.820520	1.889770	-2.122240
C	-3.786232	0.516709	-2.360696
C	-2.856375	-0.254898	-1.673784
C	-1.025451	2.110785	0.429391
N	-0.143693	1.187693	0.900295
C	0.851024	1.583789	1.714269
C	0.977085	2.899694	2.149585
C	0.042934	3.839367	1.723622
C	-0.959968	3.443028	0.840628
C	0.887497	-1.701746	1.409492
O	1.726299	-2.152922	2.074295
C	-1.026161	-2.643946	-0.316263
O	-1.372356	-3.672898	-0.733739
C	1.280040	0.815141	-1.747990
C	2.452436	1.333206	-1.488927
C	3.580534	0.851083	-0.778790
O	4.580943	0.368482	-1.601008
C	5.798867	-0.009796	-0.945993
O	3.765560	0.922852	0.448773
H	0.421636	1.277089	-2.221519
H	6.230181	0.835436	-0.405605
H	6.472508	-0.330911	-1.739790
H	5.629404	-0.828679	-0.242812
H	1.565410	0.828087	2.008982
H	1.793246	3.167189	2.807990
H	0.099550	4.868720	2.055651
H	-4.527358	2.525399	-2.641838
H	-4.458833	0.044854	-3.065259
H	-2.793103	-1.323991	-1.829711
H	-2.933326	3.504482	-1.018674
H	-1.681574	4.164528	0.482189
O	0.833309	-0.576466	-1.416039
C	1.832474	-1.588493	-1.665119
H	2.314610	-1.373749	-2.619389
H	2.584440	-1.604769	-0.872442
H	1.317018	-2.548116	-1.704275

TS1'

Re	0.159436	-0.743966	-0.370071
C	0.706235	-1.020279	-2.174669
O	1.045256	-1.183447	-3.277417
N	2.220002	-0.262412	0.252134
C	2.572274	1.049561	0.241975
C	3.873897	1.445374	0.569963
C	4.821090	0.486335	0.913878
C	4.449126	-0.857844	0.922824
C	3.142639	-1.187140	0.585749
C	1.512621	2.001111	-0.150846
N	0.312713	1.455397	-0.491076
C	-0.700773	2.268178	-0.846356
C	-0.558123	3.652376	-0.905283
C	0.667326	4.219575	-0.569790
C	1.710826	3.383072	-0.180533
C	-1.675160	-1.015072	-0.936224
O	-2.736945	-1.239619	-1.345595
C	0.271976	-2.637871	-0.054497
O	0.386921	-3.779946	0.140938
C	-1.387080	0.648678	2.294193
C	-2.526729	0.882143	1.739381
C	-3.528823	0.822183	0.783087
O	-4.488977	-0.135585	1.070732
C	-5.597617	-0.194710	0.165886
O	-3.669096	1.565822	-0.213406
H	-0.980337	1.047435	3.220416
H	-6.143299	0.751949	0.148779
H	-6.241139	-0.988220	0.545650

H	-5.261760	-0.433390	-0.845200
H	-1.661177	1.808132	-1.046909
H	-1.405343	4.259008	-1.198266
H	0.812699	5.292925	-0.600950
H	5.831948	0.784806	1.164567
H	5.149644	-1.641632	1.180709
H	2.813096	-2.217587	0.569477
H	4.148568	2.490679	0.551863
H	2.665013	3.807065	0.099805
O	-0.298779	-0.266948	1.773183
C	0.196272	-1.136859	2.807698
H	-0.475451	-1.990102	2.937287
H	1.191620	-1.480197	2.528605
H	0.262299	-0.579376	3.746016

P*cco*

Re	-0.356086	-0.532086	0.271207
N	0.121005	1.507316	0.999952
N	-2.019029	0.778861	-0.397113
C	-1.054074	-1.091283	1.914027
O	-1.466763	-1.452387	2.957997
C	1.564318	-1.267212	0.658993
O	2.038378	-1.864869	1.636173
C	-0.903929	-2.183688	-0.482857
O	-1.281124	-3.194478	-0.956391
C	2.081535	-0.331497	-1.591910
H	2.657445	-0.096867	-2.480626
C	2.542274	-0.952457	-0.493671
C	3.990401	-1.279532	-0.433158
O	4.688283	-1.206911	0.562500
O	4.479204	-1.664911	-1.638807
C	5.893617	-1.944187	-1.682113
H	6.464095	-1.061844	-1.386870
H	6.104395	-2.211266	-2.715273
H	6.133367	-2.772093	-1.013979
C	1.194970	1.788545	1.763903
H	1.837620	0.949735	2.000500
C	1.465559	3.068837	2.233374
H	2.338378	3.240137	2.850201
C	0.595609	4.104793	1.893019
H	0.772882	5.115628	2.239548
C	-0.512707	3.820946	1.100557
C	-0.736353	2.509202	0.668173
C	-1.907099	2.111256	-0.140494
C	-2.859264	3.024530	-0.609584
C	-3.943941	2.569091	-1.352000
H	-4.683873	3.267858	-1.724609
C	-4.062013	1.199952	-1.598494
H	-4.892644	0.794421	-2.162472
C	-3.084381	0.345242	-1.104506
H	-3.135417	-0.723122	-1.269227
H	-2.753861	4.080560	-0.400406
H	-1.202714	4.609773	0.833586
O	0.781692	0.107872	-1.614459
C	0.176214	0.259959	-2.923025
H	-0.699561	0.888783	-2.784021
H	0.891475	0.741755	-3.591176
H	-0.111286	-0.721943	-3.302189

TS2*ccb*

Re	0.720007	-0.889801	-0.259652
N	-0.196706	0.969604	-0.909016
N	2.151414	0.685316	0.297496
C	1.642942	-1.283423	-1.874311
O	2.214428	-1.508029	-2.864978
C	-0.704349	-2.086363	-0.782253
O	-1.550637	-2.806237	-1.120435
C	1.607251	-2.368951	0.608729
O	2.160765	-3.241371	1.147223
C	-1.780331	-0.013653	1.677135
H	-2.123019	-0.151476	2.701558
C	-2.496908	0.291328	0.614230
C	-3.919499	0.476922	0.609331
O	-4.508410	1.560683	0.539766
O	-4.615094	-0.705542	0.549186
C	-6.040721	-0.587220	0.410879
H	-6.469086	-0.055055	1.262495
H	-6.415313	-1.608939	0.373922
H	-6.297637	-0.054522	-0.506972
C	-1.525207	0.990307	-1.247823
H	-1.886713	0.101253	-1.741449
C	-2.150638	2.243721	-1.546304
H	-3.142855	2.244690	-1.978427
C	-1.512180	3.408763	-1.203752
H	-1.983662	4.368855	-1.381670
C	-0.224415	3.357902	-0.622459
C	0.401305	2.125710	-0.503697
C	1.759083	1.964634	0.053699
C	2.620039	3.035138	0.323401
C	3.881775	2.791373	0.853293
H	4.553440	3.616091	1.060634
C	4.270103	1.473755	1.106668
H	5.242155	1.235785	1.518650
C	3.376994	0.453331	0.811944
H	3.632817	-0.584506	0.982625
H	2.306013	4.047169	0.108459
H	0.269490	4.266953	-0.307717
O	-0.356823	-0.192691	1.619785
C	0.241843	-0.506635	2.893327
H	1.310659	-0.323404	2.796350
H	-0.173710	0.147212	3.661974
H	0.057643	-1.553788	3.147536

P*ccb*

Re	0.720007	-0.889801	-0.259652
N	-0.196706	0.969604	-0.909016
N	2.151414	0.685316	0.297496
C	1.642942	-1.283423	-1.874311
O	2.214428	-1.508029	-2.864978
C	-0.704349	-2.086363	-0.782253
O	-1.550637	-2.806237	-1.120435
C	1.607251	-2.368951	0.608729
O	2.160765	-3.241371	1.147223
C	-1.780331	-0.013653	1.677135
H	-2.123019	-0.151476	2.701558
C	-2.496908	0.291328	0.614230
C	-3.919499	0.476922	0.609331
O	-4.508410	1.560683	0.539766
O	-4.615094	-0.705542	0.549186
C	-6.040721	-0.587220	0.410879
H	-6.469086	-0.055055	1.262495
H	-6.415313	-1.608939	0.373922
H	-6.297637	-0.054522	-0.506972
C	-1.525207	0.990307	-1.247823
H	-1.886713	0.101253	-1.741449
C	-2.150638	2.243721	-1.546304
H	-3.142855	2.244690	-1.978427
C	-1.512180	3.408763	-1.203752
H	-1.983662	4.368855	-1.381670
C	-0.224415	3.357902	-0.622459
C	0.401305	2.125710	-0.503697
C	1.759083	1.964634	0.053699
C	2.620039	3.035138	0.323401
C	3.881775	2.791373	0.853293
H	4.553440	3.616091	1.060634
C	4.270103	1.473755	1.106668
H	5.242155	1.235785	1.518650
C	3.376994	0.453331	0.811944
H	3.632817	-0.584506	0.982625
H	2.306013	4.047169	0.108459
H	0.269490	4.266953	-0.307717
O	-0.356823	-0.192691	1.619785
C	0.241843	-0.506635	2.893327
H	1.310659	-0.323404	2.796350
H	-0.173710	0.147212	3.661974
H	0.057643	-1.553788	3.147536

TS2*ins*

C	1.584227	3.462817	0.064352
C	1.431972	2.073311	-0.000150
N	0.290993	1.515506	-0.485459
C	-0.710565	2.321042	-0.891165
C	-0.613967	3.706722	-0.864421
C	0.557033	4.288807	-0.380741
C	2.474311	1.126846	0.442105
N	2.143594	-0.189107	0.375567
C	3.047618	-1.107579	0.770959
C	4.311042	-0.769718	1.238916
C	4.664821	0.579022	1.293704
C	3.735039	1.533141	0.895351
Re	0.182682	-0.692895	-0.535050
C	0.303595	-2.600028	-0.343662
O	0.422725	-3.752833	-0.211825
C	-1.603153	-0.878957	-1.264515
O	-2.621544	-1.006834	-1.814014
C	0.941408	-0.835442	-2.246162
O	1.389204	-0.925575	-3.326000
C	-2.182753	0.031959	1.060646

C	-3.486041	0.585391	0.864457
O	-3.736610	1.730119	0.465359
C	-1.479683	-0.129946	2.157448
O	-4.491589	-0.331205	1.034765
C	-5.815065	0.116891	0.698362
H	-1.616614	1.832725	-1.220703
H	-1.447397	4.305263	-1.208249
H	0.667622	5.365761	-0.340091
H	2.492293	3.896916	0.459843
H	2.740099	-2.142279	0.695375
H	4.997397	-1.550227	1.541544
H	5.645141	0.883190	1.640612
H	3.992616	2.582717	0.926709
H	-1.728346	0.080236	3.199024
H	-6.118352	0.946937	1.340399
H	-5.860680	0.437082	-0.344007
H	-6.464366	-0.741842	0.859853
O	-0.144731	-0.580393	2.013995
C	0.237498	-1.614992	2.932170
H	1.313278	-1.756467	2.826170
H	0.007667	-1.312006	3.958013
H	-0.287295	-2.547586	2.699340

Pins

Re	0.292019	-0.218778	-0.967254
N	-1.368756	1.078136	-0.286196
C	-2.042932	2.942039	1.069865
H	-1.785104	3.718813	1.776736
O	-1.352459	-2.113079	-2.769129
C	0.354941	2.148502	1.004105
C	3.040490	2.217951	1.616599
H	4.103263	2.208904	1.822235
N	1.191592	1.242866	0.434995
C	-3.658264	1.802006	-0.287393
H	-4.662449	1.661060	-0.665571
C	-3.352895	2.816554	0.618607
C	0.660226	0.933958	-2.497268
C	-1.062521	2.061547	0.599495
C	2.502094	1.283404	0.740194
H	3.121843	0.535719	0.264877
C	2.187727	3.146245	2.213529
C	0.832598	3.109530	1.902960
H	0.154531	3.823221	2.350730
C	1.864610	-1.257067	-1.335203
C	2.943467	-2.287936	2.541418
H	3.956110	-1.938840	2.345762
C	-0.729649	-1.410914	-2.076764
C	-2.641698	0.950729	-0.706128
H	-2.836641	0.136185	-1.388580
C	0.765025	-1.891021	1.732966
O	0.891233	1.598256	-3.429391
O	2.830278	-1.872286	-1.563685
H	2.568956	3.887102	2.906258
H	-4.118469	3.498014	0.970086
H	2.663706	-2.042575	3.571128
H	2.896290	-3.371695	2.397126
O	2.092372	-1.616347	1.608579
C	-0.154410	-1.435598	0.851972
C	-1.538152	-1.845261	1.140721
O	-1.771739	-2.229553	2.438138
C	-3.100022	-2.686568	2.733802
H	-3.828703	-1.893773	2.553985
H	-3.359183	-3.553068	2.122045
H	-3.088805	-2.957018	3.788394
O	-2.463329	-1.845206	0.329678
H	0.518745	-2.513563	2.592858

Figure S14. PCM-B3LYP/6-31+G(d,p) (LANL2DZ for Re) optimized geometries in THF solution of the critical structures involved in the reaction between the complex [Re(OMe)$(CO)_3$(bipy)] (bipy = 2,2'-bipyridine) and methyl propiolate (HMAD, HC≡CCO_2Me). Relevant distances are given in angstroms.

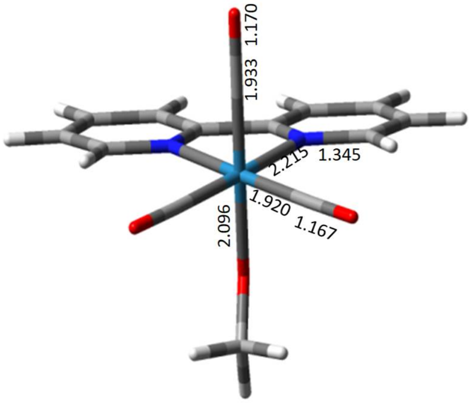

[Re(OMe)$(CO)_3$(bipy)]

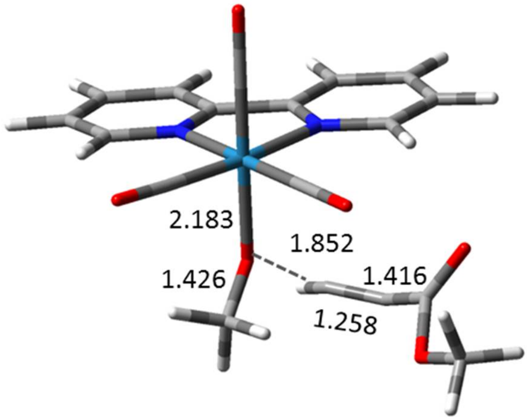

TS1

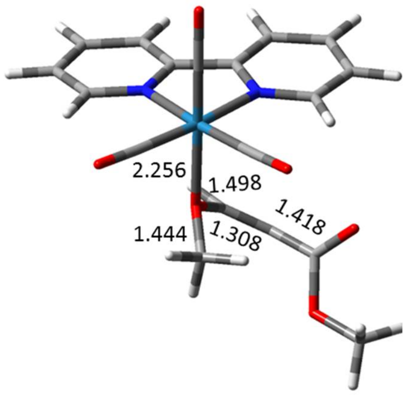

I1

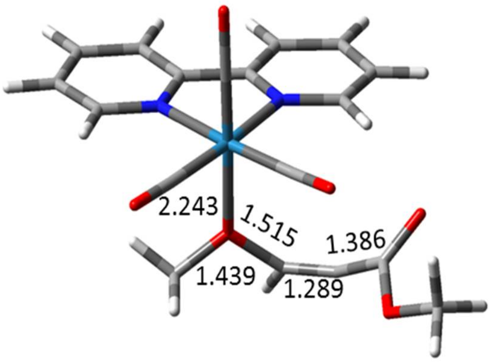

TS1'

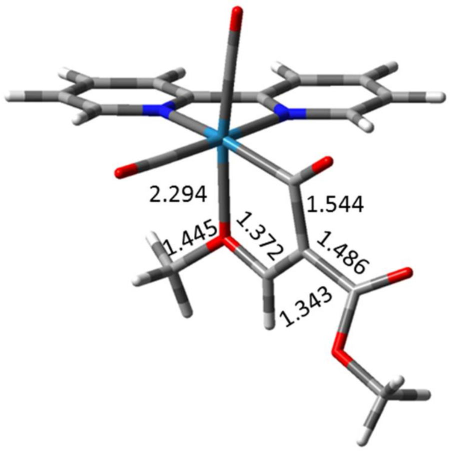

P*cco*

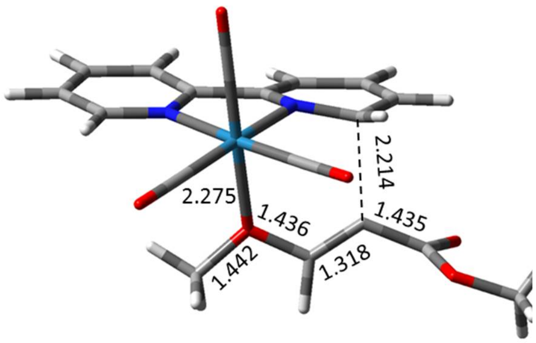

TS2*ccb*

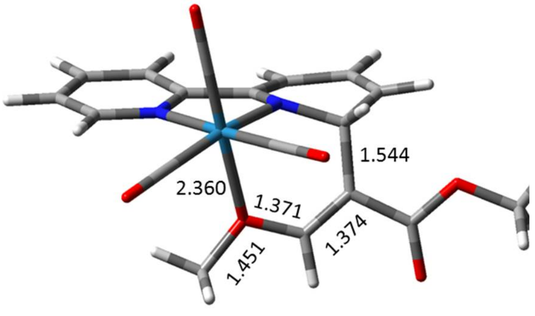

P*ccb*

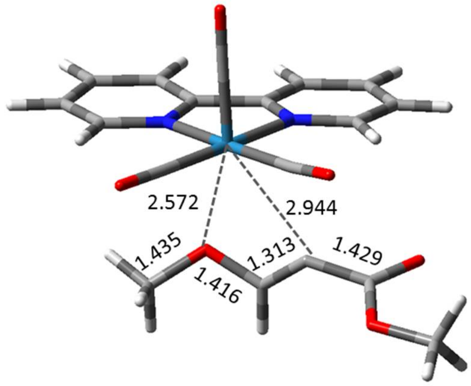

TS2*ins*

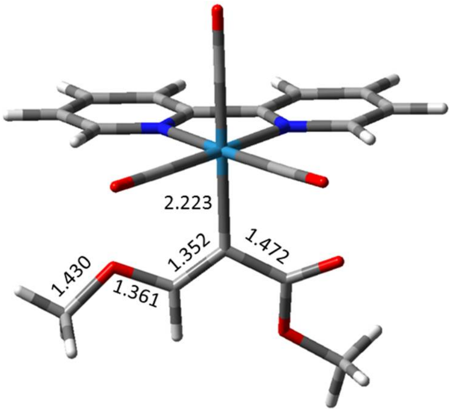

P*ins*

Table S43. PCM-B3LYP/6-31+G(d,p) (LANL2DZ for Re) energies without and with including thermal corrections (E and G, respectively), enthalpies (H), and entropies (S), and CPCM-DLPNO-CCSD(T)/def2-TZVPP//PCM-B3LYP/6-31+G(d,p) (LANL2DZ for Re) energies without and with including thermal corrections (E' and G', respectively) in THF solution of the critical structures involved in the reaction between the complex [Re(PPh$_2$)(CO)$_3$(bipy)] (bipy = 2,2'-bipyridine) and methyl propiolate (HMAD, HC≡CCO$_2$Me). All the values are given in hartree, except entropies that are in cal/K mol.[a]

Species	E	H	S	G	E'	G'[b]
[Re(PPh$_2$)(CO)$_3$(bipy)]	-1719.308861	-1718.908312	189.038	-1718.998130	-1715.584032	-1715.273301
HMAD	-305.215726	-305.136831	77.228	-305.173524	-304.738441	-304.696239
Reactants	-2024.524587	-2024.045143	266.266	-2024.171654	-2020.322473	-2019.969540
**TS1**	-2024.511051	-2024.031147	225.138	-2024.138117	-2020.314569	-2019.941635
**I1**	-2024.532967	-2024.050487	220.506	-2024.155256	-2020.343776	-2019.966065
**TS1'**	-2024.530736	-2024.049944	216.761	-2024.152934	-2020.340671	-2019.962869
**I1'**	-2024.536860	-2024.054702	218.671	-2024.158600	-2020.348808	-2019.970548
**TS2*cco***	-2024.534530	-2024.053418	214.029	-2024.155110	-2020.341578	-2019.962158
**P*cco***	-2024.559571	-2024.075675	215.346	-2024.177993	-2020.366016	-2019.984438
**TS2*ccb***	-2024.530715	-2024.049864	214.592	-2024.151824	-2020.341456	-2019.962565
**P*ccb***	-2024.560353	-2024.077303	217.000	-2024.180406	-2020.381387	-2020.001440
**TS2*ins***	-2024.512732	-2024.031404	216.487	-2024.134264	-2020.316123	-2019.937655
**P*ins***	-2024.566603	-2024.083988	221.600	-2024.189278	-2020.373572	-2019.996247

[a] Thermal magnitudes were computed in THF solution at 298.15 K and 1 atm. [b] For each species, G' was calculated as G' = G – E + E', in which G is the PCM-B3LYP/6-31+G(d,p) (LANL2DZ for Re) energy with including thermal corrections and E and E' are the PCM-B3LYP/6-31+G(d,p) (LANL2DZ for Re) and CPCM-DLPNO-CCSD(T)/def2-TZVPP//PCM-B3LYP/6-31+G(d,p) (LANL2DZ for Re) energies without including thermal corrections, respectively.

Table S44. PCM-B3LYP/6-31+G(d,p) (LANL2DZ for Re) relative energies without and with including thermal corrections (ΔE and ΔG, respectively), enthalpies (ΔH), and entropic contributions (TΔS), and CPCM-DLPNO-CCSD(T)/def2-TZVPP//PCM-B3LYP/6-31+G(d,p) (LANL2DZ for Re) relative energies without and with including thermal corrections (E' and G', respectively) in THF solution of the critical structures involved in the reaction between the complex [Re(PPh$_2$)(CO)$_3$(bipy)] (bipy = 2,2'-bipyridine) and methyl propiolate (HMAD, HC≡CCO$_2$Me). All the values are given in kcal/mol.[a]

Species	ΔE	ΔH	TΔS	ΔG	ΔE'	ΔG'
[Re(PPh$_2$)(CO)$_3$(bipy)] + HMAD	0.0	0.0	0.0	0.0	0.0	0.0
**TS1**	8.5	8.8	-12.3	21.0	5.0	17.5
**I1**	-5.3	-3.4	-13.6	10.3	-13.4	2.2
**TS1'**	-3.9	-3.0	-14.8	11.7	-11.4	4.2
**I1'**	-7.7	-6.0	-14.2	8.2	-16.5	-0.6
**TS2*cco***	-6.2	-5.2	-15.6	10.4	-12.0	4.6
**P*cco***	-22.0	-19.2	-15.2	-4.0	-27.3	-9.3
**TS2*ccb***	-3.8	-3.0	-15.4	12.4	-11.9	4.4
**P*ccb***	-22.4	-20.2	-14.7	-5.5	-37.0	-20.0
**TS2*ins***	7.4	8.6	-14.8	23.5	4.0	20.0
**P*ins***	-26.4	-24.4	-13.3	-11.1	-32.1	-16.8

[a] Thermal magnitudes were evaluated in THF solution at 298.15 K and 1 atm.

Table S45. PCM-B3LYP/6-31+G(d,p) (LANL2DZ for Re) optimized cartesian coordinates, in Å, for the critical structures involved in the reaction of the complex [Re(PPh_2)$(CO)_3$(bipy)] (bipy = 2,2'-bipyridine) towards methyl propiolate (HMAD, HC≡CCO_2Me).

[Re(PPh_2)$(CO)_3$(bipy)]

Re	-0.515602	-0.732362	-0.834506
C	-1.780748	-1.353318	-2.166330
O	-2.495990	-1.723568	-3.009854
C	0.584989	0.144330	-2.142791
O	1.248249	0.724102	-2.907190
C	0.504734	-2.356055	-0.981432
O	1.085033	-3.366474	-1.032291
N	-1.762389	-1.384394	0.855736
C	-1.739870	-2.625341	1.383210
C	-2.515209	-2.992509	2.475100
C	-3.347233	-2.036562	3.060226
C	-3.371489	-0.753743	2.526571
C	-2.568943	-0.446469	1.421199
C	-2.562465	0.877444	0.775077
N	-1.755579	1.000663	-0.315037
C	-1.729594	2.175018	-0.977334
C	-2.495436	3.269297	-0.596318
C	-3.317943	3.155104	0.524238
C	-3.345870	1.949536	1.216851
H	-1.078493	-3.335644	0.904962
H	-2.458600	-4.004660	2.854402
H	-3.965669	-2.284670	3.914551
H	-4.008351	0.002662	2.964148
H	-1.066956	2.225430	-1.830499
H	-2.437209	4.187052	-1.166839
H	-3.923692	3.989302	0.856956
H	-3.978569	1.838953	2.086635
P	1.076538	0.164339	1.035169
C	1.435658	1.940870	0.627166
C	0.568880	2.920846	1.151171
C	0.781881	4.281691	0.918909
C	1.882276	4.703648	0.167177
C	2.752651	3.745811	-0.363470
C	2.531098	2.384303	-0.139224
C	2.783287	-0.573008	0.903609
C	3.514204	-0.690545	2.101267
C	4.810676	-1.213077	2.117869
C	5.407952	-1.638833	0.928279
C	4.696165	-1.538695	-0.270288
C	3.401412	-1.010533	-0.281055
H	-0.281392	2.610558	1.753463
H	0.096437	5.011807	1.337975
H	2.058590	5.760761	-0.004219
H	3.609389	4.059551	-0.951981
H	3.218364	1.660612	-0.564013
H	3.056522	-0.366233	3.031039
H	5.350482	-1.289031	3.056796
H	6.413073	-2.048173	0.935386
H	5.149692	-1.866991	-1.200160
H	2.873684	-0.936439	-1.224906

TS1

Re	-0.377259	-0.385588	-1.363104
C	-1.061953	-1.535888	-2.768250
O	-1.451881	-2.200286	-3.640585
C	-0.510754	1.163990	-2.493727
O	-0.647903	2.117334	-3.149410
C	1.424833	-0.726766	-1.949890
O	2.509233	-0.975875	-2.295747
N	-0.505111	-1.985179	0.152147
C	0.528800	-2.772419	0.504737
C	0.398758	-3.824209	1.404270
C	-0.853369	-4.076891	1.961911
C	-1.916648	-3.239613	1.635120
C	-1.717170	-2.186678	0.736771
C	-2.773235	-1.225686	0.366947
N	-2.404921	-0.249032	-0.504587
C	-3.309946	0.685021	-0.858301
C	-4.617552	0.679183	-0.389900
C	-5.009191	-0.329296	0.490767
C	-4.076212	-1.286802	0.872494
H	1.488062	-2.552273	0.057916
H	1.276315	-4.406347	1.653521
H	-0.999061	-4.896909	2.654770
H	-2.889591	-3.402566	2.077896
H	-2.967025	1.445154	-1.547535
H	-5.305836	1.449685	-0.712625
H	-6.021531	-0.370439	0.874129
H	-4.358105	-2.072269	1.560768
P	0.564092	1.024750	0.580305
C	-0.800856	1.609691	1.672423
C	-1.188095	0.770625	2.735612
C	-2.213052	1.138686	3.612995
C	-2.873433	2.357755	3.449031
C	-2.508365	3.199398	2.391603
C	-1.491557	2.827019	1.510904
C	1.442214	2.565724	0.088843
C	2.155784	2.640914	-1.123679
C	2.971187	3.736165	-1.419041
C	3.091069	4.795596	-0.516156
C	2.392483	4.737709	0.694395
C	1.586132	3.639006	0.993628
H	-0.680920	-0.179532	2.880172
H	-2.486064	0.475526	4.427480
H	-3.658921	2.652009	4.136933
H	-3.019299	4.146902	2.250693
H	-1.220697	3.493845	0.699320
H	2.084094	1.835284	-1.844463
H	3.507115	3.761560	-2.362440
H	3.720967	5.647719	-0.748454
H	2.478742	5.547950	1.411573
H	1.069081	3.611917	1.946428
C	2.134178	-0.026270	2.431906
C	3.030948	-0.835599	2.170948
C	3.884007	-1.801741	1.582500
O	3.574790	-2.977998	1.373886
O	5.112360	-1.306635	1.287514
C	6.045685	-2.248956	0.725167
H	1.576619	0.571434	3.123837
H	5.689041	-2.609544	-0.241384
H	6.183476	-3.095140	1.400064
H	6.976000	-1.697035	0.607256

I1

Re	-0.118626	-0.712099	-1.099802
C	-0.790476	-2.028676	-2.364252
O	-1.159962	-2.816093	-3.131463
C	0.405336	0.425105	-2.557166
O	0.658534	1.126277	-3.452212
C	1.618534	-1.567250	-1.177972
O	2.647432	-2.085766	-1.313969
N	-1.070340	-1.778427	0.591506
C	-0.444025	-2.696084	1.354153
C	-1.101901	-3.396799	2.361824
C	-2.449922	-3.141038	2.596380
C	-3.094257	-2.179429	1.822340
C	-2.384417	-1.505318	0.825618
C	-2.992944	-0.470366	-0.032195
N	-2.162222	0.115009	-0.934461
C	-2.650795	1.075271	-1.744313
C	-3.978075	1.482333	-1.712570
C	-4.841480	0.877081	-0.799946
C	-4.340472	-0.102425	0.050017
H	0.614788	-2.860053	1.176015
H	-0.549977	-4.121671	2.945993
H	-2.992694	-3.670508	3.370502
H	-4.138677	-1.958601	1.995582
H	-1.951931	1.516795	-2.442407
H	-4.317636	2.252614	-2.392944
H	-5.885164	1.161279	-0.749156
H	-4.993284	-0.580048	0.767627
P	0.717804	0.929756	0.681015
C	-0.689221	1.935114	1.323104
C	-1.407037	1.476301	2.439823
C	-2.500371	2.196222	2.929780
C	-2.899811	3.378028	2.303399

C	-2.206625	3.831517	1.176485
C	-1.113164	3.114702	0.687597
C	1.950194	2.195048	0.140845
C	2.821017	1.940128	-0.929149
C	3.808139	2.865402	-1.280497
C	3.943232	4.056199	-0.564372
C	3.090630	4.313994	0.513498
C	2.106522	3.390047	0.866093
H	-1.111316	0.556615	2.932582
H	-3.033022	1.833880	3.802452
H	-3.742835	3.941201	2.688345
H	-2.515410	4.744675	0.679311
H	-0.578344	3.484114	-0.180674
H	2.741352	1.017238	-1.490773
H	4.469910	2.652542	-2.112955
H	4.706718	4.775757	-0.839818
H	3.190686	5.232423	1.082234
H	1.453347	3.604188	1.704319
C	1.452097	0.249766	2.264682
C	2.290294	-0.754420	2.453681
C	2.982227	-1.785614	1.813034
O	2.614746	-2.979024	1.700642
O	4.259282	-1.415584	1.422076
C	5.103872	-2.475756	0.958972
H	1.118735	0.878152	3.095016
H	4.715171	-2.911921	0.036039
H	5.199613	-3.260216	1.714223
H	6.074939	-2.016440	0.775173

TS1′

Re	0.117955	-0.530847	-1.203112
C	-0.168013	-1.835410	-2.614041
O	-0.317535	-2.610300	-3.464480
C	0.424689	0.836709	-2.518963
O	0.548435	1.666592	-3.326973
C	2.007627	-0.975028	-1.222365
O	3.122626	-1.280581	-1.328211
N	-0.584043	-1.946430	0.341538
C	0.226360	-2.762039	1.044603
C	-0.260288	-3.649684	1.999888
C	-1.630118	-3.696511	2.244379
C	-2.469378	-2.848322	1.526834
C	-1.924857	-1.980337	0.576935
C	-2.746393	-1.062533	-0.235266
N	-2.064704	-0.238452	-1.073945
C	-2.755691	0.622575	-1.847584
C	-4.142629	0.690587	-1.842750
C	-4.851680	-0.163338	-0.998131
C	-4.144677	-1.042818	-0.186049
H	1.293055	-2.698514	0.869198
H	0.442192	-4.272708	2.537860
H	-2.042292	-4.374774	2.982134
H	-3.535076	-2.856754	1.711379
H	-2.168643	1.261851	-2.493567
H	-4.645536	1.395340	-2.492343
H	-5.934092	-0.147532	-0.973117
H	-4.673948	-1.716674	0.473425
P	0.445825	1.078952	0.761424
C	-1.133516	1.441644	1.638948
C	-1.430665	0.788321	2.845028
C	-2.651234	1.008047	3.491671
C	-3.593281	1.875119	2.935627
C	-3.311943	2.521407	1.726978
C	-2.094276	2.303961	1.081515
C	1.131939	2.743949	0.354335
C	2.172365	2.827123	-0.586235
C	2.778070	4.051602	-0.871434
C	2.354852	5.213181	-0.219721
C	1.331765	5.140004	0.728348
C	0.728091	3.913419	1.019379
H	-0.705266	0.114287	3.287135
H	-2.859530	0.504450	4.429504
H	-4.538840	2.049882	3.437824
H	-4.039822	3.195720	1.288606
H	-1.886581	2.815915	0.147350
H	2.513381	1.934655	-1.099604
H	3.576176	4.097599	-1.604860
H	2.820108	6.166024	-0.447532
H	1.002582	6.034809	1.245929
H	-0.061334	3.872691	1.761123
C	1.569239	0.472725	2.113352
C	2.465459	-0.470310	1.983337
C	3.362311	-1.475755	1.806010
O	3.161144	-2.717023	1.953222
O	4.629777	-1.013575	1.427510
H	1.416315	1.062225	3.026038
C	5.521629	-2.010264	0.923494
H	5.150698	-2.429146	-0.017085
H	5.665837	-2.818542	1.643639
H	6.467662	-1.496413	0.748521

I1′

Re	-0.380196	-0.175852	-1.422277
C	-1.235873	-1.023482	-2.945737
O	-1.725823	-1.517029	-3.875480
C	-0.368336	1.495817	-2.366500
O	-0.430233	2.514111	-2.930443
C	1.354240	-0.616131	-2.181694
O	2.336103	-0.861777	-2.749958
N	-0.729804	-1.957843	-0.152953
C	0.147777	-2.971596	-0.017484
C	-0.111787	-4.080676	0.781999
C	-1.316077	-4.140443	1.478957
C	-2.222152	-3.091352	1.348546
C	-1.912691	-2.011264	0.517167
C	-2.828955	-0.873829	0.301798
N	-2.357216	0.125854	-0.488678
C	-3.144695	1.192167	-0.733415
C	-4.432377	1.307417	-0.227255
C	-4.932523	0.278635	0.570378
C	-4.120147	-0.817498	0.839037
H	1.089506	-2.855625	-0.535854
H	0.628167	-4.867292	0.856455
H	-1.547316	-4.983234	2.119677
H	-3.158936	-3.115962	1.887991
H	-2.724961	1.963007	-1.365666
H	-5.023935	2.182756	-0.463862
H	-5.936235	0.327505	0.974927
H	-4.491708	-1.625986	1.453553
P	0.754050	0.819917	0.647116
C	-0.417474	0.986680	2.060861
C	-0.417758	0.021806	3.079701
C	-1.355910	0.080153	4.115340
C	-2.308553	1.100218	4.141785
C	-2.319266	2.064607	3.128721
C	-1.384758	2.006193	2.094169
C	1.481186	2.509160	0.455126
C	2.151502	2.820838	-0.739233
C	2.813634	4.040529	-0.889036
C	2.811222	4.974139	0.150852
C	2.152029	4.672923	1.345240
C	1.491672	3.450280	1.497316
H	0.319518	-0.774113	3.066770
H	-1.337886	-0.670956	4.898672
H	-3.035156	1.147912	4.946175
H	-3.057067	2.860378	3.143260
H	-1.402076	2.761585	1.314625
H	2.173277	2.103888	-1.552631
H	3.326286	4.260163	-1.819225
H	3.323389	5.923479	0.033149
H	2.149483	5.388457	2.160984
H	0.992115	3.230467	2.433637
C	2.132804	-0.172075	1.309029
C	2.658542	-1.200743	0.637121
C	3.727703	-2.028028	1.085506
O	3.614256	-3.106893	1.693206
O	4.965557	-1.595753	0.659482
C	6.061652	-2.490036	0.895652
H	2.479983	0.198994	2.283588
H	5.914004	-3.437207	0.370605
H	6.182829	-2.691788	1.962259
H	6.943108	-1.980135	0.508265

TS2*cco*

Re	0.004719	-0.523551	-1.266523
C	-0.315754	-1.847511	-2.633875
O	-0.479722	-2.638858	-3.472940
C	0.306957	0.764144	-2.642013
O	0.421873	1.548503	-3.503199
C	1.927584	-1.041202	-1.274861
O	2.954883	-1.418082	-1.688257
N	-0.712439	-1.928950	0.305471
C	0.071051	-2.822904	0.940237
C	-0.434736	-3.753862	1.841097
C	-1.802916	-3.759414	2.105836
C	-2.614145	-2.828530	1.464838
C	-2.047041	-1.919442	0.565788
C	-2.841745	-0.912030	-0.163688
N	-2.151337	-0.123961	-1.030193
C	-2.821375	0.817133	-1.726212

C	-4.191631	1.009209	-1.607463
C	-4.909209	0.197040	-0.728875
C	-4.224488	-0.767722	0.000699
H	1.131341	-2.761899	0.732446
H	0.240812	-4.451264	2.319325
H	-2.232373	-4.471829	2.800335
H	-3.677887	-2.812954	1.657976
H	-2.230986	1.420828	-2.402599
H	-4.676726	1.775936	-2.197949
H	-5.980333	0.312663	-0.612589
H	-4.760173	-1.404083	0.692033
P	0.508456	1.077473	0.648034
C	-0.885983	1.316653	1.824633
C	-0.930324	0.602716	3.031347
C	-2.016423	0.747213	3.900919
C	-3.069166	1.604706	3.574929
C	-3.041772	2.307397	2.365197
C	-1.961008	2.160619	1.495229
C	1.089197	2.795685	0.288825
C	1.934536	2.995818	-0.814439
C	2.468450	4.257804	-1.082713
C	2.173803	5.336667	-0.244716
C	1.342959	5.145771	0.862030
C	0.806975	3.883143	1.130569
H	-0.114369	-0.060555	3.296326
H	-2.031809	0.196549	4.835288
H	-3.905617	1.724860	4.254434
H	-3.858514	2.970601	2.101249
H	-1.949026	2.710710	0.559485
H	2.183140	2.163461	-1.462549
H	3.116943	4.394463	-1.940827
H	2.588899	6.316861	-0.452274
H	1.114582	5.976105	1.521853
H	0.164333	3.750319	1.992488
C	1.863235	0.328632	1.609540
C	2.494115	-0.714356	1.075204
C	3.572040	-1.510329	1.560986
O	3.454535	-2.574301	2.189940
O	4.794755	-1.077250	1.120579
C	5.905798	-1.949787	1.381296
H	2.105262	0.811328	2.561886
H	5.775247	-2.905779	0.869084
H	6.780736	-1.430641	0.993073
H	6.017722	-2.130737	2.452241

P*cco*

Re	-0.114439	-0.733028	-1.140507
C	-0.385627	-2.325212	-2.168365
O	-0.509240	-3.306412	-2.795617
C	0.512358	0.100729	-2.723019
O	0.860390	0.592194	-3.736566
C	1.811216	-1.438181	-0.620121
O	2.535541	-2.282601	-1.174040
N	-1.101393	-1.665997	0.630901
C	-0.482022	-2.498599	1.492251
C	-1.137689	-3.100836	2.559426
C	-2.491592	-2.830204	2.755173
C	-3.138101	-1.974662	1.869907
C	-2.425425	-1.406066	0.807154
C	-3.049532	-0.521811	-0.195452
N	-2.226954	-0.073616	-1.182177
C	-2.744729	0.733363	-2.133933
C	-4.078014	1.120288	-2.153670
C	-4.929906	0.654930	-1.149157
C	-4.405889	-0.172460	-0.163502
H	0.570442	-2.682504	1.315871
H	-0.589825	-3.764634	3.215971
H	-3.036220	-3.278233	3.577642
H	-4.188920	-1.755838	2.002514
H	-2.054234	1.062102	-2.899419
H	-4.435259	1.765789	-2.946055
H	-5.977986	0.927919	-1.135866
H	-5.047023	-0.548040	0.622305
P	0.608584	1.098237	0.409288
C	-0.535629	1.556772	1.773154
C	-1.755229	2.163560	1.427485
C	-2.677621	2.515323	2.413264
C	-2.397530	2.256321	3.758709
C	-1.191693	1.646438	4.111446
C	-0.263612	1.298589	3.124714
C	1.307384	2.722932	-0.128150
C	2.042169	2.781824	-1.323183
C	2.627391	3.981038	-1.734604
C	2.480917	5.136170	-0.961488
C	1.752581	5.084562	0.229544
C	1.166723	3.886093	0.645253
H	-1.982194	2.360906	0.384198
H	-3.613540	2.984810	2.130940
H	-3.114913	2.527682	4.525453
H	-0.968306	1.443173	5.153548
H	0.667002	0.825285	3.416056
H	2.160532	1.894574	-1.932331
H	3.190426	4.010876	-2.660808
H	2.933626	6.067984	-1.282869
H	1.637543	5.975813	0.836480
H	0.604087	3.861367	1.571429
C	2.027423	0.288541	1.225778
C	2.448386	-0.859899	0.676949
C	3.548194	-1.670620	1.284476
O	3.343191	-2.722755	1.867418
O	4.763474	-1.134251	1.111573
C	5.878298	-1.906172	1.616842
H	2.514896	0.741541	2.084357
H	5.902724	-2.882457	1.130762
H	6.764959	-1.328346	1.367820
H	5.788731	-2.034902	2.696241

TS2*ccb*

Re	-0.594378	-0.167720	-1.453891
C	-1.281566	-1.257751	-2.911441
O	-1.673018	-1.897968	-3.796351
C	-1.594836	1.355319	-2.080667
O	-2.253680	2.255590	-2.419593
C	0.933472	0.316871	-2.528371
O	1.861477	0.574370	-3.181286
N	0.286416	-1.895270	-0.440267
C	1.614669	-2.213268	-0.548078
C	2.095015	-3.422624	0.043473
C	1.291626	-4.113580	0.916531
C	-0.016826	-3.651801	1.175639
C	-0.488075	-2.553047	0.469843
C	-1.870761	-2.053548	0.600331
N	-2.177981	-0.950091	-0.132170
C	-3.425648	-0.441375	-0.068018
C	-4.426901	-1.007622	0.707251
C	-4.125763	-2.145033	1.459537
C	-2.839049	-2.665571	1.406847
H	2.115050	-1.826113	-1.422625
H	3.106485	-3.743475	-0.170230
H	1.648568	-5.015288	1.401167
H	-0.652359	-4.173849	1.877102
H	-3.615004	0.438849	-0.668260
H	-5.414383	-0.563718	0.716725
H	-4.881454	-2.617836	2.075471
H	-2.584502	-3.546294	1.980276
P	0.400110	1.080014	0.523084
C	-0.599852	0.939213	2.065020
C	-0.252014	0.010038	3.055145
C	-1.036853	-0.123941	4.204920
C	-2.175710	0.665987	4.373800
C	-2.533559	1.588761	3.384481
C	-1.755103	1.720271	2.234360
C	0.639510	2.901585	0.354373
C	0.708487	3.513378	-0.905505
C	0.952421	4.885232	-1.016565
C	1.131817	5.661728	0.129958
C	1.074198	5.059535	1.390612
C	0.831508	3.689600	1.502372
H	0.636392	-0.599532	2.931603
H	-0.751763	-0.840008	4.968151
H	-2.779166	0.568050	5.269463
H	-3.415682	2.207101	3.509517
H	-2.033316	2.446094	1.477045
H	0.573312	2.926211	-1.805784
H	0.999006	5.343165	-1.998963
H	1.316331	6.727352	0.043460
H	1.215143	5.654930	2.286043
H	0.780873	3.234536	2.485932
C	2.068091	0.530605	1.039461
C	2.671007	-0.581276	0.618831
C	3.958493	-1.067913	1.024991
O	4.152572	-1.882327	1.933967
O	4.969448	-0.674116	0.190023
C	6.257285	-1.265283	0.438425
H	2.526133	1.235646	1.743968
H	6.595872	-1.045618	1.452242
H	6.929412	-0.817024	-0.291213
H	6.217803	-2.348225	0.302132

P*ccb*

Re	-0.639268	-0.305161	-1.456611
C	-1.307763	-1.589033	-2.753766
O	-1.695050	-2.348157	-3.542289
C	-1.599946	1.123644	-2.331862
O	-2.209287	1.984983	-2.833925
C	0.908471	0.017812	-2.557826
O	1.853471	0.191153	-3.218109
N	0.236260	-1.800603	-0.156135
C	1.682966	-2.015135	-0.081540
C	1.997819	-3.384269	0.498002
C	1.161170	-3.924479	1.415361
C	-0.099400	-3.306722	1.707531
C	-0.538173	-2.319921	0.839061
C	-1.928360	-1.825234	0.865624
N	-2.244384	-0.874866	-0.055968
C	-3.501470	-0.381662	-0.098235
C	-4.499276	-0.797133	0.768713
C	-4.187539	-1.770772	1.724214
C	-2.900297	-2.284052	1.767873
H	2.083064	-1.967175	-1.101157
H	2.902151	-3.884697	0.175681
H	1.404082	-4.875182	1.881287
H	-0.745153	-3.711131	2.473546
H	-3.694259	0.366991	-0.855637
H	-5.490914	-0.369551	0.692564
H	-4.939863	-2.123204	2.420325
H	-2.638285	-3.042571	2.492488
P	0.353624	1.084757	0.400551
C	-0.652815	1.274073	1.931123
C	-0.449024	0.452101	3.047128
C	-1.239966	0.610816	4.189470
C	-2.241884	1.582483	4.220383
C	-2.459079	2.395098	3.102514
C	-1.673451	2.238270	1.960744
C	0.922743	2.809542	0.077623
C	0.885509	3.345272	-1.216521
C	1.358724	4.639245	-1.459308
C	1.877580	5.402970	-0.412720
C	1.914965	4.876352	0.883251
C	1.438561	3.588713	1.127975
H	0.319664	-0.314150	3.023468
H	-1.071034	-0.025230	5.051659
H	-2.849866	1.708846	5.109198
H	-3.237439	3.151257	3.120442
H	-1.840218	2.880770	1.101834
H	0.489306	2.757502	-2.035876
H	1.321179	5.043448	-2.464806
H	2.247898	6.404405	-0.601587
H	2.312622	5.467075	1.700965
H	1.460525	3.193830	2.138873
C	1.908432	0.329745	0.993127
C	2.397648	-0.886253	0.695070
C	3.820480	-1.125664	1.121502
O	4.321634	-0.714931	2.154767
O	4.499856	-1.823069	0.196276
H	5.963203	-2.666912	1.420692
C	5.886627	-2.097453	0.494104
H	2.541759	0.999826	1.570590
H	6.253791	-2.676655	-0.349442
H	6.440877	-1.163466	0.591674

TS2*ins*

Re	-0.907213	-0.349117	-1.155353
C	-2.148648	-0.128670	-2.555647
O	-2.920431	-0.017912	-3.430800
C	0.475867	-0.623259	-2.452809
O	1.307260	-0.719961	-3.265383
C	-1.251892	-2.246379	-1.091324
O	-1.505492	-3.381357	-1.086513
N	-2.453059	0.350712	0.301172
C	-3.357183	-0.465533	0.873433
C	-4.372988	-0.001307	1.701186
C	-4.453873	1.364975	1.961597
C	-3.519240	2.215196	1.379158
C	-2.528345	1.685458	0.545992
C	-1.520992	2.520162	-0.134927
N	-0.637360	1.854210	-0.921333
C	0.309619	2.558362	-1.573126
C	0.411279	3.940132	-1.492387
C	-0.499625	4.633241	-0.693300
C	-1.468922	3.914107	-0.005693
H	-3.250316	-1.519377	0.662361
H	-5.072284	-0.705624	2.132628
H	-5.228467	1.764263	2.605307
H	-3.565924	3.279468	1.565225
H	0.995097	1.983955	-2.182361
H	1.187533	4.453744	-2.044823
H	-0.454581	5.711805	-0.605094
H	-2.180207	4.430696	0.624144
P	1.652863	-0.136184	0.502256
C	2.265562	1.372376	1.384313
C	1.310898	2.153298	2.061204
C	1.686676	3.322015	2.725025
C	3.021432	3.741090	2.712352
C	3.976365	2.977445	2.036838
C	3.601682	1.802754	1.377143
C	3.206140	-0.988964	-0.014823
C	3.697661	-2.115215	0.664558
C	4.853935	-2.763827	0.222070
C	5.533907	-2.298523	-0.906448
C	5.047045	-1.185076	-1.597801
C	3.888214	-0.540853	-1.159187
H	0.273308	1.833936	2.082000
H	0.939142	3.905933	3.251564
H	3.313977	4.650039	3.226904
H	5.014884	3.290309	2.025658
H	4.355971	1.217608	0.863842
H	3.174669	-2.490162	1.536954
H	5.219808	-3.633156	0.758141
H	6.430201	-2.804224	-1.249277
H	5.561457	-0.826336	-2.482967
H	3.505961	0.307145	-1.718346
C	0.899503	-1.179514	1.770411
C	-0.291161	-1.689276	1.434708
C	-1.088450	-2.591105	2.227865
O	-2.048897	-2.272858	2.939666
O	-0.768084	-3.902953	2.012337
C	-1.640610	-4.875015	2.612837
H	1.426703	-1.366415	2.711146
H	-1.225027	-5.844940	2.345581
H	-2.654309	-4.777042	2.218817
H	-1.664553	-4.757695	3.697603

P*ins*

Re	-1.157838	0.007144	-1.053544
C	-2.338708	-0.009906	-2.605396
O	-3.016159	-0.039028	-3.552826
C	-0.029450	1.297743	-1.923539
O	0.572647	2.146541	-2.448329
C	-0.114987	-1.436515	-1.774963
O	0.515442	-2.325645	-2.186990
N	-2.636979	-1.160906	0.108411
C	-2.667799	-2.507810	0.152487
C	-3.642940	-3.214704	0.844563
C	-4.628111	-2.501374	1.527969
C	-4.594891	-1.111298	1.498659
C	-3.587069	-0.457180	0.780807
C	-3.477655	1.012907	0.702953
N	-2.436095	1.490959	-0.025999
C	-2.269565	2.823773	-0.126486
C	-3.126580	3.736400	0.475984
C	-4.209278	3.253835	1.210715
C	-4.383392	1.878932	1.324115
H	-1.883223	-3.020022	-0.388239
H	-3.622935	-4.296436	0.842544
H	-5.406981	-3.016583	2.077353
H	-5.343827	-0.541150	2.030984
H	-1.424205	3.157160	-0.713558
H	-2.944396	4.797636	0.360338
H	-4.905175	3.933893	1.687202
H	-5.215778	1.483913	1.889693
P	2.743913	0.148687	-0.439865
C	3.783492	1.525984	0.241526
C	3.252016	2.484815	1.118810
C	4.016993	3.580980	1.532868
C	5.326427	3.739379	1.074131
C	5.867065	2.792697	0.196526
C	5.101572	1.701609	-0.218811
C	3.834863	-1.317008	-0.099317
C	3.857547	-2.348303	-1.050897
C	4.649706	-3.484453	-0.856604
C	5.426125	-3.605604	0.298027
C	5.408361	-2.586569	1.256984
C	4.618010	-1.452157	1.059367
H	2.237224	2.371355	1.485316
H	3.588276	4.307479	2.215719
H	5.918814	4.589285	1.396197
H	6.882528	2.906632	-0.168196
H	5.535199	0.977813	-0.902444

H	3.247342	-2.261428	-1.944238
H	4.660091	-4.270585	-1.605592
H	6.043962	-4.484341	0.448858
H	6.009497	-2.676830	2.155808
H	4.615645	-0.661544	1.803716
C	1.515320	-0.054429	0.910453
C	0.171467	-0.091769	0.770071
C	-0.567026	-0.314609	2.039059
O	-1.191266	0.534341	2.664812
O	-0.543508	-1.613834	2.427110
C	-1.337717	-1.947428	3.582167
H	1.962664	-0.189834	1.901006
H	-1.013586	-1.372004	4.450071
H	-2.392564	-1.746036	3.382591
H	-1.177981	-3.011248	3.748359

Figure S15. PCM-B3LYP/6-31+G(d,p) (LANL2DZ for Re) optimized geometries in THF solution of the critical structures involved in the reaction between the complex $[Re(PPh_2)(CO)_3(bipy)]$ (bipy = 2,2'-bipyridine) and methyl propiolate (HMAD, $HC{\equiv}CCO_2Me$). Relevant distances are given in angstroms.

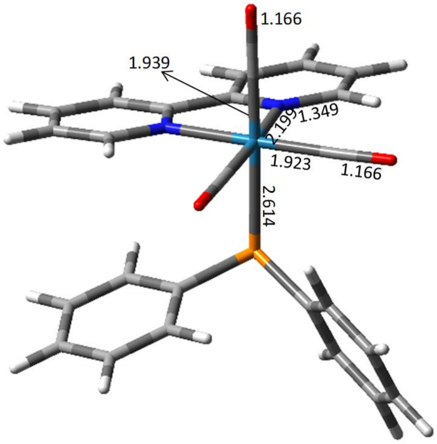

$[Re(PPh_2)(CO)_3(bipy)]$

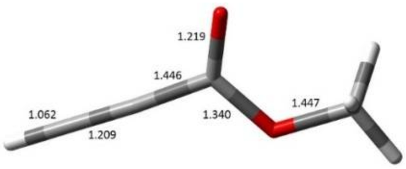

HMAD

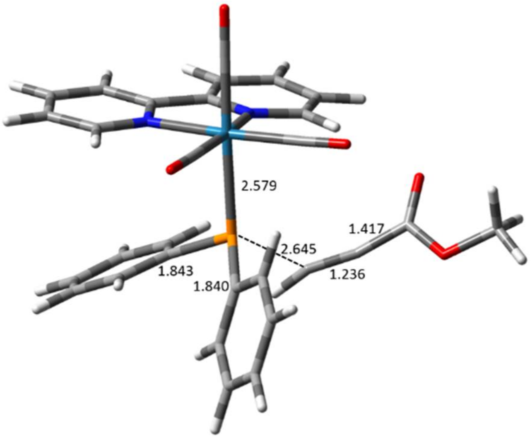

TS1

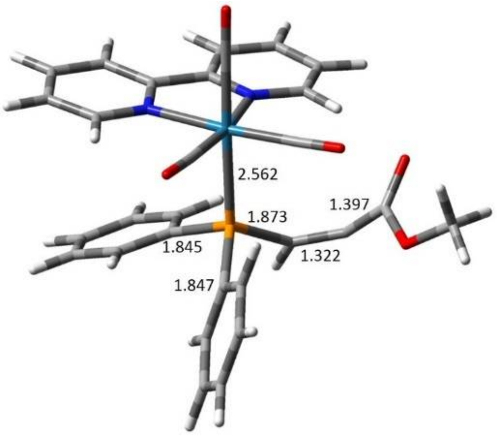

I1

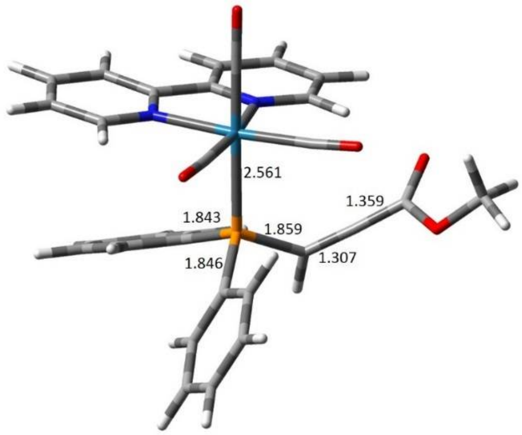

TS1'

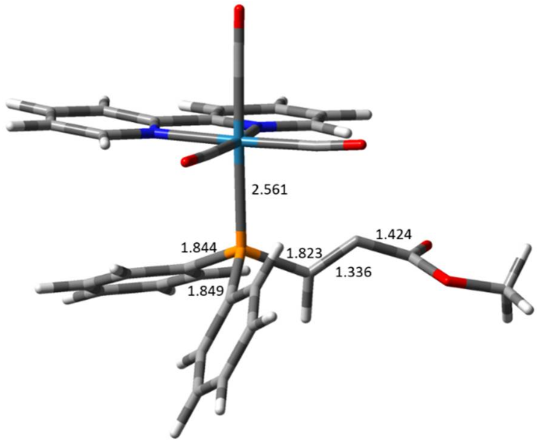

I1'

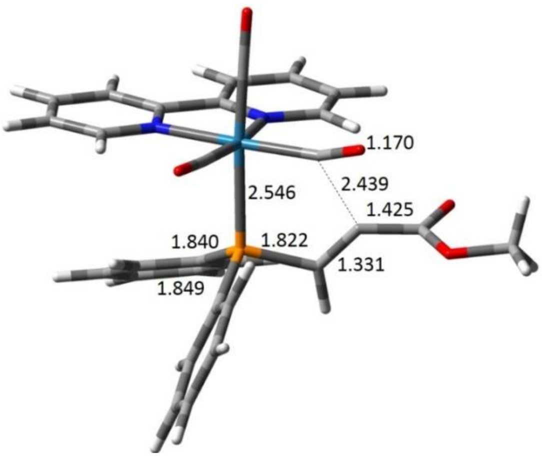

TS2*cco*

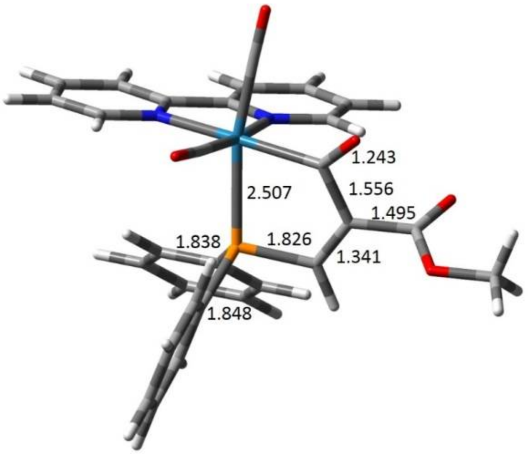

P*cco*

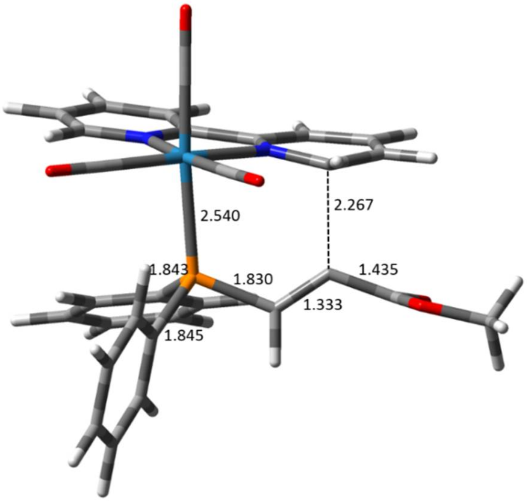

TS2*ccb*

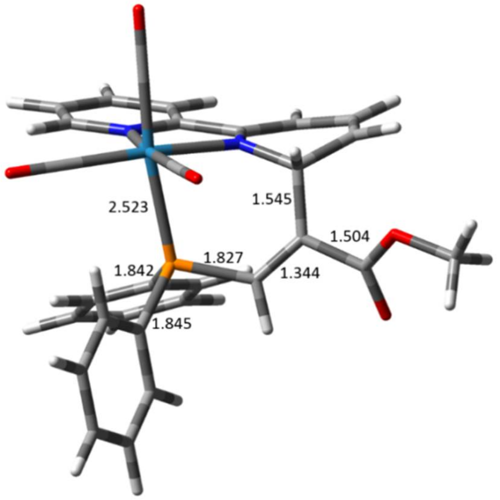

P*ccb*

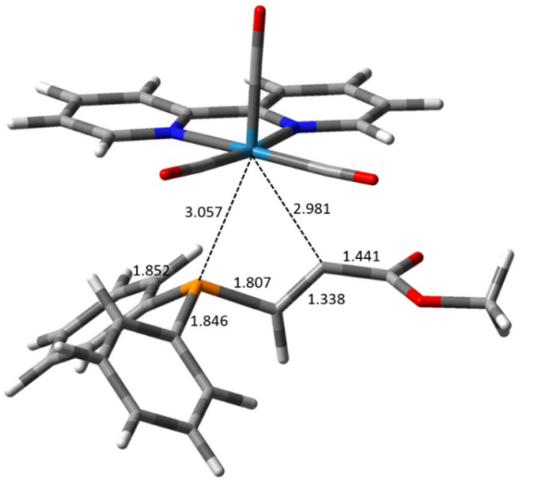

TS2*ins*

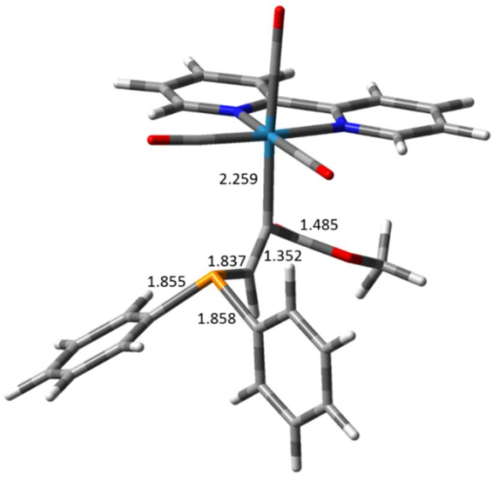

P*ins*

Figure S16. Pictures of the HOMO of the reactant complexes [ReY(CO)$_3$(bipy)] (Y = PHMe, PPH$_2$, PMePh).

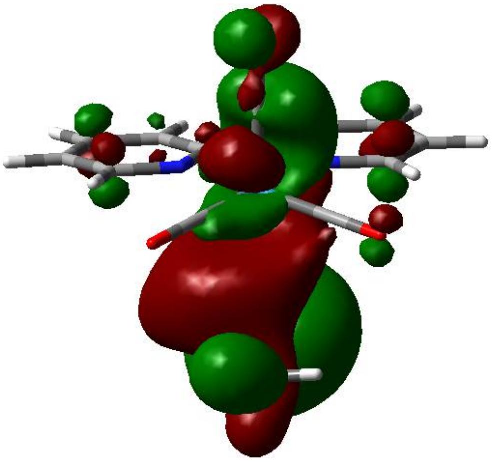

PHMe

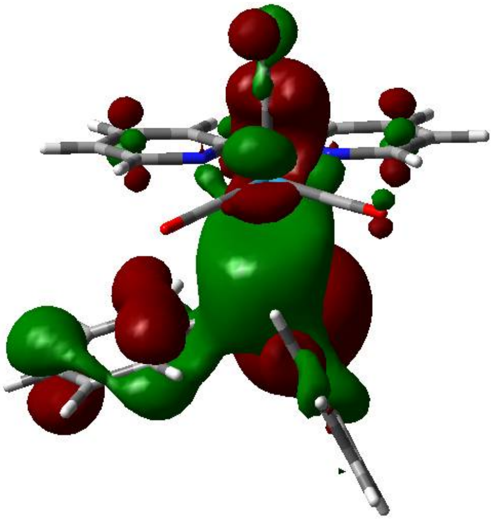

PPh$_2$

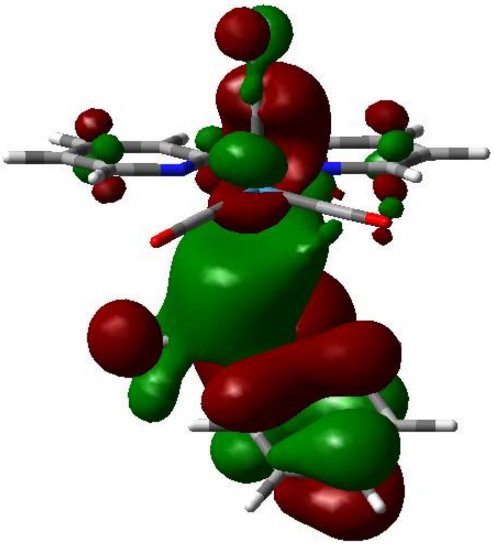

PMePh

Table S46. CPCM-DLPNO-CCSD(T)/def2-TZVPP//PCM-B3LYP/6-31+G(d,p) (LANL2DZ for Re) relative Gibbs energies, in kcal/mol, of all the species found for each nucleophilic ligand containing a nitrogen atom referred to the analogous ones obtained for the NH_2 ligand in the reaction between [ReY(CO)$_3$(bipy)] (bipy = 2,2'-bipyridine) and methyl propiolate (HMAD, HC≡CCO$_2$Me).

Y	**TS1**	**I1**	**TS1'**	**I1'**	**TS2*cco***	**P*cco***	**P*ccoh***	**TS1*b***	**I1*b***	**I1'*b***	**TS2*ccb***	**P*ccb***	**TS2*ins***	**P*ins***
NH_2	0.0	0.0	0.0	0.0	0.0	0.0	0.0	0.0	0.0	0.0	0.0	0.0	0.0	0.0
NHMe	-1.3	0.8	-4.2	-2.2	-4.4	-4.4	-7.4	-5.3	-3.8	-4.0	-4.3	-4.2	-2.8	-2.1
NH*p*Tol	3.9	14.8	8.5			7.8	3.8	2.1	9.9	11.3	10.6	10.0	9.2	6.7

Table S47. CPCM-DLPNO-CCSD(T)/def2-TZVPP//PCM-B3LYP/6-31+G(d,p) (LANL2DZ for Re) relative Gibbs energies, in kcal/mol, of all the species found for each nucleophilic ligand containing an oxygen atom referred to the analogous ones obtained for the OH ligand in the reaction between [ReY(CO)$_3$(bipy)] (bipy = 2,2'-bipyridine) and methyl propiolate (HMAD, HC≡CCO$_2$Me).

Y	**TS1**	**I1**	**TS1'**	**I1'**	**TS2*cco***	**P*cco***	**P*ccoh***	**TS2*ccb***	**P*ccb***	**TS2*ins***	**P*ins***
OH	0.0	0.0	0.0	0.0	0.0	0.0	0.0	0.0	0.0	0.0	0.0
OMe	-1.1	-3.6	-3.2			-5.3		-1.7	-3.2	-4.3	-2.7
OPh	6.5	8.1				9.0		7.6	8.1	8.2	8.1

Table S48. CPCM-DLPNO-CCSD(T)/def2-TZVPP//PCM-B3LYP/6-31+G(d,p) (LANL2DZ for Re) relative Gibbs energies, in kcal/mol, of all the species found for each nucleophilic ligand containing a phosphorus atom referred to the analogous ones obtained for the PH_2 ligand in the reaction between [ReY(CO)$_3$(bipy)] (bipy = 2,2'-bipyridine) and methyl propiolate (HMAD, HC≡CCO$_2$Me).

Y	**TS1**	**I1**	**TS1'**	**I1'**	**TS2*cco***	**P*cco***	**P*ccoh***	**TS2*ccb***	**P*ccb***	**TS2*ins***	**P*ins***
PH_2	0.0	0.0	0.0	0.0	0.0	0.0	0.0	0.0	0.0	0.0	0.0
PHMe	3.3	-9.4	-8.4	-8.9	-8.4	-9.0	-4.6	-7.7	-6.8	-13.1	-5.2
PMe_2	-10.2	-15.8	-15.9	-16.0	-14.8	-17.4		-16.1	-16.0	-20.8	-11.0
PHPh	-10.0	-4.7	-4.9	-5.6	-5.3	-7.5	-4.5	-4.1	-3.3	-16.7	-4.4
PPh_2	-10.1	-8.1	-10.4	-9.7	-8.9	-9.4		-9.1	-10.5	-14.2	-3.7
PMePh	-8.9	-10.2	-10.7	-10.6	-11.3	-11.7		-9.9	-10.5	-17.0	-8.0

Table S49. CPCM-DLPNO-CCSD(T)/def2-TZVPP//PCM-B3LYP/6-31+G(d,p) (LANL2DZ for Re) relative Gibbs energies, in kcal/mol, of all the species found for each nucleophilic ligand containing a sulfur atom referred to the analogous ones obtained for the SH ligand in the reaction between [ReY(CO)$_3$(bipy)] (bipy = 2,2'-bipyridine) and methyl propiolate (HMAD, HC≡CCO$_2$Me).

Y	**TS1**	**I1**	**TS1'**	**I1'**	**TS2*cco***	**P*cco***	**P*ccoh***	**TS1*b***	**I1*b***	**I1'*b***	**TS2*ccb***	**P*ccb***	**TS2*ins***	**P*ins***
SH	0.0	0.0	0.0	0.0	0.0	0.0	0.0	0.0	0.0	0.0	0.0	0.0	0.0	0.0
SMe	-9.6	-8.0			-8.2	-10.1		-7.0	-8.4	-9.8	-9.4	-10.6	-7.0	-6.7
SPh	-1.9	-3.8			-3.9	-6.7		-2.4	-3.1	-3.6	-4.8	-4.8	-4.3	-3.3

Table S50. CPCM-DLPNO-CCSD(T)/def2-TZVPP//PCM-B3LYP/6-31+G(d,p) (LANL2DZ for Re) relative Gibbs energies, in kcal/mol, of the species with the nucleophilic ligands PH_2, PHMe, and PHPh referred to the analogous ones with the nucleophilic ligands NH_2, NHMe, and NH*p*Tol, respectively.

	**TS1**	**I1**	**TS1'**	**I1'**	**TS2*cco***	**P*cco***	**P*ccoh***	**TS2*ccb***	**P*ccb***	**TS2*ins***	**P*ins***
PH_2-NH_2	9.1	9.9	6.1	7.7	9.2	8.1	25.2	5.1	5.6	23.0	16.0
PHMe-NHMe	13.7	-0.3	1.9	1.0	5.2	3.5	28.0	1.7	3.0	12.7	12.9
PHPh-NH*p*Tol	-4.8	-9.6	-7.3			-7.2	16.9	-9.6	-7.7	-2.9	4.9

Table S51. CPCM-DLPNO-CCSD(T)/def2-TZVPP//PCM-B3LYP/6-31+G(d,p) (LANL2DZ for Re) relative Gibbs energies, in kcal/mol, of the species with the nucleophilic ligands SH, SMe, and SPh referred to the analogous ones with the nucleophilic ligands OH, OMe, and OPh, respectively.

	**TS1**	**I1**	**TS1'**	**I1'**	**TS2*cco***	**P*cco***	**P*ccoh***	**TS2*ccb***	**P*ccb***	**TS2*ins***	**P*ins***
SH-OH	5.7	6.1			6.1	5.9	9.4	4.6	5.6	10.8	11.3
SMe-OMe	-2.8	1.7	1.3			1.1		-3.1	-1.8	8.1	7.3
SPh-OPh	-2.7	-5.8				-9.8		-7.8	-7.3	-1.7	-0.1
